# The human tRNA-guanine transglycosylase displays promiscuous nucleobase preference but strict tRNA specificity

**DOI:** 10.1093/nar/gkab289

**Published:** 2021-05-01

**Authors:** Claire Fergus, Mashael Al-qasem, Michelle Cotter, Ciara M McDonnell, Emiliano Sorrentino, Franciane Chevot, Karsten Hokamp, Mathias O Senge, John M Southern, Stephen J Connon, Vincent P Kelly

**Affiliations:** School of Biochemistry and Immunology, Trinity Biomedical Sciences Institute, Trinity College Dublin, The University of Dublin, 152–160 Pearse Street, Dublin 2, Ireland; School of Biochemistry and Immunology, Trinity Biomedical Sciences Institute, Trinity College Dublin, The University of Dublin, 152–160 Pearse Street, Dublin 2, Ireland; School of Chemistry, Trinity Biomedical Sciences Institute, Trinity College Dublin, The University of Dublin, 152–160 Pearse Street, Dublin 2, Ireland; School of Chemistry, Trinity Biomedical Sciences Institute, Trinity College Dublin, The University of Dublin, 152–160 Pearse Street, Dublin 2, Ireland; School of Chemistry, Trinity Biomedical Sciences Institute, Trinity College Dublin, The University of Dublin, 152–160 Pearse Street, Dublin 2, Ireland; School of Chemistry, Trinity Biomedical Sciences Institute, Trinity College Dublin, The University of Dublin, 152–160 Pearse Street, Dublin 2, Ireland; School of Genetics and Microbiology, Trinity College Dublin, The University of Dublin, Dublin 2, Ireland; School of Chemistry, Trinity Biomedical Sciences Institute, Trinity College Dublin, The University of Dublin, 152–160 Pearse Street, Dublin 2, Ireland; School of Chemistry, Trinity Biomedical Sciences Institute, Trinity College Dublin, The University of Dublin, 152–160 Pearse Street, Dublin 2, Ireland; School of Chemistry, Trinity Biomedical Sciences Institute, Trinity College Dublin, The University of Dublin, 152–160 Pearse Street, Dublin 2, Ireland; School of Biochemistry and Immunology, Trinity Biomedical Sciences Institute, Trinity College Dublin, The University of Dublin, 152–160 Pearse Street, Dublin 2, Ireland

## Abstract

Base-modification can occur throughout a transfer RNA molecule; however, elaboration is particularly prevalent at position 34 of the anticodon loop (the wobble position), where it functions to influence protein translation. Previously, we demonstrated that the queuosine modification at position 34 can be substituted with an artificial analogue via the queuine tRNA ribosyltransferase enzyme to induce disease recovery in an animal model of multiple sclerosis. Here, we demonstrate that the human enzyme can recognize a very broad range of artificial 7-deazaguanine derivatives for transfer RNA incorporation. By contrast, the enzyme displays strict specificity for transfer RNA species decoding the dual synonymous NAU/C codons, determined using a novel enzyme-RNA capture-release method. Our data highlight the broad scope and therapeutic potential of exploiting the queuosine incorporation pathway to intentionally engineer chemical diversity into the transfer RNA anticodon.

## INTRODUCTION

**Figure 1.**
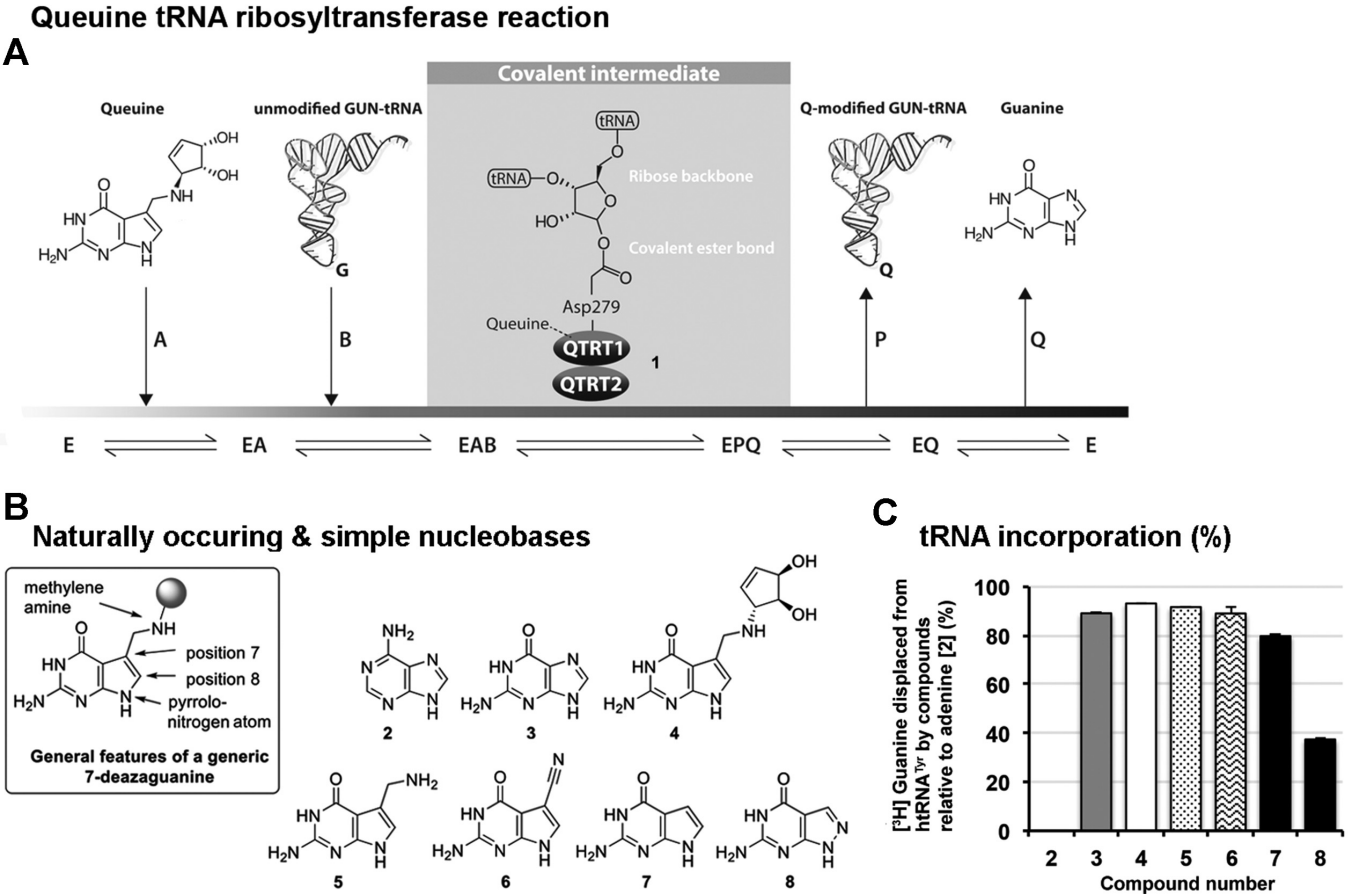
The QTRT enzyme recognizes 7-deazaguanine derivatives as substrates. (**A**) The QTRT catalyzed ribosyltransferase reaction removes a nuclear encoded guanine base from position 34 of G_34_U_35_N_36_ tRNA and inserts the queuine micronutrient in its place, in an energy-independent, base-for-base exchange reaction. (**B**) Structure of adenine and simple deazaguanine derivatives used in tRNA incorporation assays. (**C**) Nucleobases (50 μM) were assessed for their ability to displace [^3^H] Guanine from pre-labeled tRNA^Tyr^ (10 μM) catalyzed by the QTRT enzyme (100 nM). Data are mean ± SD (*n* = 3). Representative of two independent experiments.

To date, almost 100 base modifications have been documented to occur in eukaryotic tRNA (1), the greatest variety of which are found at positions 34 and 37 of the anticodon loop (2). The queuosine (Q) modification at position 34 is the only example of an exogenously supplied RNA modification in eukaryotes; this relies upon the supply of queuine base from eubacterial species (Figure 1A). In humans and other metazoans, the queuine micronutrient is salvaged from ingested food and the gut microbiome. Subsequently, it is inserted into tRNA by the unique action of the queuine tRNA ribosyltransferase (QTRT) enzyme—also frequently referred to as tRNA guanine transglycosylase (TGT)—a complex of two related proteins in eukaryotes comprising a catalytic QTRT1 subunit and a noncatalytic QTRT2 partner (3,4).

During the catalytic process, the QTRT enzyme first binds queuine, followed by tRNA, which results in the displacement of the nuclear-encoded guanine base at position 34 of the tRNA and the formation of a covalent intermediate between the tRNA molecule and aspartate 279 of the QTRT1 catalytic subunit (5). The enzyme-catalyzed reaction of this intermediate with bound queuine results in the release of Q-modified tRNA (Q-tRNA) and free guanine base. The uniqueness of the 7-deazaguanine scaffold in biology, which is limited to queuine related compounds and antibiotic molecules (6), and the singular nature of the QTRT enzymatic pathway raises the tantalizing possibility of utilizing this enzyme to install a variety of artificial nucleobases into the anticodon loop of tRNA. Ostensibly, this approach could be expected to influence protein translation, given the known importance of position 34 modification in dynamic modulation of translation rate and accuracy (7–9).

In line with this reasoning, we previously developed a series of artificial queuine analogues and identified a novel 7-deazaguanine derivative (NPPDAG) as an effective QTRT substrate capable of bringing about a remarkable recovery of clinical symptoms in an animal model of multiple sclerosis, when incorporated into tRNA (10). Analogue substitution of guanine at position 34 was found to limit T-cell proliferation *in vitro*, curtail the T-helper (Th)1 and Th17 response and modulate the production of the signature cytokines in both the periphery and the central nervous system. Notably, despite the expected reduction in molecular and cellular immune responses, therapeutic administration of NPPDAG to the animals re-established the expression of genes associated with neural repair and regeneration in diseased animals (10).

The possibility that queuine may be incorporated into RNA species apart from tRNA has not been extensively explored in eukaryotic species. Microinjection of yeast aspartyl tRNA into the oocytes of *Xenopus laevis* found that the specificity determinants are largely limited to a U_33_G_34_U_35_ sequence positioned within a 7-base anticodon loop of an intact tRNA molecule (11,12). In addition, replacing the normal anticodon of either yeast arginyl or leucyl tRNA with a trinucleotide GUC sequence resulted in these chimeric tRNA becoming substrates for queuine incorporation (11). In the case of the eubacterial enzyme, substrates have been shown to include a nonphysiological tRNA dimer (13), the T-arm of an *in vitro* transcribed yeast phenylalanyl tRNA (14), a uracil-containing DNA stem loop (15), and an mRNA transcript encoding the *VirF* virulence factor from *Shigella flexneri* (16). Other studies on the eubacterial enzyme have exploited a tritiated version of preQ_1_ (a precursor molecule for Q-tRNA biosynthesis) that resulted in the labeling of a number of non-tRNA species in *Escherichia coli;* the identity of which was not determined (17). In light of the significant number of noncoding RNA species with catalytic and structural function in the human genome (18,19), it was of interest to determine if additional undiscovered substrates for the mammalian QTRT enzyme may exist.

A review of nucleobase specificity identified pterins as reasonable inhibitors of the QTRT reaction and identified the amino nitrogen atoms at the 2 and 9 position, and the oxygen atom at the 6 position (purine numbering) as being important for recognition by the enzyme (20). By examining the incorporation of various substrates into the tRNA of intact L-M cells, the same study identified queuine, dihydroqueuine, 7-aminomethyl-7-deazaguanine and 7-deazaguanine as being irreversibly incorporated, whereas the exchange of guanine and 8-azaguanine was reversible—revealing a possible role for the carbon atom at the 7 position in rendering the modified tRNA essentially inert to the action of QTRT (20). Nishimura and coworkers previously examined the impact of a limited number of aliphatic and aromatic substitutions in the 7-position and a sulfur atom at the 6 position on cell proliferation (i.e. antitumour activity) and tRNA incorporation rate (21,22)

Here, we show that the QTRT enzyme has a broad ability to both recognise and incorporate 7-deazaguanine compounds harbouring a range of substitutions at the 7-position. This is in stark contrast to the high specificity that the enzyme shows towards RNA recognition, where a strict requirement is observed for mitochondrial and cytoplasmic tRNA that belong to the G_34_U_35_N_36_ family (N represents any of the canonical bases), which are responsible for decoding the dual synonymous NAU and NAC codons. This variance in substrate specificity allows the potential for a unique medicinal chemistry approach to modulate protein translation through the supply of artificial mimetics based on the exogenously derived queuine micronutrient.

## MATERIALS AND METHODS

### Recombinant protein production

BL21(DE3) tgt::Km_r_*E. coli* cells (3) were transformed with pET15b plasmid containing codon optimized human QTRT1 cDNA to produce an N-terminal His-tag fusion (His-hQTRT1), or a pCDF-1b plasmid containing codon optimized human QTRT2 cDNA encoding an N-terminal StrepII-SUMO (StrepII-hQTRT2) fusion or a C-terminal StrepII (hQTRT2-StrepII) fusion (Supplementary Data S1). Cells were grown in Terrific broth (4 L) in a shaker-incubator at 37°C until an OD_600_ of 0.8, the temperature decreased to 18°C, 0.5 mM IPTG added, the cells grown overnight at 18°C, then collected by centrifugation (3500 × *g*) for 10 min at 4°C. Cell pellets were resuspended in ice-cold binding (BD) buffer (50 mM NaH_2_PO_4_, 300 mM NaCl, 10% glycerol, pH 7.4) with complete EDTA-free protease inhibitor cocktail (Roche) and lysed using a French-Press. Samples were centrifuged (17 500 × *g*) for 20 min, the supernatant retained and 1 μg/ml DNase I (Ambion) added. After 30 min at 4°C, the supernatant was passed through a Nalgene 0.45 μm syringe filter. In the case of His-hQTRT1, 5 mM imidazole was added to the extract, which was loaded onto a pre-packed 1 ml HisTrap™ HP column (GE Healthcare). The column was washed successively with BD Buffer containing 5, 30, 50 and 80 mM imidazole before protein was eluted with 250 mM imidazole solution. StrepII-hQTRT2 and hQTRT2-StrepII extract was dialysed in BD Buffer (pH 8.0), loaded twice through a 2 ml bed volume of Streptactin Superflow Plus resin (Qiagen, 50% w/v solution). The resin was washed twice with BD Buffer (30 ml) and the protein eluted in buffer containing 2.5 mM desthiobiotin. PD-10 columns were used to remove imidazole and desthiobiotin from purified protein preparations. Purified proteins were stored at -20°C in 50% glycerol.

### Compound synthesis

The synthesis of the 7-deazaguanine derivatives and NMR spectroscopic analyses are described in the supporting information (Supplementary Data S2 and S3).

### Charging of tRNA with natural and artificial nucleobase

Prelabeling of *in vitro* transcribed human tyrosyl tRNA (htRNA^Tyr^; 4) with [8–^3^H] guanine (specific activity is 21.2 Ci/ mmol; Moravek Inc.), [methylene-^3^H] queuine (7 Ci/ mmol; Moravek Inc.) or non-radiolabeled nucleobases was carried out in RBT buffer (50 mM Tris-HCl pH 7.5, 20 mM NaCl, 5 mM MgCl_2_, 2 mM DTT) containing 10 μM of htRNA^Tyr^, 700 nM His-hQTRT1, 700 nM hQTRT2-StrepII, and either 200 nM [^3^H] guanine, 200 nM [^3^H] quenine or 50 μM of non-labeled natural or artificial nucleobase to a final volume of 500 μl. The reaction was incubated for 1 h at 37°C, followed by the addition of the equal volume of (1:1) acid-phenol:chloroform (pH 4.5), the solution was mixed well and centrifuged at 16 000 × *g* for 5 min. The upper aqueous phase was removed to a new tube and the pre-charged tRNA precipitated by the addition of 0.1 volume of 3 M sodium acetate and two volumes of ethanol. The sample was incubated overnight at -20°C, before the tRNA was pelleted at 16 000 × *g* for 20 min. The pellet was washed with ice-cold 70% ethanol, dried and the tRNA resuspended in nuclease-free water and the concentration measured at *A*_260_ spectrophotometrically.

### tRNA base incorporation assays

Evaluating novel compounds as substrates for tRNA incorporation was performed by the displacement of [^3^H] guanine from pre-charged htRNA^Tyr^. Each reaction contained 100 nM His-hQTRT1, 100 nM hQTRT2-StrepII, 50 μM nucleobase and 10 μM pre-labeled htRNATyr in RBT buffer. Assays were performed for 30 min at 37°C, quenched with 2.5 ml ice-cold 10% TCA, and placed on ice for one hour. Precipitated tRNA was collected onto GF/C glass fiber filter disks (GE Healthcare Whatman™) by vacuum manifold and each disk washed with 40 ml ice-cold 5% TCA followed by 5 ml of ice-cold 95% ethanol. The filters were dried and placed in 10 ml of Ecoscint A (National Diagnostics) and counted by scintillation. Adenine served as a negative control as it is not a substrate of the QTRT enzyme (5).

### tRNA base displacement assays

To examine the ability of guanine or queuine to displace natural and artificial 7-deazaguanine compounds from tRNA, the tRNA was first pre-charged with non-labeled bases and the ability to replace these with [^3^H] guanine or [^3^H] queuine examined. Each reaction contained 100 nM His-hQTRT1, 100 nM hQTRT2-StrepII, 10 μM pre-labeled htRNA^Tyr^ and either [^3^H] guanine (200 nM) or [^3^H] queuine (200 nM) in RBT buffer over a period of 30 min or up to 24 h at 37°C. Samples were captured on filter paper and scintillation counted as described above.

### Queuine incorporation into in vitro synthesized RNA and RNA isolated from MDA-MB-231 cells

*In vitro* transcribed htRNA^Tyr^, synthetic stem loops (Eurofins Genomics) of human aspartyl tRNA (CCCCGCCUGUCACGCGGGA) or arginyl tRNA (ACUGGCCUCCUAAGCCAGG) or small (<200 nucleotides) and large RNA (>200 nucleotides) were examined as substrates for the human QTRT enzyme. Stem-loops were heated to 60°C for 3 min and cooled slowly at 1°C per minute to ensure proper folding. Small RNA (<200 nucleotides) and large RNA (>200 nucleotides) were isolated from MDA-MB-231 cells grown in serum-free UltraCulture medium (Lonza; 23) by mirVana kit (Ambion). Reactions of htRNA^Tyr^ (500 pmol), stem-loop (500 pmol), small RNA (1.5 μg) or large RNA (15 μg) were incubated in RBT buffer (150 μl) with 30 pmol [^3^H] queuine, 1.5 μg His-QTRT1 and/or 1.5 μg StrepII-QTRT2 for 90 min at 37°C. Reactions were stopped by loading on a 1 ml pre-swollen DEAE cellulose spin column (pre-equilibrated with 200 mM Tris-HCl until the pH was stabilised at 7.5) and centrifuged at 0.1 × *g* for 10 s. The sample was re-loaded onto the same DEAE spin column five times to allow maximum binding of RNA. The column was then washed with 8 × 250 μl of wash buffer (20 mM Tris-HCl, pH 7.5, 10 mM MgCl_2_, 200 mM NaCl). The bound tRNA was eluted with 4 × 250 μl of elution buffer (20 mM Tris-HCl pH 7.5, 10 mM MgCl_2_, 1 M NaCl) and the eluted RNA analyzed by liquid scintillation counting. Heat inactivated enzyme was included in the assays to ensure that the incorporation into RNA was due to the active recombinant enzyme complex. Data are means ± SD (*n* = 3). Representative of two independent experiments.

### 5′ end-labeling of RNA isolated from MDA-MB-231 cells

The 5′-termini of RNA were labeled with γ-^32^Phosphate from [γ- ^32^P] ATP or non-radiolabeled phosphate from ATP. RNA was dephosphorylated by addition of eubacterial alkaline phosphatase (10U, FastAP, Thermofisher) in a 40 μl reaction of 10 mM Tris-HCl, pH 8; 5 mM MgCl_2_; 100 mM KCl; 0.02% Trition X-100; 0.1 mg/ml BSA at 37°C for 30 min followed by 75°C for 3 and 10 min on ice. The 5′-phosphate-end-labeling reaction was set up in 60 μl reaction containing the dephosphorylated RNA, 2U T4 polynucleotide kinase (PNK) and either 0.1 μM [γ- ^32^P] ATP (3000Ci/mmol; 10mCi/mL; Perkinelmer) or 25 μM nonradiolabeled ATP in PNK buffer (7 mM Tris-HCl, pH 7.6; 1 mM MgCl_2_; 0.5 mM DTT) at 37°C for 1 h. Unreacted ATP was removed and RNA recovered (Norgen RNA clean-up kit).

### Denaturing electromobility shift assay

Reactions (20 μl) were set up in RBT buffer with 1 μg His-hQTRT1, 1 μg StrepII-hQTRT2, 25 μM 9-deazguanine and 100 pmol of either aspartyl or arginyl stem loop, and incubated for an hour at 37°C. The samples were mixed with 5 μl of 4× LDS sample buffer (Invitrogen), loaded onto a 10% Bis-Tris gel (Invitrogen) and electrophoresed in 1× MOPS buffer (Invitrogen) at 200 V for 1 h at 4°C. The gel was stained with InstantBlue Coomassie stain (Sigma-Aldrich) for 30 min and washed with distilled water overnight.

### Capture-release method for NGS

Streptactin superflow plus beads (Qiagen; 15 μl packed volume) were pre-incubated with purified StrepII-QTRT2 (6 μg) for 1 h at 37°C in 100 μl ribosyltransferase (RBT) buffer (50 mM Tris-HCl, pH 7.5, 20 mM NaCl, 5 mM MgCl_2_, 2 mM DTT) containing 10% glycerol. The resin was washed with 1 ml RBT buffer containing 10% glycerol. RNA was isolated from MDA-MB-231 cells, neo-natal mouse liver (P0.5 pups) or spleen cells from EAE diseased mice at a clinical score of 2 (10) by mirVana kit (Ambion) and 4 μg added to the QTRT-bound Streptactin resin in 200 μl RBT buffer containing His-QTRT1 (6 μg), 9-deazaguanine (4.68 nmol), poly(U) and arginyl stem-loop competitors (1.56 nmol each) and SUPERase-In (10 U/μl; ThermoFisher). Reactions were incubated in thermomixer for 1 h at 37°C, 900 rpm. Resin was washed eight times with 1 ml of RBT buffer containing 30 μM 9-deazaguanine and 0.01% Triton X-100 before dissociation of the complex with 100 μl of denaturation (DN) buffer (50 mM Tris-HCl, pH 7.5, 300 mM NaCl, 4 M urea). A 20 μl volume of Mag Sepharose^®^ Ni beads (GE healthcare, 5% slurry in DN buffer) was added to the denaturing buffer and incubated for 30 min with shaking. Beads were isolated on a magnet, washed eight times with 1 ml of DN buffer followed by six times with 1 ml non-denaturing (NDN) buffer (50 mM Tris-HCl, pH 7.5, 100 mM NaCl). For next-generation sequencing, covalently bound RNA was dephosphorylated with FastAP (Thermo Scientific) and 5′ phosphate end-labeled with T4 PNK (NEB). The complex was again washed eight times with 1 ml guanidine-HCl buffer (50 mM Tris-HCl, pH 7.5, 100 mM NaCl, 3.5 M Guanidine HCl, 0.5% Triton X-100) and six times with 1 ml NDN buffer. Covalently bound RNA was eluted in 10 μl 100 mM NaOH for 2 min, beads removed on a magnet, the RNA-containing solution immediately neutralized with 100 mM HCl, and RNA recovered (Norgen RNA clean-up kit). For next-generation sequencing, cDNA libraries were prepared with 4 μl of each sample according to the NEXTflex small RNA-seq library prep kit v2 protocol with the following modifications (i) 3′ adapter ligation was performed at 16°C overnight, (ii) 8 μl of adapter depletion solution was used in clean-up steps, (iii) reverse transcription was performed using Sunscript RT (Expedeon) with thermocycling: 44°C for 30 s and 65°C for 4 min for 15 cycles, followed by 95°C for 10 min, then 4°C and (iv) RNase H (5 U) was added post-PCR and the sample incubated at 37°C for 20 min, then 95°C for 10 min, then placed on ice. Following amplification, samples were PAGE and bead purified, and cDNA eluted. Each cDNA library (1 μl) was tested for concentration and quality (Agilent technologies Bioanalyser high sensitivity dsDNA chip kit), before sequencing on an Illumina MiSeq on a 65-cycle, unpaired run according to the manufacturers protocol.

### NGS and analyses

In BaseSpace (Illumina), reads were 3′ and 5′ adapter trimmed, low-quality reads removed (FastQ toolkit) and examined to ensure a Phred of 30 or greater (FastQC). Fastq files were uploaded to Galaxy, aligned to the *E. coli* (eschColi_K12) genome assembly (Bowtie2). Nonaligned reads were output to a new fastq file, 3′–CCA tails trimmed using a clip adapter tool and aligned to the human (hg19) or mouse (mm10) genome assemblies (Tophat2). The output bam file was used to (i) detect variations from the reference sequence with the help of mpileup (samtools) and to (ii) create a graph of genome coverage (BEDtools) for visualization through the UCSC genome browser. A custom Perl script was used to detect regions of consecutive coverage and compile statistics, such as the length of each region, as well as average coverage and highest coverage. Parameters were set to record only regions with a minimum read count coverage of 10 reads. The script also identified SNP transversions of g→t and c→a that occur in the regions covered, the position of the transversion, the total number of reads covering the transversion, the number actually containing the transversion, and the ratio of these. RPKM and TPM values were calculated using the detected regions as representatives of transcripts. Excel was used to organise the files into the genes covered and to plot read counts as a percentage of total reads. The processed data for each of the capture-release studies is provided (Supplementary Tables S1–S7).

### RTPCR quantification of Hrpt, aspartyl tRNA and Snord42b ncRNA

Spleens were recovered from 8-week-old, EAE-disease induced C57BL/6J female mice upon reaching a clinical score of 2 as described previously (10). A single cell suspension was plated in X-vivo 15 medium (Lonza) at 2 × 10^6^ cells/ml. Suspensions were re-stimulated with MOG_[33–55]_ (Genscript; 50 μg/ml) for 48 h before adding queuine base (200 μM) for a further 24 h. Total RNA was isolated from splencoytes using mirVana kit (Ambion), the tRNA deacetylated in 20 mM Tris-HCl, pH 9 at 37°C for 40 min and the RNA recovered (Norgen RNA clean-up kit). For the qRTPCR quantification of aspartyl tRNA and Snord42b transcript, 18 μg of RNA was used in the capture-release method described above, without dephosphorylation and 5′ phosphate end-labelling.

Quantitative RT-PCR using a stem-loop adapter was used for detection of mature aspartyl tRNA (24). tRNA-Asp Stem-loop adapter (20 pmol, IDTdna) was annealed with 9 μl NaOH-eluted RNA in annealing buffer (40 mM Tris-HCl, pH 8, 5 mM EDTA, 100 mM MgCl_2_) at 37°C for 20 min followed by ligation in 1× T4 RNA ligase 2 reaction buffer with T4 RNA ligase (2U, NEB) in a volume of 20 μl at 37°C for 1 h followed by 4°C overnight. Ligated RNA (4.5 μl) was incubated with 1 pmol tRNA-Asp RT primer (0.5 μl) and 5 nmol dNTPs (0.5 μl) at 65°C for 5 min and placed on ice. First strand cDNA synthesis was performed by addition of the RNA/primer mix to first-strand buffer (Thermofisher) containing 5 mM MgCl_2_, 0.1 M DTT, 4U SUPERase-In and 100U SuperScript III reverse transcriptase (ThermoFisher) in 10 μl volume with thermocycling: 16°C for 30 min, 50°C for 30 min, 85°C for 5 min. RNase H (1U, ThermoFisher) was added for 20 min at 37°C. qPCR was performed using Taqman qPCR mix containing 0.6 μl first strand cDNA, 1× Premix Ex Taq solution (Takara), 400 nM tRNA-Asp Taqman probe (ThermoFisher), 2 pmol each tRNA-Asp Forward and reverse primers and 0.5× ROX reference dye II (Takara) in 10 μl volume and thermocycling: 95°C for 10 min, followed by 40 cycles of 95°C for 15 s and 63°C for 1 min).

A custom Taqman assay (ThermoFisher) was used to quantitate Snord42b transcript. NaOH-eluted RNA (1.5 μl) was incubated with Snord42b RT primer and 10 nmol dNTPs at 65°C for 5 min and placed on ice. First strand cDNA synthesis was performed by addition of the RNA/primer mix to 1× Taqman RT buffer containing 0.1M DTT, 4U SUPERase-In and 35U Sunscript reverse transcriptase (Expedeon) with thermocycling: 16°C for 30 min, 65°C for 30 min, 90°C for 5 min. RNase H (1U, ThermoFisher) was added for 20 min at 37°C. qPCR was performed using Taqman qPCR mix containing 2.22 μl first strand cDNA, 1× custom snord42b Taqman primer/probe mix and 1× Taqman Universal PCR Master Mix II in 10 μl volume and thermocycling: 50°C for 2 min, 95°C for 10 min, followed by 40 cycles of 95°C for 15 s and 60°C for 1 min.

Taqman assay (ThermoFisher) was used for detection HPRT transcript. Total RNA isolated from splenocytes (300 ng) was incubated with random hexamer primers (50 ng) and 10 nmol dNTPs at 65°C for 5 min and placed on ice. First strand cDNA synthesis was performed by addition of the RNA/primer mix to first-strand buffer (Thermofisher) containing 5 mM MgCl_2_, 0.1 M DTT, 4U SUPERase-In and 100U SuperScript III reverse transcriptase (ThermoFisher) with thermocycling: 25°C for 10 min, 50°C for 50 min, 85°C for 5 min. RNase H (1U, ThermoFisher) was added for 20 min at 37°C. qPCR was performed using Taqman qPCR mix containing 0.4 μl first strand cDNA, 1× HPRT Taqman primer/probe mix and 1× Taqman Universal PCR Master Mix in 10 μl volume and thermocycling: 50°C for 2 min, 95°C for 10 min, followed by 40 cycles of 95°C for 15 s and 60°C for 1 min).

## RESULTS

### 7-deazaguanine derivatives are substrates for tRNA incorporation by the QTRT enzyme

Our investigations began with examining the ability of the QTRT enzyme (**1**) to process both naturally occurring nucleobases and simple deazaguanines (Figure 1B). In the presence of the human QTRT, adenine (**2**, negative control) failed to displace [^3^H] guanine from pre-charged htRNA^Tyr^ (Figure 1C). Consistent with seminal work in this domain (20), we found that the natural substrates guanine (**3**) and queuine (**4**) almost quantitatively displaced [^3^H] guanine from the tRNA; as did the queuine biosynthetic precursors preQ_1_ (**5**—the natural substrate of the eubacterial TGT enzyme) and preQ_0_ (**6**). 7-Deazaguanine (**7**) has been reported to be a poor substrate for eukaryotic enzymes (20); however, at a 50 μM concentration (5-fold excess over tRNA) an incorporation level of 83% for this nucleobase was detected. By contrast, replacement of the C-H moiety with a nitrogen atom at position 8 (i.e. compound **8**) resulted in a dramatic reduction in the level of tRNA modification.

**Figure 2.**
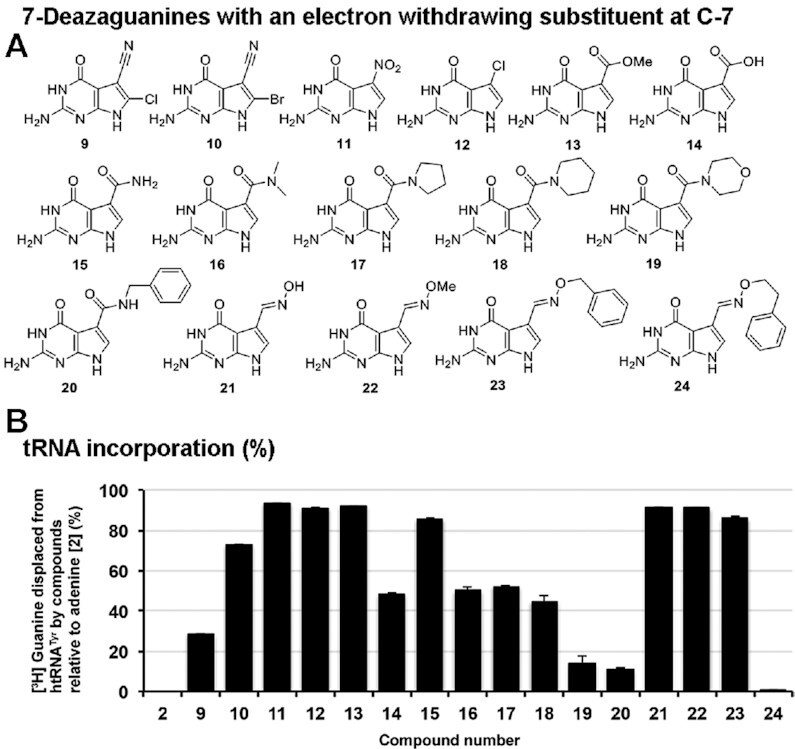
Substrate scope: influence of an electron-withdrawing group at position 7. (**A**) 7-deazaguanine nucleobases incorporating an electron-withdrawing substituent at the C-7 position. (**B**) Nucleobases (50 μM) were assessed for their ability to displace [^3^H] Guanine from pre-labeled tRNA^Tyr^ (10 μM) catalyzed by the QTRT enzyme (100 nM). Data are mean ± SD (*n* = 3). Representative of two independent experiments.

Previously, it has been shown that the rate of the base exchange reaction catalyzed by the *E. coli* enzyme correlates with the p*K*_a_ at the pyrrolo nitrogen atom; indicating that the deprotonation of this position is at least partially rate-determining (25). Notably, the human enzyme has recently been shown to operate via a ‘sequential bi-bi’ mechanism (5), which is distinct from the ‘ping-pong’ reaction described for the eubacterial TGT (26). Little is known about the influence of groups which would acidify the pyrrolo N-H group on substrate competency using the human protein. Thus, a range of 7-deazaguanines with electron-withdrawing moieties were evaluated as substrates for human TGT (Figure 2).

Relative to preQ_0_ (**6**)—which already features a nitrile substituent at C-7—analogues with halogen atoms installed at position 8 (i.e. **9** and **10**) were considerably less efficiently incorporated. However, it is surprising that these compounds could serve as substrates, given that the steric bulk at this position would encumber the nucleophile during the *N*-ribosylation step, which is suggested to be a substantial contributor to the overall rate of the reaction process. Our studies showed that the cyano functionality associated with **6** could be exchanged for either a nitro group or a chlorine atom (i.e. **11** and **12** respectively) without compromising the efficacy of tRNA incorporation, while the corresponding methyl ester-substituted nucleobase (i.e. **13**) proved a marginally inferior substrate. The analogous carboxylic acid **14** was only capable of displacing 43% of the [^3^H] guanine from the tRNA: at physiological pH this would be ionised to the carboxylate ion—a weak electron donor—which may explain in part why this molecule is a poorer substrate than even the unsubstituted 7-deazaguanine **7**. Amides are less electron-withdrawing than esters—**15** is an inferior substrate to **13**—and a clear trend exists of diminishing substrate competency as the steric bulk of the amide is increased (i.e. **15**–**20**). Oximes **21–23** appear to be well tolerated by the enzyme, while increasing the size of the *O*-substituent associated with **23** by one methylene unit (i.e. **24**) proved very deleterious to tRNA incorporation.

**Figure 3.**
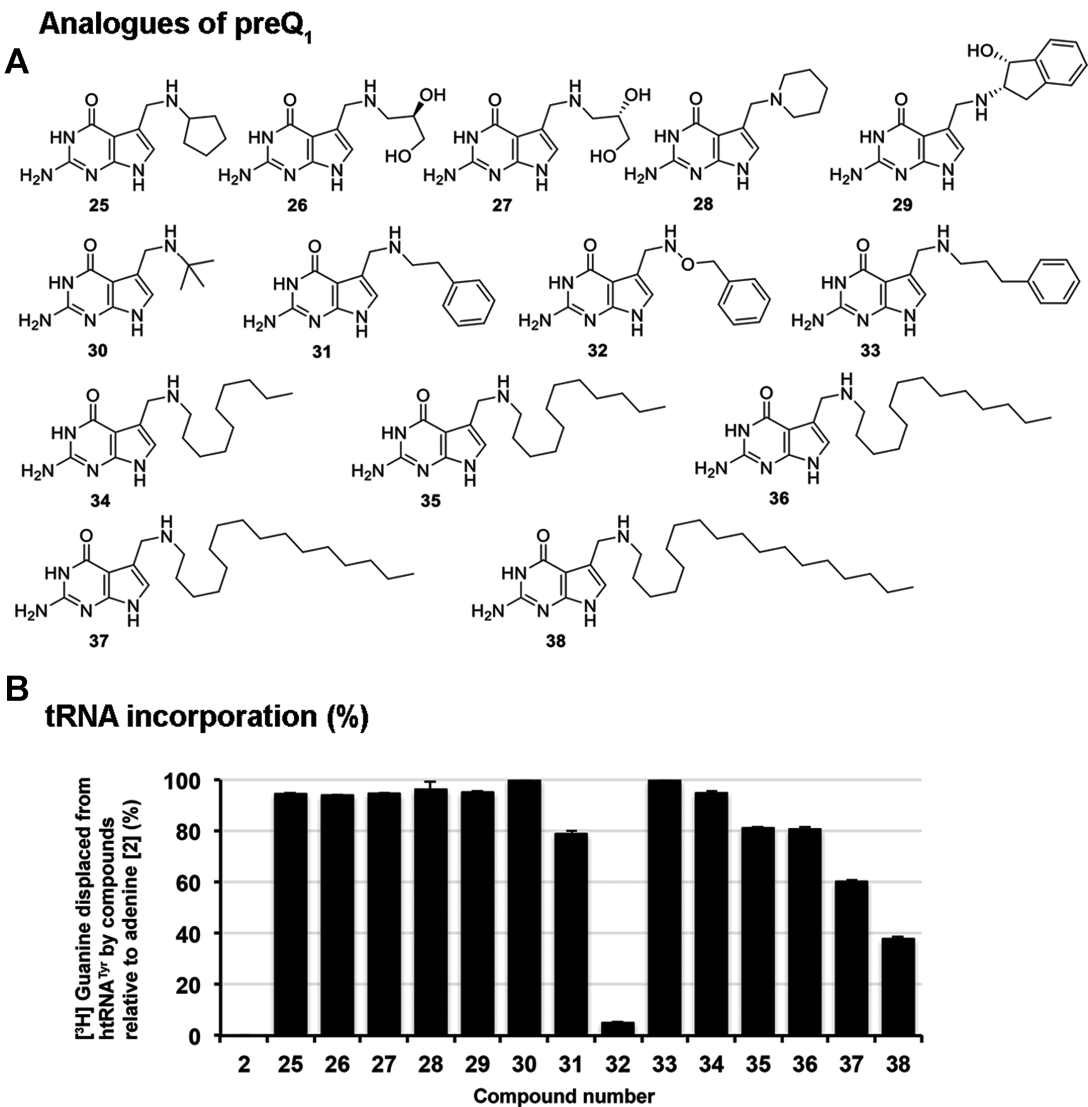
Substrate scope: influence of substitution of the methylene amine of preQ_1_. (**A**) Derivatives of preQ_1_ with substitutions at the methylene amine. (**B**) Nucleobases (50 μM) were assessed for their ability to displace [^3^H] Guanine from pre-labeled tRNA^Tyr^ (10 μM) catalyzed by the QTRT enzyme (100 nM). Data are mean ± SD (*n*=3). Representative of two independent experiments.

Nucleobases which retain the C-7 methylene amine unit (previously posited to be a key recognition element i.e. derivatives of queuine and preQ_1_—**4** and **5**, respectively) were next examined (Figure 3). Removal of all functionality associated with the queuine *N*-substituent save the 5-membered ring skeleton (i.e. **25**) resulted in a substrate which serves almost as well as queuine itself. Likewise, cleaving the ring structure but retaining the diol unit (either enantiomer—i.e. **26**–**27**) impacts incorporation only to a marginal extent. Compound **28**—a tertiary amine—serves as a comparably efficient substrate as either the primary (i.e. preQ_1_) or secondary amine (i.e. queuine) natural nucleobases. Likewise, conversion of preQ_1_ to a large *cis*-amino indanol analogue also leads to an efficient substrate **29**. *Tert*-butyl- and homobenzyl *N*-substituents (i.e. **30** and **31**, respectively) were reasonably well tolerated, however, replacement of the methylene unit adjacent to the aliphatic amine with an oxygen atom (i.e. **32**) almost completely prevented incorporation. Elongation of the side chain associated with **31** (i.e. **33**) led to a substrate well accepted by the enzyme – this is the compound named NPPDAG previously found to be highly active in a murine model of multiple sclerosis (10).

The ability of human TGT to modify tRNA with nucleobases equipped with reasonably large side chains such as **33** points to considerable space being available in the catalytic binding site of the enzyme. This is an intriguing observation from the perspective of introducing large artificial handles into the anticodon loop of tRNA. We were thus prompted to probe the limits of this enzyme promiscuity by preparing the small library of nucleobases **34**–**38** in which the side chain length has been incrementally increased from 10 carbon atoms to 18 carbon atoms in 2 carbon atom units. Somewhat surprisingly, all proved competent substrates under these conditions. Efficiency of incorporation steadily reduces with increasing chain-length; however, incorporation only slips below 50% using the very large C-18 substituent **38**. To put this into perspective—this C18 side chain alone is over 40% heavier than the entire preQ_1_ nucleobase. Clearly the enzyme can accommodate large substituents at this position.

**Figure 4.**
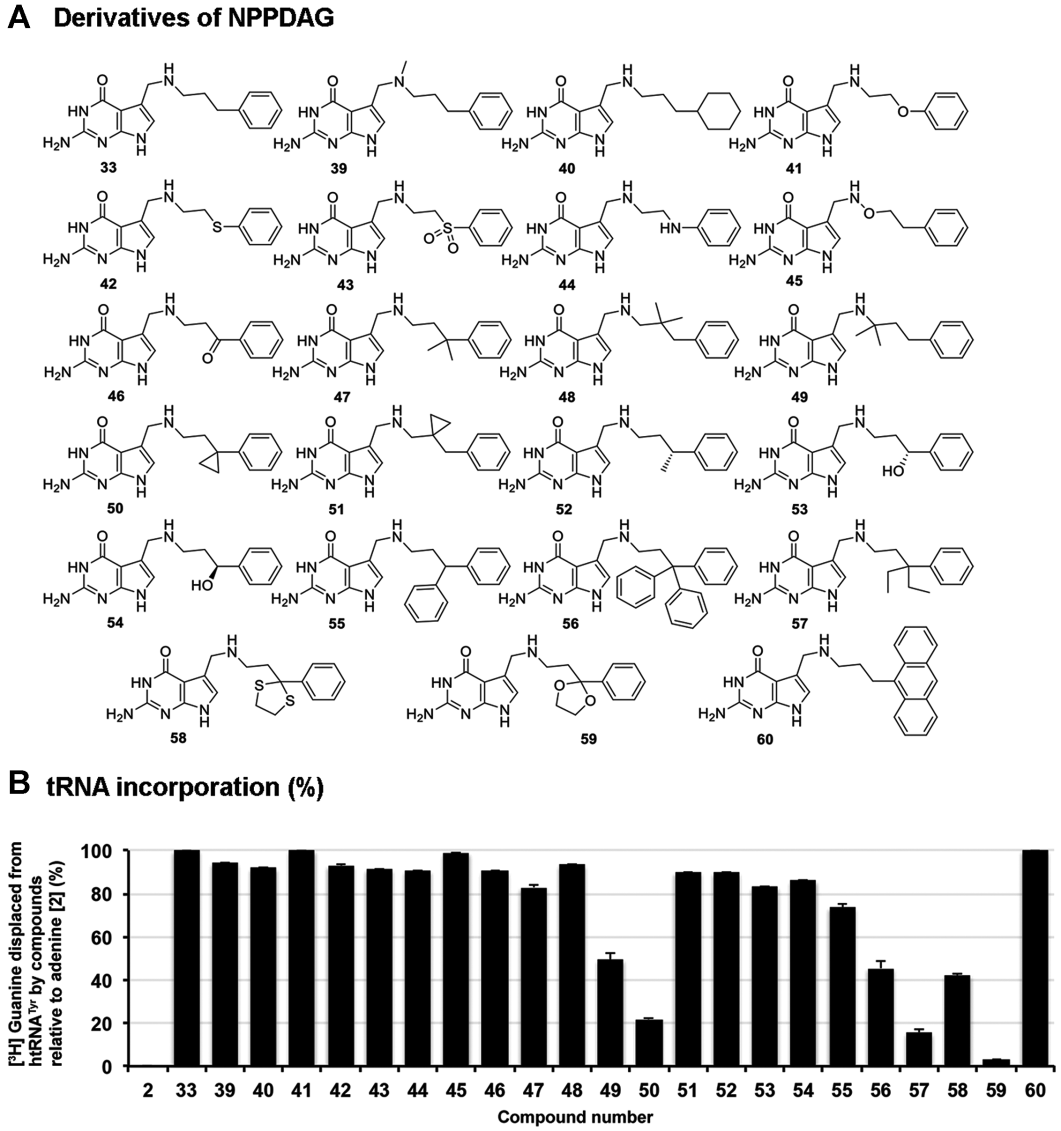
Substrate scope: influence of substitution of the artificial nucleobase NPPDAG. (**A**) Structures of NPPDAG derivatives. (**B**) Nucleobases (50 μM) were assessed for their ability to displace [^3^H] Guanine from pre-labeled tRNA^Tyr^ (10 μM) catalyzed by the QTRT enzyme (100 nM). Data are mean ± SD (*n*=3). Representative of two independent experiments.

Finally, the chemical space around the therapeutic candidate **33** was explored (Figure 4). Consistent with our earlier findings, neither *N*-methylation (i.e. **39**) nor saturation of the pendant aromatic ring (i.e. **40**) resulted in significant reductions in incorporation. Likewise, it proved possible to exchange the benzyl methylene unit with either an oxygen or a sulfur atom (i.e. **41** and **42**), sulfoxides or secondary amines (i.e. **43** and **44**) with minimal disruption to the reaction efficacy. Keeping the chain length constant but converting it to a hydroxylamine derivative such as **45** is also possible. Returning to the benzylic position: keto-functionality (i.e. **46**) is accepted, as is rigidification of the side chain through the introduction of a *gem*-dimethyl substituent (i.e. **47**)—although here a small but marked reduction in the level of tRNA modification was detected. Displacement of the *gem*-dimethyl unit to the middle of the side chain (i.e. **48**) restores substrate activity somewhat, however further displacement to adjacent to the methylene amine unit (i.e. **49**) resulted in a dramatic reduction in incorporation to 49%. The data show that the enzyme does not tolerate steric bulk next to the methylene amine of the molecule.

Nucleobase **50**—the cyclopropyl analogue of **47**—proved a surprisingly poor substrate, especially considering that with **51**—the cyclopropyl analogue of **48**—the enzyme can replace 89% of the radioactive guanine units at position **34** under these conditions. It therefore appears that the location of the cyclopropyl unit has a greater influence on substrate competency than does its inherent chemical properties. Replacement of one of the H-atoms of the benzylic unit associated with **33** with either a methyl- (i.e. **52**), hydroxy- (either enantiomer, **53** and **54**) or phenyl-group (i.e. **55**) led to less efficient substrates than **33**; howeve,r all were associated with significant levels of incorporation in the 76–83% range. To ascertain the ability of the enzyme to accept steric bulk at the benzylic position we synthesised several nucleobases where the benzylic carbon atom is quaternary. While an analogue substituted with the large trityl group (i.e. **56**) was within the scope of substrate acceptability (albeit with incorporation levels approximately half that of the parent molecule **33**), the diethyl variant of **33** (i.e. **57**) in addition to the thiolane and dioxolane derivatives **58**–**59**, served as very poor substrates. While ostensibly the enzyme can tolerate very large and very long methylene amine substituents, this remarkable sensitivity to steric bulk at a benzylic position at some distance from the nucleobase core (on a molecular scale) can best be rationalised through **33** and daughter molecules adopting a specific conformation in which the benzylic position is close to a key binding interaction in the rate-determining step of the incorporation reaction. Finally, even considerable augmentation of the steric requirement of the phenyl unit of **33** with a large electron-donating group (i.e. **60**) resulted in competent substrates.

### 7-Deazaguanine derivatives can be reversibly incorporated into tRNA by QTRT

**Figure 5.**
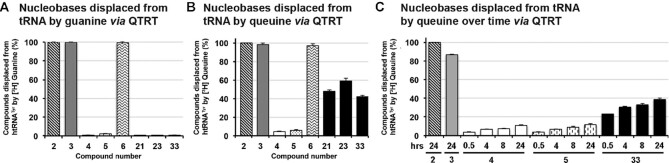
7-deazaguanine derivatives can be displaced from tRNA by QTRT. Evaluation of the ability of the QTRT enzyme (100 nM) to insert either; (**A**) [^3^H] guanine (200 nM) or (**B**) [^3^H] queuine (200 nM) into tRNA (10 μM) that had been pre-charged with natural or artificial nucleobases over a period of either 30 min or (**C**) 24 h. Data are mean ± SD (*n*=3). Representative of two independent experiments.

Previous studies using the eukaryotic rabbit reticulocyte's TGT enzyme and L-M cells determined that the incorporation of **4**, **5** and **7** into tRNA is irreversible in cells in culture, whereas both **3** and **8** could be replaced (20). The difference seen between **3** and **7** highlight the importance of the nitrogen atom at position 7 of the pyrrole ring in allowing the bi-directional reaction to occur. As it has not been determined how queuine may affect the reversibility, it was decided to examine if the human QTRT enzyme could displace tRNA-incorporated bases with either **3** or **4** as substrate (Figure 5).

In these reactions, the pre-labeled tRNA (10 μM) was supplied to the reaction at a 100-fold and 50-fold excess over the QTRT enzyme (100 nM) and [^3^H] guanine or [^3^H] queuine (200 nM), respectively, ensuring that the results are reflective of the catalytic competency of the enzyme, thereby allowing reversibility to be compared across substrates. As a control, **2** was included in the pre-labeling assays since it is neither a substrate nor an inhibitor of the eukaryotic enzyme (5,20) and therefore may be used to represent maximum displacement (set arbitrarily to 100%). It was observed (Figure 5A) that in the case of tRNA pre-labeled with either **3** or **6**, the human QTRT enzyme could displace the respective nucleobase with [^3^H] guanine, but the enzyme was incapable of replacing **5** in the anticodon loop with [^3^H] guanine. In this regard the human enzyme is similar to the eubacterial TGT (27). All 7-deazaguanine compounds, with the exception of **6**, could not be quantitatively displaced by [^3^H] guanine. Interestingly, under similar conditions it was found that both **4** and **5** could be displaced to a small degree by [^3^H] queuine, which could also exchange with the 7-deazaguanine compounds **21**, **23** and **33** to a greater (but still incomplete) extent (Figure 5B). A repeat of the [^3^H] queuine-mediated displacement study over a 24-h period demonstrated a time-dependent loss of **4**, **5** and **33**, albeit on a timeframe and concentrations not relevant to a physiological setting (Figure 5C). The moderate reduction in [^3^H] queuine incorporation into tRNA pre-labeled with **3** after 24 h incubation relative to 30 min is suspected to be the result of a small amount of RNase activity carried over from the initial labeling reaction.

### Small RNA are the principal nucleotide substrates of the human QTRT enzyme

The RNA substrate specificity of the QTRT enzyme has not been extensively characterized. Studies on the eubacterial enzyme suggest that a range of RNA substrates are compatible; even a modified DNA containing uracil in place of thymine bases could serve as a substrate (15). In the case of the mammalian QTRT enzyme the minimal recognition motif appears to be a UGU sequence in a seven-base oligonucleotide in an anticodon loop structure within an intact tRNA molecule (11,12).

**Figure 6.**
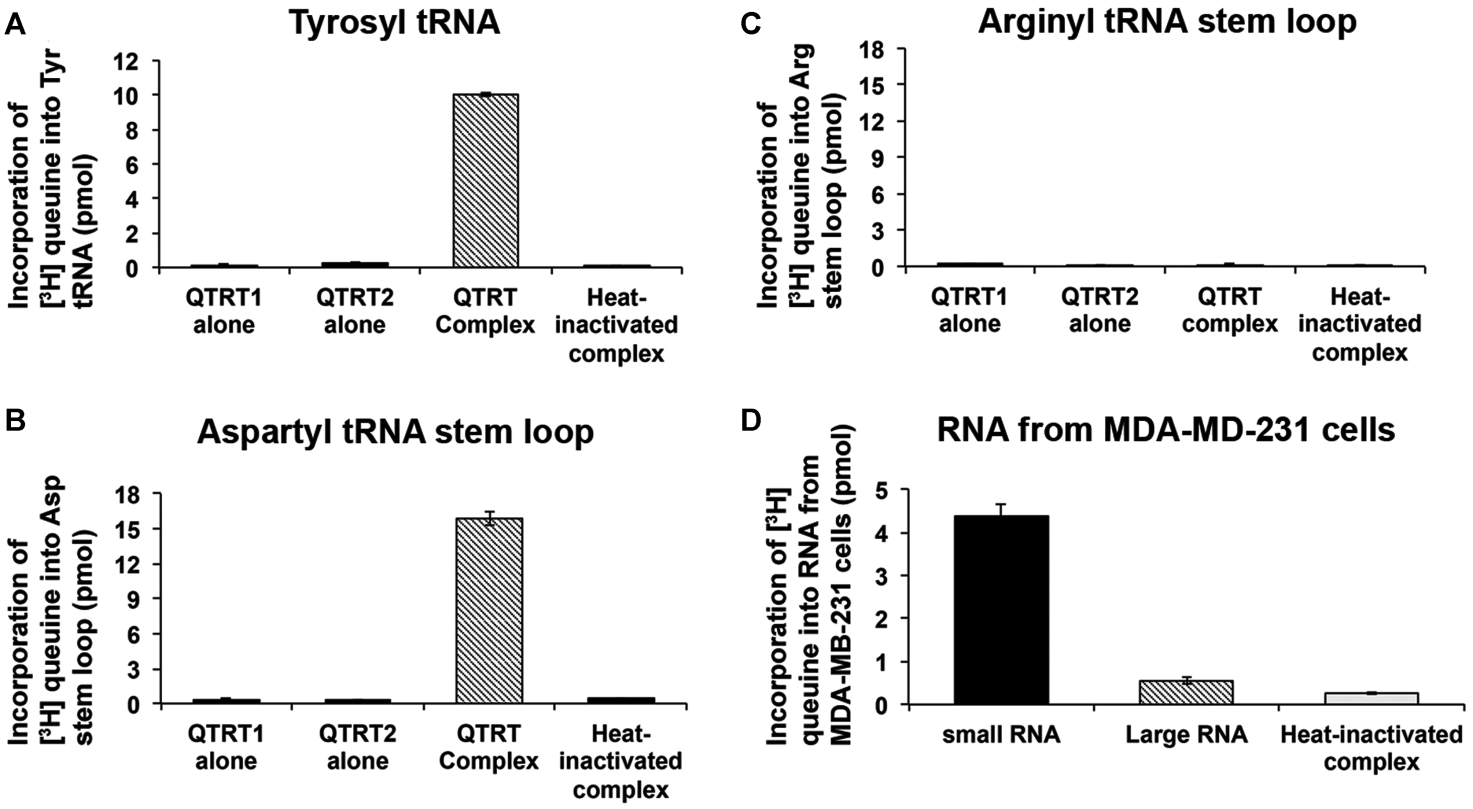
Small RNA are the principal nucleotide substrates of the QTRT enzyme. (**A**) *In vitro* transcribed tyrosyl tRNA, (**B**) synthetic aspartyl stem loop, and (**C**) synthetic arginyl step loop (all at 3.33 μM) or (**D**) 1.5 μg small RNA (<200 nucleotides) or 15 μg large RNA (>200 nucleotides) isolated from MDA-MB-231 cells, were incubated with 200 nM of [^3^H] queuine and QTRT enzyme (175 nM) in a final reaction volume of 150 μl for 90 min at 37°C. Reaction products were separated on a DEAE cellulose column and the eluted RNA evaluated for [^3^H] queuine incorporation by scintillation counting. Heat-inactivated QTRT enzyme complex served as negative control. Data are mean ± SD (*n*=3). Representative of two independent experiments.

Using the eukaryotic QTRT enzyme, reactions carried out with either *in vitro-*transcribed tyrosyl tRNA (Figure 6A) or a synthesized stem loop corresponding to aspartyl tRNA (Figure 6B) resulted a similar incorporation level of queuine base, whereas the non-GUN anticodon loop of arginyl tRNA failed to act as a substrate (Figure 6C). The results indicate that the tertiary structure of the tRNA molecule is dispensable for catalytic recognition by the eukaryotic QTRT enzyme. Given that the enzyme recognises relatively small, specific sequences of tRNA and that a large proportion of the genome is transcribed into RNA, there is a possibility that other RNA substrates for the QTRT enzyme could exist. To probe this supposition, RNA was extracted from human MDA-MB-231 breast cancer cells and fractionated into small (<200 nt) and large RNA (>200 nt) species. The ability of the hQTRT enzyme to incorporate radiolabeled queuine into both RNA fractions was examined, using a 10-fold excess of large RNA relative to small RNA (Figure 6D). It is notable that the vast majority of radiolabel is found in the small RNA sample.

### RNA capture-release strategy to identify RNA substrates of the QTRT enzyme

**Figure 7.**
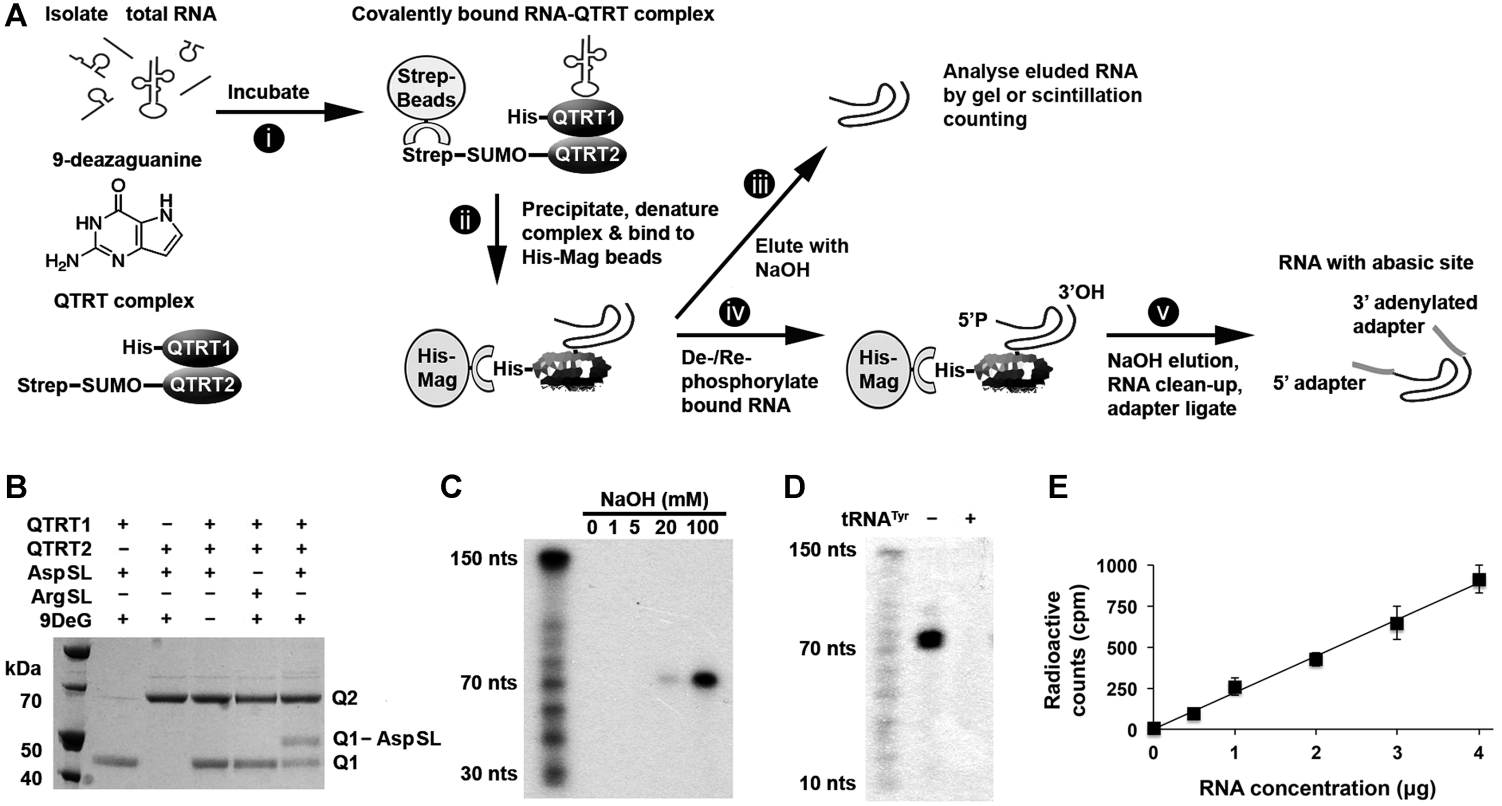
Capture-release strategy to identify RNA substrates of the QTRT enzyme. (**A**) Schematic of the covalent capture-release method for analysis of QTRT RNA substrates. (i) *In vitro* transcribed htRNA^Tyr^ or RNA isolated from cells or tissue was incubated with 9-deazaguanine and QTRT enzyme pre-bound to Streptactin beads (*via* the QTRT2 StrepII tag), to form a covalent RNA-QTRT enzyme intermediate. (ii) The RNA-QTRT enzyme intermediate was isolated using Streptactin beads, the complex washed, denatured in urea and the catalytic QTRT1 subunit (complexed to the RNA) separated from the enzyme intermediate using magnetic Ni^2+^ beads. (iii) The denatured QTRT1-bound RNA complex was washed, and the RNA released by NaOH or (iv) modified *in situ* to create 5′-phosphate and 3′-hydroxyl ends followed by (v) NaOH elution, RNA clean-up and adapter ligation for NGS. (**B**) A covalent intermediate was formed between the catalytic QTRT1 subunit (Q1) and a synthetic aspartyl stem loop (AspSL) but not an arginyl stem loop (ArgSL) in the presence of 9-deazaguanine (9DeG); In a 20 μl reaction, 200 pmol stem loop, 50 pmol QTRT1, 50 pmol QTRT2 and/or 1 nmol 9-deazaguanine were incubated for 1.5 h at 37°C, before analysis by denaturing electromobility shift assay. (**C**) *In vitro* transcribed htRNA^Tyr^ (6.24 pmol) or (**D**) Small RNA (1 μg) from MDA-MB-231 cells was 5′ end-labeled with ^32^P, re-isolated by spin column and processed by the covalent capture-release method to examine the effect of increasing concentrations of NaOH on ester bond breakage and RNA degradation or for the specificity of the RNA-QTRT enzyme interaction by the addition of excess non-labeled htRNA^Tyr^ competitor, respectively. Samples were neutralized with HCl, gel loading buffer added, the RNA denatured, and loaded onto a 10% TBE-urea polyacrylamide gel for subsequent exposure to autoradiographic film. (**E**) Small RNA from MDA-MB-231 cells was 5′ end-labeled with ^32^P, re-isolated by spin column, and increasing concentrations processed using 4 μg QTRT enzyme by the covalent capture-release method. Samples were neutralized with HCl and analyzed by scintillation counting. Data are means ± SD (*n*=3).

To identify the RNA substrates of the QTRT enzyme, a capture-release strategy was developed that exploits a covalent ester linkage between the RNA ribose backbone and Asp279 of the QTRT catalytic subunit (Figures 1A and 7A). This intermediate is formed in the presence of 9-deazaguanine but does not subsequently collapse (5). Briefly, the approach involves incubating RNA with the QTRT enzyme (His-QTRT1:StrepII-QTRT2) in the presence of 9-deazaguanine, followed by a purification of the captured QTRT–RNA–protein complex using Streptactin beads, which recognize the StrepII tag-sequence attached to the QTRT2 noncatalytic subunit. The complex was subsequently washed in buffer, the proteins denatured in urea to separate the His-QTRT1–RNA pair from the StrepII-QTRT2 subunit. The denatured His-QTRT–RNA complex was washed in denaturing buffer and captured on magnetic beads. In initial validation studies the RNA was cleaved and then eluted from His-QTRT with aqueous NaOH for examination by either scintillation counting or by gel separation. Alternatively, for NGS analysis the His-QTRT–RNA complex was de-phosphorylated and re-phosphorylated *in situ* before the RNA was cleaved and eluted with aqueous NaOH and adapter ligated for subsequent NGS analysis.

Proof of principle for the capture-release method was obtained using the Asp anticodon stem loop (Figure 7B), which was shown to form a stable covalent intermediate on SDS-PAGE only in the presence of both enzyme subunits and 9-deazaguanine (rightmost lane; Q1-ASL). To validate our earlier *in vitro* findings showing small RNA are the principal QTRT substrates, we examined the ability of the enzyme to capture ^32^P-labeled RNA isolated from MDA-MB-231 cells. The RNA captured by the QTRT enzyme all migrated at a molecular weight consistent with tRNA, approximately 70 nucleotides in length (Figure 7C) and could be competitively displaced with the addition of excess tyrosyl tRNA (Figure 7D), a known QTRT substrate. With a view towards later experiments aimed at detection/binding of even minor RNA species—the assay was performed utilizing an increasing gradient of RNA and a fixed 4 μg amount of QTRT enzyme (Figure 7E).

### GUN tRNA are the principle small RNA substrates of the QTRT enzyme

As determined earlier, RNA that is modified with queuine base is not a compatible substrate for the QTRT enzyme (Figure 5). Therefore, in order to identify the full complement of QTRT RNA substrates, RNA that was Q-deficient was recovered from a number of sources, i.e. MDA-MB-231 breast cancer cells cultured in queuine-free medium (28), fetal mouse liver, previously shown to be Q-hypomodified (29) and RNA from the splenocytes of experimental autoimmune encephalomyelitis (EAE)-diseased animals, which our previous work demonstrated can readily accept queuine and is thus also lacking in Q-modification (10).

**Figure 8.**
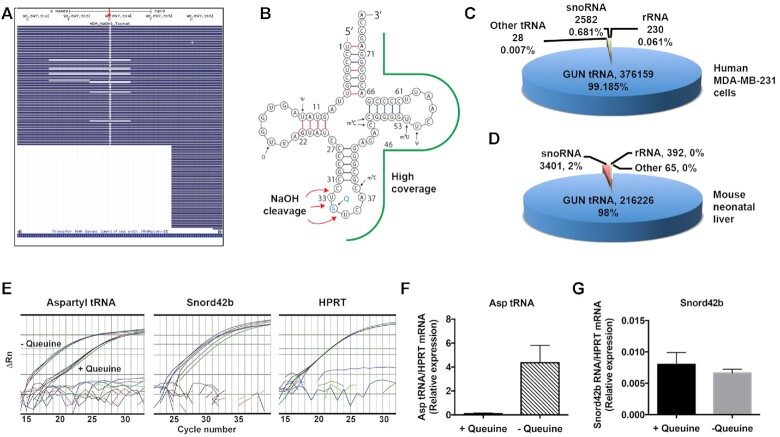
GUN tRNA are the principle small RNA substrates of the QTRT enzyme. RNA from human MDA-MB-231 cells and neo-natal mouse liver was processed by the capture and release method, adapter ligated and subjected to NGS sequencing on a MiSeq platform. Human and mouse fastq files were aligned using Tophat2 to the hg19 and mm10 genome assemblies, respectively. (**A**) Representative BAM file for an MDA-MB-231 alignment, visualized on the UCSC genome browser, showing the canonical ‘TGTN’ site—UGUC in aspartyl tRNA—showing the G to T transversion (red arrow) created by the abasic site during library generation. (**B**) Aspartyl tRNA sequence showing the regions of high coverage (green outline) and the sites of NaOH cleavage (red arrows). (**C** and**D**) Representative pie-charts showing the read number and percentage of RNA species captured from MDA-MB-231 cells and neo-natal mouse liver from a single run. For example, assay to determine if snoRNA can serve as *in vivo* substrate for the QTRT enzyme. Splenocytes recovered from 8-week-old, EAE-disease induced C57BL/6J female mice upon reaching a clinical score of 2. Single cell suspensions (2 × 10^6^ cells/ml) in X-vivo 15 medium were re-stimulated with MOG_[33–55]_ (50 μg/ml) for 48 h. Cells were left untreated or administered a large excess of queuine (200 μM) for 24 h before total RNA was isolated. (**E**) Following the capture-release method, Aspartyl tRNA and snord42b RNA quantified by stem-loop adapter-ligated RTPCR and adapter-ligated Taqman RTPCR, respectively. HPRT mRNA quantified by Taqman RTPCR directly from total RNA. (**F** and**G**) Ratio of aspartyl tRNA and Snord42b RNA relative to HRPT mRNA transcript levels.

The capture-release strategy was employed to isolate RNA substrates from the total RNA pool, with three independent runs performed for the MDA-MB-231 cells and fetal liver, and a single run in the case of splenocytes. The mapped and processed data are provided for each (Supplementary Tables S1–S7; Tab ‘regions covered’). In preliminary experiments, it became apparent that the *MMuL* reverse transcriptase (RT) used in the NGS library preparation kit was unable to adequately elongate the cDNA, most likely due to the presence of an abasic site after NaOH-mediated cleavage (data not shown). Therefore, a thermostable version of HIV RT was employed that could transcribe through secondary and tertiary RNA structures and possessed the ability to read across abasic sites (30). In the majority of cases, the use of the HIV RT led to incorporation of thymidine at this site in place of guanine (Figure 8A), although a significant proportion of the reads terminated in the vicinity of the abasic site, leading to a greater coverage of 3′-reads, as shown for a representative aspartyl tRNA (Figure 8B) Following quality control of the NGS data, the sequences were adapter-trimmed, reads were mapped to the respective mouse and human genomes, and those reads overlapping with annotated features selected. QTRT-bound RNA reads were identified as being >16 nucleotides in length, possessing a nucleotide transversion with a frequency ≥30% and having a total coverage of ≥10 reads (Supplementary Tables S1–S7; Tab ‘regions covered, compiled’).

Following compilation of the data, a number of anomalously annotated sequences existed. These were manually inspected and divided across three separate datasets; tRNA, snoRNA/ribosomal RNA (rRNA) and other RNA reads. Cytosolic and mitochondrial GUN tRNA accounted for all but a small proportion of the mapped reads (MDA-MB-231 cells >99%, neonatal liver >96%, splenocytes >92%), with cytosolic aspartyl tRNA and mitochondrial histidyl tRNA proving to be the preferred QTRT substrate based on the number of reads recovered. Notably, it was observed that the mitochondrial tRNA reads mapped both to the mitochondrial and multiple unannotated regions of the nuclear genome, corroborating earlier observations of ‘mitochondrial tRNA lookalikes’ being present in the genomes of primate and rodent species (31).

From the capture-release assays, several non-tRNA species were identified that corresponded to a range of small nucleolar RNA species of the C/D box snoRNA (SNORD) and H/ACA box snoRNA (SNORA) families (MDA-MB-231 cells, 0.2–0.7%; fetal liver, 0–3%; splenocytes 4.7%) and to pre-ribosomal 45S RNA (MDA-MB-231 cells, 0–0.06%; fetal liver, 0–1%; splenocytes, 2.6%), for which representative data are shown (Figure 8C,D). Within the nucleolus, pre-ribosomal 45S RNA are post-transcriptionally processed into the 18S, 5.8S and 28S rRNAs by a series of cleavage events (32). Splenocyte reads aligning to mouse 45S pre-ribosomal RNA genes in chromosomes 5, 6, 9 and 17 show that the reads principally fall within the 5′ external transcribed spacer (5′ETS), the 18S rRNA, and the internal transcribed spacer 1 (ITS1) with few reads observed to map to the 28S rRNA (Supplementary Figure S1).

SNORD and SNORA noncoding RNA are also found within the nucleolus and function in small nucleolar ribonucleoprotein (snoRNP) complexes to direct the site-specific 2′-O-methylation and pseudouridylation of rRNAs at target uridines, respectively (33,34). Previously, it has been shown that the eukaryotic QTRT enzyme is localized within the cytosol, in association with the mitochondria (3) and therefore, it is considered improbable that RNA within the nucleolar compartment could function as a true target substrate for queuine insertion. To investigate this in a cellular context, the queuine modification status of both SNORD42b (the most abundantly identified snoRNA) and aspartyl tRNA, was examined in primary splenocytes cultured in the presence and absence of queuine base (Figure 8E) using the capture-release method followed by four leaf clover quantitative RT-PCR (24). As expected, adding queuine to the splenocyte cultures resulted in a loss of captured aspartyl tRNA due to the fact that queuine-modified tRNA cannot be bound by the enzyme (Figure 8E, left-hand panel and Figure 8F). By contrast, the addition of queuine did not result in a decrease in bound SNORD42b RNA:, suggesting it is not a true QTRT substrate *in vivo* (Figure 8E, middle panel and Figure 8G). Therefore, the data from the capture-release studies indicate the QTRT enzyme has a strict preference for GUN tRNA of both nuclear and mitochondrial origin.

## DISCUSSION

The post-translational modification of RNA, including tRNA, is known to affect a diverse array of eukaryotic biological processes, including proliferation, development, differentiation and metabolism (35–37) There is an increasing interest in the potential of modulating these posttranslational modifications on RNA for therapeutic purposes (38,39). The queuine nucleobase is unique among such RNA modifications as it is exclusively acquired from external sources as a micronutrient—the gut microbiome and ingested food (23)—and thus an attractive candidate for molecular replacement with exogenously supplied mimetics.

Our tRNA incorporation data shows that the QTRT enzyme accepts molecules encompassing a wide area of chemical space around the natural nucleobase substrate, queuine. Both preQ_1_ (**5**) and preQ_0_ (**6**), the natural substrates for the eubacterial and archaeal enzymes respectively, are accepted surprisingly well by the human QTRT enzyme in the tRNA incorporation assay; although it should be highlighted that there is no evidence to date that preQ_1_ and preQ_0_ are either present in the eukaryotic cell or incorporated into either RNA or DNA in a physiological setting. The 7-deazaguanine analogues synthesized here include analogues equipped with remarkably large alkyl substituents: derivatives incorporating electron-withdrawing substituents at C-7, which are structurally different to preQ_0_ (e.g. 7-chloro-7-deazaguanine (**12**) and 7-nitro-7-deazaguanine (**11**)), and a range of ester, carboxy/amide derivatives (**13**–**20**) and oximes (**20**–**24**). Ficner and colleagues (40) have reported the crystal structure of QTRT1 where electron density corresponding to bound queuine was detected in the active site. In this structure the cyclopentene moiety occupies a groove that is expanded by rotation of Ser231 away from the nucleobase. We would propose that this same conformational change creates space for the accommodation of often large side chains associated with the nucleobases evaluated in this study.

In general, it is only when the alkyl substituents become large (e.g. **38**) or when considerable steric bulk is introduced at the benzylic position of derivatives of **33** (e.g. **57–59**) does the substrate competency begin to dramatically deteriorate. It is noteworthy that every substrate prepared for this study served as a substrate for the enzyme, to at least some degree. There is one anomaly, i.e., compound **32**—that potentially acts as an inhibitor. A potential explanation for the promiscuous nucleobase recognition may be an absence of evolutionary pressure to optimise binding of exocyclic substituents at the 7-position. Indeed, queuine and related molecules (i.e. preQ_0_, preQ_1_ and antibiotic structures such as toyocamycin) are the only known examples of 7-deazaguanine compounds commonly found in nature (6). Conversely, it is noteworthy that 7-deazaguanine compounds are not substrates for cellular DNA or RNA incorporation, since the carbon at position 7 (in place of nitrogen) is a critical recognition element for the HPRT salvage enzyme for purine nucleoside formation (41).

Kinetic analysis of the human QTRT enzyme has shown that it exhibits similar *K*_M_ and *k*_cat_ values for both guanine and queuine as substrate (4,42), raising the question of how the enzyme exercises selectivity for the correct substrate. This anomaly has previously been explained by the irreversible nature of queuine-incorporation, which differs from guanine, which can be reversibly displaced after tRNA insertion (20). Expanding upon this work, the data from this study show that guanine (**3**) is incapable of displacing queuine (**4**), preQ_1_ (**5**) or other 7-deazaguaine derivatives (**21**, **23**, **33**) from the tRNA anticodon loop, but may readily displace preQ_0_ (**6**)—showing the importance of the amino-methyl side chain in prohibiting base removal post-incorporation. In contrast, queuine proved capable of partially displacing either itself or other 7-deazaguenine derivatives in an *in vitro* context, demonstrating that enzyme-catalyzed incorporation is reversible under certain conditions.

The capture-release method provides unambiguous evidence for the specificity of the eukaryotic QTRT enzyme toward tRNA of cytosolic and mitochondrial origin (i.e. tyrosyl, aspartyl, histidyl and asparaginyl tRNA). It was somewhat surprising to find that the enzyme could modify an aspartyl tRNA stem loop (9-nucleotides in length) *in vitro*. This intriguing finding suggested that additional substrates, apart from tRNA, may be recognized by the enzyme, given the abundance of non-coding RNA transcribed from the human genome (43,19). Previously, Brooks *et al.*, examined the incorporation of radiolabeled preQ_1_ into *E. coli* RNA and observed bands by gel electrophoresis that they speculated may be 16S rRNA or tRNA and 5S rRNA, although the exact identity of these bands was not determined (17). The capture-release studies returned a minority of reads ascribable to pre-ribosomal RNA and both SNORD and SNORA species, that direct the site-specific 2′-O-methylation and pseudouridylation of ribosomal RNA, respectively. Conceivably, the ribosomal RNA may be recognized by the QTRT enzyme due to the presence of primitive tRNA-like structures, which are said to be the evolutionary relics of early ribosome biogenesis (44). Whether the ribosomal RNA could be recognized by the enzyme post-assembly, specially within the context of the cytosol where the QTRT enzyme is found, is an outstanding question. With respect to the snoRNA and SNORA, it would appear unlikely—though not to be completely ruled out—that they can serve as substrates (or inhibitors) of the QTRT enzyme. First, their compartmentalization to the nucleolus of the cell nucleus ensures a physical separation from the cytoplasmic QTRT protein and secondly the results of our capture-release RT-PCR analysis could find no evidence of queuine modification in extracts from MDA-MB-231 cells.

In conclusion, the data reveal a tantalizing opportunity to direct the site-specific alteration of tRNA using queuine-mimetic substrates and thereby malleably alter the properties of the wobble position of the anticodon loop, a position known to serve a critical role in codon recognition and translation regulation (2,8,9,45).

## Supplementary Material

gkab289_Supplemental_Files
