# Supplementary material for: The human tRNA-guanine transglycosylase displays promiscuous nucleobase preference but strict tRNA specificity
Source: Nucleic Acids Res. 2021 May 1;49(9):4877–90. doi: 10.1093/nar/gkab289 (PMC8136771; doi:10.1093/nar/gkab289)
Supplement: gkab289_Supplemental_Files [file gkab289_supplemental_files.zip › 2a. Supplementary Data S1.pdf]

Sequence of His-tag- hQTRT1 insert cloned into the NdeI and XhoI sites of pET-15b
Insert length:1,290 bp

Restriction sites shown underlined
NdeI restriction enzyme in blue
XhoI restriction enzyme in gold
His- tag in orange
Nucleotide sequence of codon optimized hQTRT in brown
ATG: start codon
TGA: stop codon

AGGAGATATACCATGGGCAGCAGCCATCATCATCATCATCACAGCAGCGGCCTGGTGCCGC
GCGGCAGCCATATGGCTGGTGCCGCAACCCAAGCGTCACTGGAAAGTGCTCCGCGTATTAT
GCGTCTGGTCGCCGAATGCTCCCGTAGTCGTGCTCGTGCAGGCGAACTGTGGCTGCCGCA
TGGTACCGTGGCTACGCCGGTTTTTATGCCGGTCGGCACCCAGGCGACGATGAAAGGTATT
ACCACGGAACAACTGGATGCTCTGGGCTGCCGTATCTGTCTGGGTAACACCTATCACCTGG
GTCTGCGTCCGGGTCCGGAACTGATTCAGAAAGCAAATGGCCTGCATGGTTTTATGAACTG
GCCGCACAATCTGCTGACCGATAGCGGCGGTTTCCAAATGGTTTCTCTGGTCAGCCTGTCC
GAAGTGACCGAAGAAGGCGTTCGTTTTCGCTCACCGTATGATGGTAACGAAACGCTGCTG
AGTCCGGAAAAATCCGTGCAGATCCAAAATGCCCTGGGCTCGGACATTATCATGCAGCTGG
ATGACGTGGTTAGCTCTACCGTCACGGGTCCGCGTGTGGAAGAAGCGATGTACCGTTCCAT
TCGCTGGCTGGATCGTTGTATCGCGGCCCATCAGCGCCCGGACAAACAAAACCTGTTCGC
CATTATCCAGGGCGGTCTGGATGCAGACCTGCGTGCAACCTGCCTGGAAGAAATGACGAA
ACGCGATGTCCCGGGCTTTGCCATTGGCGGTCTGAGCGGCGGTGAATCAAAATCGCAGTT
CTGGCGCATGGTTGCACTGAGCACCTCTCGTCTGCCGAAAGATAAACCGCGCTATCTGATG
GGCGTGGGTTACGCAACGGACCTGGTCGTGTGCGTTGCTCTGGGCTGTGATATGTTTGACT
GCGTCTTCCCGACCCGTACGGCACGTTTTGGCAGCGCCCTGGTGCCGACCGGTAATCTGC
AGCTGCGTAAAAAAGTTTTCGAAAAAGATTTCGGTCCGATCGACCCGGAATGCACCTGTC
CGACGTGTCAAAAACATAGTCGCGCATTCCTGCATGCTCTGCTGCACTCCGATAACACCGC
AGCTCTGCATCACCTGACGGTGCACAATATTGCGTATCAGCTGCAACTGATGTCAGCCGTT
CGTACCTCGATCGTCGAAAAACGTTTTCCGGATTTCGTTCGCGACTTTATGGGCGCGATGT
ACGGTGATCCGACGCTGTGTCCGACCTGGGCTACCGACGCACTGGCATCCGTTGGCATTAC
GCTGGGTTGACTCGAG

Sequence of StrepII-SUMO- hQTRT2 insert cloned into the NcoI and XhoI sites of pCDF-1b
Insert length:1,634 bp

Restriction sites shown underlined
NcoI restriction enzyme in blue
XhoI restriction enzyme in gold
StrepII- tag in **bold gray**
SUMO fusion cDNA sequence in green
Nucleotide sequence of codon optimized hQTRT2 in orange
ATG: start codon
TGA: stop codon

ATACCATGGCAAGC**TGGAGCCACCCGCAGTTCGAAAA**GGGTGCACTTGAAGTCCTCTTT
CAGGGACCCGCGGCCGCAGGATCCATGTCGGACTCAGAAGTCAATCAAGAAGCTAAGCC
AGAGGTCAAGCCAGAAGTCAAGCCTGAGACTCACATCAATTTAAAGGTGTCCGATGGATC
TTCAGAGATCTTCTTCAAGATCAAAAAGACCACTCCTTTAAGAAGGCTGATGGAAGCGTT
CGCTAAAAGACAGGGTAAGGAAATGGACTCCTTAAGATTCTTGTACGACGGTATTAGAATT
CAAGCTGATCAGACCCCTGAAGATTTGGACATGGAGGATAACGATATTATTGAGGCTCACA
GAGAACAGATTGGTGGTCATATGAAGCTGAGTCTTACCAAGGTAGTTAATGGCTGTCGCCT
AGGAAAAATAAAAAACCTGGGCAAAACAGGGGACCACACCATGGATATTCCAGGCTGCCT
TCTGTATACCAAGACTGGCTCCGCCCCACACCTCACCCATCACACGCTGCATAATATCCAC
GGGGTTCCTGCCATGGCTCAGCTTACGCTGTCATCCCTAGCAGAACATCATGAAGTCTTGA
CAGAATATAAAGAAGGAGTTGGAAAGTTTATAGGCATGCCAGAATCACTCTTGTACTGCTC
CCTGCACGATCCAGTCAGCCCCTGCCCGGCTGGTTATGTAACAAACAAGTCTGTGTCTGTG
TGGAGTGTTGCAGGACGAGTGGAAATGACTGTTTCCAAGTTCATGGCAATTCAGAAGGCC
CTTCAGCCAGACTGGTTCCAGTGCCTCTCCGATGGAGAAGTATCTTGTAAGGAAGCAACTT
CCATAAAAAGGGTCAGAAAGTCTGTTGACCGATCACTTCTTTTCTTGGATAACTGTCTGCG
GCTGCAGGAAGAGTCAGAGGTTCTTCAGAAGAGTGTGATCATTGGAGTGATTGAAGGTGG
AGATGTGATGGAAGAGAGGCTGAGGTCAGCACGAGAGACAGCCAAGCGGCCTGTGGGTG
GCTTCCTTCTGGATGGTTTTCAAGGAAATCCAACAACCCTGGAGGCTAGACTACGCTTGCT
GTCATCAGTCACTGCAGAGCTGCCGGAGGACAAGCCAAGGCTCATATCTGGTGTTAGTCG
GCCAGATGAGGTGCTCGAGTGTATTGAAAGAGGAGTGGACTTATTTGAGAGTTTTTTCCCT
TATCAAGTAACAGAGCGGGGATGTGCCCTGACTTTCAGTTTTGATTACCAGCCGAATCCTG
AAGAGACACTACTACAACAAAATGGAACACAAGAAGAAATAAAATGTATGGATCAAATAA
AGAAAATTGAAACAACTGGTTGCAACCAAGAAATAACATCATTTGAAATTAATCTGAAGG
AAAAAAAGTACCAGGAGGACTTTAACCCGCTGGTGAGAGGATGTTCCTGTTACTGCTGTA
AGAATCACACTCGGGCATACATCCACCATCTGCTGGTGACCAATGAGCTGCTGGCCGGAGT
CCTGCTTATGATGCACAACTTTGAACACTACTTTGGGTTTTTCCATTACATCCGGGAAGCAC
TAAAAAGTGACAAACTGGCACAGTTGAAAGAGCTCATCCACAGGCAAGCATCT**TGA**CTC
GAG

Sequence of hQTRT2-StrepII-tag insert cloned into the NcoI and XhoI sites of pCDF-1b
Insert length: 1,319 bp

NcoI restriction enzyme in blue
XhoI restriction enzyme in gold
Nucleotide sequence of codon optimized hQTRT2 in black
TEV site is in orange
StrepII- tag in **bold** gray
ATG: start codon
TGA: stop codon

CGCCCATGGACCTGTCGCTGACGAAAGTTGTTAATGGCTGCCGCCTGGGTAAAATCAAAA
ACCTGGGTAAAACGGGTGACCACACGATGGACATTCCGGGCTGCCTGCTGTATACCAAAA
CGGGTAGCGCGCCGCATCTGACCCATCACACGCTGCATAACATCCACGGTGTTCCGGCAAT
GGCACAGCTGACCCTGAGCTCTCTGGCCGAACATCACGAAGTCCTGACGGAATACAAAGA
AGGCGTGGGTAAATTTATTGGTATGCCGGAATCACTGCTGTATTGCTCGCTGCATGATCCGG
TTTCTCCGTGTCCGGCAGGCTACGTCACCAATAAATCAGTGTCGGTTTGGAGCGTCGCTGG
TCGTGTGGAAATGACGGTTTCGAAATTTATGGCAATTCAGAAAGCTCTGCAACCGGATTGG
TTCCAGTGCCTGAGCGACGGCGAAGTGTCTTGTAAAGAAGCAACCAGTATCAAACGTGTC
CGCAAAAGTGTGGATCGTTCCCTGCTGTTCCTGGACAACTGTCTGCGCCTGCAGGAAGAA
AGTGAAGTTCTGCAAAAATCCGTTATTATCGGTGTCATCGAAGGCGGTGATGTCATGGAAG
AACGTCTGCGTAGCGCGCGTGAAACCGCAAAACGTCCGGTTGGCGGTTTTCTGCTGGACG
GCTTCCAGGGTAATCCGACCACGCTGGAAGCACGTCTGCGCCTGCTGAGTTCCGTGACCG
CTGAACTGCCGGAAGATAAACCGCGTCTGATTAGCGGCGTGTCTCGCCCGGATGAAGTTCT
GGAATGCATCGAACGTGGTGTCGACCTGTTTGAAAGCTTTTTCCCGTATCAGGTGACCGAA
CGCGGCTGTGCCCTGACGTTTTCTTTCGATTACCAACCGAACCCGGAAGAAACCCTGCTG
CAGCAAAATGGCACGCAGGAAGAAATCAAATGCATGGACCAAATTAAGAAAATTGAAACC
ACGGGTTGTAACCAGGAAATCACCAGTTTCGAAATCAACCTGAAAGAGAAAAAATATCAA
GAAGATTTCAACCCGCTGGTGCGTGGTTGCTCCTGTTATTGCTGTAAAAATCATACCCGCG
CGTACATTCATCACCTGCTGGTGACGAACGAACTGCTGGCCGGCGTTCTGCTGATGATGCA
TAATTTTGAACACTATTTCGGCTTTTTCCACTACATCCGCGAAGCACTGAAAAGCGACAAA
CTGGCACAACTGAAAGAACTGATCCATCGTCAAGCGTCCGCGGAGAACCTGTACTTCCAA
TCCAATAGCGCG**TGGAGCCACCCGCAGTTCGAAAAA**TGACTCGAGGA
