# Supplementary material for: The human tRNA-guanine transglycosylase displays promiscuous nucleobase preference but strict tRNA specificity
Source: Nucleic Acids Res. 2021 May 1;49(9):4877–90. doi: 10.1093/nar/gkab289 (PMC8136771; doi:10.1093/nar/gkab289)
Supplement: gkab289_Supplemental_Files [file gkab289_supplemental_files.zip › 2b. Supplementary Data S2.pdf]

Instrumentation

NMR spectra were recorded on Bruker DPX-400 Avance spectrometers, operating at 400.13 and 600.1 MHz for 1H NMR; 100.6 and 150.9 MHz for 13C NMR. Shifts are referenced to the internal solvent signals. HRMS spectra were measured on a MicromassLCT electrospray TOF instrument with a WATERS 2690 autosampler and methanol/acetonitrile as carrier solvent. Melting points were determined using a Stuart SP10 melting point apparatus and are uncorrected. Infrared spectra were recorded on a PerkinElmer Spectrum One FT-IR spectrometer equipped with a Universal ATR sampling accessory. Analytical TLC was performed using either Merck Kieselgel 60 F254 silica gel plates. Visualization was by UV light (254 nm) and staining with potassium permanganate.

6-halosubstituted derivatives of preQ$_0$ 9 and 10 were prepared by the following route:

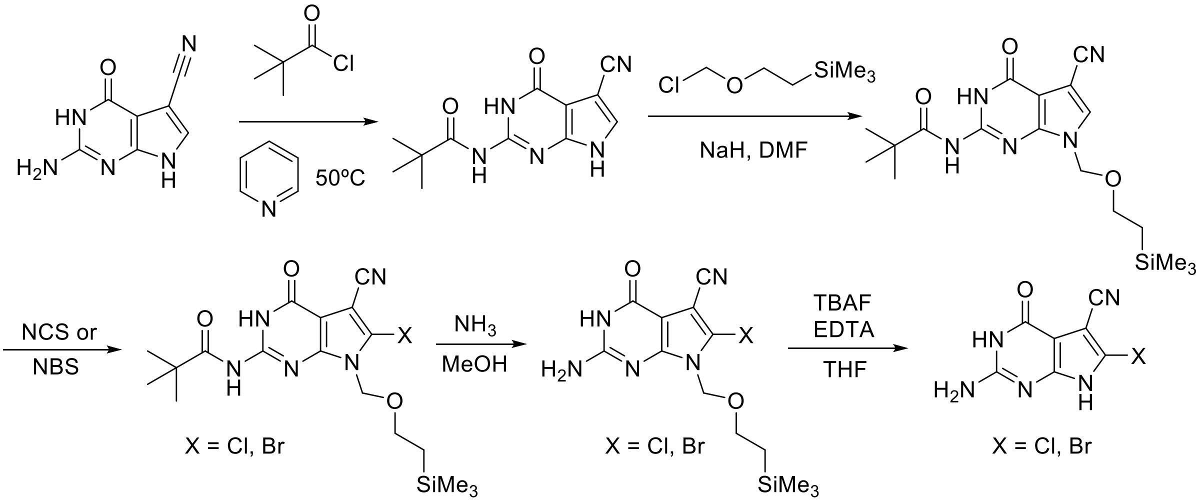

***N*-(5-cyano-4-oxo-4,7-dihydro-3*H*-pyrrolo[2,3-d]pyrimidin-2-yl)pivalamide**

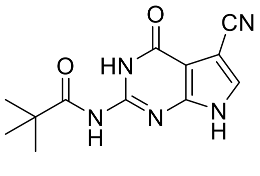

PreQ$_0$ (1.42 g, 6.52 mmol) and triethylamine (2.3 mL, 16.3 mmol) were suspended in pyridine (11.4 mL). The mixture was cooled to 0 ºC and pivaloyl chloride (2.4 mL, 19.6 mmol) was added drop-wise. The reaction was then warmed to 90 ºC and stirred for 18 h. The reaction was cooled to room temperature and the obtained solid was filtered off. The filtrate was evaporated until dryness and

the resulting solid was suspended in aqueous ammonia. After stirring for 1 h the suspension was cooled to 0 ºC and then filtered. The precipitate was dried in desiccator affording the desired product as a brown solid (1.32 g, 78%), m.p. > 300 ºC (decomp).

δ_H ^{1}H NMR (400 MHz, DMSO-d$_6$):	1.21 (s, 9 H), 7.90 (d, *J* 2.4, 1 H), 10.95 (s, 1 H, NH), 12.07 (br s, 1 H, NH), 12.53 (br s 1 H, NH).
HMRS (*m/z* - ESI)	Found: 282.0962 [M + Na]$^+$ $C_{12}H_{13}N_5NaO_2$; Requires: 282.0961

***N*-(5-cyano-4-oxo-7-((2-(trimethylsilyl)ethoxy)methyl)-4,7-dihydro-3*H*-pyrrolo[2,3-d]pyrimidin-2-yl)pivalamide**

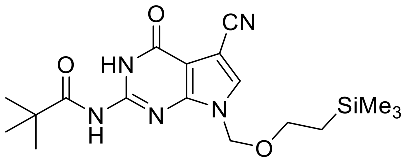

A suspension of sodium hydride (60% in mineral oil, 187 mg, 4.66 mmol) in DMF (25.0 mL) was prepared and cooled to 0ºC before *N*-(5-cyano-4-oxo-4,7-dihydro-3*H*-pyrrolo[2,3-d]pyrimidin-2-yl)pivalamide was added in 3 portions at 0 ºC. The ice-bath was removed and the mixture stirred for 10 min before to be re-cooled to 0 ºC. Neat 2-(Trimethylsilyl)ethoxymethyl chloride (788 µL, 4.45 mmol) was added drop-wise and the mixture stirred for 1 h at 0ºC. The ice-bath was removed and the reaction left stirring for further 40 min. After the addition of EtOAc (150 mL) and water (80 mL), the phases were separated. The organic phase was washed with water (50 mL × 3) and brine (50 mL), dried over $MgSO_4$, filtered and concentrated *in vacuo* to afford a crude residue which was purified by column chromatography (DCM/MeOH 98:2). The resultant solid was triturated with Et_2O affording the desired product as an off-white solid (770 mg, 47%), m.p. 203-206 °C.

δ_H ^{1}H NMR (600 MHz, DMSO-d$_6$):	-0.07 (s, 9 H), 0.84 (t, *J* 8.3, 2 H), 1.24 (s, 9 H), 3.52 (t, *J* 8.3, 2H), 5.46 (s, 2 H), 8.15 (s, 1 H), 11.18 (s, 1 H, NH), 12.24 (s, 1 H, NH).
δ_C ^{13}C NMR (150 MHz, CDCl$_3$):	-1.45, 17.1, 26.2, 40.1, 65.9, 73.4, 86.4, 103.2, 114.6, 132.9, 148.3, 149.0, 155.5, 181.4.
ν_{max} (film)/cm^{-1}:	745, 793, 836, 913, 1083, 1151, 1242, 1421, 1478, 1541, 1608, 1665, 2233, 2972, 3123, 3284.
HRMS (*m/z* –ESI):	Found: 390.1957 [M + H]$^+$ $C_{18}H_{28}N_5O_3Si$ requires: 390.1961

_N_-(6-chloro-5-cyano-4-oxo-7-((2-(trimethylsilyl)ethoxy)methyl)-4,7-dihydro-3_H_-pyrrolo[2,3-d]pyrimidin-2-yl)pivalamide

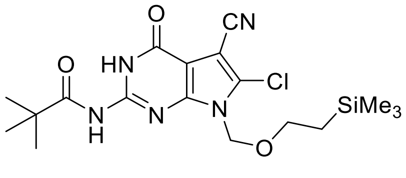

A solution of *N*-(5-cyano-4-oxo-7-((2-(trimethylsilyl)ethoxy)methyl)-4,7-dihydro-3*H*-pyrrolo[2,3-d]pyrimi din -2-yl)pivalamide (239 mg, 0.614 mmol) in DMF (6.0 mL) was cooled to -15 °C (ice/acetone bath). A solution of NCS (98.3 mg, 0.736 mmol) in DMF (3.0 mL) was added dropwise. The reaction vessel was wrapped in an aluminium foil and the mixture was left stirring for 18 h while the ice/bath warmed to room temperature. EtOAc (100 mL) followed by a saturated aqueous solution of $Na_2S_2O_5$ (20 mL) was added to the mixture. The organic phase was separated, washed with water (50 mL × 3), brine (30 mL) and then dried over $MgSO_4$. Filtration and concentration under reduced pressure afforded a crude solid which purified by column chromatography (DCM/EtOAc 9:1) to yield the desired product as a white solid (225 mg, 86%) m.p. 163-165 °C.

δ_H ^{1}H NMR (600 MHz, DMSO-d_6):	-0.07 (s, 9H), 0.87 (t, *J* 8.3, 2H), 1.25 (s, 9H), 3.57 (t, *J* 8.3, 2H), 5.51 (s, 2H), 11.21 (br s, 1H, NH), 12.33 (br s, 1H, NH)
δ_C ^{13}C NMR (150 MHz, $CDCl_3$):	-1.5, 17.0, 26.2, 40.1, 66.2, 71.3, 86.4, 102.4, 112.7, 128.7, 148.0, 149.5, 154.5, 181.4.
ν_{max} (film)/cm^{-1}:	633, 980, 803, 962, 1067, 1148, 1202, 1247, 1289, 1552, 1597, 1670, 2235, 2966, 3126.
HRMS (*m/z* –ESI):	Found 424.1570 $[M + H]^+$ $C_{18}H_{27}N_5O_3SiCl$ requires: 424.1572

2-Amino-6-chloro-4-oxo-7-((2-(trimethylsilyl)ethoxy)methyl)-4,7-dihydro-3_H_-pyrrolo[2,3-d]pyrimidine-5-carbonitrile

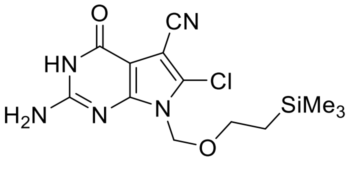

N-(6-chloro-5-cyano-4-oxo-7-((2-(trimethylsilyl)ethoxy)methyl)-4,7-dihydro-3*H*-pyrrolo[2,3-d]pyrimidin-2-yl)pivalamide (275 mg, 0.649 mmol) was dissolved in methanolic NH_3 (7N, 9.0 mL) and stirred at room temperature for 64 h. The precipitate was collected by filtration, washed with CH_2Cl_2 and dried *in vacuo* affording the desired product as a white solid (151 mg, 68%) m.p. > 260 °C (decomp.

δ_H ^1H NMR (400 MHz, DMSO-d_6):	-0.07 (s, 9H), 0.84 (t, *J* 7.9, 2H), 3.55 (t, *J* 7.9, 2H), 5.35 (s, 2 H), 6.71 (br s, 2H, NH_2), 11.01 (br s, 1H, NH)
δ_C ^{13}C NMR (100 MHz, $CDCl_3$):	-1.0, 17.5, 66.5, 71.2, 86.3, 98.2, 113.8, 126.3, 151.5, 155.0, 157.1
ν_{max} (film)/cm^{-1}:	699, 774, 824, 929, 1087, 1246, 1353, 1396, 1516, 1561, 1584, 1638, 1676, 2229, 2951, 3165, 3333, 3507.
HRMS (*m/z* –ESI):	Found 340.0997 $[M + H]^+$ $C_{13}H_{19}N_5O_2SiCl$ requires: 340.0997

2-Amino-6-chloro-4-oxo-4,7-dihydro-3*H*-pyrrolo[2,3-d]pyrimidine-5-carbonitrile (9)

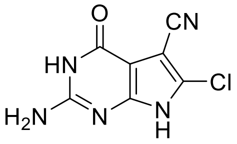

To a solution of 2-Amino-6-chloro-4-oxo-7-((2-(trimethylsilyl)ethoxy)methyl)-4,7-dihydro-3*H*-pyrrolo[2,3-d]pyrimidine-5-carbonitrile (125 mg, 0.367 mmol) and ethylenediamine (74 µL, 1.103 mmol)in THF (6.0 mL) was added TBAF (1.0 M in THF, 2.2 mL, 2.207 mmol). The mixture was then heated to 60 °C until the reaction was deemed complete (by ^1H NMR analysis). The suspension was cooled to room temperature and then acidified to pH 5 by the addition of HCl (0.5 N). The precipitate was collected by filtration, washed with methanol and dried in desiccator affording the desired product as a light brown solid. (68 mg, 88%) m.p. > 300 °C (decomp).

δ_H ^1H NMR (600 MHz, DMSO-d_6):	6.48 (br s, 2H, NH_2), 10.81 (br s, 1H, NH), 12.94 (br s, 1H, NH).
δ_C ^{13}C NMR (150 MHz, $CDCl_3$):	84.5, 98.9, 113.7, 124.2, 150.8, 154.0, 156.6.
ν_{max} (film)/cm^{-1}:	592, 675, 771, 891, 1155, 1241, 1580, 1655, 2239, 3116, 3313, 3426.
HRMS (*m/z* –ESI):	Found 208.0019 $[M – H]^+$ $C_7H_3N_5OCl$ requires: 208.0026

***N*-(6-bromo-5-cyano-4-oxo-7-((2-(trimethylsilyl)ethoxy)methyl)-4,7-dihydro-3*H*-pyrrolo[2,3-d]pyrimidin-2-yl)pivalamide**

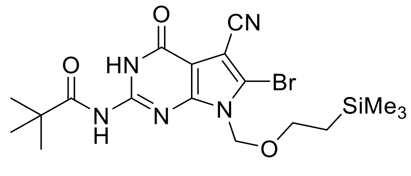

A solution of *N*-(5-cyano-4-oxo-7-((2-(trimethylsilyl)ethoxy)methyl)-4,7-dihydro-3*H*-pyrrolo[2,3-d]pyrimi din -2-yl)pivalamide (147 mg, 0.377 mmol) in DMF (3.5 mL) was cooled to -6 °C (ice/salt bath). A solution of NBS (74 mg, 0.415 mmol) in DMF (1.5 mL) was added dropwise and the mixture was left stirring for 30 min while turning green. The reaction was then allowed to reach room temperature and stirred for further 40 min. EtOAc (100 mL) followed by a saturated aqueous solution of $Na_2S_2O_5$ (20 mL) were added to the mixture. The organic phase was separated, washed with water (50 mL × 3), brine (30 mL) and then dried over $MgSO_4$. Filtration and concentration at reduced pressure afforded a crude solid which was purified by column chromatography (DCM/EtOAc 9:1) to yield the desired product as a white solid (145 mg, 82%) m.p. 170-172 °C.

δ_H ^{1}H NMR (600 MHz, DMSO-d$_6$):	-0.07 (s, 9 H), 0.87 (t, *J* 8.3, 2H), 1.25 (s, 9 H), 3.57 (t, *J* 8.3, 2H), 5.51 (s, 2 H), 11.15 (br s, 1 H, NH), 12.30 (br s, 1 H, NH).
δ_C ^{13}C NMR (150 MHz, $CDCl_3$):	-1.5, 17.0, 26.2, 40.1, 66.1, 72.3, 90.1, 103.5, 113.6, 117.6, 149.0, 149.3, 154.4, 181.4.
ν_{max} (film)/cm^{-1}:	695, 771, 790, 837, 958, 1086, 1106, 1148, 1244, 1286, 1421, 1485, 1548, 1607, 1678, 1714, 2228, 2898, 2961, 3200, 3430.
HRMS (*m/z* –ESI):	Found 468.1063 $[M + H]^+$ $C_{18}H_{27}N_5O_3BrSi$ requires: 468.1067

2-Amino-6-bromo-4-oxo-7-((2-(trimethylsilyl)ethoxy)methyl)-4,7-dihydro-3*H*-pyrrolo[2,3-d]pyrimidine-5-carbonitrile

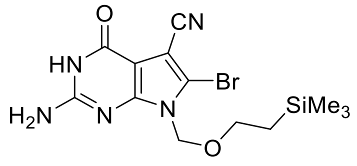

N-(6-bromo-5-cyano-4-oxo-7-((2-(trimethylsilyl)ethoxy)methyl)-4,7-dihydro-3H-pyrrolo[2,3d]-pyrimidin-2-yl)pivalamide (103 mg, 0.246 mmol) was dissolved in methanolic NH_3 (7N, 3.4 mL) and stirred at room temperature for 64 h. The precipitate was collected by filtration, washed with CH_2Cl_2 and dried *in vacuo* affording the desired product as a white solid (43 mg, 45%), m.p. > 238 °C (decomp)

δ_H ^{1}H NMR (600 MHz, DMSO-d$_6$):	-0.07 (s, 9H), 0.84 (t, *J* 7.9, 2H), 3.5 (t, *J* 7.9, 2H), 5.35 (s, 2 H), 6.68 (br s, 2 H, NH_2), 10.96 (br s, 1 H, NH)

δ_C ^{13}C NMR (150 MHz, $CDCl_3$):	-1.43, 17.04, 65.9, 71.8, 89.6, 98.9, 114.2, 114.3, 151.9, 154.3, 156.4
ν_{max} (film)/cm^{-1}:	777, 800, 833, 1095, 1245, 1376, 1560, 1586, 1635, 1677, 2230, 2951, 3169, 3337, 3457.
HRMS (*m/z* –ESI):	Found 384.0488, $[M + H]^+$ $C_{13}H_{19}N_5O_2SiBr$ requires: 384.0491

2-Amino-6-bromo-4-oxo-4,7-dihydro-3*H*-pyrrolo[2,3-d]pyrimidine-5-carbonitrile (10)

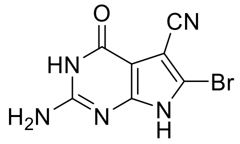

To a solution of 2-Amino-6-bromo-4-oxo-7-((2-(trimethylsilyl)ethoxy)methyl)-4,7-dihydro-3*H*-pyrrolo[2,3-d]pyrimidine-5-carbonitrile (48 mg, 0.125 mmol) and ethylenediamine (25 µL, 0.375 mmol) in THF (2.3 mL) was added TBAF (1.0 M in THF, 750 µL, 0.749 mmol). The mixture was then heated at 60 °C until the reaction was deemed complete (by ^1H NMR analysis). The suspension was cooled to room temperature and then acidified to pH 5 by the addition of HCl (0.5 N). The precipitate was collected by filtration and dried in desiccator affording the desired product as a light brown solid. (20 mg, 62%), m.p. > 300 °C (decomp).

δ_H ^1H NMR (600 MHz, DMSO-d_6):	6.44 (br s, 2H, NH_2), 10.75 (br s, 1H, NH), 12.81 (br s, 1H, NH).
δ_C ^{13}C NMR (150 MHz, $CDCl_3$):	88.3, 99.9, 110.8, 114.5, 152.0, 154.0, 156.4.
ν_{max} (film)/cm^{-1}:	662, 715, 772, 874, 1019, 1154, 1232, 1357, 1418, 1502, 1578, 1655, 1686, 2241, 3112, 3316, 3432.
HRMS (*m/z* –ESI):	Found 251.9526 $[M – H]^+$ $C_7H_3N_5OBr$ requires: 251.9521

2-Amino-5-chloro-3,7-dihydro-4*H*-pyrrolo[2,3-*d*]pyrimidin-4-one (12)

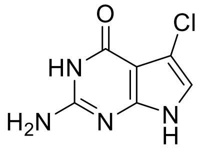

A round-bottomed flask containing was charged with 4,5-Dichloro-7*H*-pyrrolo[2,3-*d*]pyrimidin-2-amine (80.0 mg, 0.39 mmol) and aq. HCl (2.0 M, 8 cm^3), was fitted with a condenser and was heated at 65 °C for 16 hours. After cooling, the reaction mixture was basified to pH 8 using aqueous ammonia solution. The resulting precipitate was isolated using vacuum filtration to yield a brown solid which was purified by column chromatography using EtOAc:MeOH 9:1 to yield the title compound as a white solid (57 mg, 78 %) m.p. > 200 ˚C (dec.)

δ_H (600 MHz, DMSO-d_6):	6.17 (br s, 2H), 6.70 (s, 1H), 10.41 (br s, 1H), 11.16 (br s, 1H)
δ_C (150 MHz, DMSO-d_6):	97.5, 105.5, 114.5, 150.6, 153.2, 158.2
ν_{max} (film)/cm^{-1}:	1063, 1404, 1565, 1549, 1687, 2949, 3667
HRMS (*m/z* –ESI):	Found: 183.0084 $[M – H]^-$ $C_6H_4ClN_4O$; Requires: 183.0080

Ester (13) and acid (14) were prepared in the following manner:

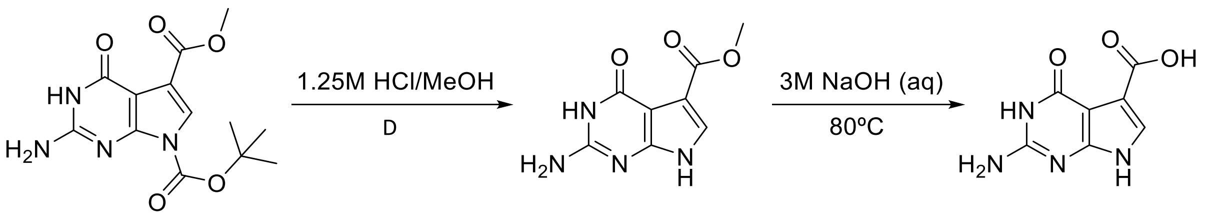

13 **14**

Methyl 2-amino-4-oxo-4,7-dihydro-3*H*-pyrrolo[2,3-*d*]pyrimidine-5-carboxylate (13)

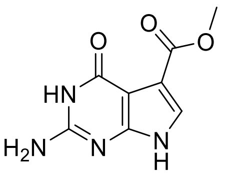

A round-bottomed flask was charged with 7-(*tert*-butyl) 5-methyl 2-amino-4-oxo-3,4-dihydro-7*H*-pyrrolo[2,3-*d*]pyrimidine-5,7-dicarboxylate (70 mg, 0.22 mmol) and a solution of HCl (1.25 M) in MeOH. The resulting suspension was refluxed for 6 hours and then filtered to yield the title compound as a white powder (41.0 mg, 89 %), m.p. > 300 ˚C (dec.) The isolated compound exhibited identical spectroscopic data to those in the literature.

δ_H ^{1}H NMR (400 MHz, DMSO-d_6):	3.64 (s 3H), 6.19 (br s, 2H, NH_2), 7.34 (s, 1H), 10.36 (br s, 1H, NH), 11.62 (br s, 1H, NH)

2-Amino-4-oxo-4,7-dihydro-3*H*-pyrrolo[2,3-*d*]pyrimidine-5-carboxylic acid (14)

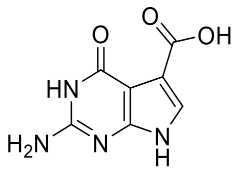

A round bottomed flask was charged with Methyl 2-amino-4-oxo-4,7-dihydro-3*H*-pyrrolo[2,3-*d*]pyrimidine-5-carboxylate (**13**) (41 mg, 0.20 mmol) and aqueous NaOH (5.00 cm^3, 3.0 M). The resulting suspension was heated at 80 ˚C for 3 hours. The reaction mixture was then extracted with 2 x EtOAc (10.0 cm^3) and acidified to pH 4 *via* dropwise addition of aqueous HCl (2.0 M). The resulting precipitate was filtered via vacuum filtration and subsequently dried in a vacuum desiccator over P_2O_5 to yield a brown solid 32 (36 mg, 96%) m.p. > 300 ˚C (dec.)

δ_H ^1H NMR (600 MHz, DMSO-d_6):	6.70 (2H, br s), 7.43 (d, *J* 2.4, 1H), 11.60 (1H, br s), 12.03 (1H, br s), 14.13 (1H, br s,)
δ_c ^{13}C NMR (150 MHz, DMSO-d_6):	96.4, 110.5, 125.9, 152.4, 153.2, 161.5, 163.2
ν_{max} (film)/cm^{-1}:	751, 1335, 1509, 1606, 2922, 3114, 3343
HRMS (*m/z* –ESI):	Found: 217.0341 $[M + H]^+$ $C_7H_6N_4NaO_2$; Requires: 217.0332

Amides (16 to 20) were prepared by the general procedure:

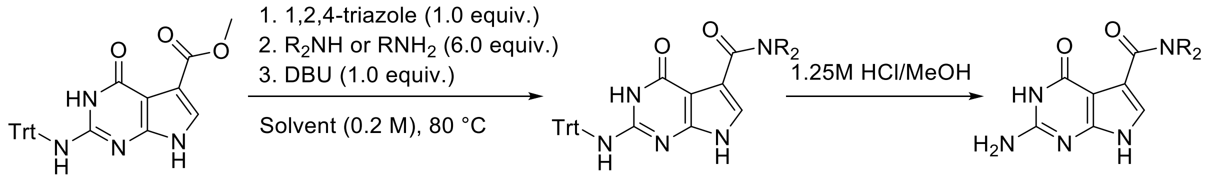

Methyl 4-oxo-2-(tritylamino)-4,7-dihydro-3*H*-pyrrolo[2,3-*d*]pyrimidine-5-carboxylate

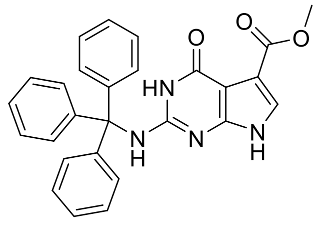

A round-bottomed flask containing a stirring bar was charged with Methyl 2-amino-4-oxo-4,7-dihydro-3*H*-pyrrolo[2,3-*d*]pyrimidine-5-carboxylate (1.50 g, 7.20 mmol) and trityl chloride (3.00 g, 10.8 mmol). The flask was fitted with a condenser and placed under an Ar atmosphere (balloon). Freshly distilled pyridine (20.0 cm^3) was added and the resulting suspension was heated at 100 ˚C for 16 hours. The solution was concentrated *in vacuo* to give a brown oil. Ammonia solution (30.0 cm^3, 35% aqueous) was added and the resulting suspension was stirred at room temperature for 2 hours. The precipitate was collected *via* vacuum filtration, then dissolved in Et_2O (20.0 cm^3) and finally

stirred at room temperature for two hours. The resulting suspension was filtered *via* vacuum filtration to yield the title compound as a beige powder (2.30 g, 72 %) m.p. > 160 ˚C (dec.)

δ_H ^{1}H NMR (600 MHz, DMSO-d_6):	3.63 (s, 3H,), 7.14 (d, *J* 1.9, 1H), 7.21-7.31 (m, 15H), 7.46 (br s, 1H,NH), 10.40 (br s, 1H, NH), 11.45 (br s, 1H,NH)
δ_C ^{13}C NMR (150 MHz, DMSO-d_6):	51.2, 70.5, 98.7, 110.2, 125.2, 127.1, 128.1, 129.1, 145.3, 151.1, 151.6, 157.4, 163.7
ν_{max} (film)/cm^{-1}:	698, 1046, 1441, 1653, 1712, 3401 (br)
HRMS (*m/z* –ESI):	Found: 451.1766 $[M + H]^+$ $C_{27}H_{23}N_4O_3$ Requires: 451.1764

General procedure A: general procedure for aminolysis of Methyl 4-oxo-2-(tritylamino)-4,7-dihydro-3*H*-pyrrolo[2,3-*d*]pyrimidine-5-carboxylate.

To a suspension of Methyl 4-oxo-2-(tritylamino)-4,7-dihydro-3*H*-pyrrolo[2,3-*d*]pyrimidine-5-carboxylate and 1,2,4-triazole (1 eq) in the appropriate anhydrous solvent (0.2 M solution) was added the appropriate amine (6 eq) and DBU (1 eq). The mixture was stirred at 80 ºC till the reaction reached completion as monitored by ^{1}H NMR spectroscopic analysis. The reaction mixture was then concentrated *in vacuo.* The crude residue was then purified by column chromatography.

General Procedure B: general procedure for deprotection of trityl protected amides.

The appropriate trityl-protected compound was dissolved in 1.25 M methanolic HCl (100 mL/mmole of substrate) and the resulting solution was stirred for 16 hours at room temperature. The precipitate was isolated *via* vacuum filtration and washed with cold Et_2O and DCM to yield the desired product.

2-Amino-N,N-dimethyl-4-oxo-4,7-dihydro-3H-pyrrolo[2,3-d]pyrimidine-5-carboxamide (16)

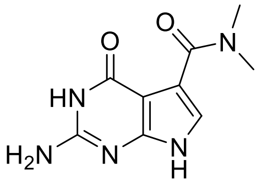

Prepared according to general procedures **A** (using a 2 M solution of dimethylamine in MeOH, solvent - MeOH) and **B** to give the title compound as a white powder (13 mg, 41% over two steps) m.p. > 350 ˚C (dec.)

δ_H ^{1}H NMR (400 MHz, DMSO-d_6):	2.98 (s, 6H), 6.84 (br s, 2H, NH_2), 7.09 (s, 1H), 11.31 (br s, 1H,NH), 11.82 (s, 1H,NH)
δ_C ^{13}C NMR (150 MHz, DMSO-d_6):	37.5, 98.0, 113.1, 121.5, 149.0, 153.0, 158.9, 165.5

ν_{max} (film)/cm^{-1}: 1217, 1371, 1738, 2970, 2851, 3016, 3405

HRMS (*m/z*-ESI): Found: 220.0837 [M - H]$^-$ $C_9H_{10}N_5O_2$ requires: 220.0840

2-Amino-5-(pyrrolidine-1-carbonyl)-3,7-dihydro-4*H*-pyrrolo[2,3-*d*]pyrimidin-4-one (17)

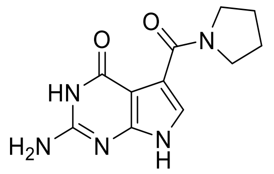

Prepared according to general procedures **A** (solvent – DMF) and **B** to give the title compound as a white powder (17 mg, 45% over two steps) m.p. > 250 ˚C (dec.)

δ_H ^{1}H NMR (600 MHz, DMSO-d$_6$): 1.89 (m, 4H), 3.55 (m, 4H), 6.68 (br s, 2H, NH_2), 7.43 (s, 1H), 11.59 (br s, 1H, NH), 12.09 (br s, 1H, NH)

δ_C ^{13}C NMR (150 MHz, DMSO-d$_6$): 24.2, 26.1, 47.7, 48.6, 98.0, 102.2, 128.0, 152.8, 153.6, 162.7, 163.8

ν_{max} (film)/cm^{-1}: 989, 1577, 1691, 2904, 3549

HRMS (*m/z*-ESI): Found: 270.0959 [M + Na]$^+$ $C_{11}H_{13}N_5NaO_2$ requires: 270.09615

2-Amino-5-(piperidine-1-carbonyl)-3,7-dihydro-4*H*-pyrrolo[2,3-*d*]pyrimidin-4-one (18)

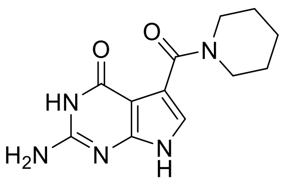

Prepared according to general procedures **A** (solvent – MeOH) and **B** to give the title compound as a white powder (27 mg, 45% over two steps) m.p. > 250 ˚C (dec.)

δ_H ^{1}H NMR (600 MHz, DMSO-d$_6$): 1.38-1.44 (m, 4H), 1.50-1.55 (m, 2H,), 3.36-3.42 (m, 4H,), 6.59 (br s, 2H, NH_2), 6.82 (d, *J* 2.4, 1H), 10.79 (br s, 1H, NH), 11.45 (s, 1H, NH)

δ_C ^{13}C NMR (150 MHz, DMSO-d$_6$): 24.6, 25.9, 48.6, 98.0, 111.7, 114.3, 149.3, 152.7, 158.1, 164.6

ν_{max} (film)/cm^{-1}: 1205, 365, 1429, 1738, 2539, 2947, 2997, 3144, 3405

HRMS (*m/z*-ESI): Found: 260.1156 [M - H]$^-$ $C_{12}H_{14}N_5O_2$ requires: 260.1153

2-Amino-5-(morpholine-4-carbonyl)-3,7-dihydro-4*H*-pyrrolo[2,3-*d*]pyrimidin-4-one (19)

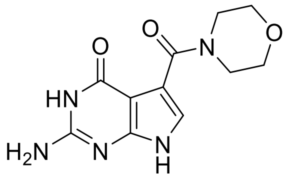

Prepared according to general procedures **A** (solvent – MeOH) and **B** to give the title compound as a white powder (60 mg, 51% over two steps) m.p. > 250 ˚C (dec.)

δ_H ^{1}H NMR (400 MHz, DMSO-d$_6$):	3.22-3.48 (m, 4H), 3.49-3.57 (m, 4H), 6.30 (br s, 2H, NH_2), 6.83 (d, *J* 2.1, 1H), 10.50 (br s, 1H, NH), 11.36 (s, 1H, NH)
δ_C ^{13}C NMR (150 MHz, DMSO-d$_6$):	40.5, 66.5, 98.1, 113.6, 119.0, 149.4, 152.9, 158.3, 165.2
ν_{max} (film)/cm^{-1}:	999, 114, 1284, 1604, 1702, 2716, 3001, 3135, 3362
HRMS (*m/z*-ESI):	Found: 262.0948 [M - H]$^-$ $C_{11}H_{12}N_5O_3$: requires 262.0946

2-Amino-*N*-benzyl-4-oxo-4,7-dihydro-3*H*-pyrrolo[2,3-*d*]pyrimidine-5-carboxamide (20)

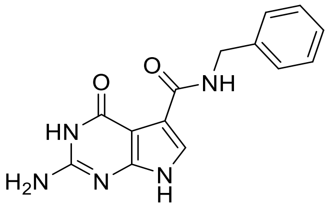

Prepared according to general procedures **A** (solvent – MeOH) and **B** to give the title compound as a white powder (11 mg, 52% over two steps) m.p. > 190 ˚C (dec.)

δ_H ^{1}H NMR (600 MHz, DMSO-d$_6$):	4.45 (d, *J* 6.2, 2H), 6.38 (br s, 2H, NH_2), 7.20-7.31 (m, 6H), 10.55 (t, *J* 6.2, 1H), 10.84 (br s, 1H, NH), 11.62 (s, 1H, NH)
δ_C ^{13}C NMR (150 MHz, DMSO-d$_6$):	42.4, 96.3, 114.9, 123.4, 127.1, 127.5, 128.8, 140.2, 151.9), 153.1, 160.8, 163.0
ν_{max} (film)/cm^{-1}:	1217, 1355, 1738, 2266, 2945, 2970, 3015, 3463
HRMS (*m/z*-ESI):	Found: 282.1006 [M - H]$^-$ $C_{14}H_{12}N_5O_2$ requires: 282.0997

Oximes were prepared by the general procedure:

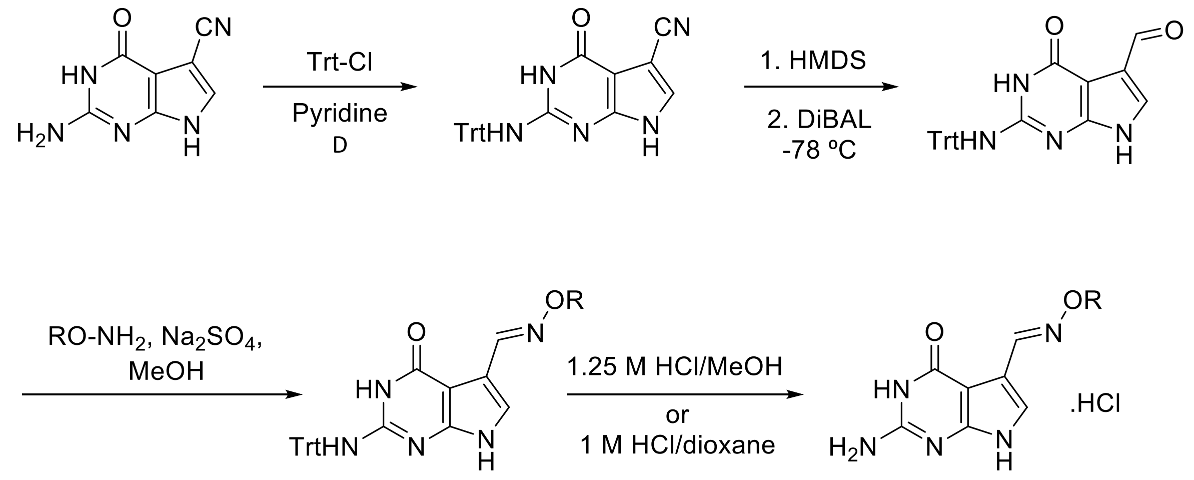

4-Oxo-2-(tritylamino)-4,7-dihydro-3*H*-pyrrolo[2,3-*d*]pyrimidine-5- carbonitrile

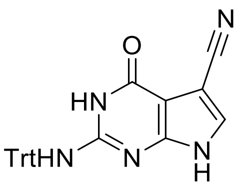

To a mixture of $PreQ_0$ (1.00 g, 5.71 mmol) and trityl chloride (2.40 g, 8.56 mmol) under an argon atmosphere was added freshly distilled pyridine (18 cm^3) and the suspension was heated under reflux for 24 h. The resulting solution was then concentrated *in vacuo*. The crude residue was taken up into 35% aqueous NH_3 (20 cm^3) and stirred for 30 min. Et_2O (20 cm^3) was added and the resulting precipitate removed *via* vacuum filtration, finally the cake was washed with Et_2O. The precipitate was dried at reduced pressure and then triturated in Et_2O followed by HPLC grade hexane to remove residual trityl chloride. The solid was then filtered out and washed with HPLC grade hexane followed by Et_2O to yield the title compound as a yellow powder (1.64 g, 69%) used without further purification, mp: 196-198 °C.

δ_H ^{1}H NMR (400 MHz, DMSO-d_6): 7.13-7.26 (m, 15H), 7.37 (s, 1H), 7.57 (br s, 1H, NH), 10.67 (br s, 1H, NH), 11.74 (br s, 1H, NH)

HRMS (*m/z* -ES): Found: 418.1665 $[M + H]^+$ $C_{26}H_{26}N_5O$ Requires: 418.1662

4-Oxo-2-(tritylamino)-4,7-dihydro-3*H*-pyrrolo[2,3-*d*]pyrimidine-5-carbaldehyde

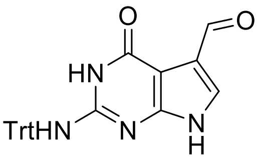

An oven-dried reaction vessel containing a stirring bar was charged with 4-Oxo-2-(tritylamino)-4,7-dihydro-3*H*-pyrrolo[2,3-*d*]pyrimidine-5- carbonitrile (4.00 g, 9.58 mmol), a few mg of $(NH_4)_2SO_4$ and placed under an argon atmosphere. Freshly distilled toluene (8 cm^3) was added *via* syringe followed by dropwise addition of hexamethyldisilazane (4.00 cm^3, 19.16 mmol). The resulting solution was heated under reflux for 2 h, then cooled to room temperature and finally concentrated *in vacuo*. The obtained oil was dissolved in freshly distilled CH_2Cl_2 (25 cm^3) and the reaction mixture cooled to -78 °C before DIBAL-H (20.00 cm^3, 19.16 mmol) was added dropwise. The solution was stirred at -78 °C for 2.5 h. An aliquot of the reaction mixture was taken and analysed by TLC which showed unreacted starting compound remaining, thus a further equivalent of DIBAL-H (10.00 cm^3, 9.58 mmol) was added. On detection of total conversion the reaction was quenched by careful addition of 9:1 H_2O-AcOH (9 cm^3) at -78 °C before the resulting suspension was allowed to warm to room temperature. The suspension was then diluted with 1:1 EtOAc-H_2O (300 cm^3) and stirred until all of the solids had broken up and two layers could be clearly observed on standing. The layers were then separated and the aqueous layer further extracted with EtOAc (2 x 150 cm^3). The combined organic extracts were washed with brine, dried ($MgSO_4$) and concentrated *in vacuo* to yield the crude product. The crude product was taken up into EtOAc and filtered through a pad of silica eluting with EtOAc until no trace of product could be detected in the eluent. The filtrate was then concentrated *in vacuo* to yield the title compound as a yellow powder (2.56 g, 65%), mp: > 250 °C (decomp.).

δ_H ^1H NMR (400 MHz, DMSO-d_6):	7.15-7.29 (m, 16H), 7.54 (br s, 1H, NH), 9.99 (s, 1H), 10.64 (br s, 1H, NH), 11.81 (br s, 1H, NH)
HRMS (*m/z* -ES):	Found: 443.1478 ($[M + Na]^+$ $C_{26}H_{20}N_4NaO_2$; Requires: 443.1478)

General procedure C: Synthesis of oximes

2-Amino-4-oxo-4,7-dihydro-3*H*-pyrrolo[2,3-*d*]pyrimidine-5-carbaldehyde oxime (21)

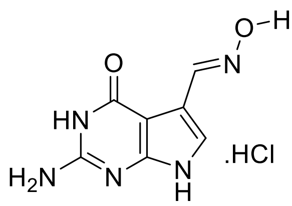

To a suspension of N-((4-oxo-2-(tritylamino)-4,7-dihydro-3H-pyrrolo[2,3-d]pyrimidin-5-yl)methyl)formamide (200 mg, 0.48 mmol) and Na_2SO_4 (5 mg) in MeOH (5 mL) was added the appropriate hydroxylamine, in this case hydroxylamine hydrochloride (0.52 mmol, 36 mg), and triethylamine (104 μL). The resulting suspension was stirred at room temperature until complete consumption of aldehyde starting material was observed by TLC analysis. Water (5 mL) was added and the resulting suspension stirred for 10 mins before being extracted with CH_2Cl_2 (3 x 5 mL). The combined organic layers were dried ($MgSO_4$) and concentrated *in vacuo* to yield the crude product which was purified by flash chromatography (7:3 hexane ethyl acetate) to yield the desired compound as a white solid (100 mg, 48%), mp > 300 °C.

A solution of the trityl-protected compound (100 mg, 0.23 mmol) in 1 M HCl in dioxane (2.2 mL) was prepared and stirred at room temperature for 3 h. The precipitated product was collected by filtration and washed with CH_2Cl_2 to yield the title compound as a white powder, (44 mg, 41 % over two steps), containing an inseparable mixture of E/Z isomers in a 92:8 ratio, mp >250 °C (decomp.).

δ_H ^{1}H NMR (400 MHz, DMSO-d_6):	*major isomer* – 7.54 (d, *J* 2.4, 1H), 7.84 (s, 1H), 11.00 (br s, 1H), 11.67 (br s, 1H)
	minor isomer – 7.16 (d, *J* 2.2, 0.08H), 8.37 (s, 0.08H), 11.81 (br s, 0.08H)
δ_C ^{13}C NMR (100 MHz, DMSO-d_6):	*major isomer* - 102.7, 114.6, 129.2, 142.9, 143.0, 156.9, 162.7
	minor isomer – 102.5, 115.9, 127.9, 146.2, 146.3, 157.6, 163.5
ν_{max} (film)/cm^{-1}:	1578, 1671, 2625, 2971, 3088, 3676
HRMS (*m/z* –APCI):	Found: 192.0525 ([M - H]$^-$ $C_7H_6N_5O_2$; Requires: 192.0527)

2-Amino-4-oxo-4,7-dihydro-3*H*-pyrrolo[2,3-*d*]pyrimidine-5-carbaldehyde *O*-methyl oxime (22)

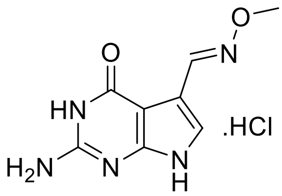

Prepared as per general procedure **C**, using 1M HCl in dioxane, to yield the title compound as a white powder (10 mg, 16% over two steps) containing an inseparable mixture of *E*/*Z* isomers in a 97:3 ratio, mp: > 250 °C (decomp.).

δ_H ^{1}H NMR (400 MHz, DMSO-d$_6$):	3.87 (s, 3H), 6.18 (br s, 2H, NH$_2$), 7.38 (s, 1H), 7.84 (s, 1H), 10.46 (br s, 1H, NH), 11.47 (br s, 1H, NH)
δ_C ^{13}C NMR (100 MHz, DMSO-d$_6$):	41.0, 97.8, 109.0, 123.0, 139.4, 150.6, 153.4, 159.5
ν_{max} (film)/cm^{-1}:	1053, 1593, 1672, 2854, 3132
HRMS (*m/z* –ESI):	Found: 230.0668 ([M + Na]$^+$ $C_8H_9N_5NaO_2$; Requires: 230.0648)

2-Amino-4-oxo-4,7-dihydro-3*H*-pyrrolo[2,3-*d*]pyrimidine-5-carbaldehyde *O*-benzyl oxime (23)

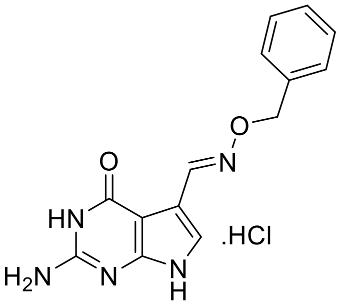

Prepared as per general procedure **C** using 1.25M HCl/MeOH to yield the title compound as a white powder (33 mg, 41% over two steps) containing an inseparable mixture of E/Z isomers in a 7:1 ratio, mp: > 250 °C (decomp.).

δ_H ^{1}H NMR (400 MHz, DMSO-d$_6$):	*major isomer* – 5.16 (s, 2H), 6.30 (bs, 2H), 7.25-7.37 (m, 5H), 7.40 (d, *J* 2.4, 1H), 7.89 (s, 1H), 10.57 (s, 1H), 11.49 (s, 1H)
	minor isomer – 5.03 (s, 2H), 6.30 (bs, 2H), 7.00 (d, *J* 2.5, 1H), 7.25-7.37 (m, 5H), 8.42 (s, 1H), 10.57 (s, 1H), 11.44 (s, 1H).

δ_C ^{13}C NMR (100 MHz, DMSO-d_6):	*major isomer* – 76.0, 98.0, 109.4, 124.1, 128.1, 128.2, 128.8, 138.6, 139.6, 146.1, 152.8, 158.4
ν_{max} (film)/cm^{-1}:	1571, 1671, 2112, 2780, 2901, 2988, 3128, 3676
HRMS (*m/z* –APCI):	Found: 284.1147 [M + H]$^+$; $C_{14}H_{14}N_5O_2$: Requires: 284.1142

2-Amino-4-oxo-4,7-dihydro-3*H*-pyrrolo[2,3-*d*]pyrimidine-5-carbaldehyde *O*-phenethyl oxime (24)

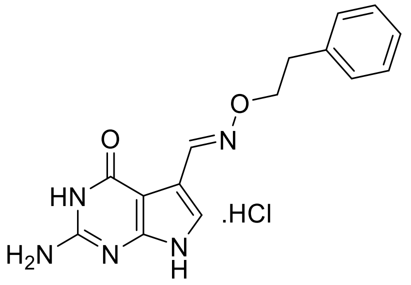

Prepared as per general procedure **C** using 1.25M HCl/MeOH to yield the title compound as a white powder (30 mg, 26% over two steps), mp: > 250 °C (decomp.).

δ_H ^{1}H NMR (400 MHz, DMSO-d_6):	2.97 (t, *J* 6.7, 2H), 4.32 (t, *J* 6.7, 2H), 6.30 (br s, 2H), 7.18-7.29 (m, 6H), 7.88 (s, 1H), 10.56 (s, 1H, NH), 11.58 (s, 1H, NH)
δ_C ^{13}C NMR (100 MHz, DMSO-d_6):	35.5, 49.0, 74.9, 97.9, 109.3, 124.0, 126.6, 128.7, 129.3, 139.2, 147.0, 152.8, 158.7
ν_{max} (film)/cm^{-1}:	1650, 1671, 2765, 2923
HRMS (*m/z* –APCI):	Found: 298.1298 [M + H]$^+$ $C_{15}H_{16}N_5O_2$; Requires: 298.1299

Amine hydrochloride salts were prepared by the general procedure:

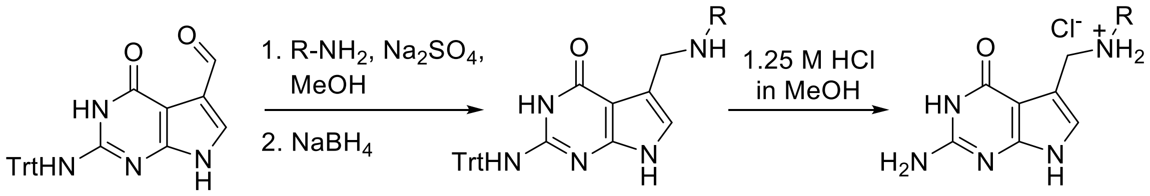

General procedure D: Synthesis amine hydrochloride salts

N-((2-amino-4-oxo-4,7-dihydro-3H-pyrrolo[2,3-d]pyrimidin-5-yl)methyl)-3-phenylpropan-1-aminium chloride (33)

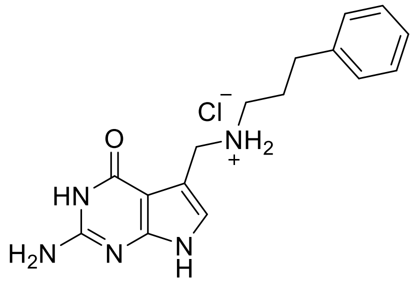

To a suspension of 4-oxo-2-(tritylamino)-4,7-dihydro-3H-pyrrolo[2,3-d]pyrimidine-5-carbaldehyde (200 mg, 0.48 mmol) and Na_2SO_4 (5 mg) in MeOH (5 mL) was added the appropriate amine, in this case 3-phenylpropylamine (0.52 mmol, 0.074 mL), and the resulting suspension stirred at room temperature until complete consumption of aldehyde starting material was observed by TLC analysis. Solid $NaBH_4$ (1.43 mmol, 55.00 mg) was added and the reaction mixture stirred at room temperature for a further hour. Water (5 mL) was added and the resulting suspension was stirred for 10 mins before being extracted with CH_2Cl_2 (3 x 5 mL). The combined organic layers were dried (MgSO4) and concentrated *in vacuo* to yield the crude product which was purified by flash chromatography (9:1 DCM:MeOH) to yield the desired compound as a white solid (210 mg, 42%), mp > 300 °C (decomp.).

A solution of the trityl-protected compound (210.00 mg, 0.39 mmol) in 1.25 M methanolic HCl (3 mL) was prepared and stirred at room temperature for 16 h. The precipitated product was collected by filtration and washed with CH_2Cl_2 to yield the title compound as a white powder, (84 mg, 29 % over two steps), mp > 300 °C (decomp).

δ_H ^{1}H NMR (600 MHz, DMSO-d6):	1.90 (app qt, 2H), 2.63 (t, *J* 7.8, 2H), 2.90 (app qt, 2H), 4.13 (t, *J* 5.2, 2H), 6.57 (br s, 2H, NH_2), 6.80 (d, *J* 2.3, 1H), 7.16 (m, 3H), 7.26 (m, 2H), 9.11 (br s, 2H, NH_2), 11.05 (br s, 1H, NH), 11.31 (br s, 1H, NH).
δ_C ^{13}C NMR (150 MHz, DMSO-d6):	27.6, 32.1, 42.9, 45.6, 48.9, 98.6, 108.7, 117.9, 126.4, 128.6, 128.7, 140.9, 152.9, 160.5
ν_{max} (film)/cm^{-1}:	1456, 1625, 2443, 2713, 2756, 2873, 2933, 3184
HRMS (*m/z* ESI$^+$):	Found: 298.1662 M$^+$; $C_{16}H_{20}N_5O$ Requires: 298.1668

N-((2-Amino-4-oxo-4,7-dihydro-3H-pyrrolo[2,3-d]pyrimidin-5-yl)methyl)cyclopentanaminium chloride (25)

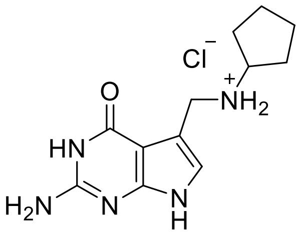

Prepared as per general procedure **D** to yield the title compound as a white powder (20 mg, 12%), mp: > 250 °C (decomp.).

δ_H ^{1}H NMR (400 MHz, DMSO-d_6):	1.52-1.56 (m, 2H), 1.62-1.71 (m, 5H), 1.89-1.97 (m, 2H), 4.13 (t, *J* 5.5, 2H), 6.56 (br s, 2H, NH_2), 6.83 (d, *J* 2.1, 1H), 9.11 (br s, 2H, NH_2), 11.01 (br s, 1H), 11.31 (br s, 1H)
δ_C ^{13}C NMR (100 MHz, DMSO-d_6):	24.0, 29.6, 41.5, 57.8, 99.0, 109.6, 118.6, 146.8, 152.7, 159.6
ν_{max} (film)/cm^{-1}:	1597, 1681, 2902, 2972, 2988, 3104, 3676
HRMS (*m/z* –APCI):	Found: 246.1358 [M – 2H]$^-$ $C_{12}H_{16}N_5O$; Requires: 246.1360

(R)-N-((2-amino-4-oxo-4,7-dihydro-3H-pyrrolo[2,3-d]pyrimidin-5-yl)methyl)-2,3-dihydroxypropan-1-aminium chloride (26)

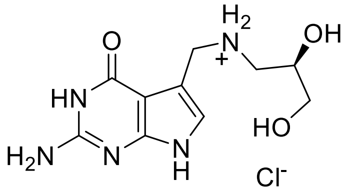

Prepared as per general procedure **D** to yield the title compound as a white powder (25 mg, 48%), mp >250 °C (decomp.), $[\alpha]^{20}_D$ -7.6 (0.5, MeOH).

δ_H ^{1}H NMR (400 MHz, DMSO-d_6):	2.79-2.91 (m, 1H), 2.99-3.02 (m, 1H), 3.23 (dd, *J* 6.6, 11.2, 1H), 3.36 (dd, *J* 6.6, 11.2, 1H), 3.72-3.73 (m, 1H), 4.14 (t, *J* 4.8, 2H), 6.80 (d, 1H, *J* 2.1), 9.12 (br m, 2H), 11.07 (br s, 1H), 11.32 (br d, *J* 2.1, 1H)

δ_C ^{13}C NMR (100 MHz, DMSO-d_6):	42.9, 49.4, 63.8, 67.6, 99.0, 109.2, 118.8, 146.6, 152.6, 159.4
ν_{max} (film)/cm^{-1}:	1092, 1217, 1229, 1366, 1671, 1738, 2970, 3321 (broad)
HRMS (*m/z* -ES):	Found: 254.1243 M^+; $C_{10}H_{16}N_5O_3$; requires: 254.1248

(*S*)-*N*-((2-amino-4-oxo-4,7-dihydro-3H-pyrrolo[2,3-d]pyrimidin-5-yl)methyl)-2,3-dihydroxypropan-1-aminium chloride (27)

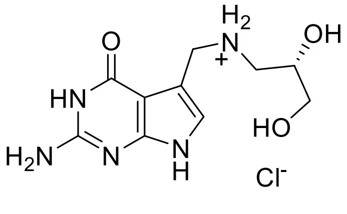

Prepared as per general procedure **D** to yield the title compound as a white powder (40 mg, 50%), mp >250 °C (decomp.); $[\alpha]^{20}_D$ +7.4 (0.5, $CHCl_3$).

δ_H ^1H NMR (400 MHz, DMSO-d_6):	2.74-2.80 (m, 1H), 2.97-3.02 (m, 1H,), 3.22 (dd, *J* 6.6, 11.1, 1H), 3.35 (dd, *J* 6.6, 11.1, 1H), 3.72-3.75 (m, 1H), 4.14 (t, *J* 4.8, 2H), 6.83 (d, *J* 2.1, 1H), 9.18 (br t, *J* 4.8, 2H, NH_2), 11.33 (br s, 1H, , NH), 11.48 (br d, *J* 2.1, 1H, NH)

δ_C ^{13}C NMR (100 MHz, DMSO-d_6):	43.0, 49.4, 63.8, 67.6, 98.9, 109.1, 118.7, 147.3, 152.6, 159.6
ν_{max} (film)/cm^{-1}:	1092, 1217, 1229, 1366, 1676, 1738, 2755, 2970, 3323 (broad)
HRMS (*m/z* -ES):	Found: 254.1247 (M^+; $C_{10}H_{16}N_5O_3$; requires: 254.1248)

(1S,2R)-N-((2-amino-4-oxo-4,7-dihydro-3H-pyrrolo[2,3-d]pyrimidin-5-yl)methyl)-2-hydroxy-2,3-dihydro-1H-inden-1-aminium chloride (29)

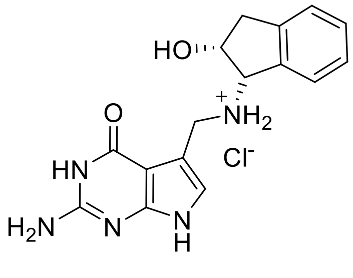

Prepared as per general procedure **D** to yield the title compound as a pale red powder (54 mg, 85%).

δ_H ^{1}H NMR (400MHz, DMSO-d_6):	2.97 (dd, *J* 5.9, 16.1, 1H), 3.09 (dd, *J* 5.9, 16.1, 1H), 4.27-4.31 (m, 2H), 4.51 (app q, 1H), 4.66 (app q, 1H), 6.93 (s, 1H), 7.26-7.36 (m, 3H), 7.55 (d, *J* 7.4, 1H), 9.15-9.08 (m, 1H), 9.80-9.88 (m, 1H), 11.18 (s, 1H), 11.36 (s, 1H)
HRMS (*m/z* -ES):	Found 312.1469 (M^+; $C_{16}H_{18}N_5O_2$ requires 312.1461)

N-((2-Amino-4-oxo-4,7-dihydro-3H-pyrrolo[2,3-d]pyrimidin-5-yl)methyl)-2-methylpropan-2-aminium chloride (30)

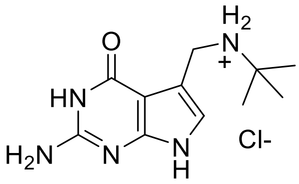

Prepared as per general procedure **D** to yield 137 as a white powder (7 mg, 42%), mp: >250 °C (decomp.).

δ_H ^{1}H NMR (400MHz, DMSO-d_6):	1.32 (s, 9H), 4.10 (t, *J* 5.3, 2H), 6.49 (br s, 2H), 6.82 (d, *J* 2.1, 1H), 9.04 (br m, 2H), 10.98 (br s, 1H)
δ_C ^{13}C NMR (100 MHz, DMSO-d_6):	25.8, 37.3, 56.4, 98.9, 117.8, 118.0, 148.9, 152.8, 160.1
ν_{max} (film)/cm^{-1}:	1366, 1680, 2664, 2740, 2970, 3300 (broad)
HRMS (*m/z* -ES):	Found: 236.1511 (M^+; $C_{11}H_{18}N_5O$; requires: 236.1506)

N-((2-amino-4-oxo-4,7-dihydro-3H-pyrrolo[2,3-d]pyrimidin-5-yl)methyl)-2-phenylethanaminium chloride (31)

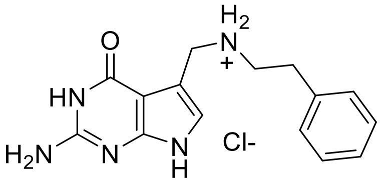

Prepared as per general procedure **D** using to yield the title compound as a white powder (80 mg, 77%), mp: > 250 °C (decomp.).

δ_H ^{1}H NMR (400 MHz, DMSO-d_6):	2.95 (t, *J* 7.2, 2H), 3.10-3.17 (m, 2H), 4.18 (t, *J* 5.4, 2H), 6.86 (d, *J* 2.3, 1H), 7.20-7.23 (m, 3H), 7.27-7.31 (m, 2H), 9.25 (br s, 2H), 11.19 (br s, 1H), 11.43 (br d, *J* 2.3, 1H)
δ_C ^{13}C NMR (100 MHz, DMSO-d_6):	31.9, 42.4, 47.2, 98.8, 109.2, 118.8), 127.2, 129.0, 129.1, 146.7, 146.8, 152.6, 159.4

ν_{max} (film)/cm^{-1}: 1313, 1625, 1667, 2427, 2710, 2932, 3177 (broad)

HRMS (*m/z* -ES): Found: 282.1367 ([M$^+$–2H]$^-$ $C_{15}H_{16}N_5O$; requires: 282.1360)

2-Amino-5-(((benzyloxy)amino)methyl)-3,7-dihydro-4*H*-pyrrolo[2,3-*d*]pyrimidin-4-one (32)

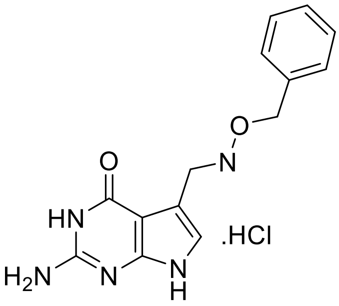

To a solution of 4-Oxo-2-(tritylamino)-4,7-dihydro-3*H*-pyrrolo[2,3-*d*]pyrimidine-5-carbaldehyde and *O*-benzylhydroxylamine (50 mg, 0.18 mmol) in CH_2Cl_2 (5 cm^3) was added $NaCNBH_3$ (23 mg, 0.36 mmol). Methanolic HCl (1.25 M) was added dropwise to adjust the pH to approximately 3. The resulting solution was stirred at room temperature for 3 h, with care taken to ensure the pH was maintained at 3. Additional methanolic HCl was added when necessary. The reaction mixture was then diluted with H_2O (15 cm^3) and extracted with CH_2Cl_2 (3 x 10 cm^3). The combined organic extracts were dried ($MgSO_4$) and concentrated *in vacuo* before being taken up into 1 M methanolic HCl and stirred at room temperature for 1 h. The precipitated product was isolated by vacuum filtration and washed with Et_2O to yield the title compound as a white powder (11 mg, 12%), mp: > 250 °C (decomp.).

δ_H ^{1}H NMR (400 MHz, DMSO-d$_6$): 4.32 (s, 2H), 5.01 (s, 2H), 6.34 (br s, 2H), 6.79 (s, 1H), 7.35-7.36 (m, 5H), 10.86 (br s, 1H), 11.24 (br s, 1H)

δ_C ^{13}C NMR (100 MHz, DMSO-d$_6$): 44.6, 75.1, 99.0, 107.1, 128.4, 129.0, 129.7, 130.3, 134.0, 146.5, 152.6, 159.4

ν_{max} (film)/cm^{-1}: 1604, 1672, 2764, 2971

HRMS (*m/z* –APCI): Found: 286.1299 ([M + H]$^+$ $C_{14}H_{16}N_5O_2$; Requires: 286.1292)

***N*-((2-amino-4-oxo-4,7-dihydro-3*H*-pyrrolo[2,3-*d*]pyrimidin-5-yl)methyl)decan-1-aminium chloride (34)**

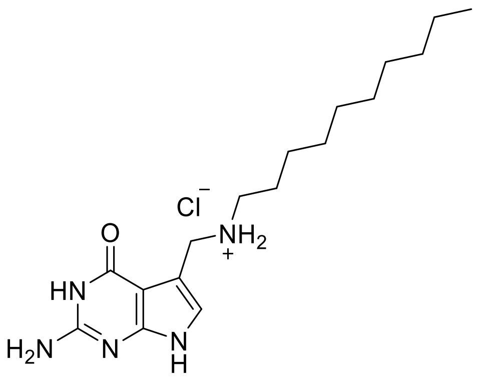

Prepared as per general procedure **D** to yield the title compound as a white powder (23 mg, 74%), mp: > 250 °C (decomp.).

δ_H ^{1}H NMR (400 MHz, DMSO-d$_6$):	0.82 (t *J* 6.6, 3H), 1.20-1.26 (m, 14H), 1.56 (app quintet, 2H), 2.87-2.89 (m, 2H), 4.11 (t, *J* 5.3, 2H), 6.33 (br s, 2H, NH_2), 6.78 (d *J* 1.8, 1H), 8.91 (br s, 2H, NH_2), 10.87 (br s, 1H, NH), 11.24 (br s, 1H, NH)
δ_C ^{13}C NMR (100 MHz, DMSO-d$_6$):	14.0, 22.1, 25.4, 25.8, 28.5, 28.7, 28.8, 28.9, 31.3, 42.4, 45.7, 98.3, 108.7, 117.7, 149.5, 152.5, 159.8
ν_{max} (film)/cm^{-1}:	1066, 1406, 1578, 1655, 2921, 1655, 2921, 2987, 3685
HRMS (*m/z* -ESI):	Found: 320.2446 (M^+; $C_{17}H_{30}N_5O$; Requires: 320.2445)

***N*-((2-Amino-4-oxo-4,7-dihydro-3*H*-pyrrolo[2,3-*d*]pyrimidin-5-yl)methyl)dodecan-1-aminium chloride (35)**

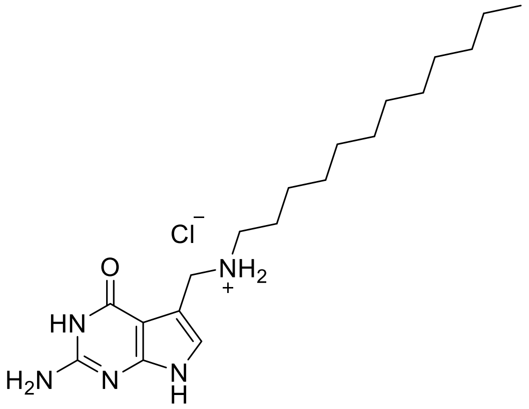

Prepared as per general procedure **D** to yield the title compound as a white powder (92 mg, 83%), mp: > 250 °C (decomp.).

δ_H ^{1}H NMR (400 MHz, DMSO-d_6):	0.82 (t, *J* 6.9, 3H), 1.15-1.30 (m, 18H), 1.56 (m, 2H), 2.88 (m, 2 H), 4.11 (t, *J* 5.4, 2H), 6.37 (br s, 2H, NH_2), 6.79 (s, 1H), 8.93 (br s, 2H, NH_2), 10.90 (br s, 1H, NH), 11.25 (br s, 1H, NH)
δ_C ^{13}C NMR (100 MHz, DMSO-d_6):	14.0, 22.1, 25.5, 25.9, 28.5, 28.7, 28.8, 28.9, 29.0, 29.1, 31.3, 42.3, 45.7, 98.4, 108.8, 118.0, 148.5, 152.4, 159.6
ν_{max} (film)/cm^{-1}:	1244, 1577, 1671, 2921, 3098, 3270, 3677
HRMS (*m/z* -ESI):	Found: 348.2752 (M^+; $C_{19}H_{34}N_5O$; Requires: 348.2758)

N-((2-Amino-4-oxo-4,7-dihydro-3*H*-pyrrolo[2,3-*d*]pyrimidin-5-yl)methyl)tetradecan-1-aminium chloride (36)

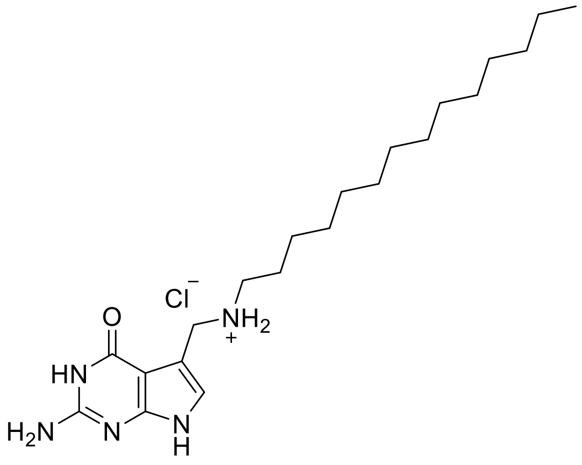

Prepared as per general procedure **D** to yield the title compound as a white powder (80 mg, 53%), mp: > 250 °C (decomp.).

δ_H ^{1}H NMR (400 MHz, DMSO-d_6):	0.82 (t, *J* 6.8, 3H), 1.16-1.28 (m, 22H), 1.56 (app quintet, 2H), 2.86-2.91 (m, 2H), 4.11 (t, *J* 5.3, 2H), 6.32 (br s, 2H, NH_2), 6.78 (d, *J* 2.1, 1H), 8.89 (br s, 2H, NH_2), 10.85 (br s, 1H, NH), 11.24 (br s, 1H, NH)
ν_{max} (film)/cm^{-1}:	1133, 1544, 1671, 2429, 2920, 3269, 3676
HRMS (*m/z* -ESI):	Found: 376.3067 (M^+; $C_{21}H_{38}N_5O$; Requires: 376.3071)

N-((2-Amino-4-oxo-4,7-dihydro-3*H*-pyrrolo[2,3-*d*]pyrimidin-5-yl)methyl)hexadecan-1-aminium chloride (37)

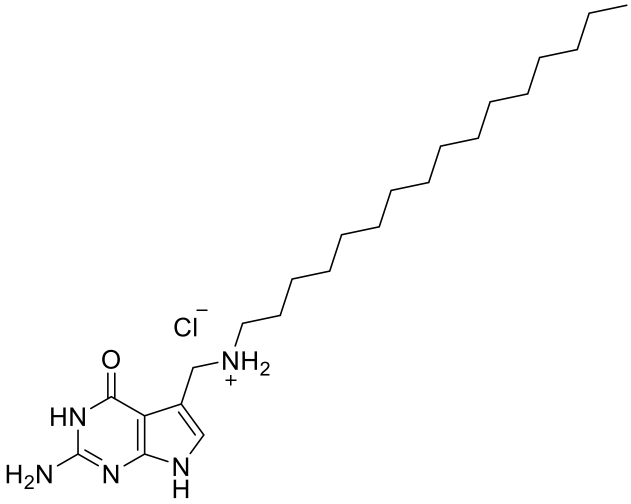

Prepared as per general procedure **D** to yield the title compound as a white powder (65 mg, 61%), mp: > 250 °C (decomp.).

δ_H ^1H NMR (400 MHz, DMSO-d_6):	0.84 (t, *J* 6.1, 3H), 1.17-1.29 (m, 26H), 1.58 (app quintet, 2H), 2.85-2.92 (m, 2H), 4.12 (t, *J* 5.2, 2H), 6.49 (br s, 2H, NH_2), 6.81 (d, *J* 2.1, 1H), 9.02 (br s, 2H, NH_2), 11.01 (br s, 1H, NH), 11.30 (br s, 1H, NH)
ν_{max} (film)/cm^{-1}:	1243, 1578, 2671, 2427, 2920, 3269, 3676
HRMS (*m/z* -ESI):	Found: 404.3391 (M^+; $C_{23}H_{42}N_5O$; Requires: 404.3384)

***N*-((2-amino-4-oxo-4,7-dihydro-3*H*-pyrrolo[2,3-*d*]pyrimidin-5-yl)methyl)octadecan-1-aminium chloride (38)**

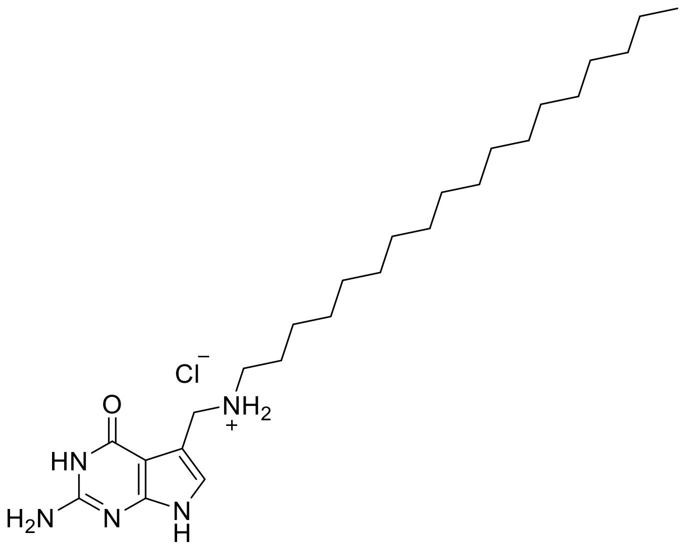

Prepared as per general procedure **D** to yield the title compound as a white powder (90 mg, 77%), mp: > 250 °C (decomp.).

δ_H ^{1}H NMR (400 MHz, DMSO-d_6):	0.83 (t, 3H), 1.21 (app s, 28H), 1.57 (app quintet, 2H), 4.12 (t, *J* 5.2, 2H), 6.39 (br s, 2H, NH_2), 6.81 (d, 1H, *J* 1.8), 8.97 (br s, 2 H, NH_2), 10.92 (br s, 1H, NH), 11.27 (br s, 1H, NH)
ν_{max} (film)/cm^{-1}:	1051, 1313, 1578, 1671, 2919, 2987, 3268, 3676
HRMS (*m/z* -ESI):	Found: 432.3696 (M^+; $C_{25}H_{46}N_5O$; Requires: 432.3697)

N-((2-amino-4-oxo-4,7-dihydro-3H-pyrrolo[2,3-d]pyrimidin-5-yl)methyl)-N-methyl-3-phenylpropan-1-aminium (39)

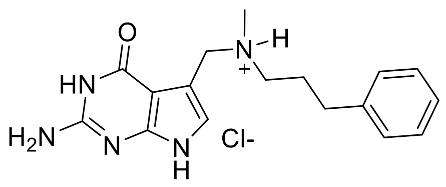

Prepared as per general procedure **D** to yield the title compound as a white powder (30 mg, 10%).

δ_H ^{1}H NMR (400 MHz, DMSO-d_6):	2.00-2.07 (m, 2 H), 2.62 (t, 2H, *J* 7.7, 2H), 2.72 (app d, 3H), 2.95-3.03 (m, 1H), 3.12-3.21 (m, 1H), 4.24 (dd, *J* 5.1, 13.6, 1H), 4.37(dd, *J* 4.2, 13.6, 1H), 6.94 (s, 1H), 7.20-7.23 (m, 3H), 7.27-7.31 (m, 2H), 10.10 (s, 1H, NH), 11.21 (br s, 1H, NH), 11.58 (s, 1H, NH)
HRMS (*m/z* -ESI):	Found 312.1825, (M^+ $C_{17}H_{22}N_5O$ requires 312.1819)

2-amino-5-(((3-cyclohexylpropyl)amino)methyl)-3,7-dihydro-4H-pyrrolo[2,3-d]pyrimidin-4-one (40)

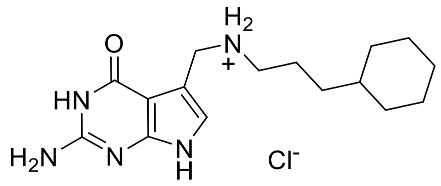

Prepared as per general procedure **D** to yield the title compound as a white powder (75mg, 30%).

δ_H ^{1}H NMR (400 MHz, DMSO-d_6):	0.84-0.89 (m, 2H), 1.15-1.21 (m, 6H), 1.60-1.68 (m, 7H), 2.85-2.92 (m, 2H), 4.15 (m, 2H), 6.8 (s, 1H), 9.13 (s, 2H, NH_2), 11.23 (s, 1H, NH), 11.42(br s, 1H, NH)
HRMS (*m/z* -ESI):	Found 304.2137, (M^+ $C_{16}H_{26}N_5O$ requires 304.2132)

***N*-((2-amino-4-oxo-4,7-dihydro-3H-pyrrolo[2,3-*d*]pyrimidin-5-yl)methyl)-2-phenoxyethan-1-aminium chloride (41)**

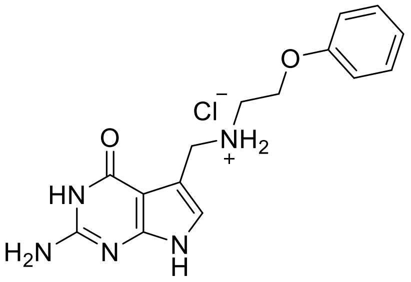

Prepared as per general procedure **D** to yield the title compound as a white powder (20 mg, 20%), mp: > 250 °C (decomp.).

δ_H ^{1}H NMR (400 MHz, DMSO-d_6):	3.38 (2H, under H_2O signal, visible by H-H COSY), 4.20-4.25 (m, 4H), 6.42 (br s, 2H, NH_2), 6.83 (d, *J* 2.2, 1H), 6.95-6.98 (m, 3H), 7.29 (t, *J* 7.9, 2H), 9.34 (br s, 2H, NH_2), 11.02 (br s, 1H, NH), 11.29 (br s, 1H, NH)
δ_C ^{13}C NMR (150 MHz, DMSO-d_6):	43.3, 45.5, 63.6, 98.8, 109.0, 115.1, 118.3, 121.7, 130.0, 150.6, 153.0, 158.1, 160.4
ν_{max} (film)/cm^{-1}:	1057, 1075, 1243, 1407, 1675, 2902, 2972, 3193, 3676
HRMS (*m/z* -ESI):	Found: 300.1449 (M^+; $C_{15}H_{18}N_5O_2$; Requires: 300.1455)

***N*-((2-Amino-4-oxo-4,7-dihydro-3*H*-pyrrolo[2,3-*d*]pyrimidin-5-yl)methyl)-2-(phenylthio)ethan-1-aminium chloride (42)**

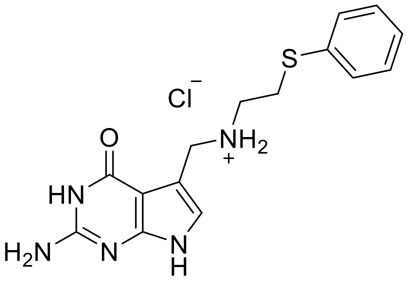

Prepared as per general procedure **D** to yield the title compound as a white powder (20 mg, 20%), mp: > 250 °C (decomp.).

δ_H ^{1}H NMR (400 MHz, DMSO-d_6):	3.04 (m, 2H), 3.22 (t, *J* 6.9, 2H), 4.16 (t, *J* 4.7, 2H), 6.35 (br s, 2 H, NH_2), 6.76 (d, *J* 1.9, 1H), 7.19 (tt, *J* 6.9 1.4, 1H), 7.26-7.34 (m, 4H,), 9.11 (br s, 2H, NH_2), 10.89 (s, 1H, NH), 11.23 (br s, 1 H, NH)
δ_C ^{13}C NMR (100 MHz, DMSO-d_6):	28.0, 42.3, 44.4, 98.3, 108.2, 117.9, 126.5, 128.8, 129.2, 133.7, 150.1, 152.6, 159.8
ν_{max} (film)/cm^{-1}:	1075, 1249, 1394, 1675, 2901, 2972, 3675
HRMS (*m/z* -ESI):	Found: 316.1227 (M^+; $C_{15}H_{18}N_5O$; Requires: 316.1227)

***N*-((2-Amino-4-oxo-4,7-dihydro-3*H*-pyrrolo[2,3-*d*]pyrimidin-5-yl)methyl)-2-(phenylsulfonyl)ethan-1-aminium chloride (43)**

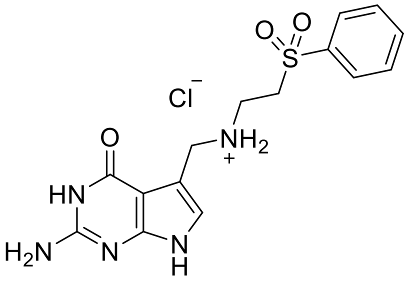

Prepared as per general procedure **D** to yield the title compound as a white powder (50 mg, 20%), mp: > 250 °C (decomp.).

δ_H ^{1}H NMR (400 MHz, DMSO-d_6):	3.13 (app s, 2H), 3.70 (t, *J* 7.3, 2H), 4.16 (app s, 2H,), 6.29 (br s, 2H, NH_2), 6.74 (s, 1H), 7.65 (app t, 2H), 7.76 (t, *J* 6.30, 1H), 7.87 (t, *J* 7.7, 2H), 9.0 (br s, 2H, NH_2), 10.79 (s, 1H, NH), 11.23 (s, 1H, NH)
δ_C ^{13}C NMR (100 MHz, DMSO-d_6):	0.2, 42.3, 51.2, 98.9, 108.3, 119.1, 128.2, 130.1, 134.9, 138.2, 147.7, 152.8, 159.4
ν_{max} (film)/cm^{-1}:	1075, 1148, 1308, 1662, 2901, 2972, 3685
HRMS (*m/z* -ESI):	Found: 348.1131 (M^+; $C_{15}H_{18}N_5O$; Requires: 348.1125)

***N*-((2-amino-4-oxo-4,7-dihydro-3*H*-pyrrolo[2,3-*d*]pyrimidin-5-yl)methyl)-2-(phenylamino)ethan-1-aminium chloride (44)**

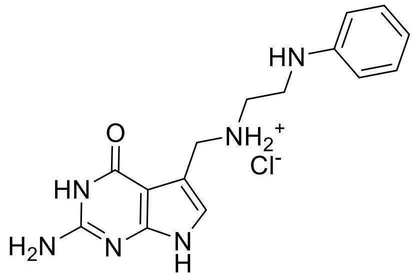

Prepared as per general procedure **D**, except the title compound does not precipitate well from methanolic HCl. Reaction mixture was concentrated *in vacuo* and the resulting residue triturated in hexane followed by Et_2O to yield the title compound as a white powder (25 mg, 93%), mp: > 250 °C (decomp.).

δ_H ^{1}H NMR (400 MHz, DMSO-d_6):	3.05 (m, 2H), 3.31 (t,*J* 6.3, 2H), 4.18 (app s, 2H), 6.32 (br s, 2H, NH_2), 6.54 (m, 3H), 6.79 (d, *J* 1.7, 1H), 7.06 (t, *J* 7.8, 2H), 9.00 (m, 2H, NH_2), 10.89 (d, *J* 1.7, 1H, NH), 11.24 (br s, 1H NH)
δ_C ^{13}C NMR (100 MHz, DMSO-d_6):	41.4, 42.3, 44.1, 99.1, 109.1, 115.5, 119.2, 120.4, 128.0, 129.7, 145.2, 152.5 159.1
ν_{max} (film)/cm^{-1}:	1057, 1075, 1243, 1407, 1675, 2902, 2972, 3193, 3676
HRMS (*m/z* -ES):	Found: 299.1635 (M$^+$; $C_{15}H_{19}N_6O$; Requires: 299.1635)

2-Amino-5-((phenethoxyamino)methyl)-3,7-dihydro-4*H*-pyrrolo[2,3-*d*]pyrimidin-4-one (45)

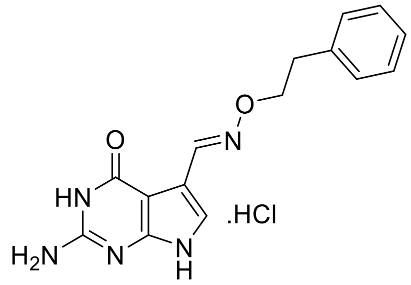

To a solution of 4-Oxo-2-(tritylamino)-4,7-dihydro-3*H*-pyrrolo[2,3-*d*]pyrimidine-5-carbaldehyde and *O*-phenethylhydroxylamine (130 mg, 0.25 mmol) in CH_2Cl_2 (5 cm^3) was added $NaCNBH_3$ (32 mg, 0.51 mmol). Methanolic HCl (1.25 M) was added dropwise to adjust the pH to approximately 3. The resulting solution was stirred at room temperature for 3 h, with care taken to ensure the pH was maintained at 3. Additional methanolic HCl was added when necessary. The reaction mixture was then diluted with H_2O (15 cm^3) and extracted with CH_2Cl_2 (3 x 10 cm^3). The combined organic extracts were dried ($MgSO_4$) and concentrated *in vacuo* before being taken up into 1 M HCl in dioxane and

stirred at room temperature for 1 h. The precipitated product was isolated by vacuum filtration and washed with Et_2O to yield the title compound as a white powder (35 mg, 50%), 250 °C (decomp.).

δ_H ^{1}H NMR (400 MHz, DMSO-d_6):	2.90 (t, *J* 6.6, 2H), 4.28 (t, *J* 6.6, 2H), 4.43 (s, 2H), 6.44 (br s, 2H, NH_2), 6.79 (d, *J* 2.2, 1H), 7.17-7.27 (m, 5H), 10.97 (br s, 1 H, NH), 11.30 (br s, 1H, NH)
δ_C ^{13}C NMR (100 MHz, DMSO-d_6):	35.4, 35.6, 66.8, 98.9, 111.4, 117.2, 125.2, 128.2, 129.2, 129.9, 148.3, 125.7, 160.0
ν_{max} (film)/cm^{-1}:	1670, 2531, 3024, 3261
HRMS (*m/z* –APCI):	Found: 300.1462 ($[M + H]^+$ $C_{15}H_{18}N_5O_2$; Requires: 300.1455)

N-((2-amino-4-oxo-4,7-dihydro-3H-pyrrolo[2,3-d]pyrimidin-5-yl)methyl)-3-oxo-3-phenylpropan-1-aminium (46)

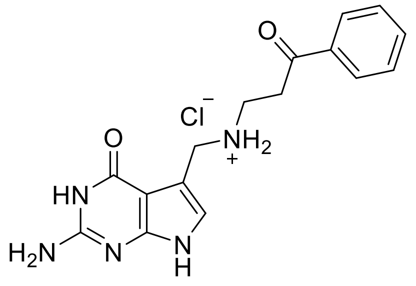

Prepared as per general procedure **D** to yield the title compound as a white powder (53 mg, 32%), mp: > 250 °C (decomp.).

δ_H ^{1}H NMR (400 MHz, DMSO-d_6):	3.26 (m, 2H), 3.48 (t, *J* 6.6, 2H), 4.20 (t, *J* 5.3, 2H), 6.40 (br s, 2H, NH_2), 6.82 (d, 1H, *J* 2.1), 7.53 (t, *J* 7.9, 2H), 7.65 (m, 1H), 7.92 (m, 2H), 8.99 (br s, 2H, NH_2), 10.90 (br s, 1H, NH), 11.27 (br s, 1H)
δ_C ^{13}C NMR (100 MHz, DMSO-d_6):	34.9, 41.3, 42.8, 99.0, 109.1, 118.8, 128.4, 129.3, 134.1, 136.3, 147.3, 152.7, 159.4, 197.4
ν_{max} (film)/cm^{-1}:	1579, 1623, 1671, 2773, 2901, 2988, 3342, 3676
HRMS (*m/z* -APCI):	Found: 312.1453 (M^+; $C_{16}H_{18}N_5O_2$; Requires: 312.1455)

***N*-((2-amino-4-oxo-4,7-dihydro-3*H*-pyrrolo[2,3-*d*]pyrimidin-5-yl)methyl)-3-methyl-3-phenylbutan-1-aminium chloride (47)**

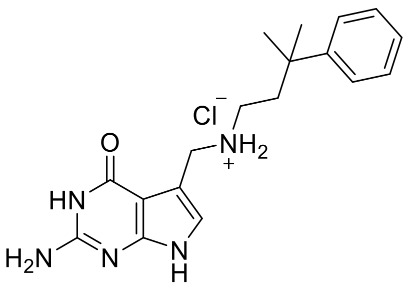

Prepared as per general procedure **D**, however, the title compound is soluble in methanol and, thus, does not precipitate to a large extent. Et_2O was added to induce selective precipitation of the desired product. The product was filtered and washed with Et_2O to yield the title compound as a white powder (18 mg, 51%), mp > 300 °C.

δ_H ^{1}H NMR (400 MHz, DMSO-d$_6$): 1.23 (s, 6 H), 1.93-1.97 (m, 2 H,), 2.60-2.63 (m, 2 H), 4.05 (t, *J* 5.1, 2 H), 6.42 (br s, 2H, NH_2), 6.71 (d, *J* 1.9, 1H), 7.14 (m, 1H), 7.23-7.32 (m, 4H), 8.95 (br s, 2H,NH_2), 10.91 (bs, 1H), 11.21 (br s, 1H, NH)

δ_C ^{13}C NMR (100 MHz, DMSO-d$_6$): 29.1, 36.9, 39.1, 42.1, 42.8, 98.8, 109.2, 118.7, 125.9, 126.3, 128.7, 146.9, 147.7, 152.6, 159.4

ν_{max} (film)/cm^{-1}: 1066, 1551, 1654, 2901, 2987, 3685

HRMS (*m/z* -ESI): Found: 326.1981 (M^+; $C_{18}H_{24}N_5O$; Requires: 326.1975)

***N*-((2-amino-4-oxo-4,7-dihydro-3*H*-pyrrolo[2,3-*d*]pyrimidin-5-yl)methyl)-2-methyl-3-phenylbutan-1-aminium chloride (48)**

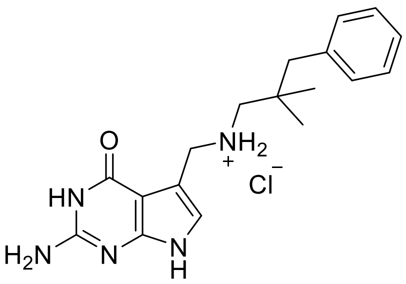

Prepared as per general procedure **D** to yield the title compound as a white powder (70 mg, 78 %), mp: > 250 °C (decomp.).

δ_H ^{1}H NMR (400 MHz, DMSO-d$_6$): 0.89 (s, 6H), 2.57 (s, 2H), 2.79-2.83 (m, 2H), 4.16 (t, *J* 5.5, 2H), 6.45 (br s, 2H, NH_2), 6.82 (s, 1H), 7.09 (d, *J* 7.2, 2H), 7.17-7.26

	(m, 3H), 9.07 (br s, 2H, NH_2), 11.09 (br s, 1H, NH), 11.30 (br s, 1H, NH)
δ_C ^{13}C NMR (100 MHz, DMSO-d_6):	24.8, 34.2, 43.3, 45.5, 56.3, 99.1, 109.1, 118.5, 126.7, 128.3, 130.9, 137.6, 149.1, 152.9, 160.2
ν_{max} (film)/cm^{-1}:	057, 1249, 1552, 1683, 2901, 2988, 3685
HRMS (*m/z* -ESI):	Found: 326.1969 (M^+; $C_{18}H_{24}N_5O$; Requires: 326.1975)

***N*-((2-amino-4-oxo-4,7-dihydro-3*H*-pyrrolo[2,3-*d*]pyrimidin-5-yl)methyl)-1-methyl-3-phenylbutan-1-aminium chloride (49)**

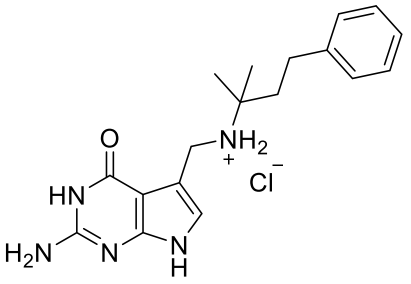

Prepared as per general procedure **D** to yield the title compound as a white powder (56 mg, 44%), mp: > 250 °C (decomp.).

δ_H ^{1}H NMR (400 MHz, DMSO-d_6):	1.37 (s, 6H), 1.89-1.93 (m, 2H), 2.64-2.68 (m, 2H), 4.13-4.16 (m, 2H), 6.71 (br s, 2H), 6.85 (s, 1H), 7.15 (t, *J* 7.2, 1H), 7.18-7.28 (m, 4H), 9.16 (br s, 2H, NH_2), 11.08 (br s, 1H, NH), 11.31 (br s, 1H, NH)
δ_C ^{13}C NMR (100 MHz, DMSO-d_6):	23.5, 29.5, 37.2, 59.0, 98.9, 118.2, 126.4, 128.7, 128.8, 141.7, 148.9, 152.9, 160.2
ν_{max} (film)/cm^{-1}:	1074, 1241, 1380, 1683, 2901, 2972, 3675
HRMS (*m/z* -ESI):	Found: 326.1995 (M^+; $C_{18}H_{24}N_5O$; Requires: 326.1981)

***N*-((2-Amino-4-oxo-4,7-dihydro-3*H*-pyrrolo[2,3-*d*]pyrimidin-5-yl)methyl)-2-(1-phenylcyclopropyl) ethan-1-aminium chloride (50)**

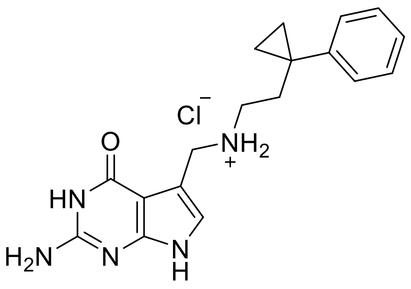

Prepared as per general procedure **D** to yield the title compound as a white powder (21 mg, 16 %) mp: > 250 °C (decomp.).

δ_H ^{1}H NMR (400 MHz, DMSO-d$_6$):	0.73-0.78 (m, 4 H), 1.89-1.93 (m, 2 H), 2.81-2.83 (m, 2H), 4.09 (t, *J* 5.2, 2H), 6.38 (br s, 2 H, NH_2), 6.74 (d, *J* 2.1, 1H), 7.13-7.17 (m, 5H), 8.90 (br s, 2H, NH_2), 10.91 (br s, 1H, NH), 11.23 (br s, 1H, NH)
δ_C ^{13}C NMR (100 MHz, DMSO-d$_6$):	13.8, 23.1, 35.4, 43.0, 44.5, 98.7, 108.9, 118.1, 126.7, 128.4, 128.8, 141.2, 143.6, 153.1, 160.5
ν_{max} (film)/cm^{-1}:	1470, 1675, 2644
HRMS (*m/z* –APCI):	Found: 324.1811 (M^+; $C_{18}H_{22}N_5O$; Requires: 324.1819)

***N*-((2-Amino-4-oxo-4,7-dihydro-3*H*-pyrrolo[2,3-*d*]pyrimidin-5-yl)methyl)-1-phenethylcyclopropan-1-aminium chloride (51)**

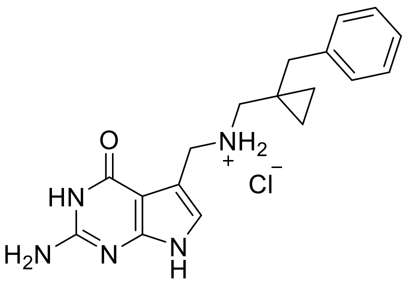

Prepared as per general procedure **D** to yield the title compound as a white powder (15 mg, 68%), mp: > 250 °C (decomp.).

δ_H ^{1}H NMR (400 MHz, DMSO-d$_6$):	0.57-0.65 (m, 4H), 2.70-2.76 (m, 4H), 4.12 (t, *J* 5.2, 2H), 6.46 (br s, 2H), 6.79 (d, *J* 1.8, 1H), 7.16-7.26 (m, 5H), 9.26 (br s, 2H), 11.13 (br s, 1H), 11.29 (br s, 1H,)
δ_C ^{13}C NMR (100 MHz, DMSO-d$_6$):	11.2, 19.3, 38.8, 43.1, 52.0, 99.0, 109.2, 118.7, 126.7, 128.6, 129.7, 139.1, 148.2, 152.1, 160.1

ν_{max} (film)/cm^{-1}: 1056, 1230, 1394, 1674, 2901, 2988, 3676

HRMS (*m/z* -ESI): Found: 324.1817 (M^+; $C_{18}H_{22}N_5O$; Requires: 324.1819)

(*R*)-*N*-((2-amino-4-oxo-4,7-dihydro-3*H*-pyrrolo[2,3-*d*]pyrimidin-5-yl)methyl)-3-phenylbutan-1-aminium chloride (52)

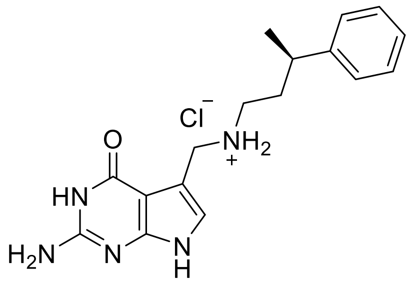

Prepared as per general procedure **D** to yield the title compound as a white powder (25 mg, 27%) mp: > 250 °C (decomp.).

δ_H ^{1}H NMR (400 MHz, DMSO-d$_6$): 1.16 (d, *J* 6.9, 3H), 1.89 (app q, 2H), 2.67-2.84 (m, 3H), 4.08 (app t, 2H), 6.42 (br s, 2H, NH_2), 6.74 (d, *J* 2.2, 1H), 7.13-7.18 (m, 3H), 7.22-7.26 (m, 2H), 8.99 (app t, 2H, NH_2), 10.95 (s, 1H,NH), 11.23 (s, 1H, NH)

δ_C ^{13}C NMR (100 MHz, DMSO-d$_6$): 22.5, 33.8, 36.9, 42.4, 44.6, 98.9 ,109.2, 118.6, 126.7, 127.2, 128.9, 146.0, 147.9, 152.7, 159.7

ν_{max} (film)/cm^{-1}: 1623, 1671, 2902, 2972, 3271, 3676

HRMS (*m/z* –ESI): Found: 310.1667 ($[M^+ – 2H]^-$, $C_{17}H_{20}N_5O$; Requires: 310.1673)

(*R*)-*N*-((2-amino-4-oxo-4,7-dihydro-3*H*-pyrrolo[2,3-*d*]pyrimidin-5-yl)methyl)-3-hydroxy-3-phenylpropan-1-aminium chloride (53)

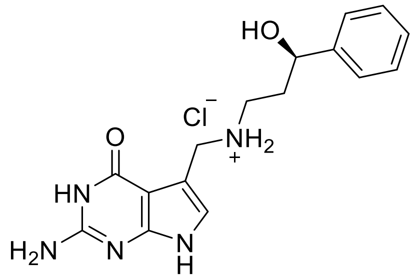

Prepared as per general procedure **D** yielding the title compound as a white powder (58 mg, 60%) mp: > 250 °C (decomp.).

δ_H ^{1}H NMR (400 MHz, DMSO-d$_6$):	1.82-1.97 (m, 2H), 2.96-2.97 (m, 2H), 4.12 (app t, 2H), 4.64 (dd, *J* 4.3, 8.1, 1H), 6.38 (br s, 2H NH$_2$), 6.77 (d, *J* 2.2, 1H), 7.18-7.31 (m, 5H), 8.93 (br s, 2H, NH$_2$), 10.86 (br s, 1H, NH), 11.25 (br s, 1H, NH)
δ_C ^{13}C NMR (100 MHz, DMSO-d$_6$):	35.5, 42.4, 43.7, 70.2, 99.0, 109.3, 118.9, 125.0, 127.4, 128.5, 145.5, 146.5, 156.2, 159.3
ν_{max} (film)/cm^{-1}:	1623, 1675, 2901, 2988, 2972, 3341, 3676
HRMS (*m/z* –APCI):	Found: 314.1621 (M$^+$; $C_{16}H_{20}N_5O_2$; Requires 314.1612)

(*S*)-*N*-((2-amino-4-oxo-4,7-dihydro-3*H*-pyrrolo[2,3-*d*]pyrimidin-5-yl)methyl)-3-hydroxy-3-phenylpropan-1-aminium chloride (54)

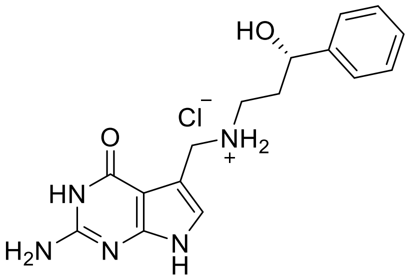

Prepared as per general procedure **D** yielding the title compound as a white powder (34 mg, 54%) mp: > 250 °C (decomp.).

δ_H ^{1}H NMR (400 MHz, DMSO-d$_6$):	1.86-1.97 (m, 2H), 2.92-2.97 (m, 2H), 4.14 (app t, 2H), 4.64-4.67 (m, 1H), 6.53 (br s, 2H, NH$_2$), 6.80 (d, *J* 2.1, 1H), 7.19-7.26 (m, 1H), 7.28-7.33 (m, 4H), 9.00 (br s, 2H, NH$_2$), 10.99 (br s, 1H NH), 11.23 (br s, 1H, NH)
δ_C ^{13}C NMR (100 MHz, DMSO-d$_6$):	35.5, 42.5, 43.7, 70.2, 99.0, 109.2, 118.8, 126.0, 127.4, 128.5, 145.5, 147.3, 152.7, 159.5
ν_{max} (film)/cm^{-1}:	1624, 1671, 2902, 2972, 3400 (broad), 3676
HRMS (*m/z* –ESI):	Found: 312.1466 ([M$^+$ – 2H]$^-$, $C_{16}H_{18}N_5O_2$; Requires: 312.1466)

***N*-((2-amino-4-oxo-4,7-dihydro-3*H*-pyrrolo[2,3-*d*]pyrimidin-5-yl)methyl)-3,3-diphenylpropan-1-aminium chloride (55)**

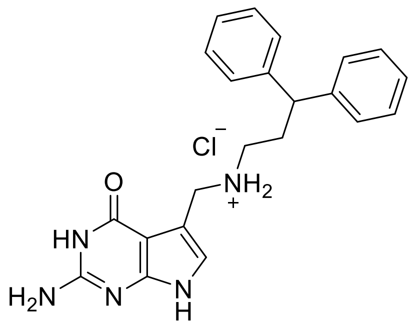

Prepared as per general procedure **D** to yield the title compound as a white powder (57 mg, 50%), mp: > 250 °C (decomp.).

δ_H ^{1}H NMR (400 MHz, DMSO-d$_6$):	2.37 (app q, 2H), 2.74 (m, 2H), 4.03 (t, *J* 8.0, 1H), 4.11 (t, *J* 5.1, 2H), 6.65 (br s, 2H, NH_2), 6.77 (d, *J* 1.8, 1H), 7.11-7.16 (m, 2H), 7.24-7.25 (m, 8H), 9.19 (br s, 2H, NH_2), 11.13 (br s, 1H, NH), 11.31 (d, *J* 1.8, 1H, NH)
δ_C ^{13}C NMR (100 MHz, DMSO-d$_6$):	35.5, 42.1, 43.0, 55.3, 98.7, 108.7, 118.7, 126.7, 128.6, 128.8, 146.2, 149.0, 152.8, 159.9
ν_{max} (film)/cm^{-1}:	1130, 1615, 1685, 2445, 2973, 3313
HRMS (*m/z* -ESI):	Found: 374.1963 (M^+; $C_{22}H_{24}N_5O$; Requires: 374.1975)

***N*-((2-Amino-4-oxo-4,7-dihydro-3*H*-pyrrolo[2,3-*d*]pyrimidin-5-yl)methyl)-3,3,3-triphenylpropan-1-aminium chloride (56)**

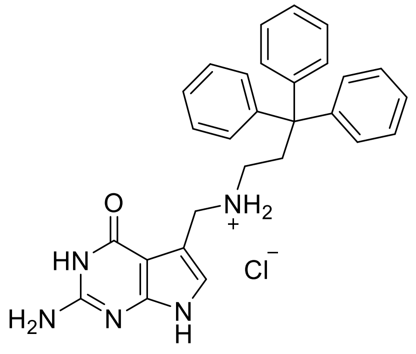

Prepared as per general procedure **D** to yield the title compound as a white powder (100 mg, 79%), mp: > 250 °C (decomp.).

δ_H ^{1}H NMR (400 MHz, DMSO-d$_6$):	2.88-2.9 (m, 2H), 4.11 (t, *J* 4.4, 2H), 6.40 (br s, 2H,NH_2), 6.70 (d, *J* 2.1, 1H), 7.13-7.18 (m, 9H), 7.24 (m, 6H,) , 9.08 (br s, 2H, NH_2), 10.93 (br s, 1H, NH), 11.20 (br s, 1H, NH)

δ_C ^{13}C NMR (100 MHz, DMSO-d$_6$): 35.5, 42.1, 43.0, 55.3, 98.7, 108.7, 118.7, 126.7, 128.6, 128.8, 146.2, 149.0, 152.8, 159.9

ν_{max} (film)/cm^{-1}: 1156, 1594, 1676, 1692, 2780, 2923, 3130

HRMS (*m/z* -ESI): Found: 448.2155 ([M$^+$ – 2H]$^-$, $C_{28}H_{26}N_5O$; Requires: 448.2143)

***N*-((2-amino-4-oxo-4,7-dihydro-3*H*-pyrrolo[2,3-*d*]pyrimidin-5-yl)methyl)-3-ethyl-3-phenylpentan-1-aminium chloride (57)**

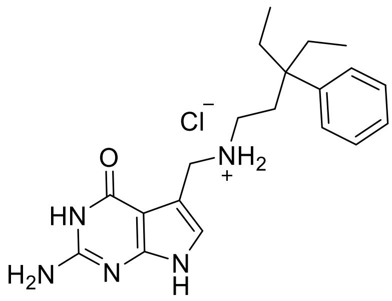

Prepared as per general procedure **D** to yield the title compound as a white powder (53 mg, 65%), mp: > 250 °C (decomp.).

δ_H ^{1}H NMR (400 MHz, DMSO-d$_6$): 0.56 (t, *J* 7.1, 6H), 1.58 (q, *J* 7.1, 4H), 1.93-1.97 (m, 2H), 2.55-2.61 (m, 2H), 4.09 (t, *J* 4.5, 2H), 6.45 (br s, 2H, NH$_2$), 6.74 (d, *J* 2.1, 1H), 7.12 (t, *J* 6.9, 1H), 7.22-7.28 (m, 4H), 9.02 (br s, 2H, NH$_2$), 10.98 (s, 1H), 11.23 (s, 1H, NH)

δ_C ^{13}C NMR (100 MHz, DMSO-d$_6$): 8.13, 29.1, 31.7, 42.1, 42.3, 42.8, 98.7, 109.2, 118.5, 126.2, 126.7, 128.6, 145.6, 148.2, 152.8, 159.8

ν_{max} (film)/cm^{-1}: 1598, 1677, 2902, 2972, 2988, 3676

HRMS (*m/z* -ESI): Found: 354.2285 (M$^+$; $C_{20}H_{28}N_5O$; Requires: 354.2288)

***N*-((2-amino-4-oxo-4,7-dihydro-3*H*-pyrrolo[2,3-*d*]pyrimidin-5-yl)methyl)-2-(2-phenyl-1,3-dithiolan-2-yl)ethan-1-aminium chloride (58)**

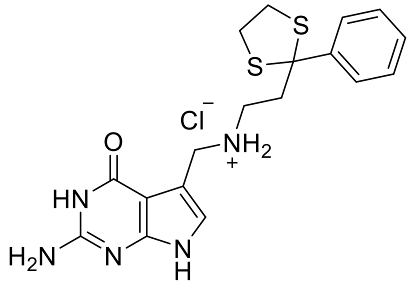

Prepared as per general procedure **D** to yield the title compound as a white powder (55 mg, 24%), mp: > 250 °C (decomp.).

δ_H ^1H NMR (400 MHz, DMSO-d$_6$):	2.62-2.67 (m, 2H), 2.84-2.86 (m, 2H), 3.39-3.44 (m, 4H), 4.13 (app s, 2H), 6.30 (br s, 2H, NH_2), 6.74 (app s, 1H), 7.24 (m, 1H), 7.31 (t, *J* 7.9, 2H), 7.55-7.57 (m, 2H), 8.91 (br s, 2H, NH_2), 10.81 (br s, 1H, NH), 11.21 (br s, 1H, NH)
δ_C ^{13}C NMR (100 MHz, DMSO-d$_6$):	0.6, 40.7, 42.2, 44.0, 71.1, 98.8, 108.8, 118.7, 127.0, 127.9, 128.7, 143.4, 148.2, 152.9, 159.7
ν_{max} (film)/cm^{-1}:	1598, 1676, 2758, NH stretches missing
HRMS (*m/z* -APCI):	Found: 388.1252 (M^+; $C_{18}H_{22}N_5OS_2$; Requires: 388.1260)

***N*-((2-amino-4-oxo-4,7-dihydro-3*H*-pyrrolo[2,3-*d*]pyrimidin-5-yl)methyl)-2-(2-phenyl-1,3-dioxolan-2-yl)ethan-1-aminium chloride (59)**

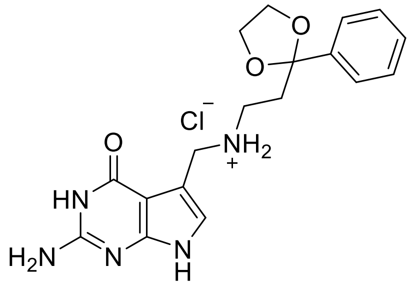

Prepared as per general procedure **D** to yield the title compound as a white powder (6 mg), mp: > 250 °C (decomp.).

δ_H ^1H NMR (400 MHz, DMSO-d$_6$):	2.17 (t, *J* 6.9, 2H), 2.95 (t *J* 6.9, 2H), 3.62-3.65 (m, 2H), 3.96-3.99 (m, 2H), 4.11 (app s, 2H), 6.27 (br s, 2H, NH_2), 6.75 (app s, 1H), 7.28-7.35 (m, 5H), 8.72 (br s, 2H,NH_2), 10.81 (br s, 1H, NH), 11.21 (br s, 1H, NH)

δ_C ^{13}C NMR (100 MHz, DMSO-d_6):	35.7, 41.5, 43.2, 64.3, 98.1, 108.1, 117.5, 125.2, 125.5, 128.3, 128.3, 141.3, 152.2, 152.7, 160.2
ν_{max} (film)/cm^{-1}:	1596, 1678, 2901, 2988, 3341, 3676

***N*-((2-amino-4-oxo-4,7-dihydro-3*H*-pyrrolo[2,3-*d*]pyrimidin-5-yl)methyl)-3-(anthracen-9-yl)propan-1-aminium chloride (60)**

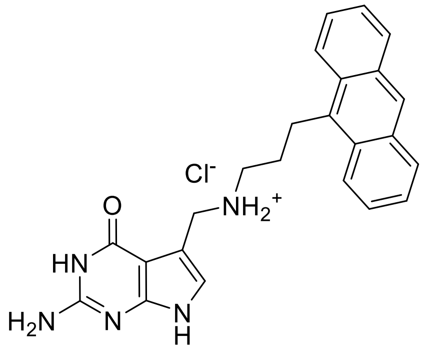

Prepared as per general procedure **D** to yield the title compound as a white powder (10 mg, 39%), mp > 250 °C (decomp.).

δ_H ^1H NMR (400 MHz, DMSO-d_6):	1.99-2.00 (m, 2H), 3.20-3.24 (m, 2H), 3.61-3.65 (m, 2H), 4.16 (t, *J* 4.8, 2H), 6.33 (br s, 2H, NH_2), 6.79 (app s, 1H), 7.50-7.53 (m, 4H), 8.07 (d, *J* 8.8, 2H), 8.32 (d, 2H), 8.47 (s, 1H), 9.05 (br m, 2H, NH_2), 10.88 (br s, 1H, NH), 11.26 (br s, 1H, NH)
δ_C ^{13}C NMR (100 MHz, DMSO-d_6):	23.5, 30.6, 42.3, 46.2, 98.9, 109.1, 118.6, 123.8, 125.1, 125.5, 126.0, 128.2, 130.5, 132.1, 136.8, 140.2, 152.7, 159.7
ν_{max} (film)/cm^{-1}:	1629, 1679, 1738, 2801, 2935, 3330 (broad)
HRMS (m/z -ES):	Found: 398.1979 (M^+; $C_{24}H_{24}N5O$; requires: 398.1975)
