# Supplementary material for: The human tRNA-guanine transglycosylase displays promiscuous nucleobase preference but strict tRNA specificity
Source: Nucleic Acids Res. 2021 May 1;49(9):4877–90. doi: 10.1093/nar/gkab289 (PMC8136771; doi:10.1093/nar/gkab289)
Supplement: gkab289_Supplemental_Files [file gkab289_supplemental_files.zip › 2k. Supplementary Fig. S1.pdf]

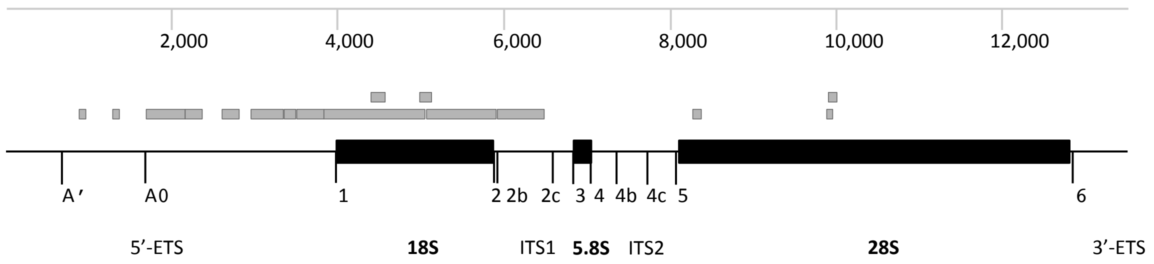

Supplementary Fig. S1. Mouse splenocyte reads mapping to pre-ribosomal RNA
Isolated and contiguous reads mapping to pre-ribosomal RNA from mouse primary splenocytes are shown in grey. The coding sequences for the mature 18S, 5.8S and 28S rRNAs are shown by the black bars which are positioned within non-coding spacers, the 5'- and 3'-external transcribed spacers (5'- and 3'-ETS) and the internal transcribed spacers 1 and 2 (ITS1 and ITS2). The positions of the known and predicted processing sites are shown (Mullineux and Lafontaine, 2012).

Mullineux, S.T. and Lafontaine, D.L., 2012. Mapping the cleavage sites on mammalian pre-rRNAs: where do we stand?. *Biochimie*, *94*(7), pp.1521-1532.
