# Supplementary figures and images for: The human tRNA-guanine transglycosylase displays promiscuous nucleobase preference but strict tRNA specificity
Source: Nucleic Acids Res. 2021 May 1;49(9):4877–90. doi: 10.1093/nar/gkab289 (PMC8136771; doi:10.1093/nar/gkab289)

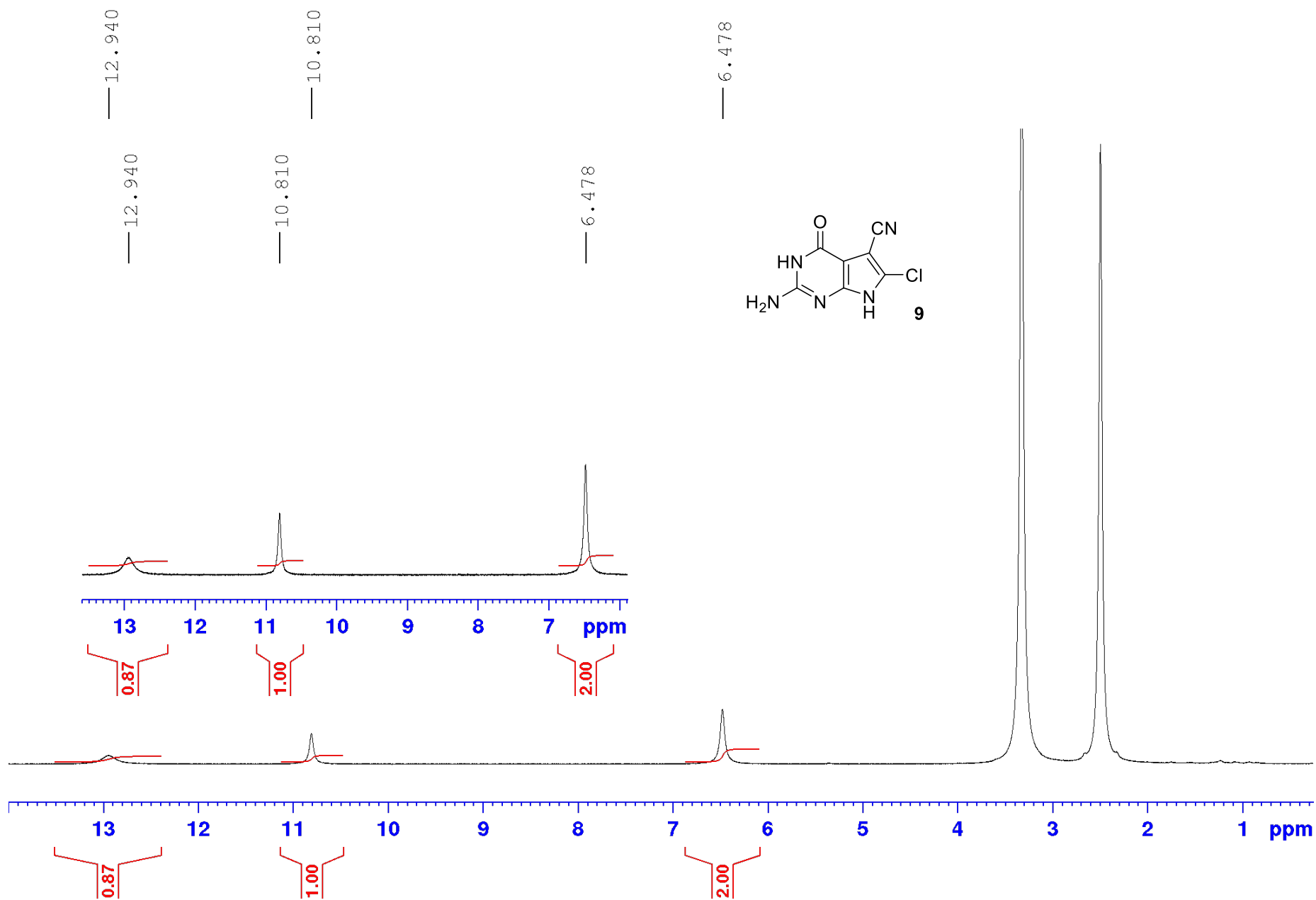

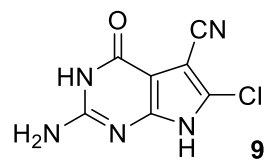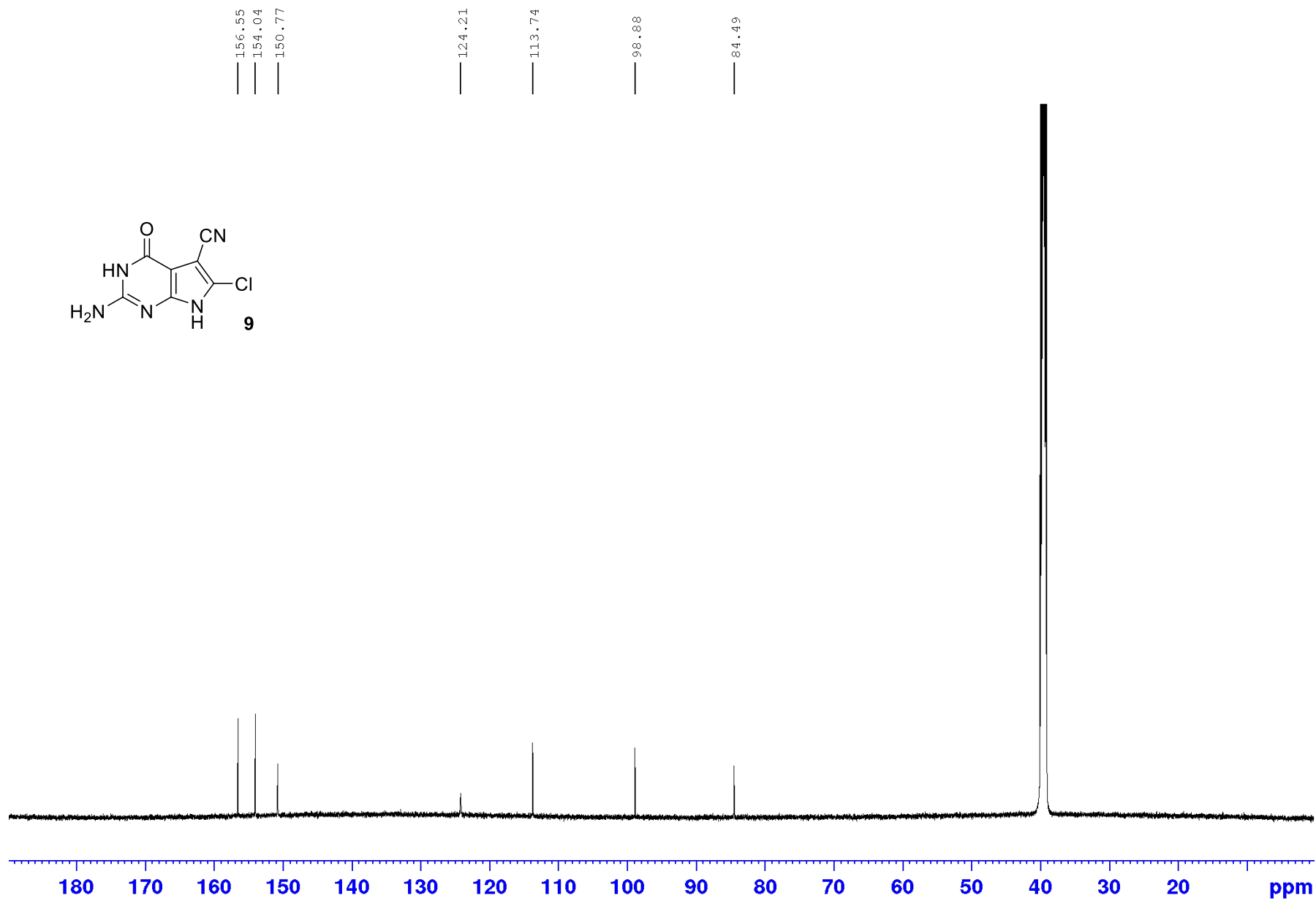

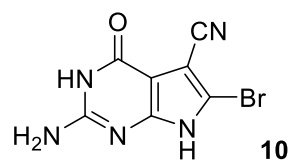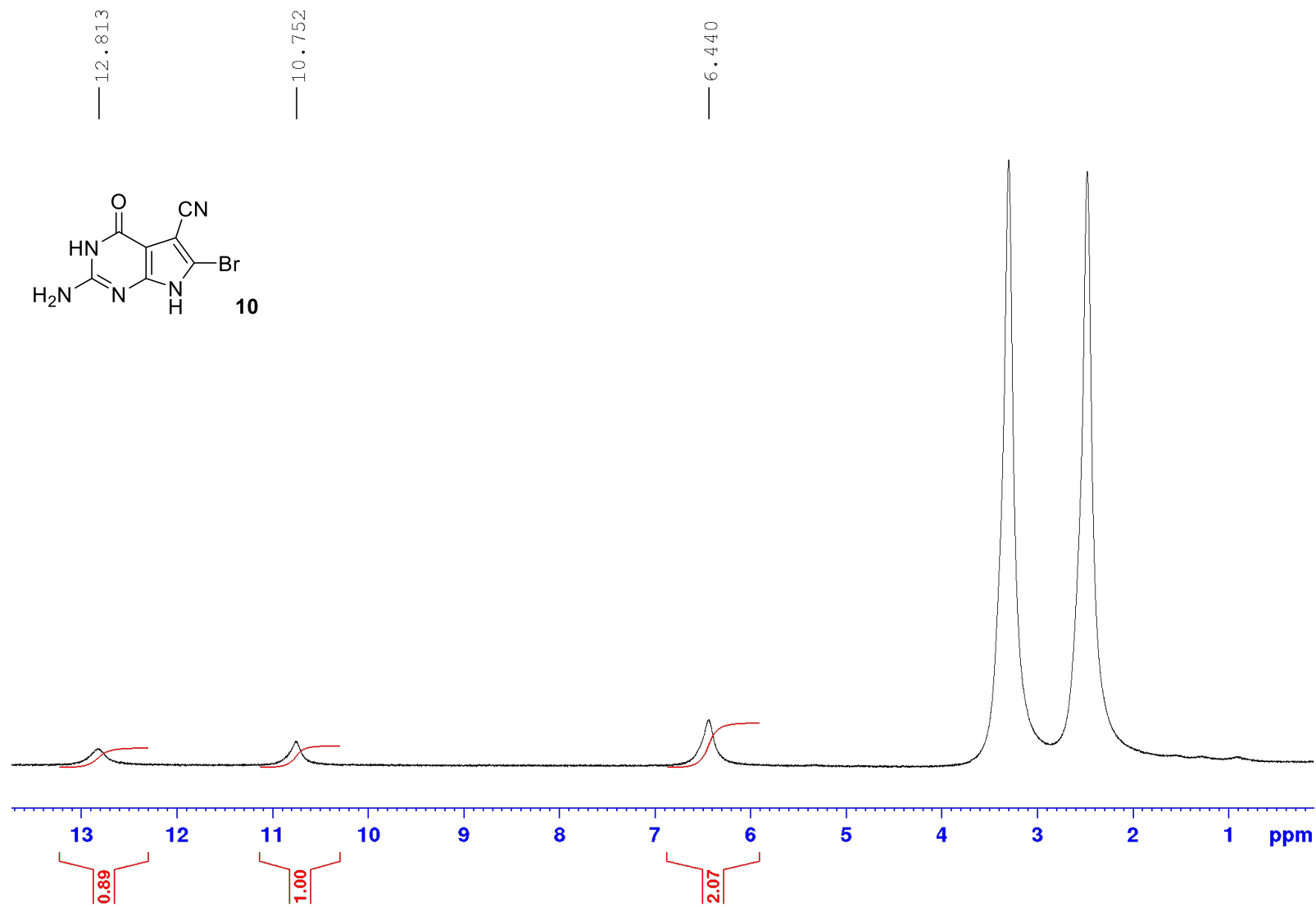

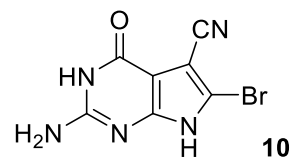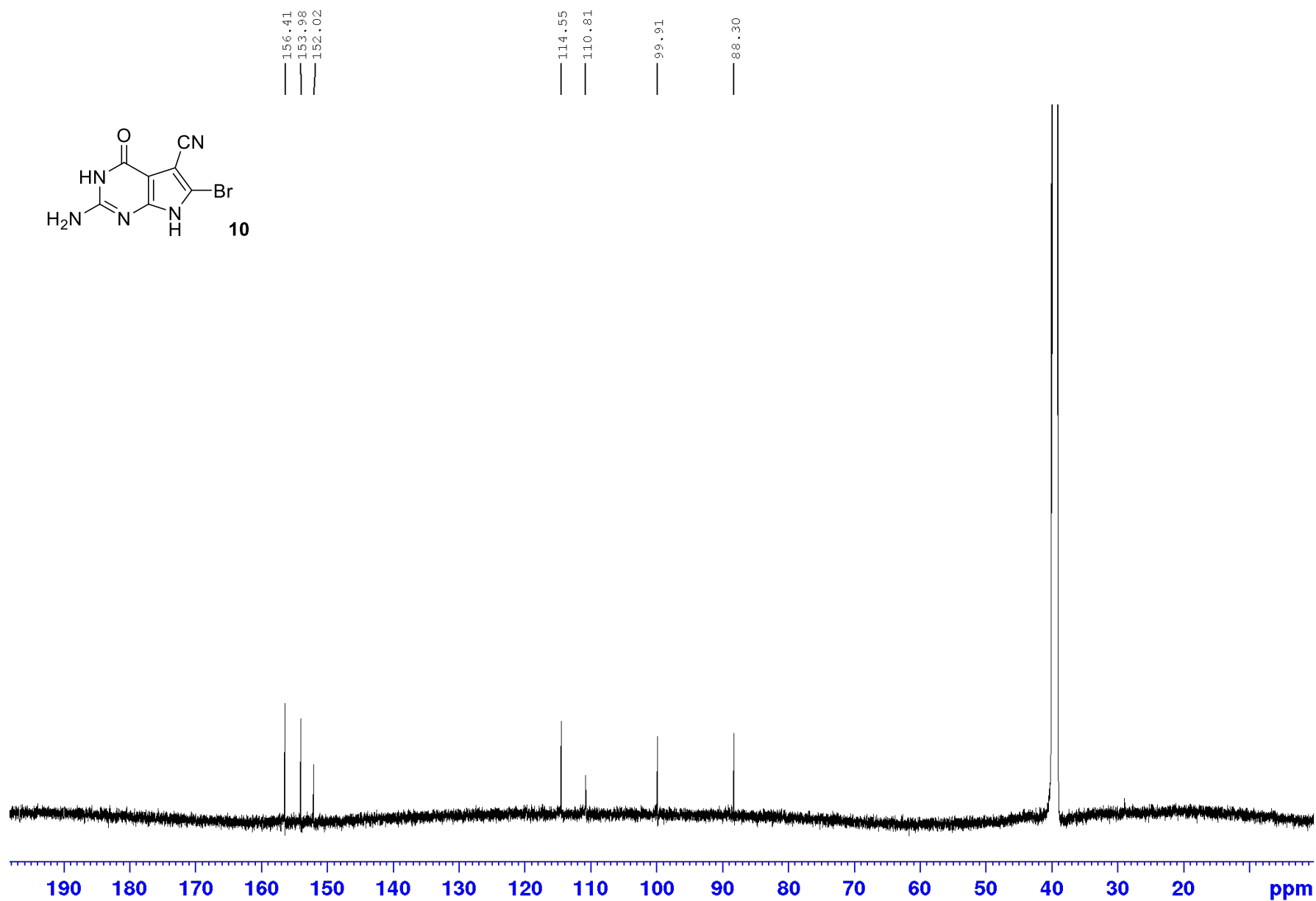

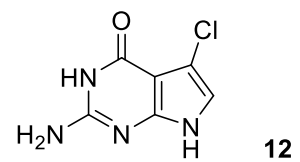

12

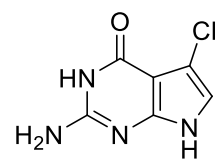

12

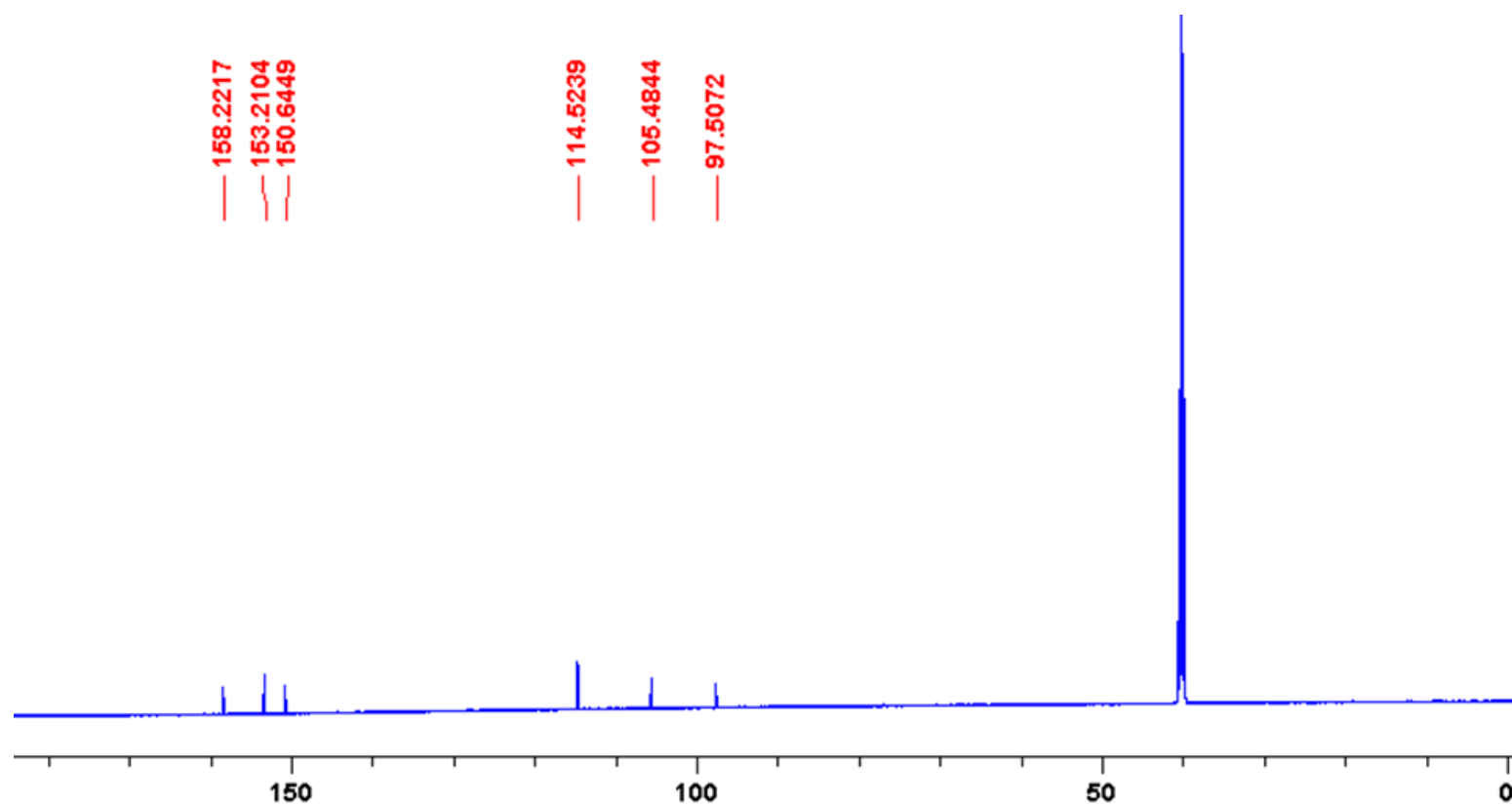

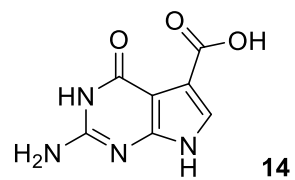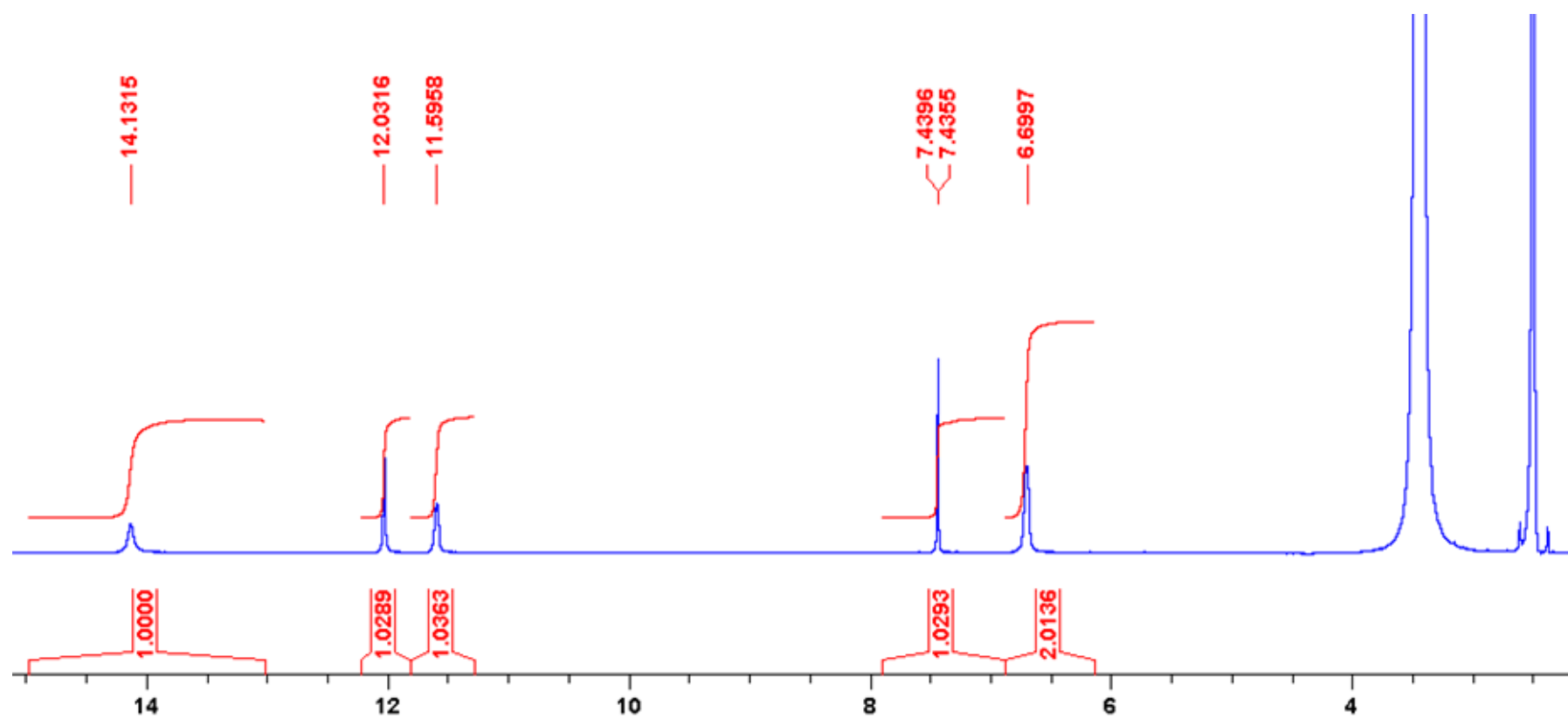

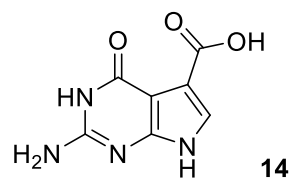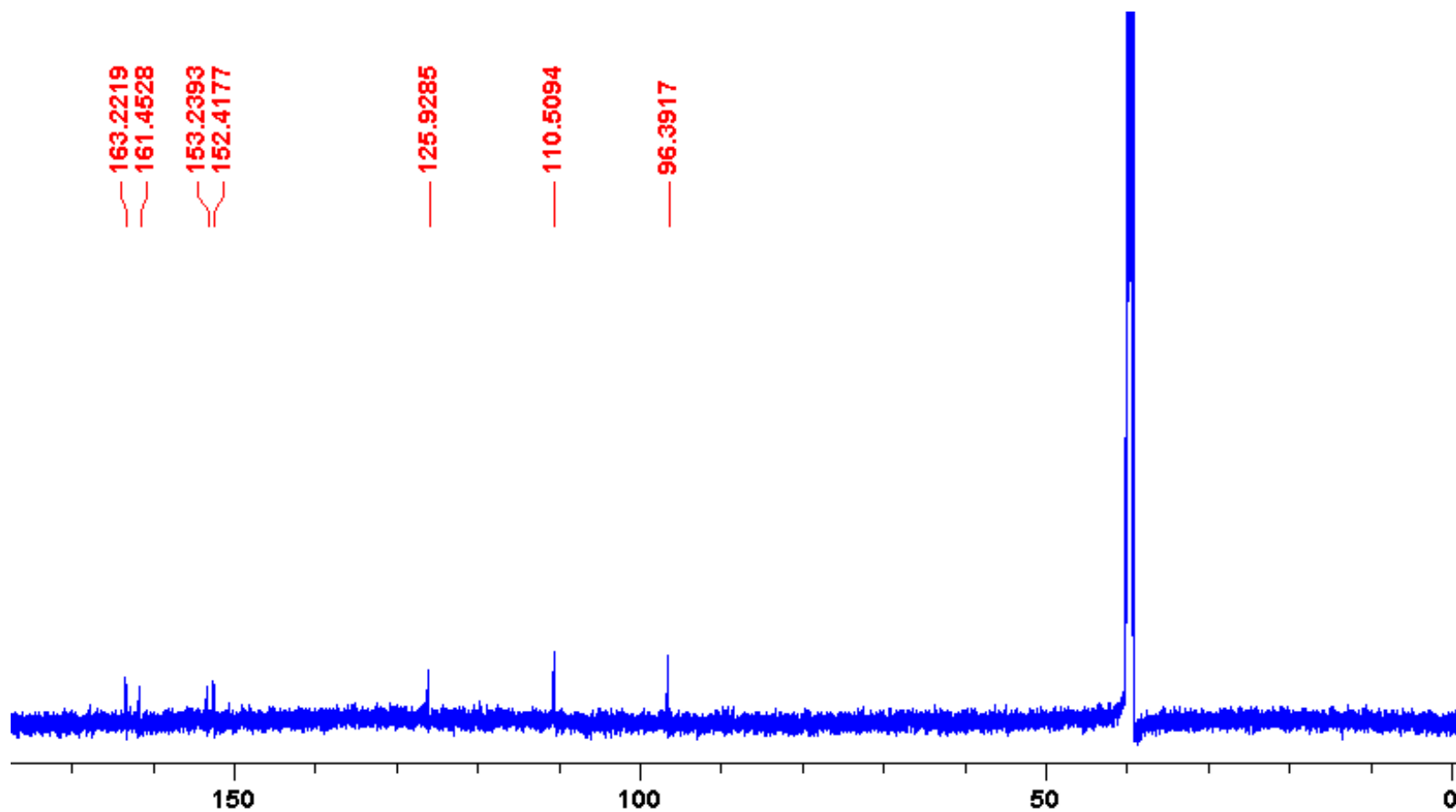

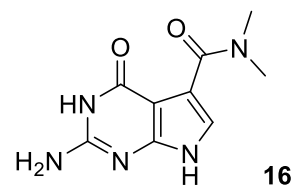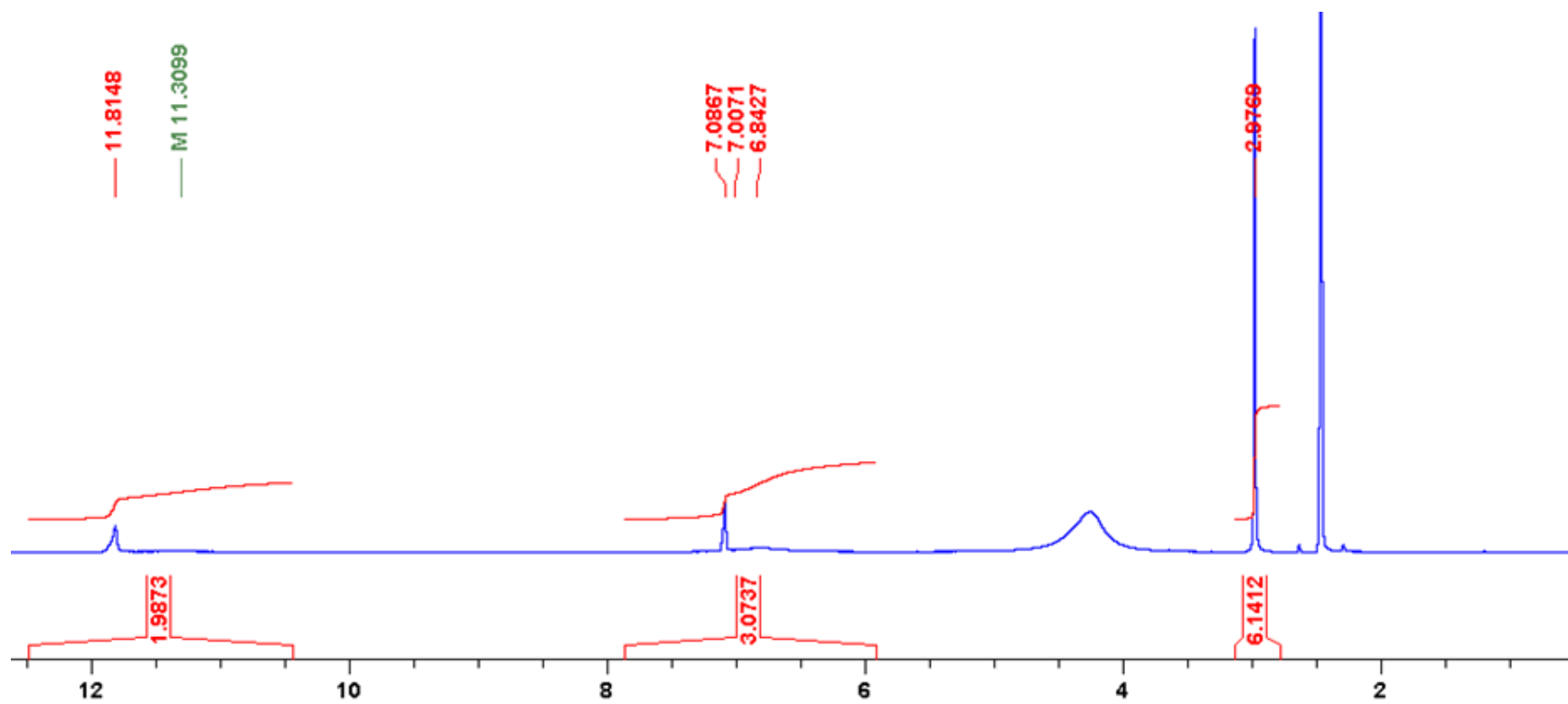

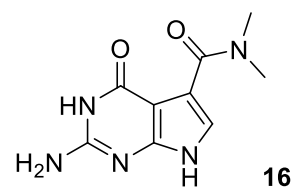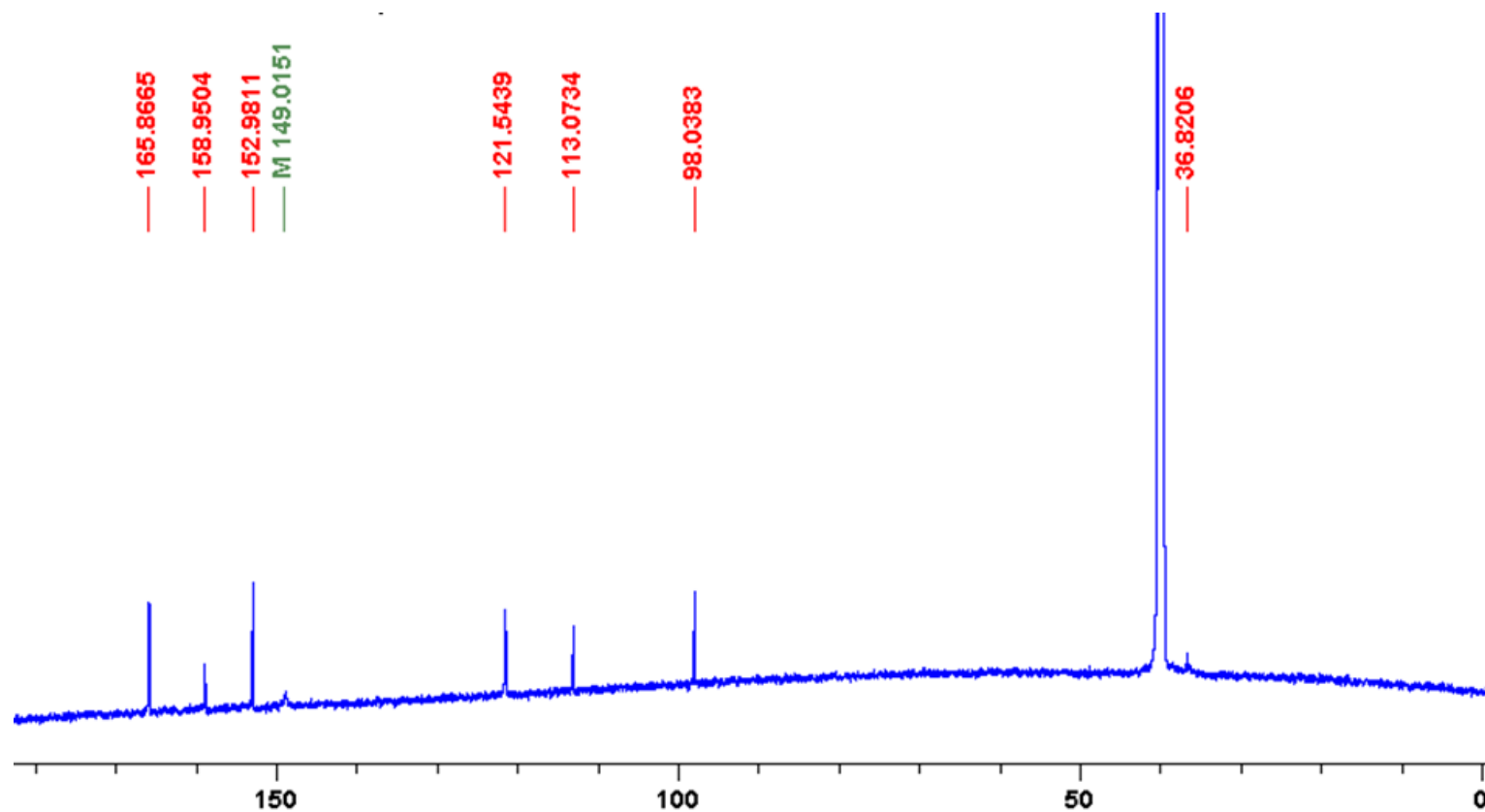

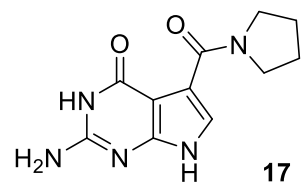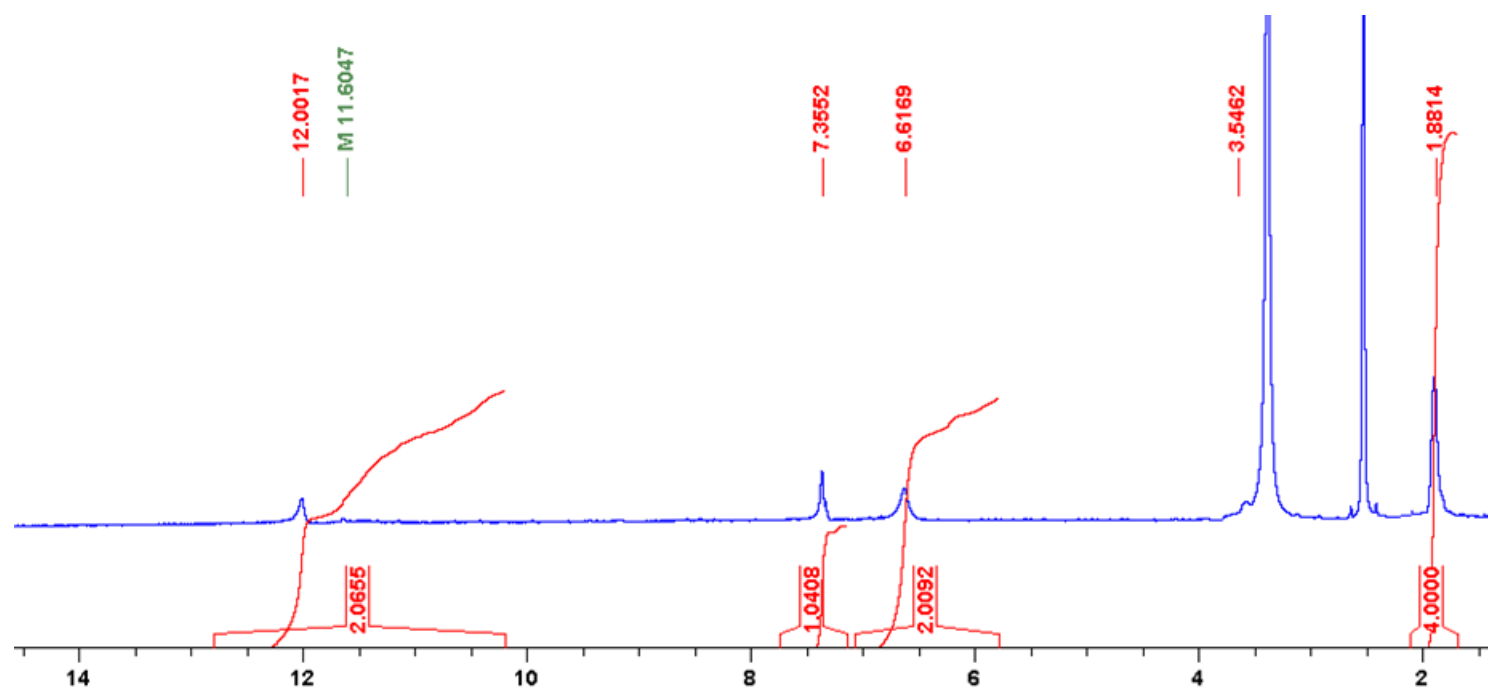

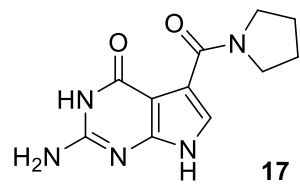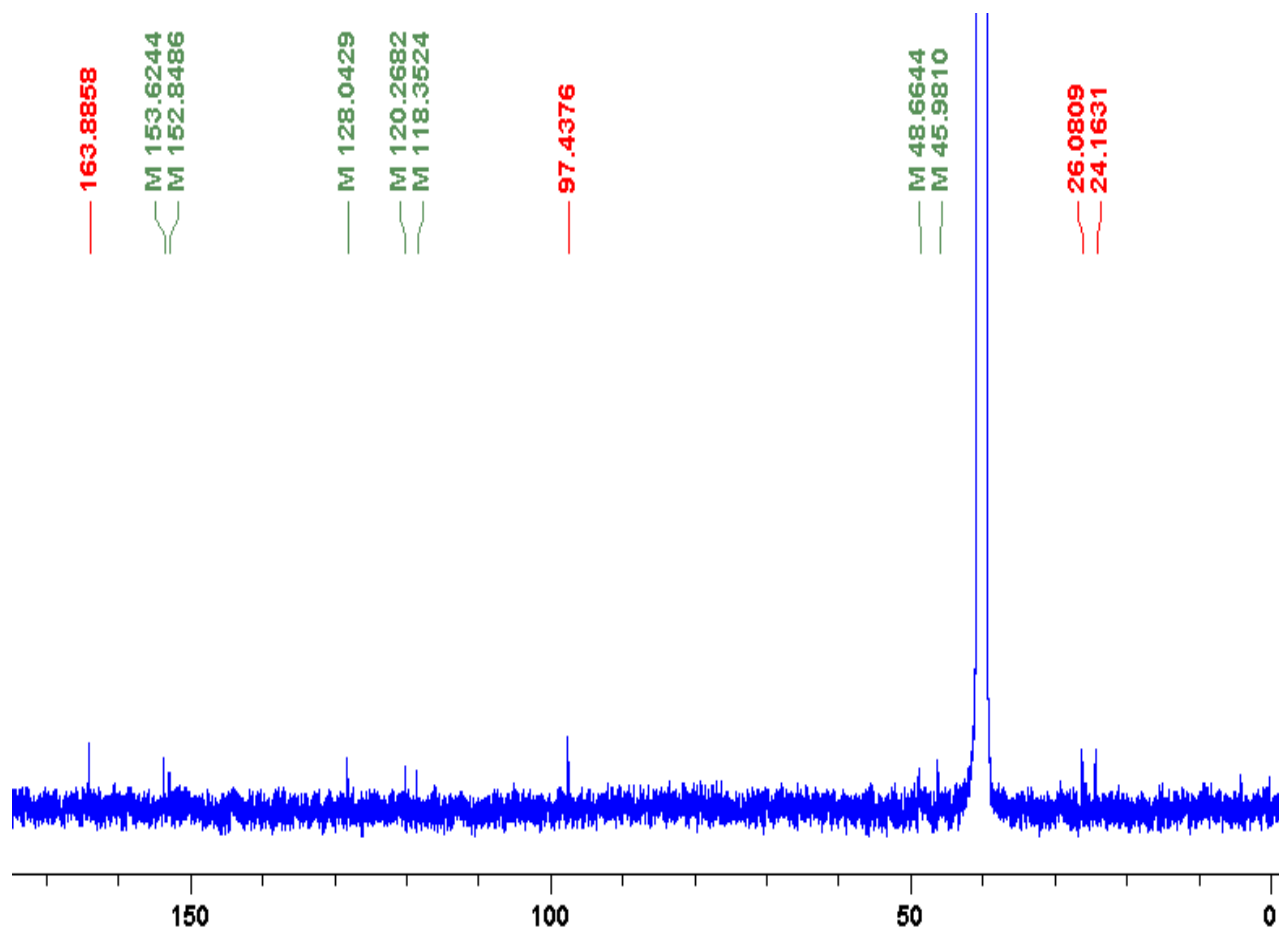

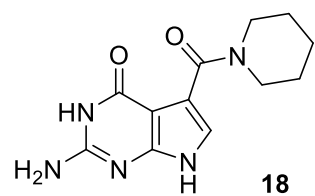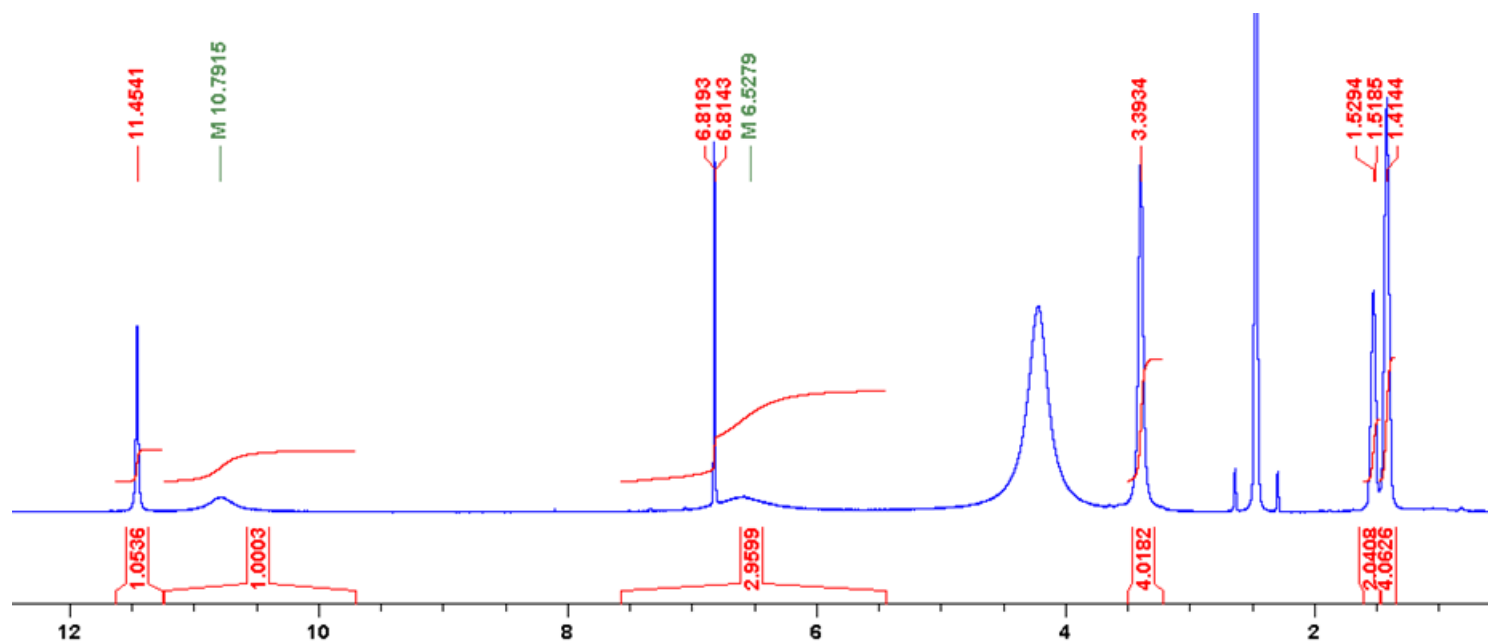

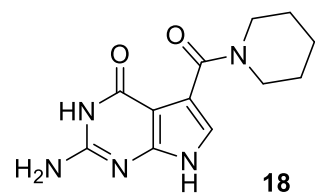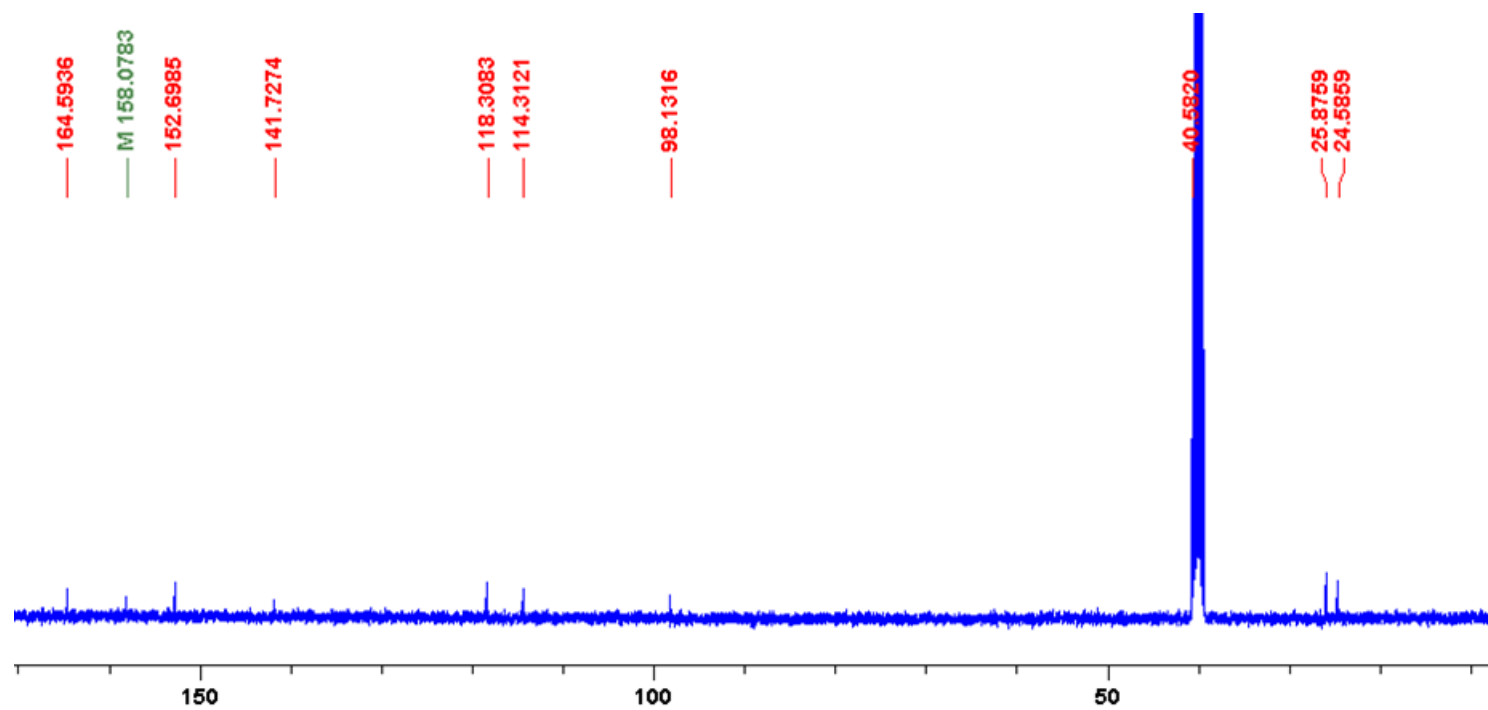

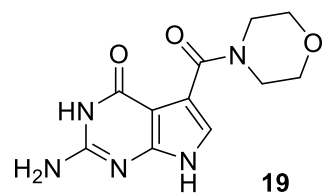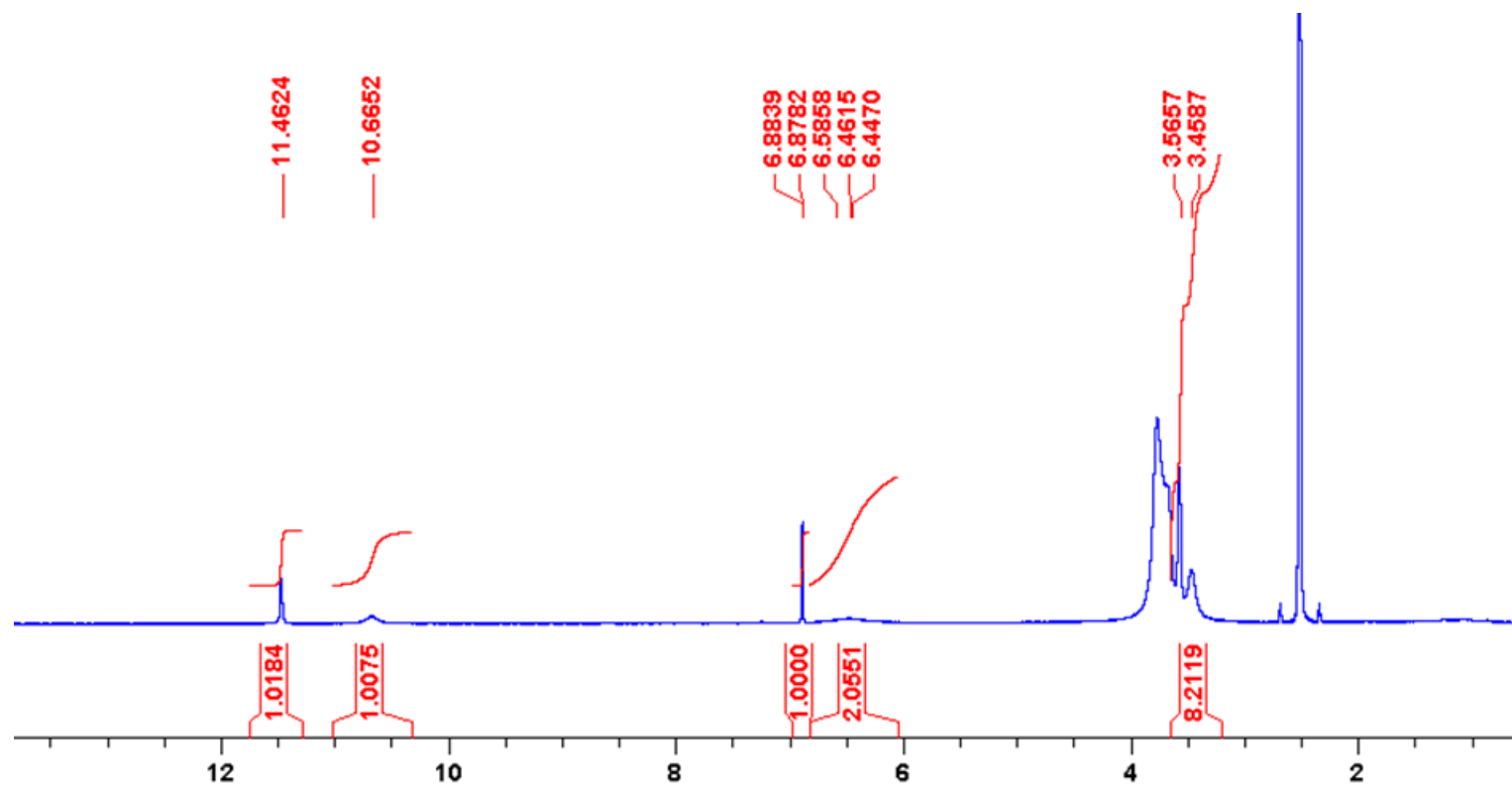

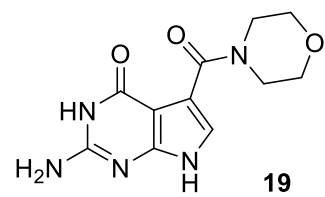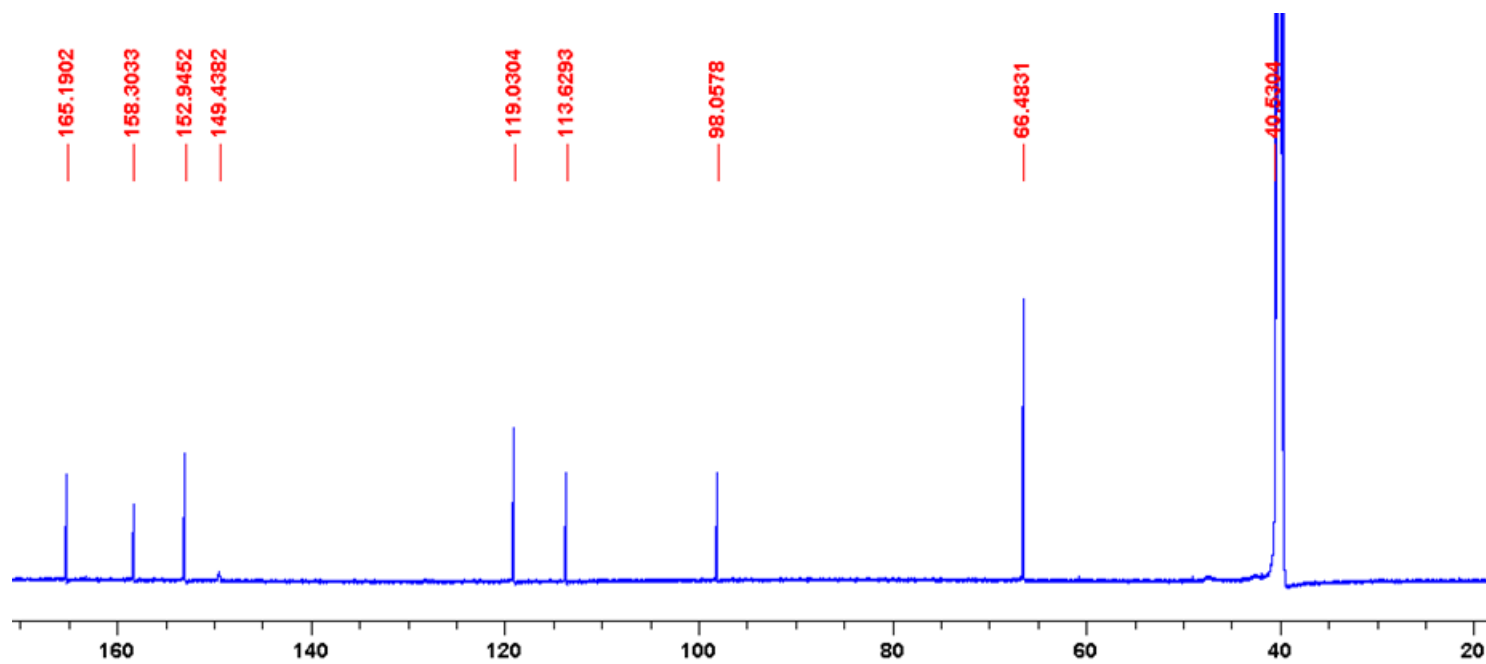

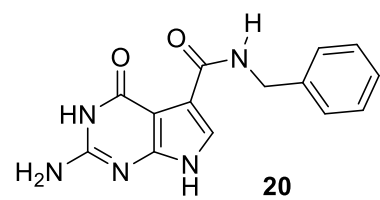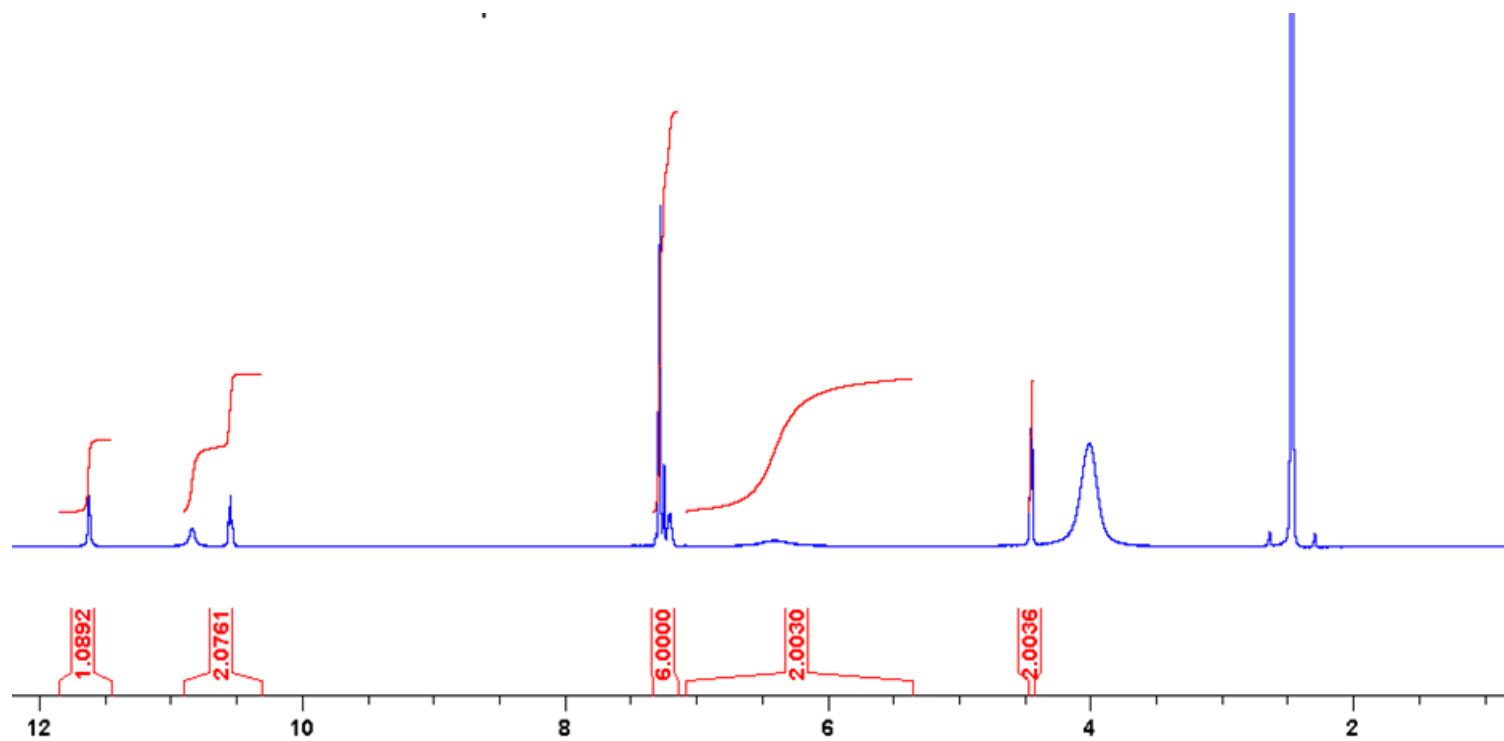

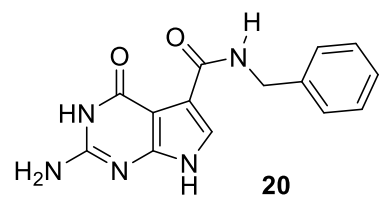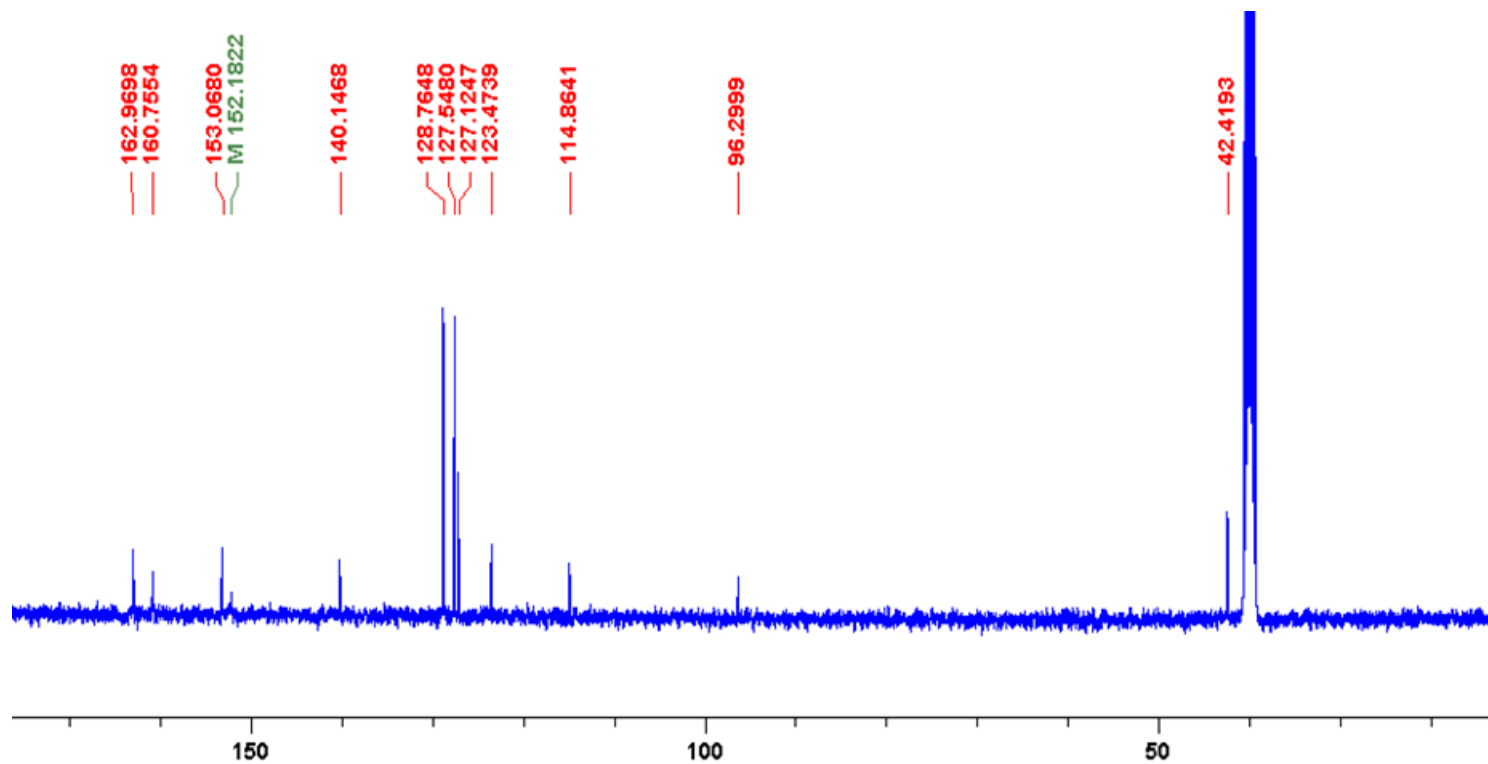

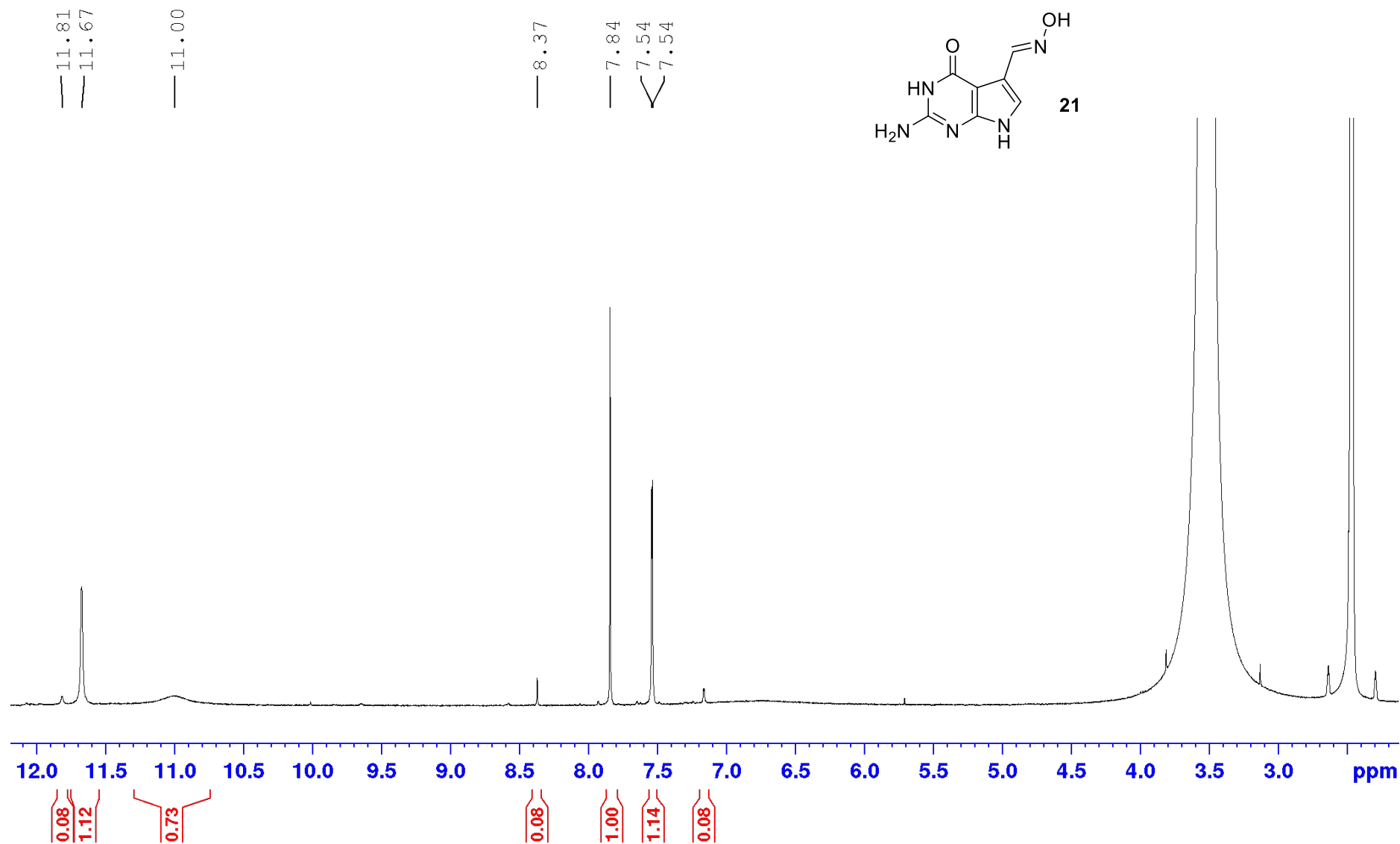

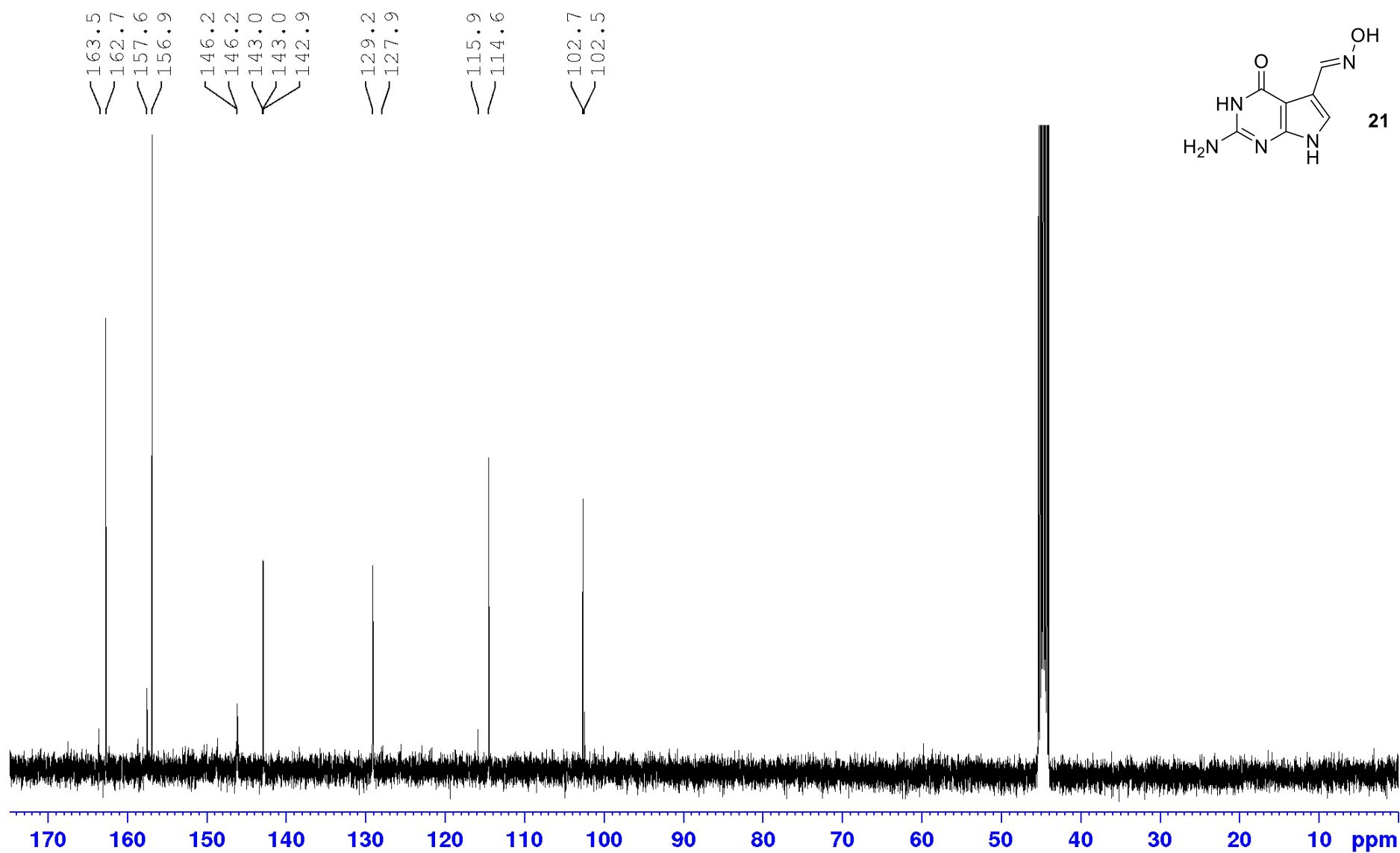

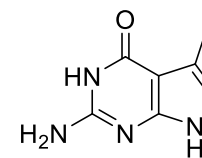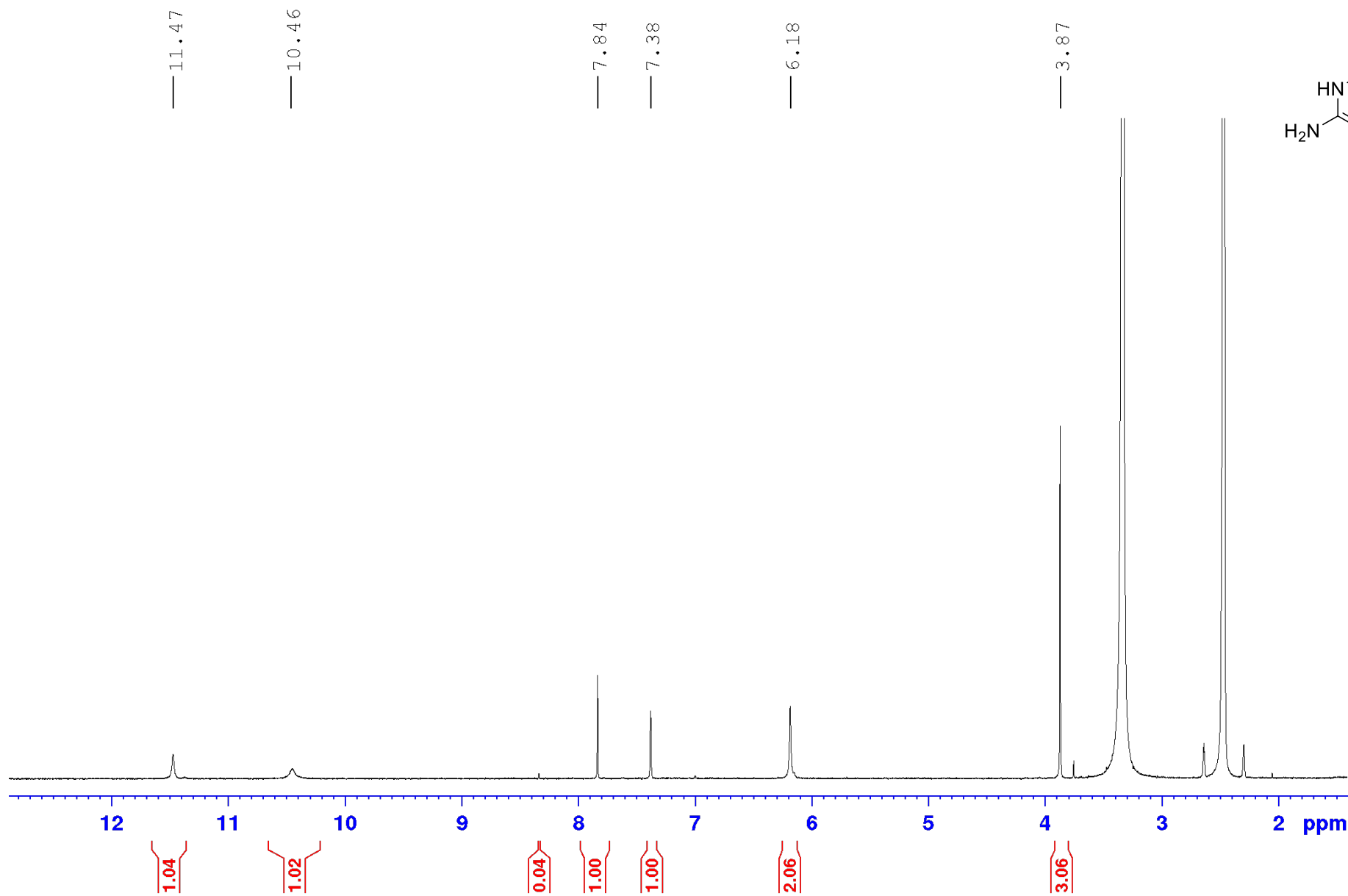

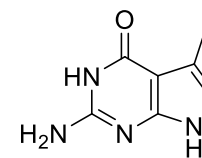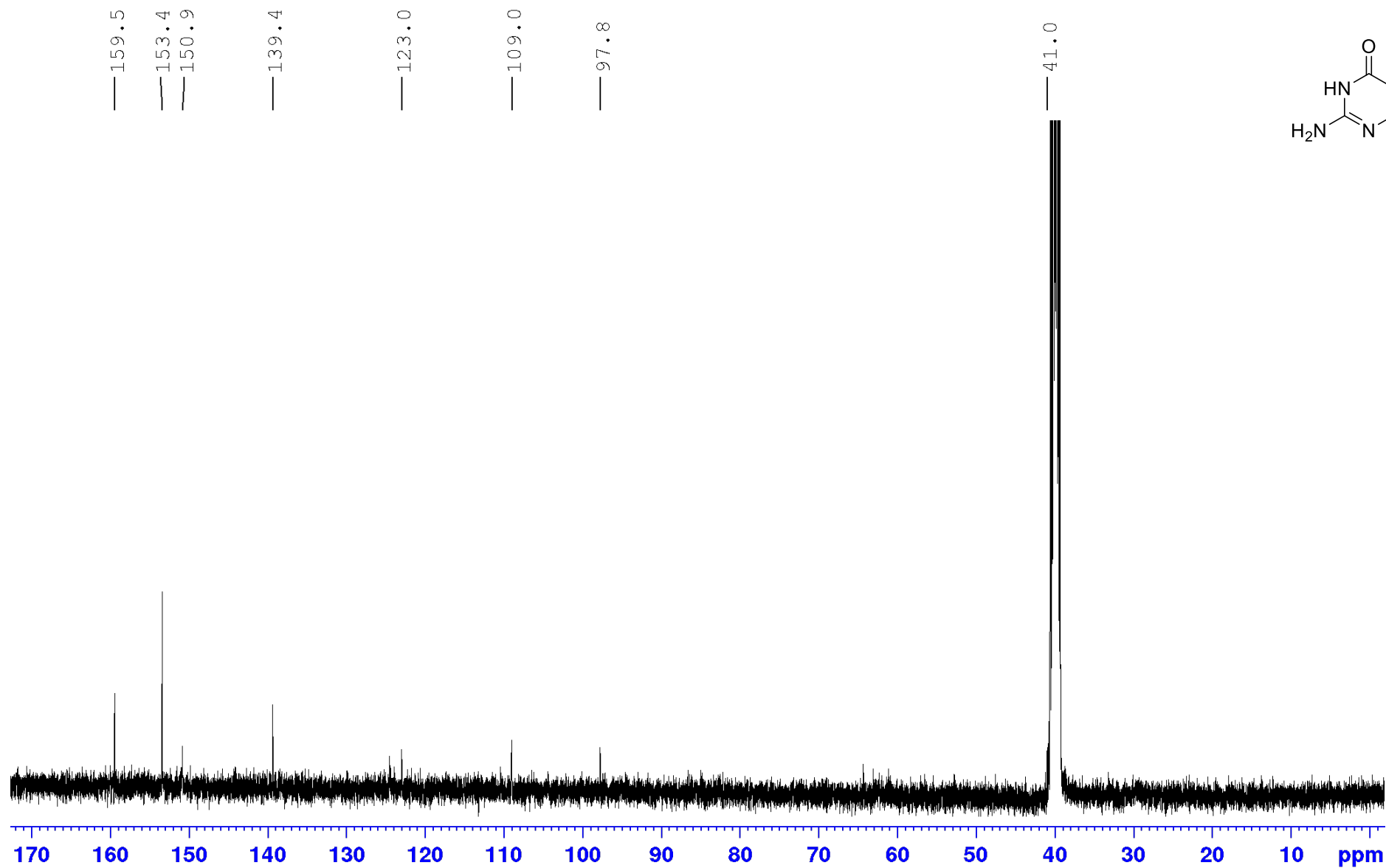

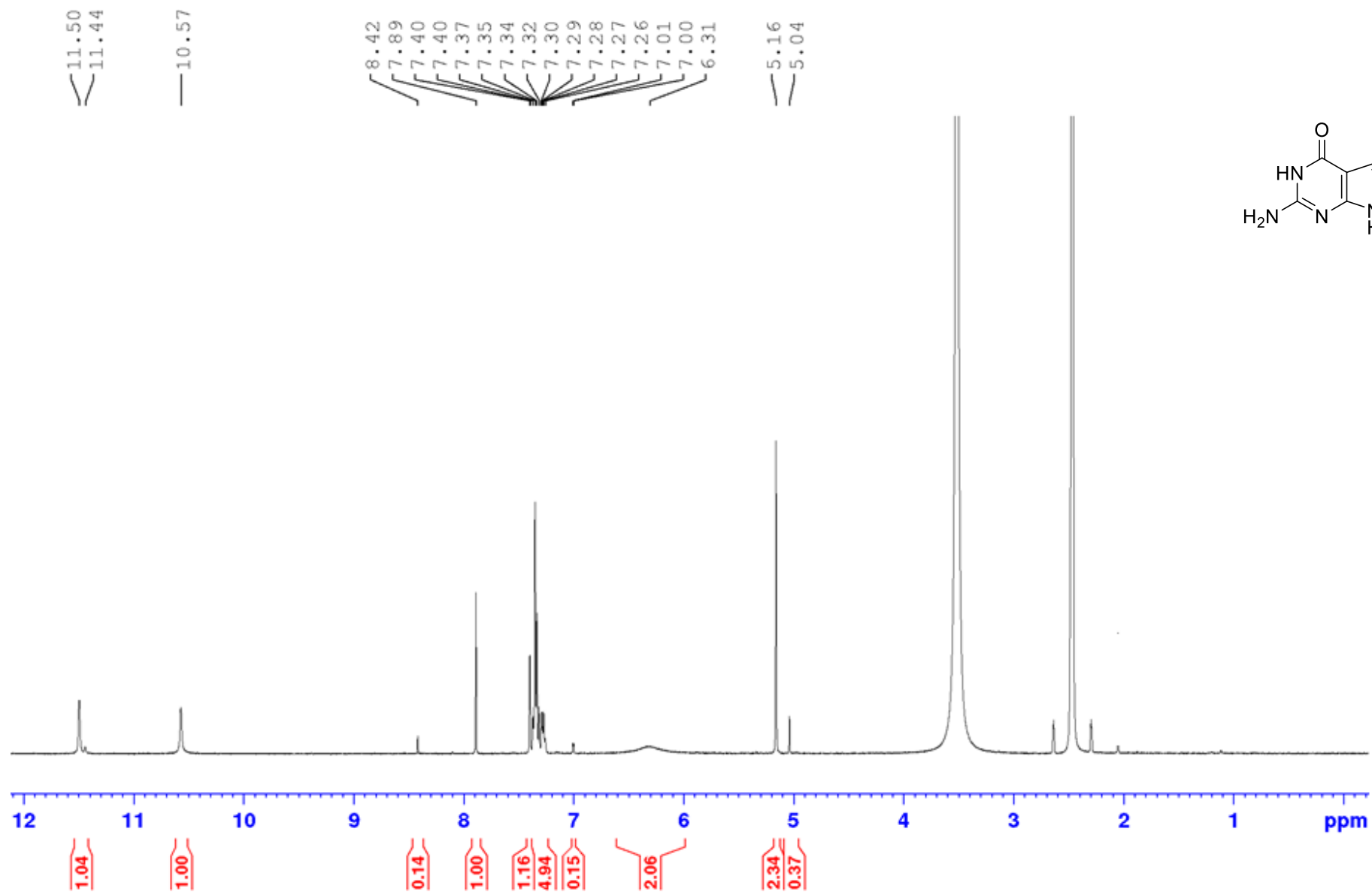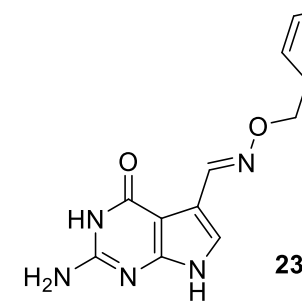

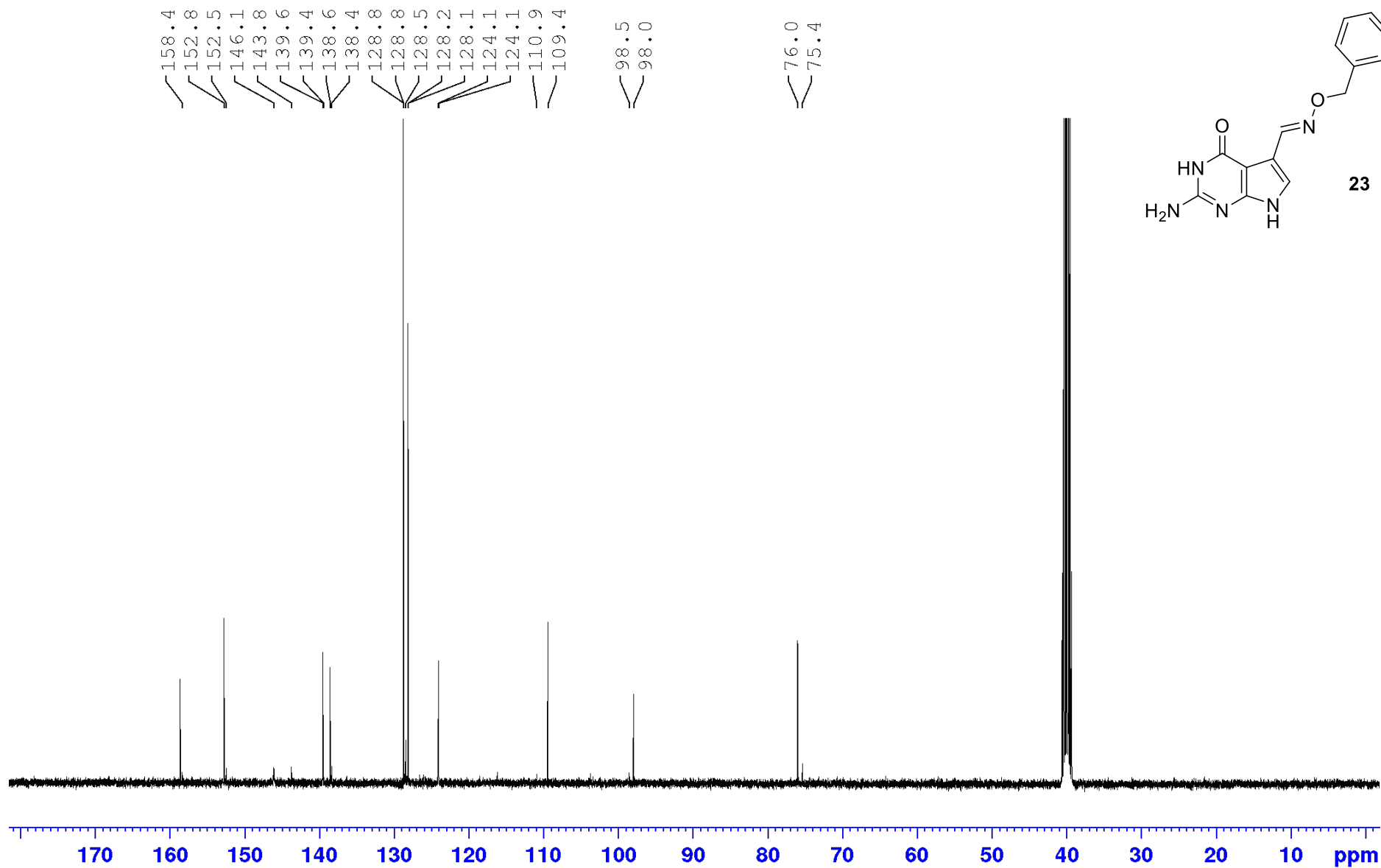

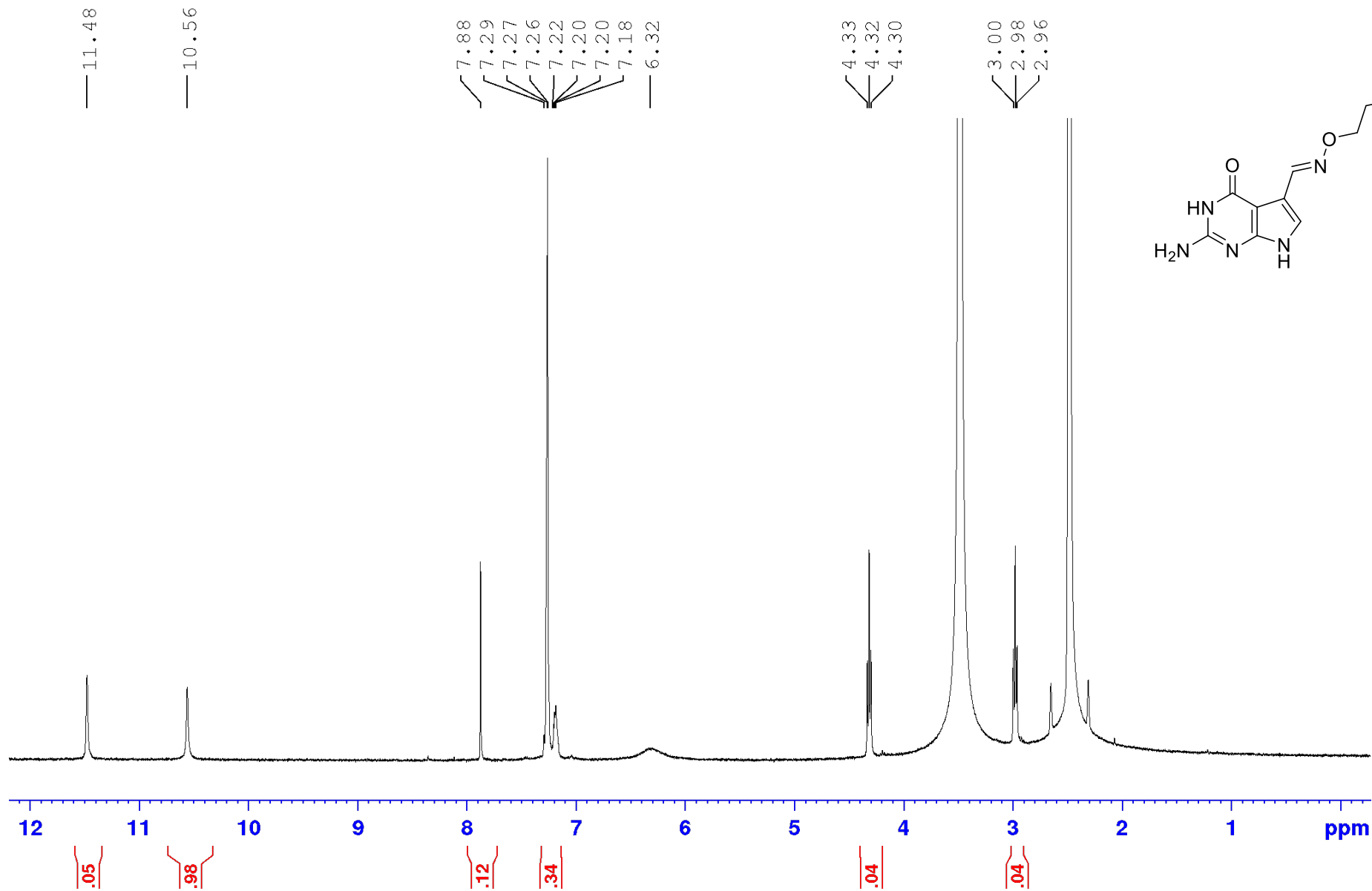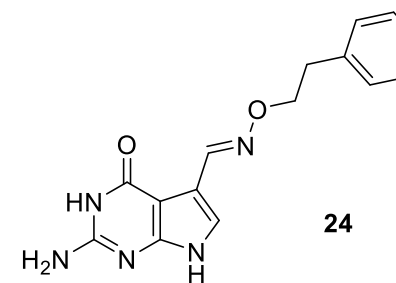

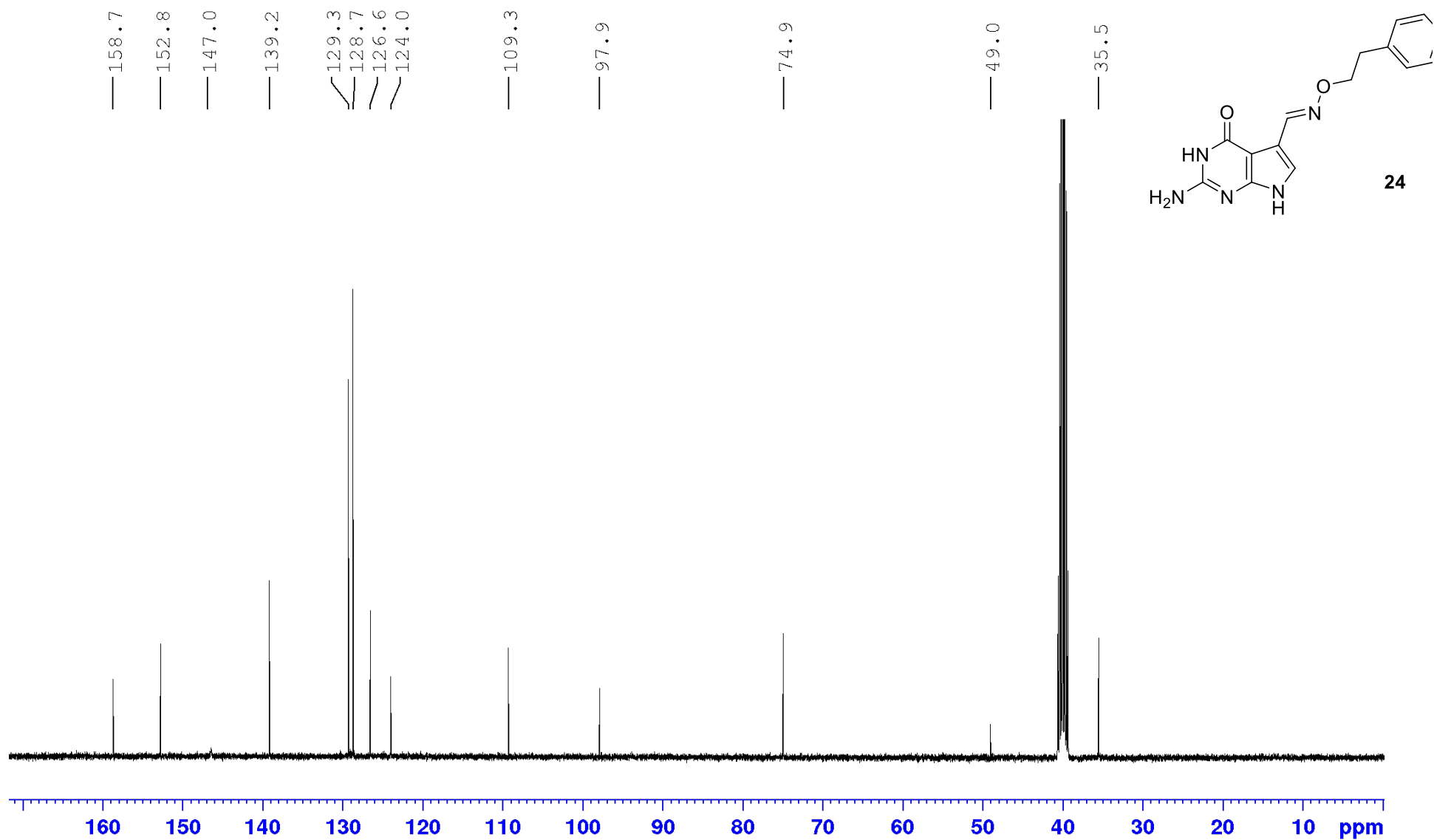

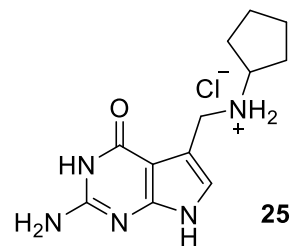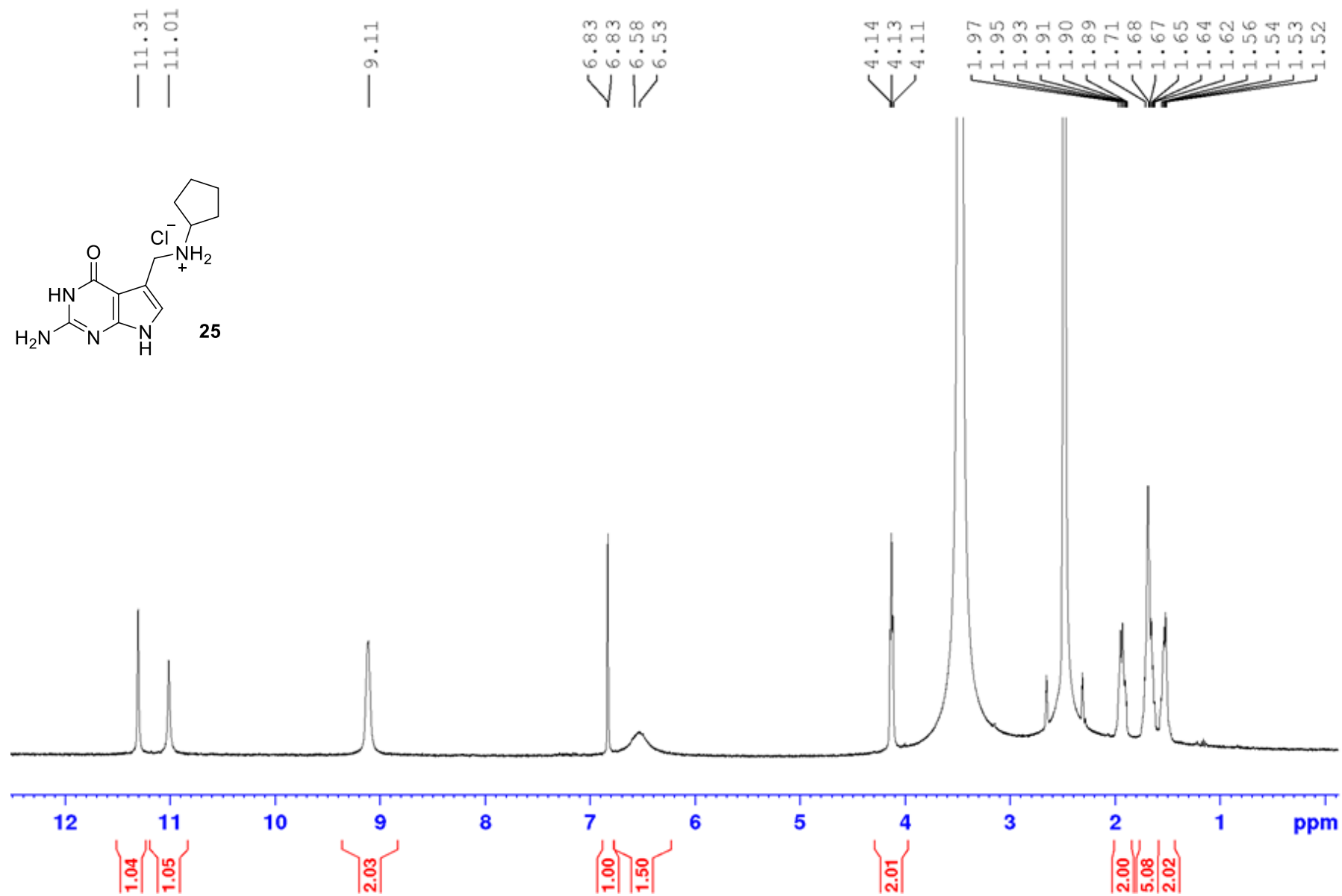

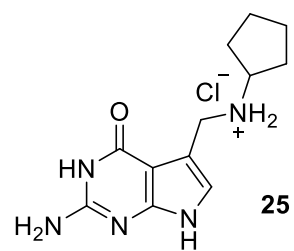

25

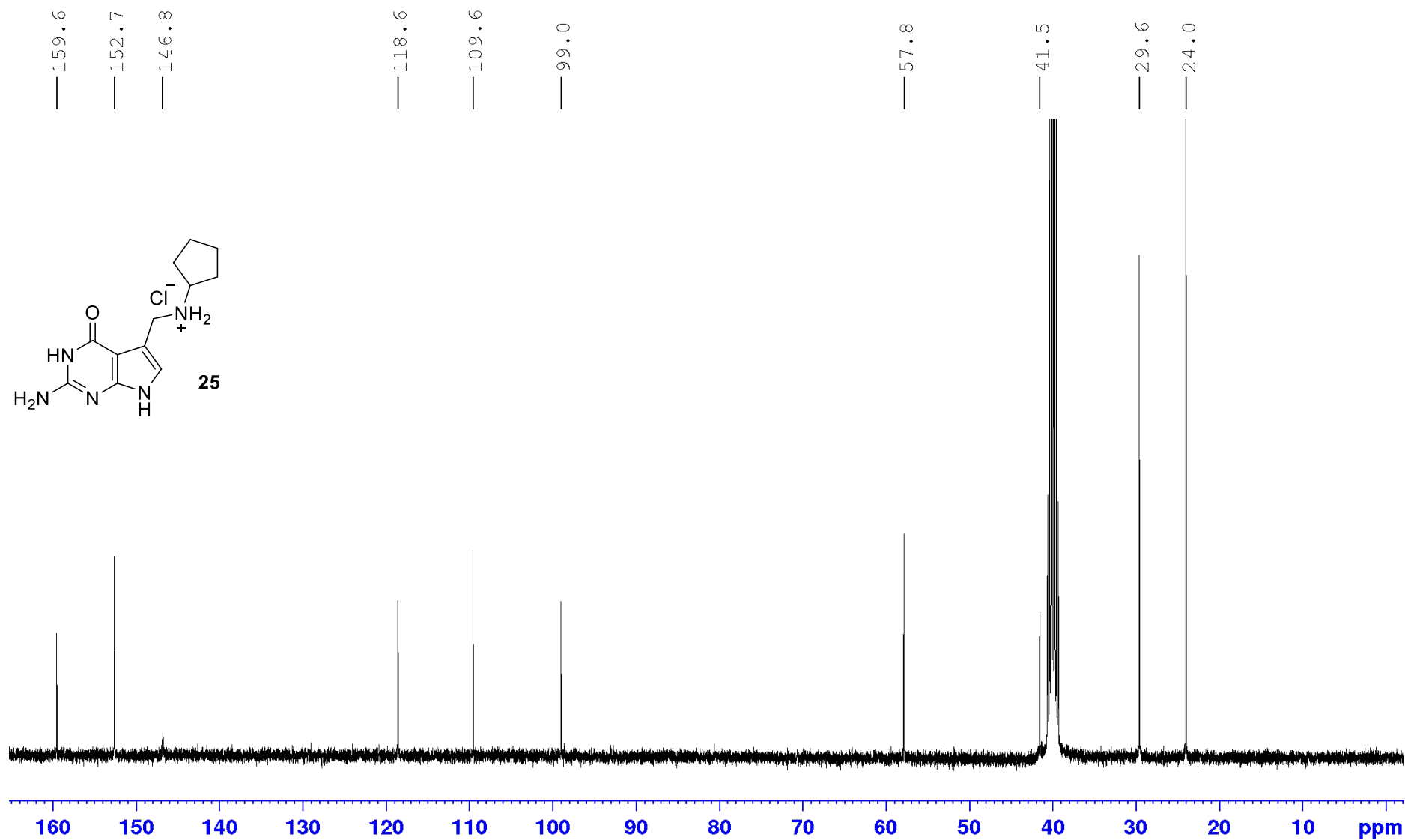

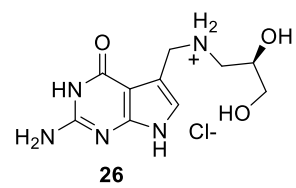

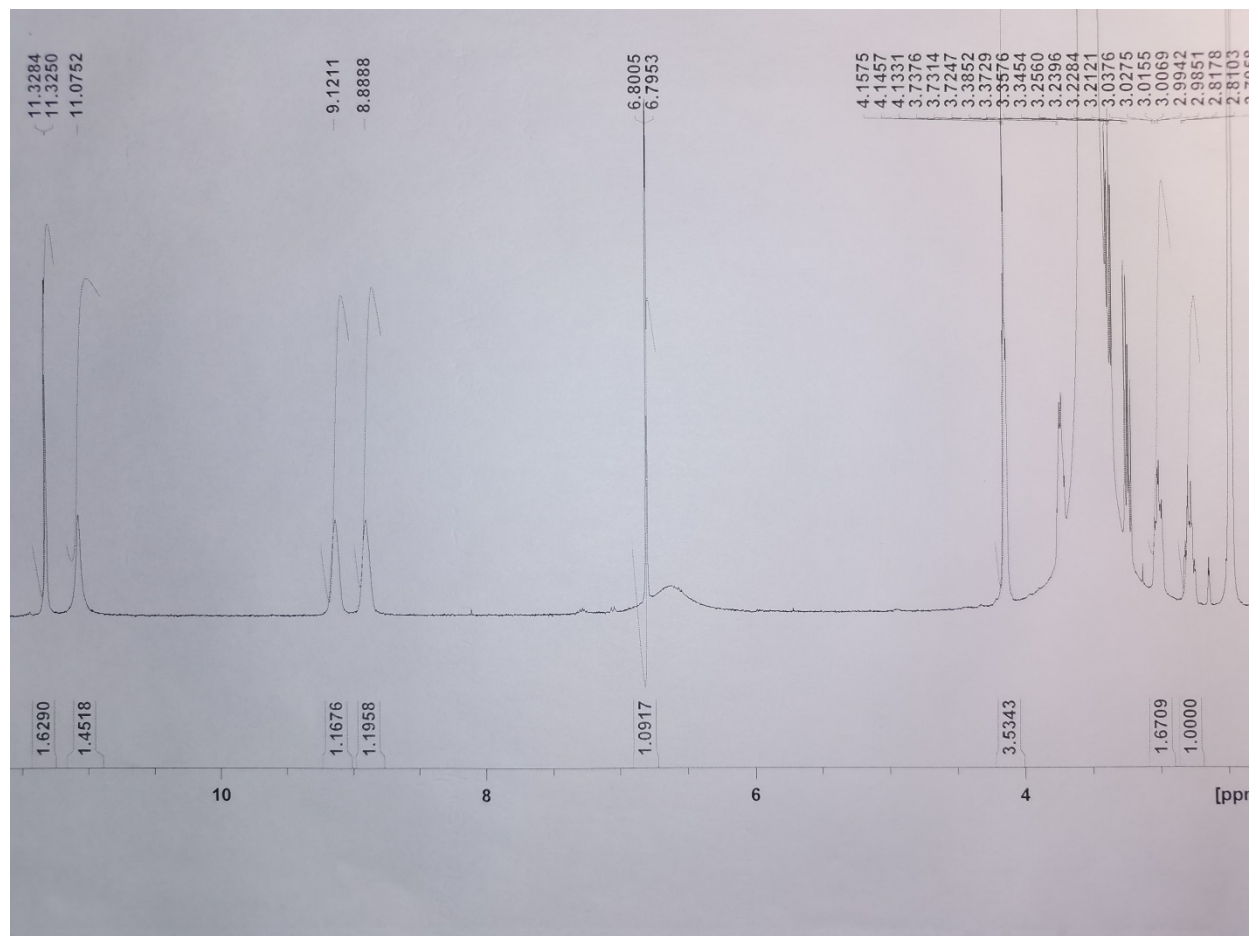

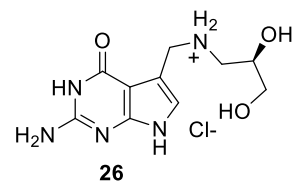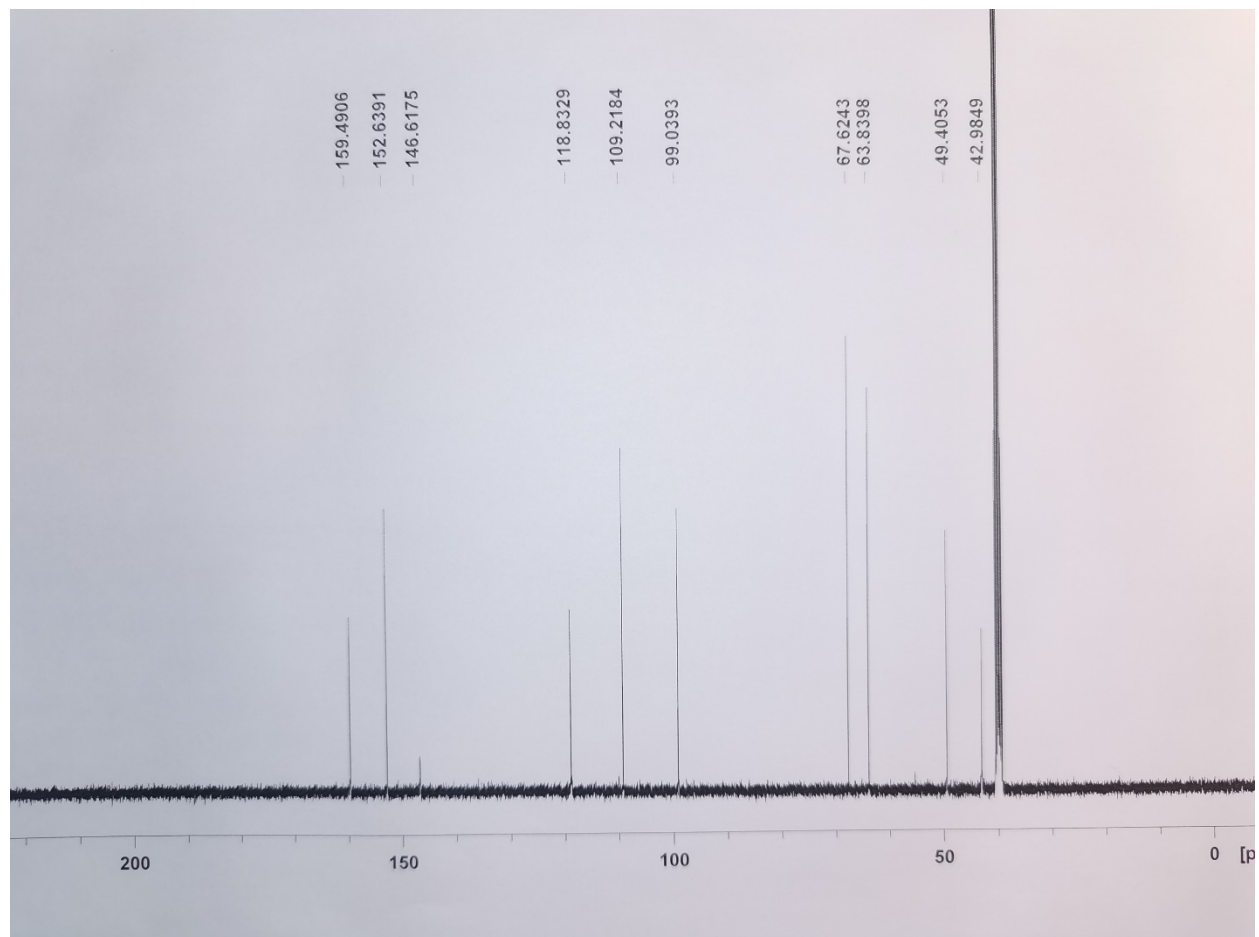

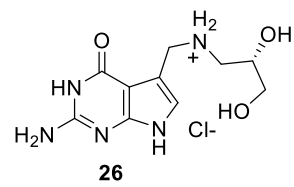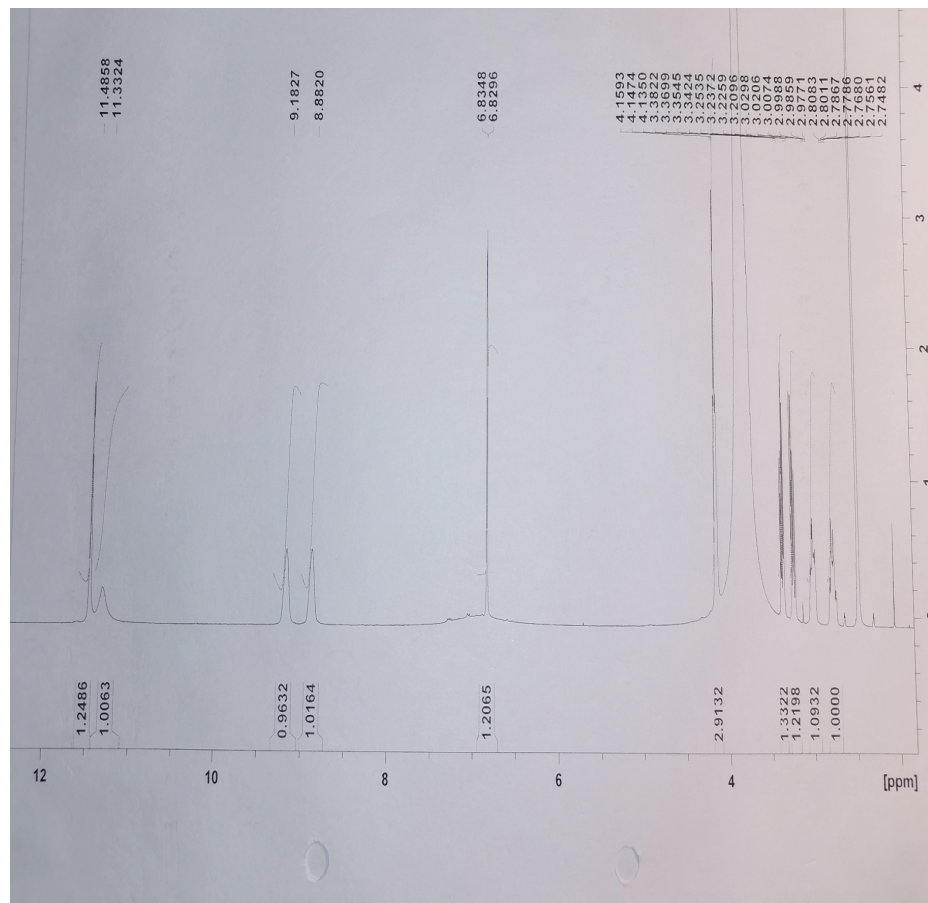

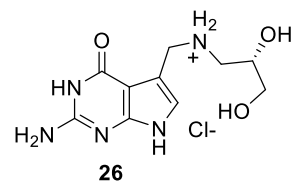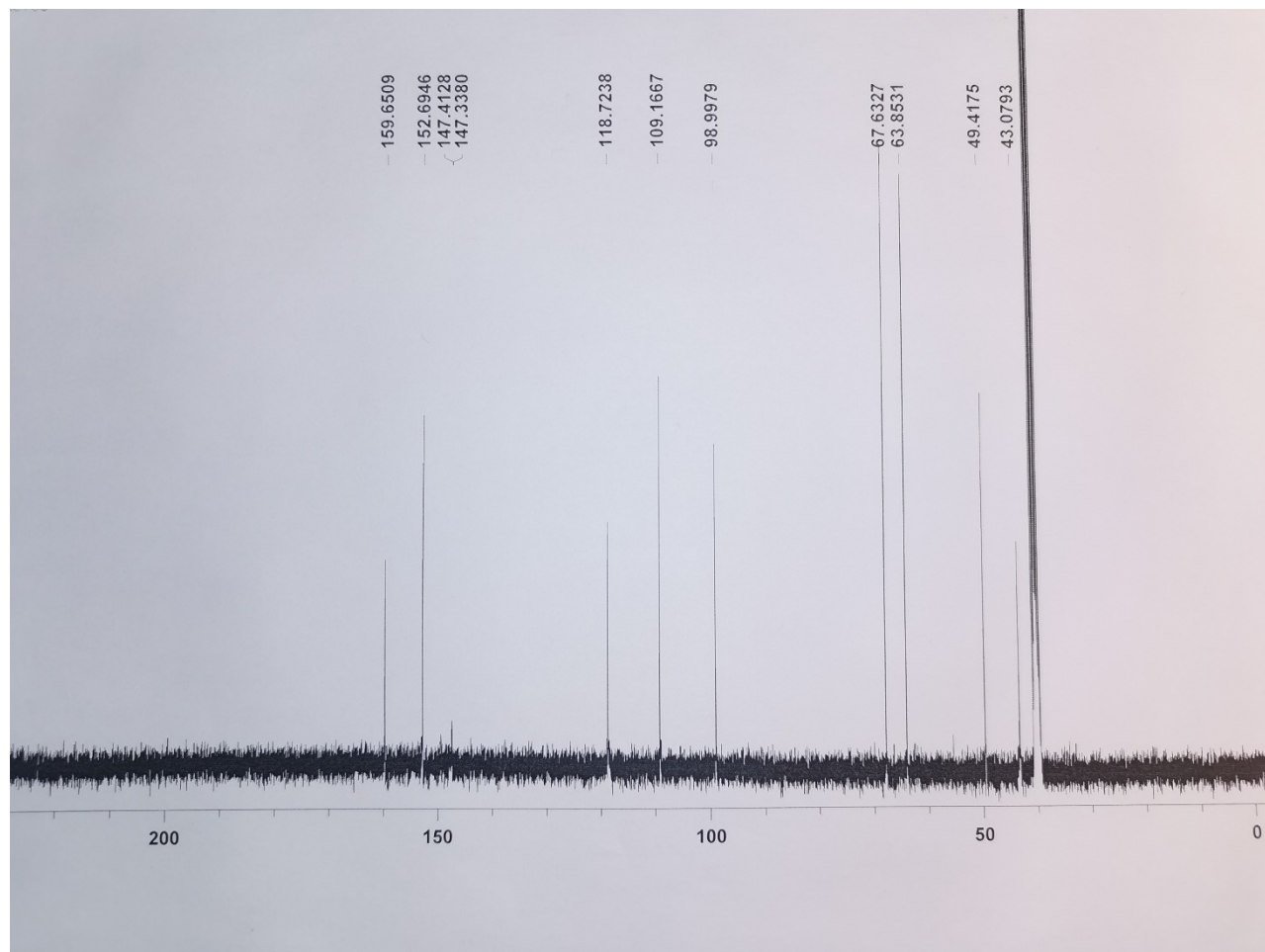

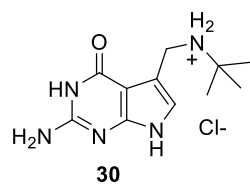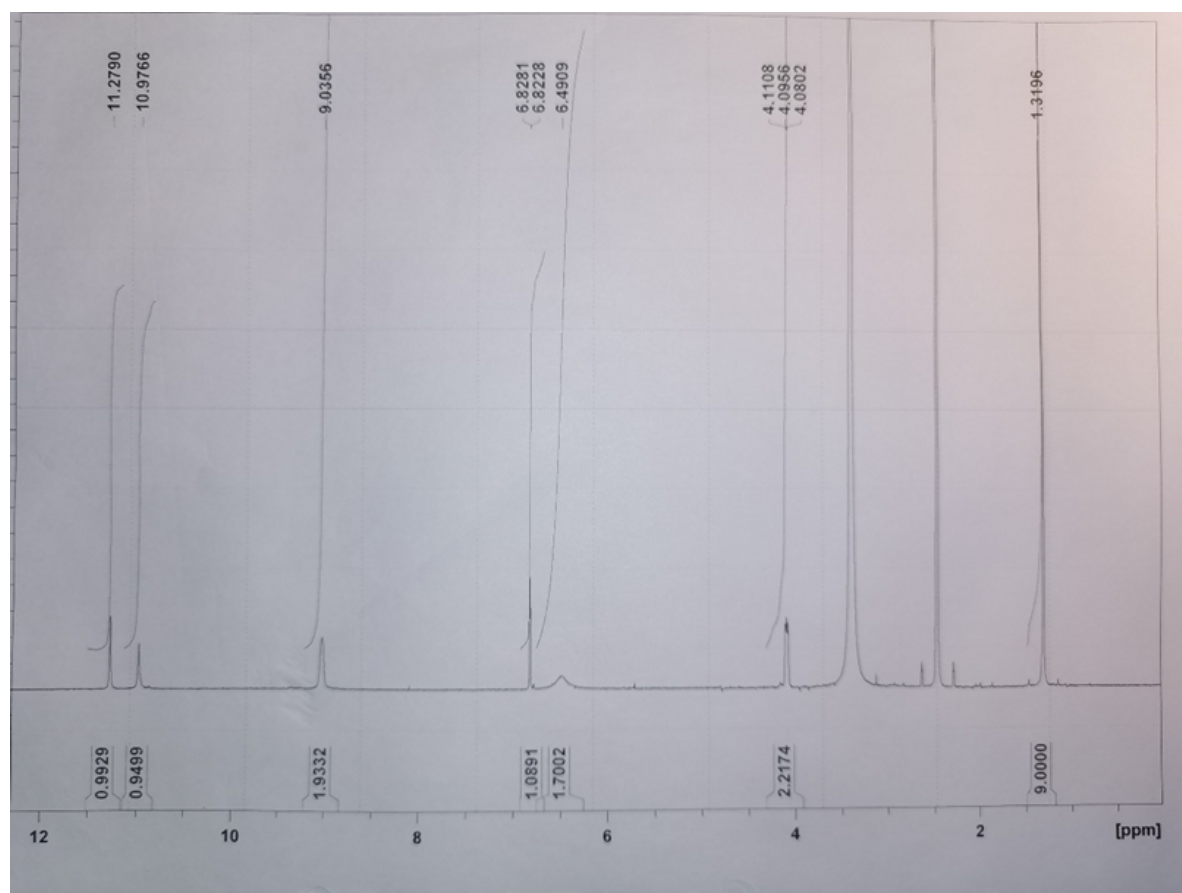

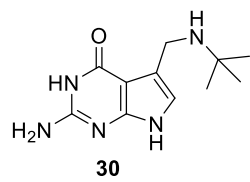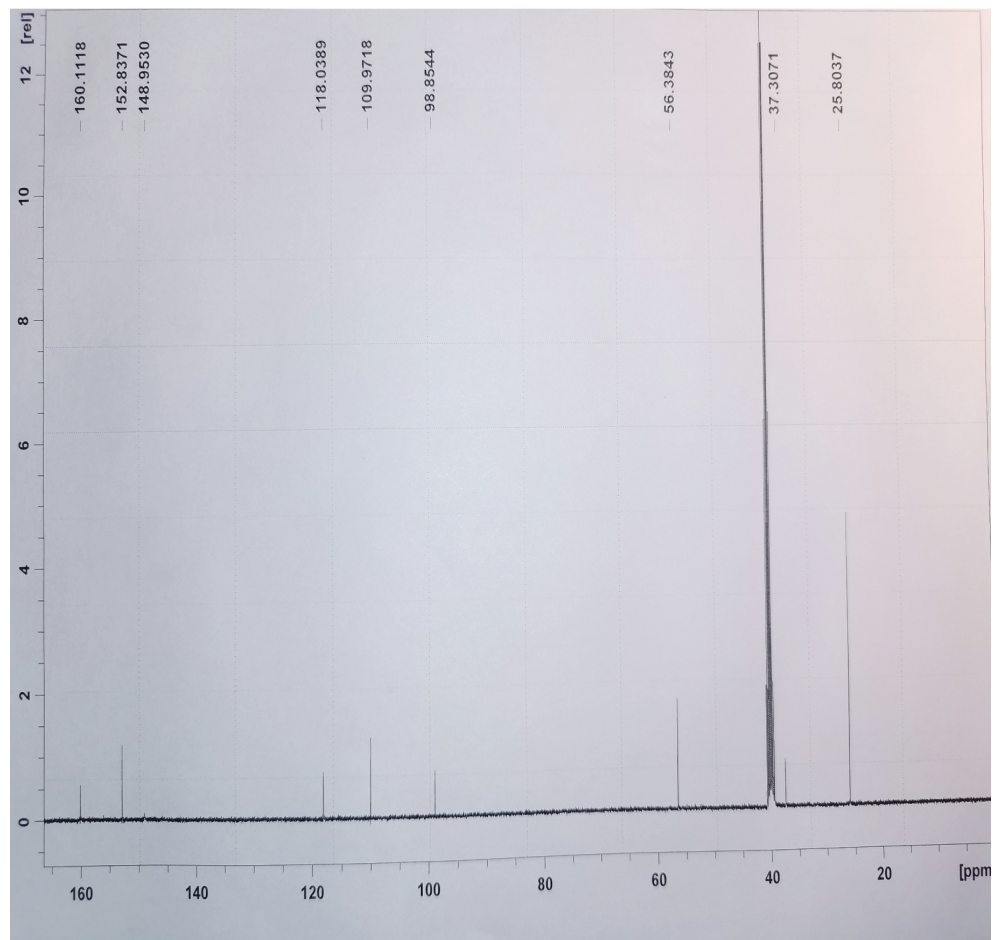

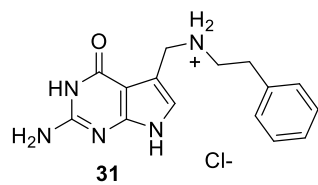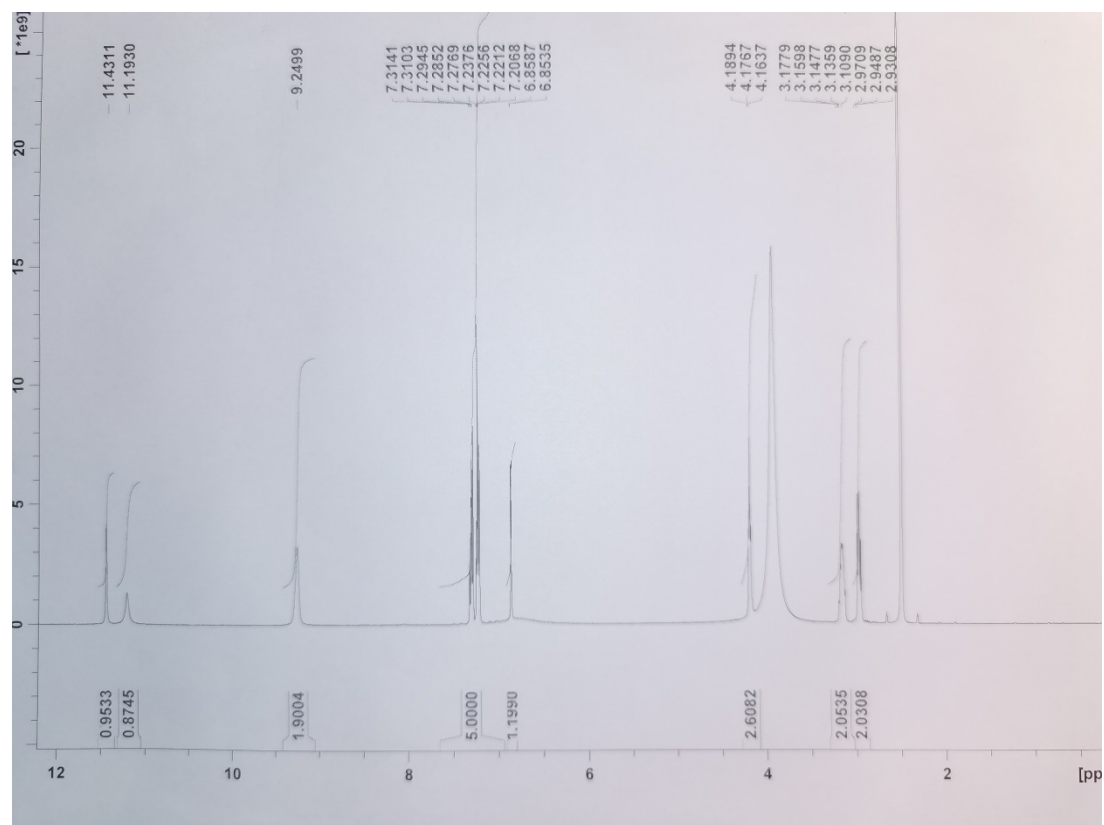

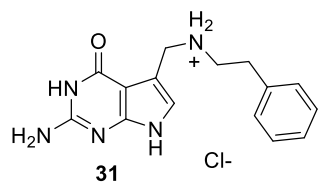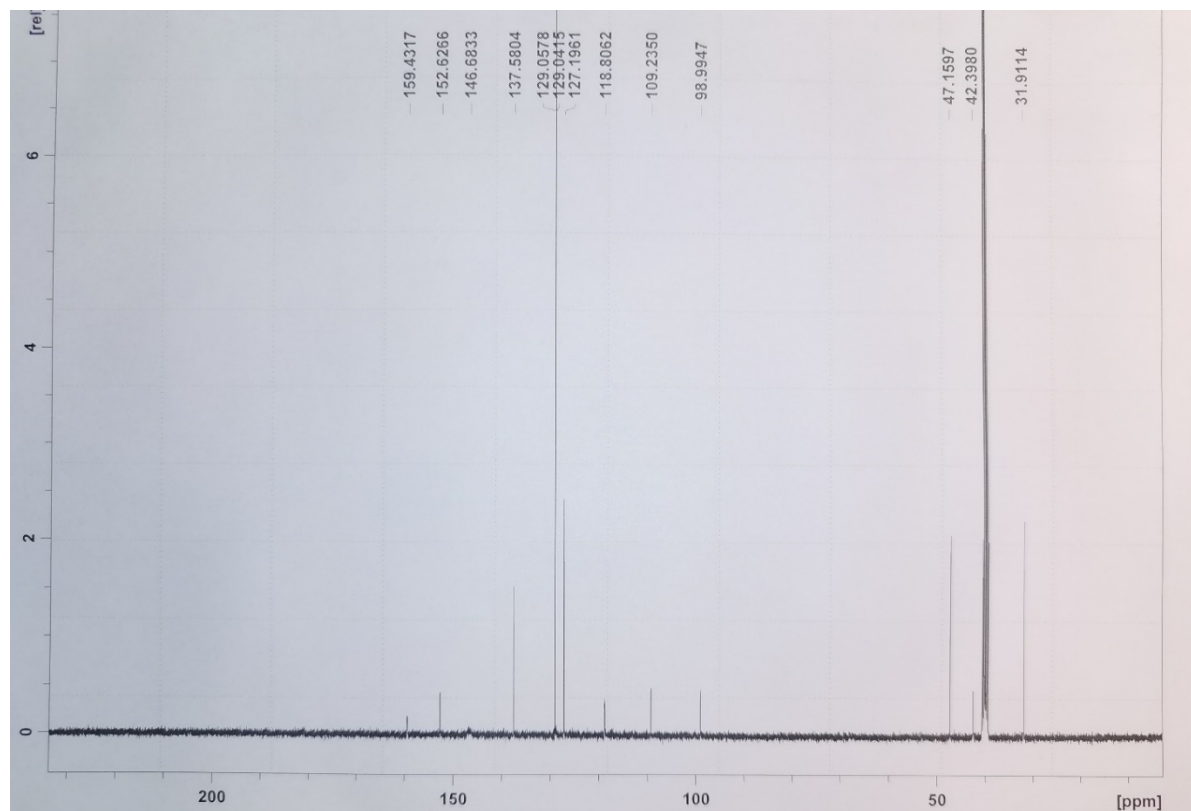

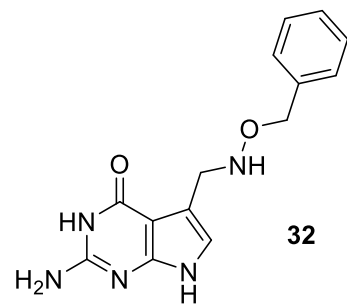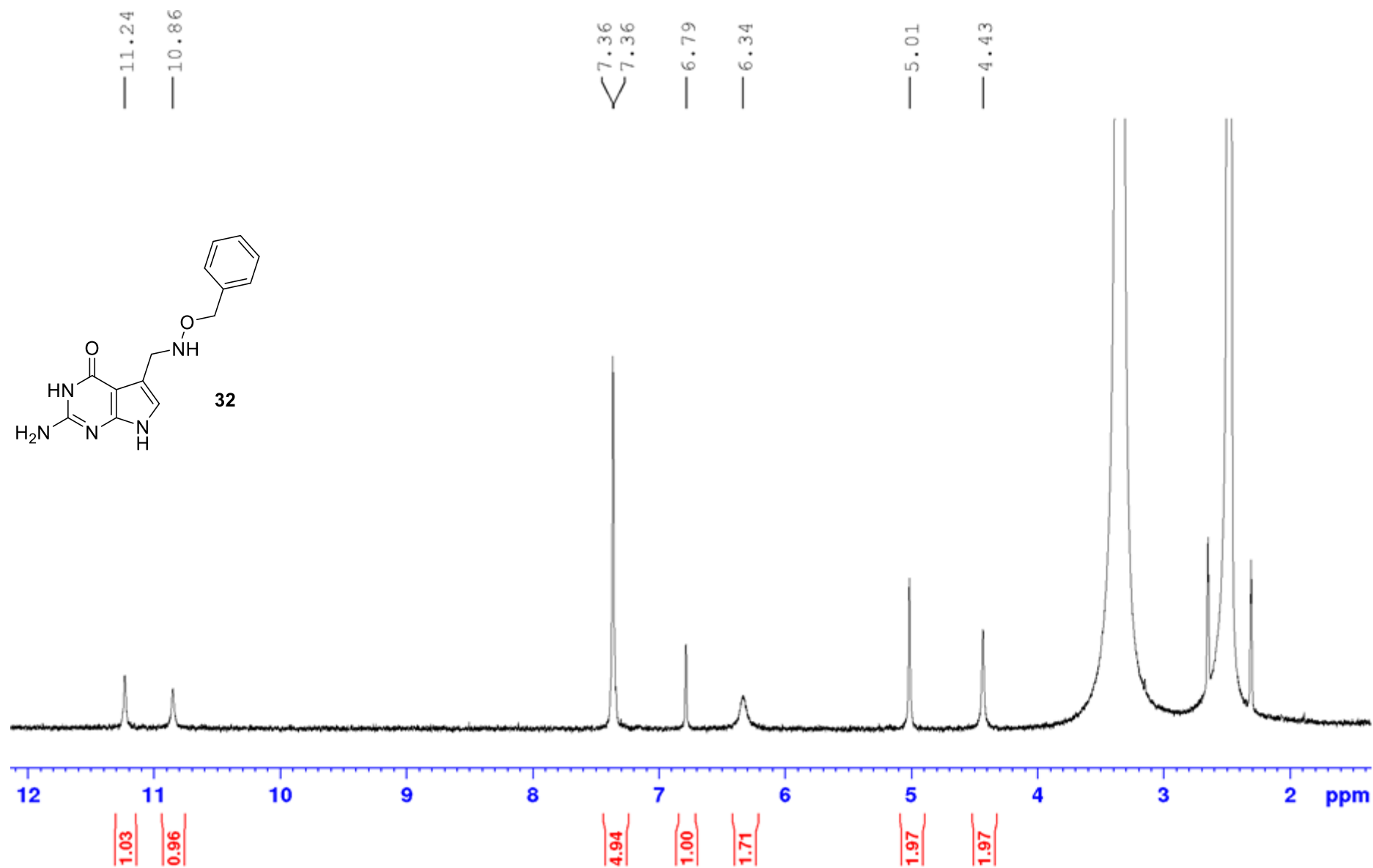

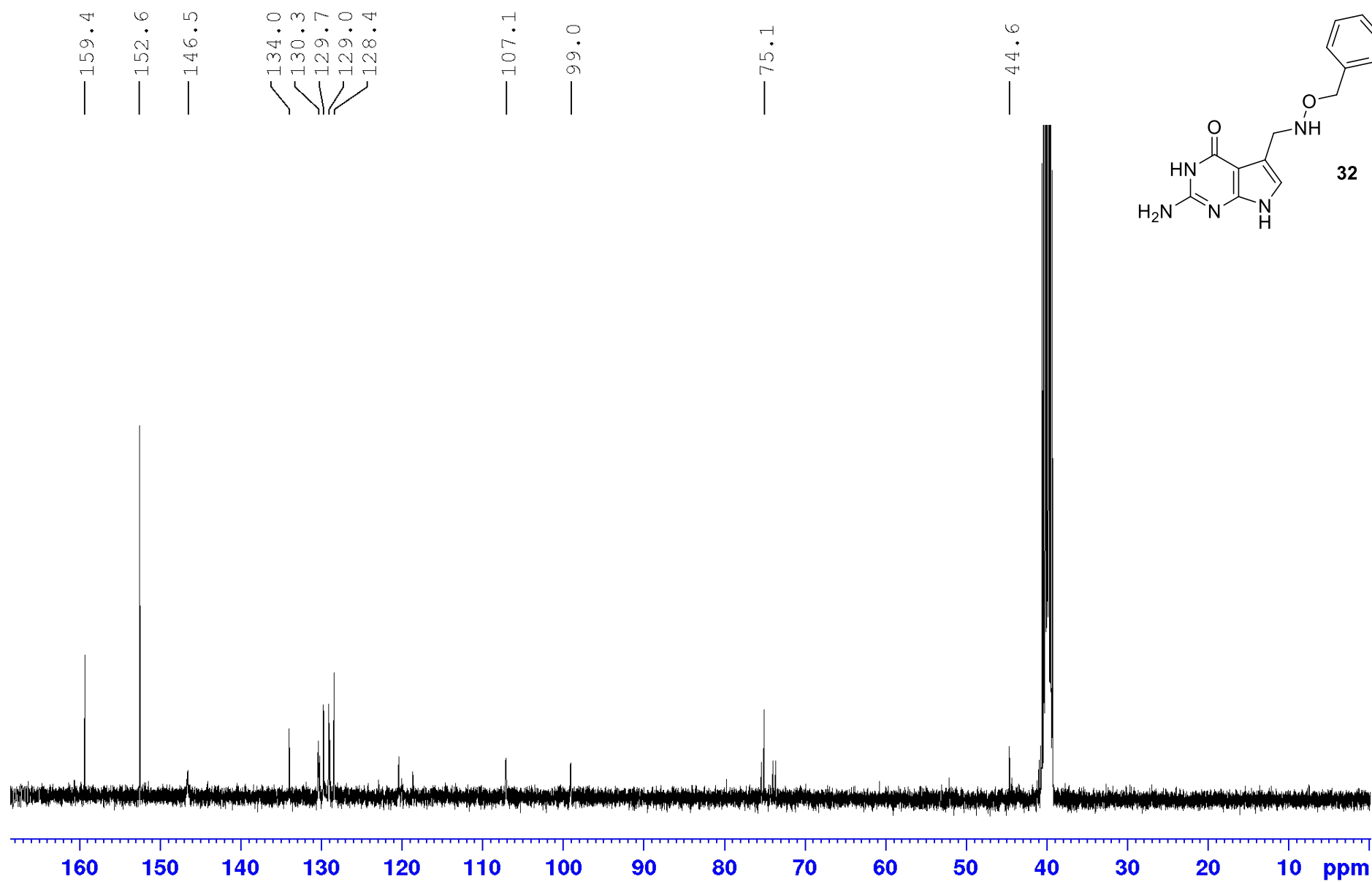

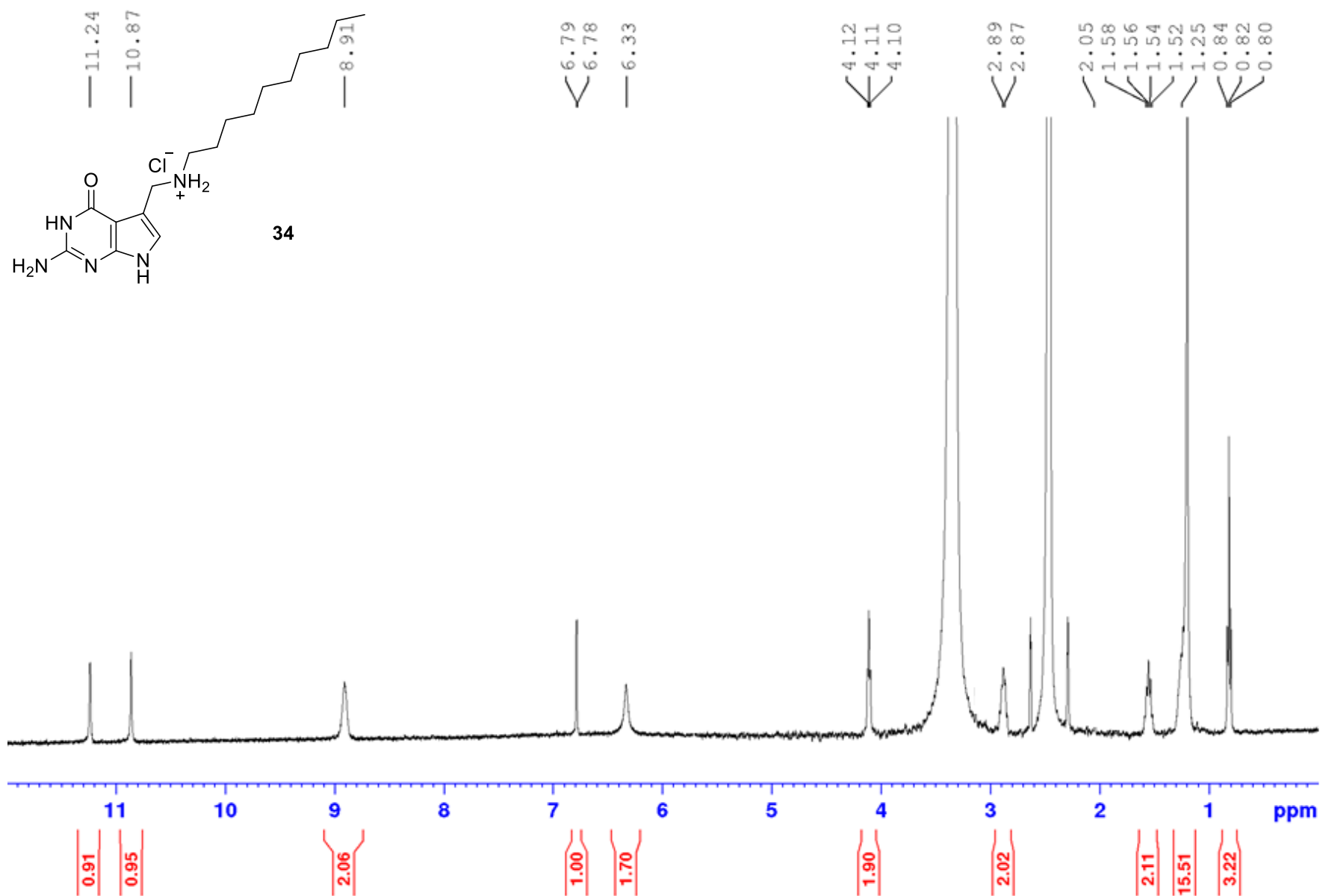

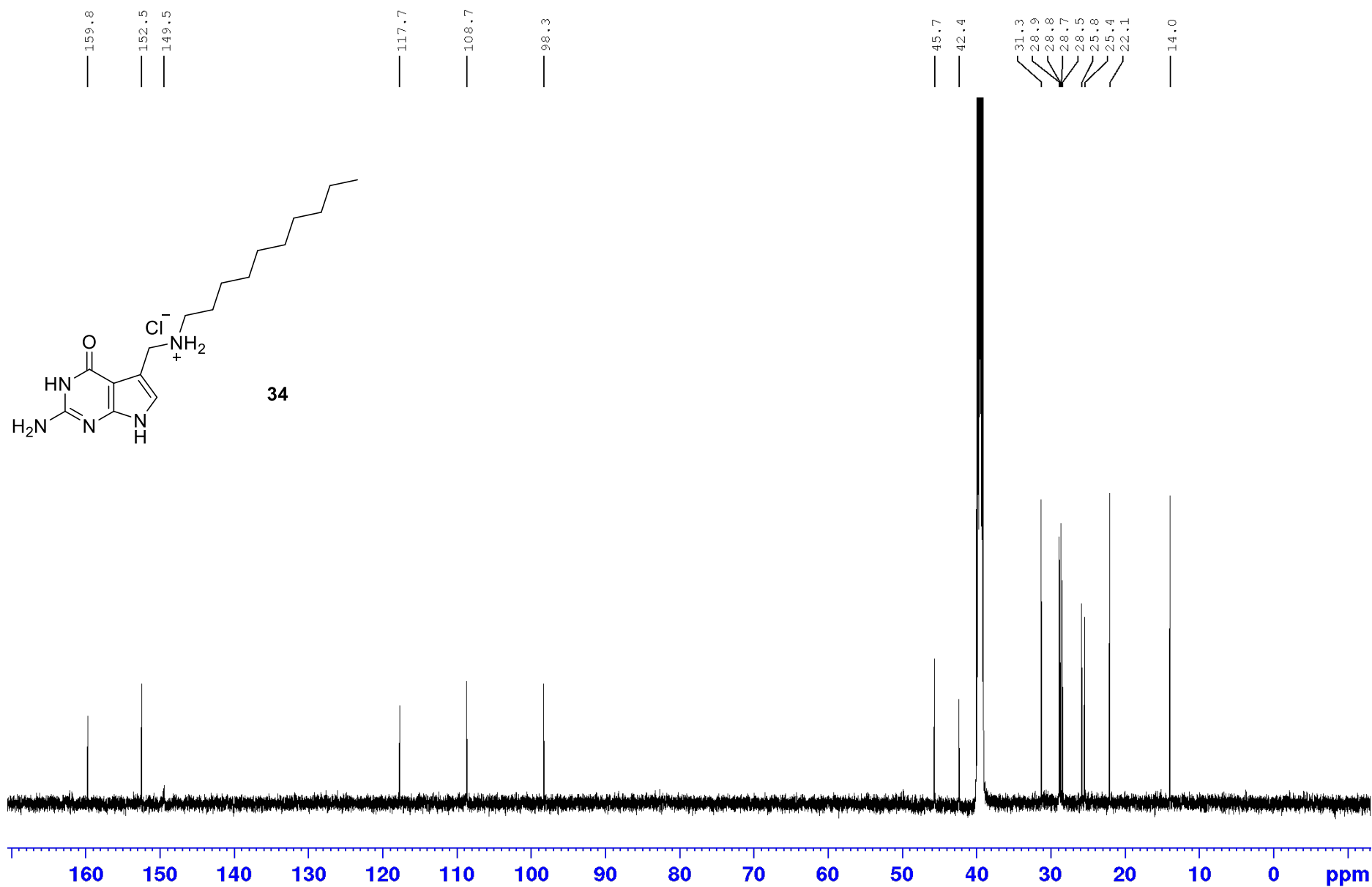

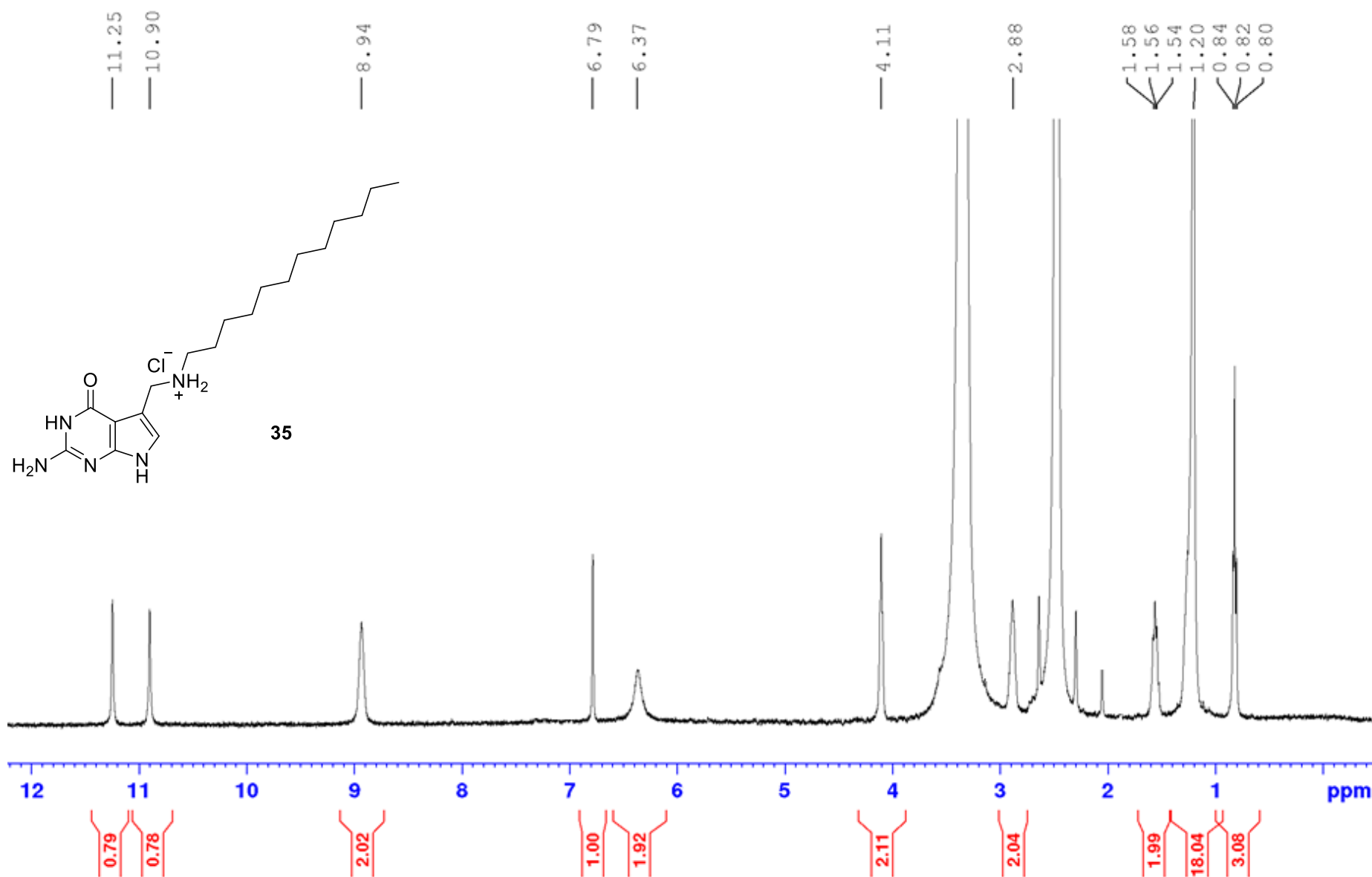

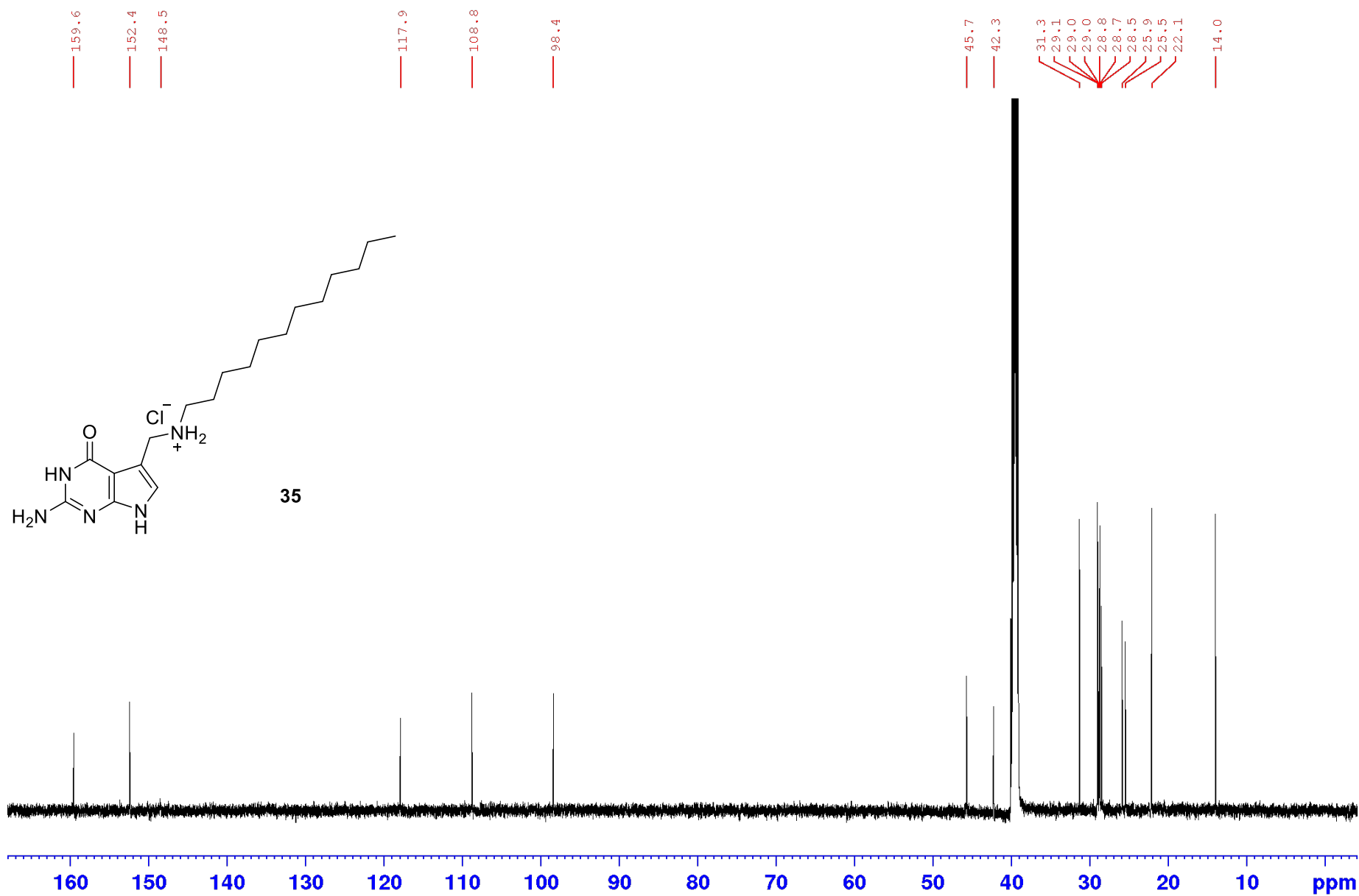

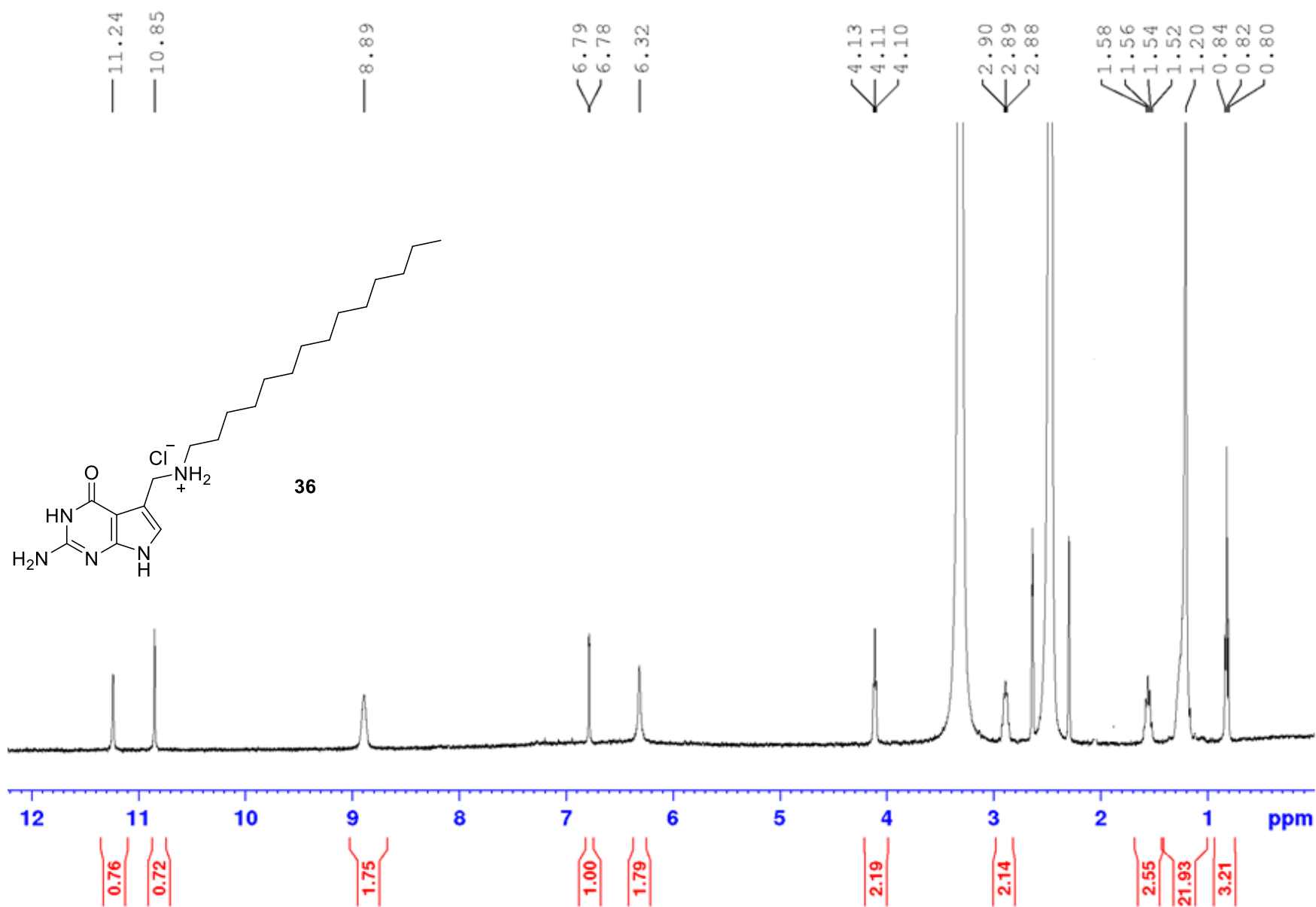

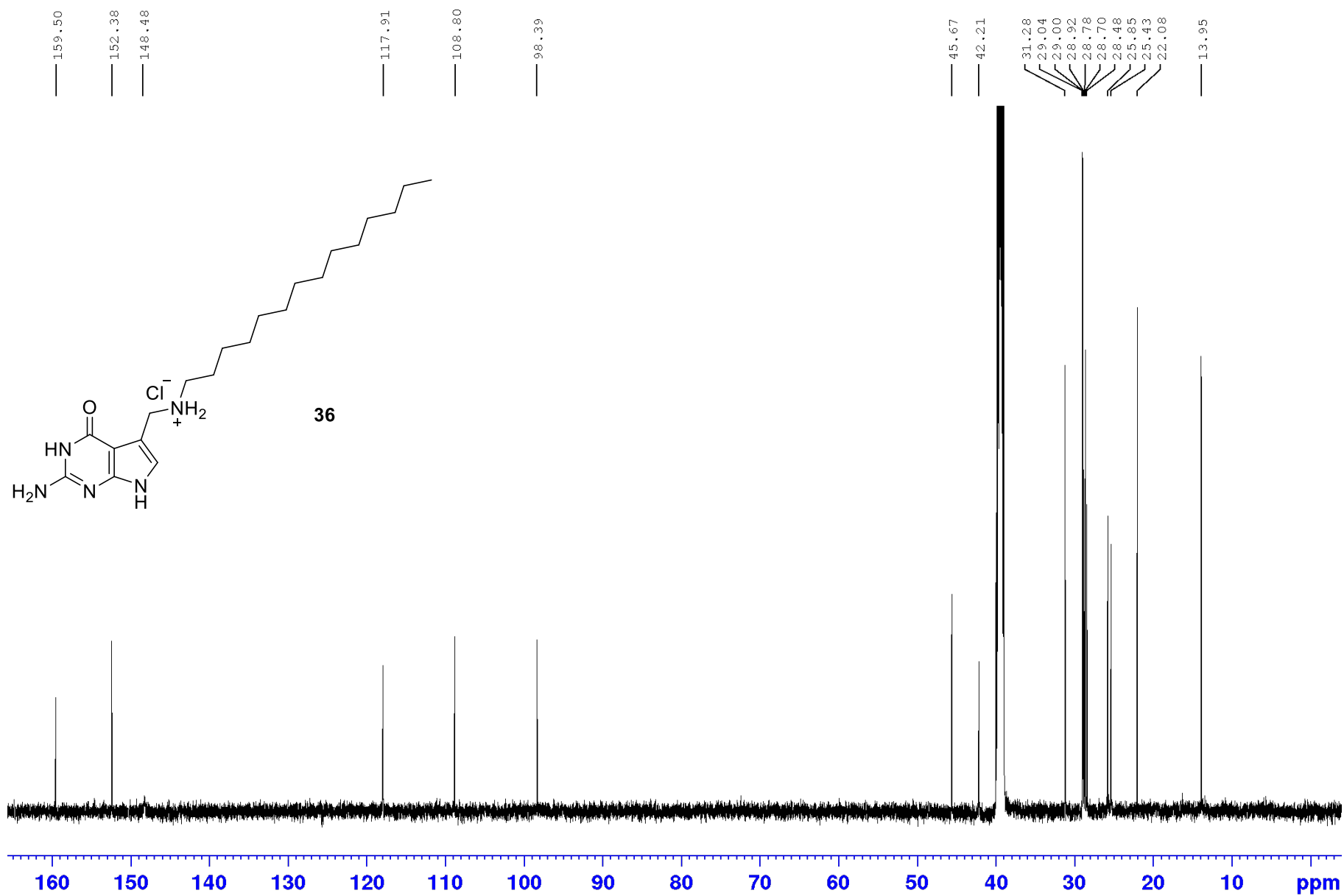

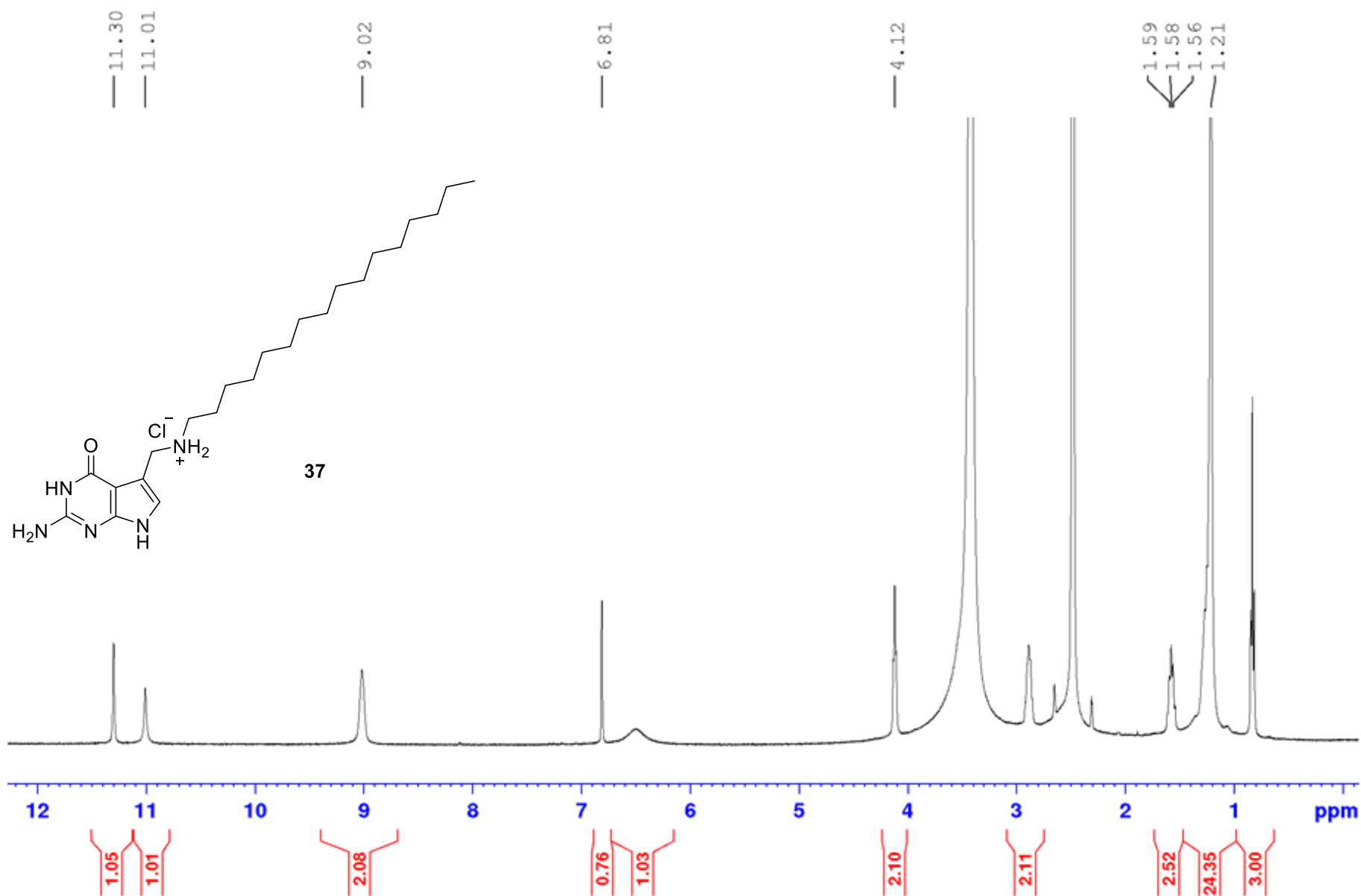



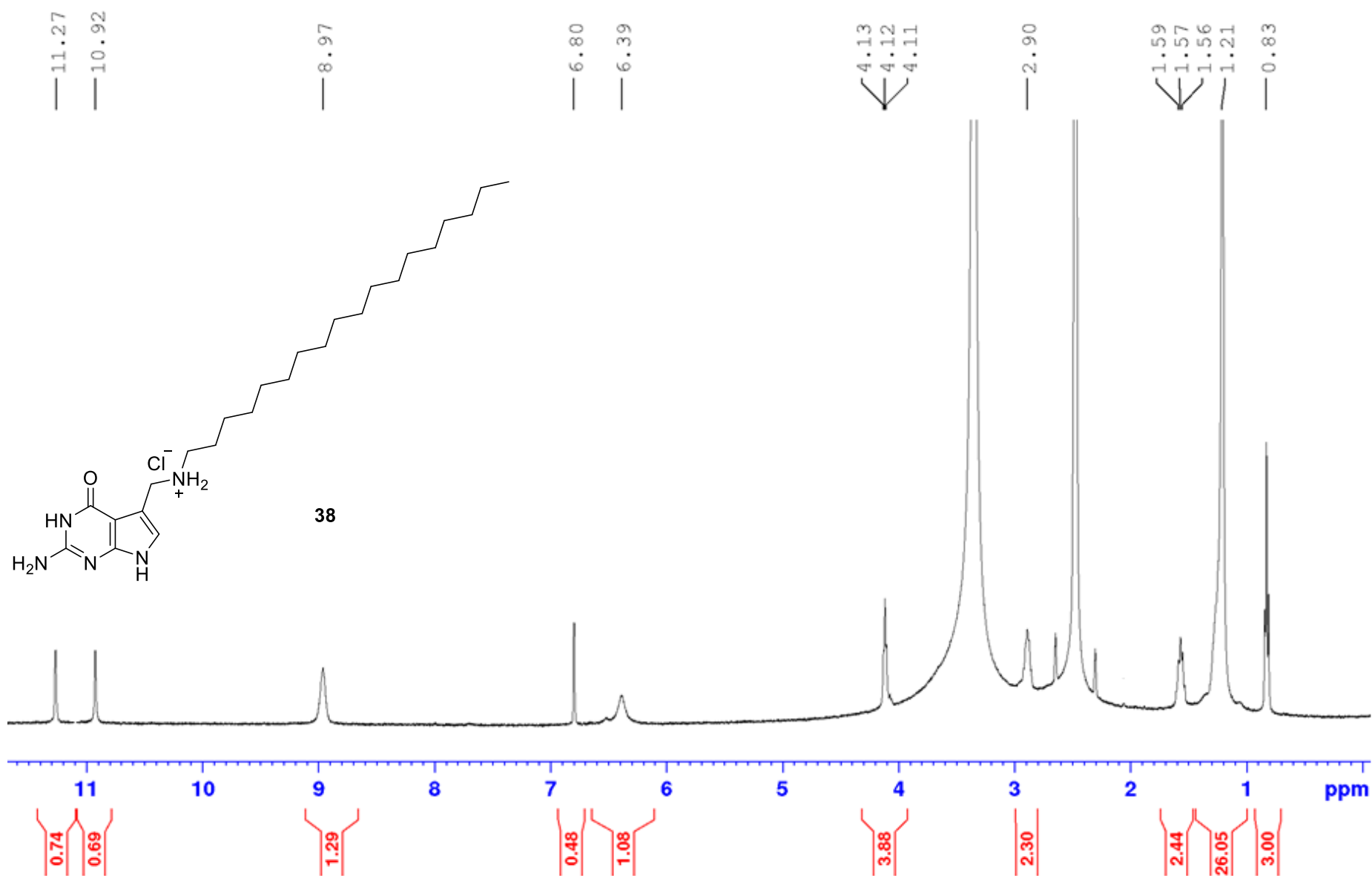

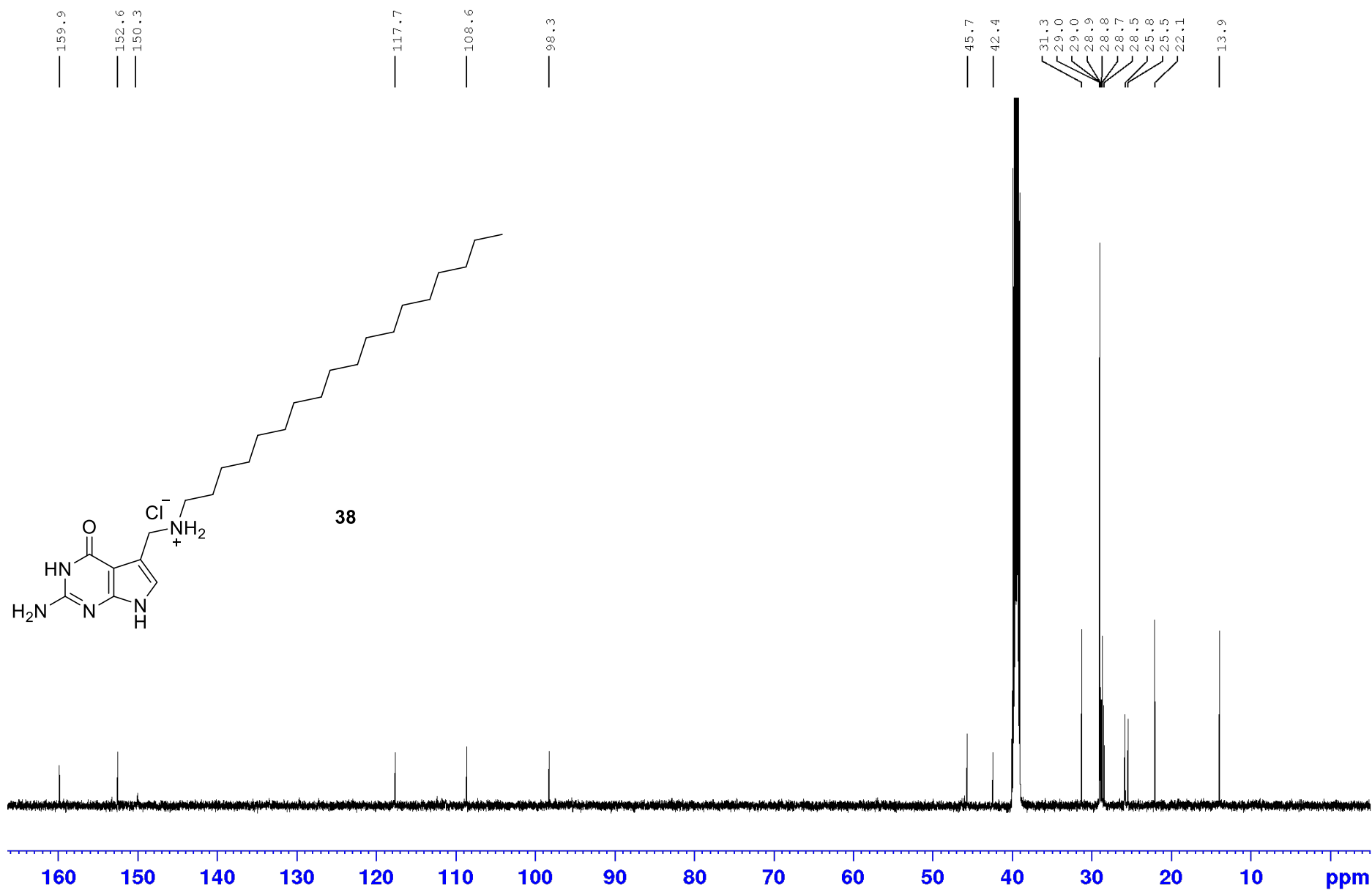

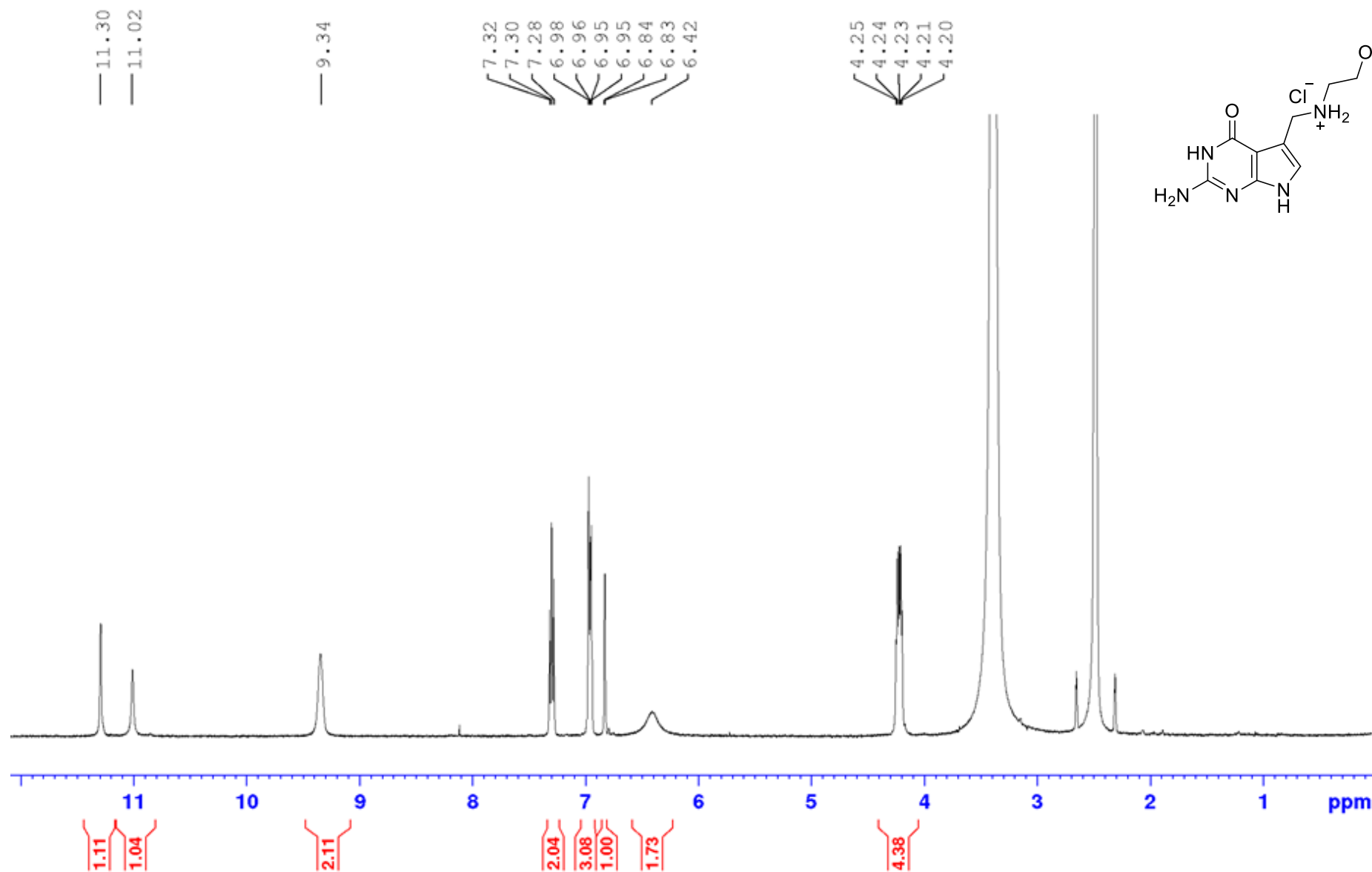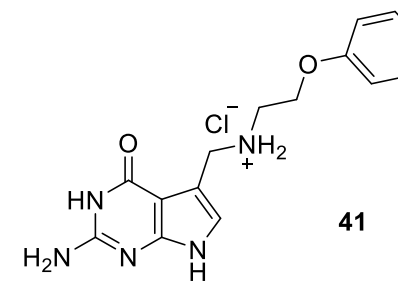

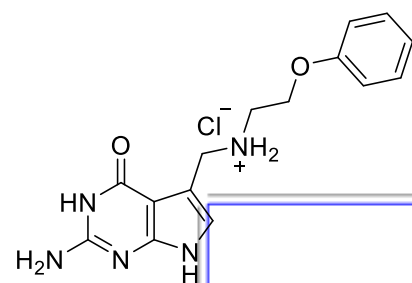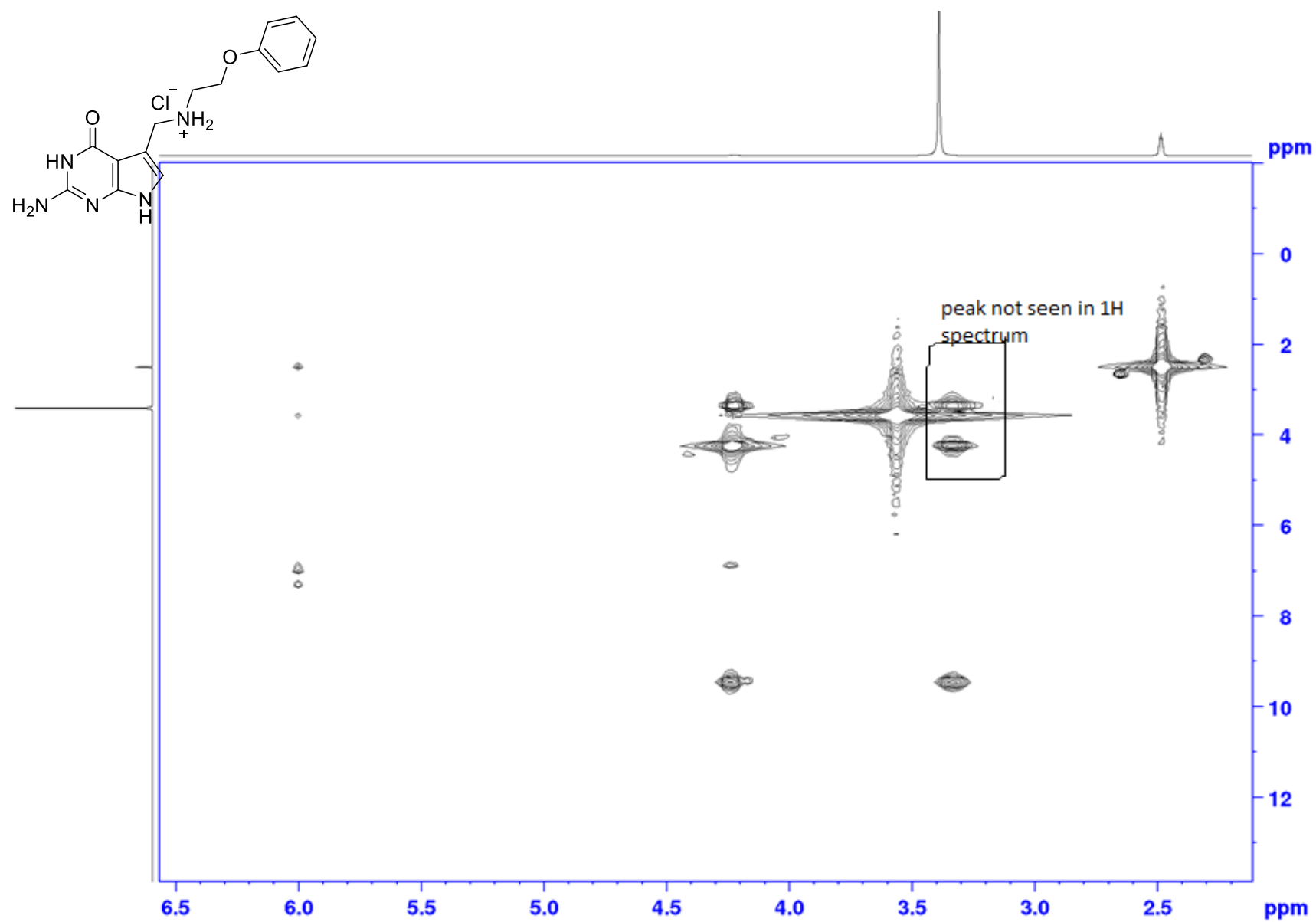

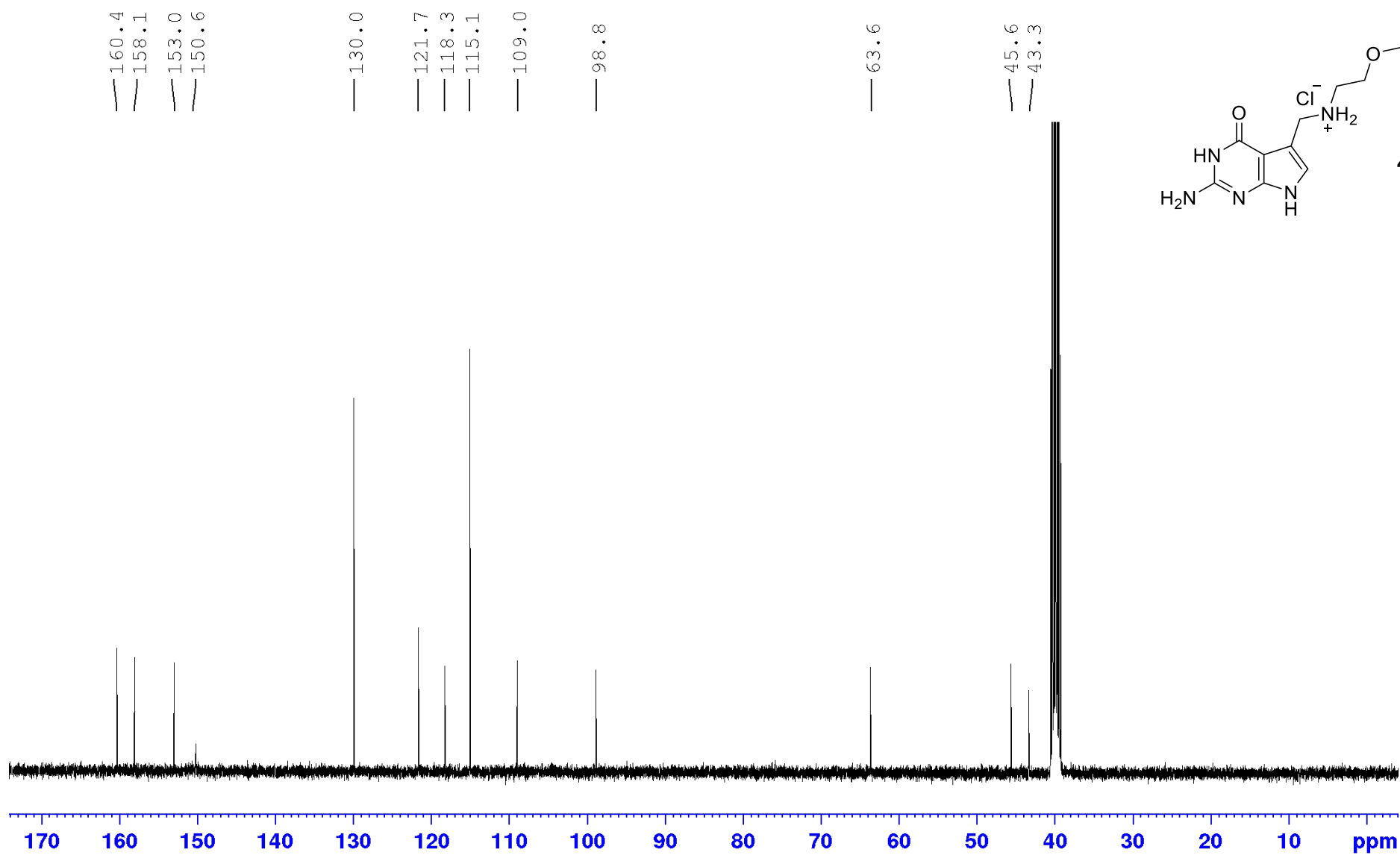

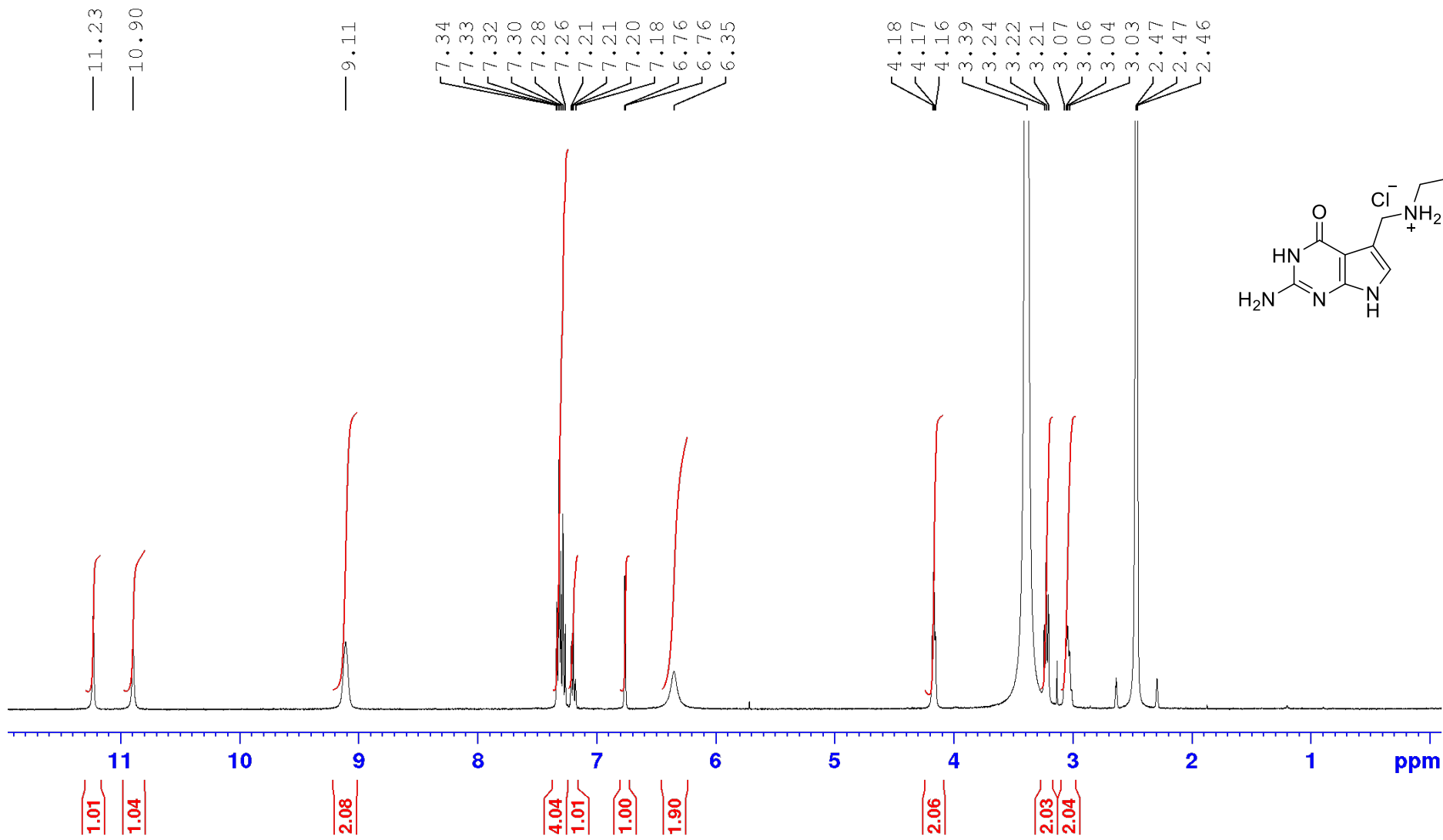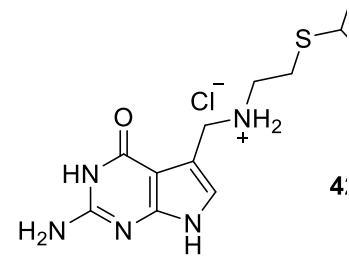

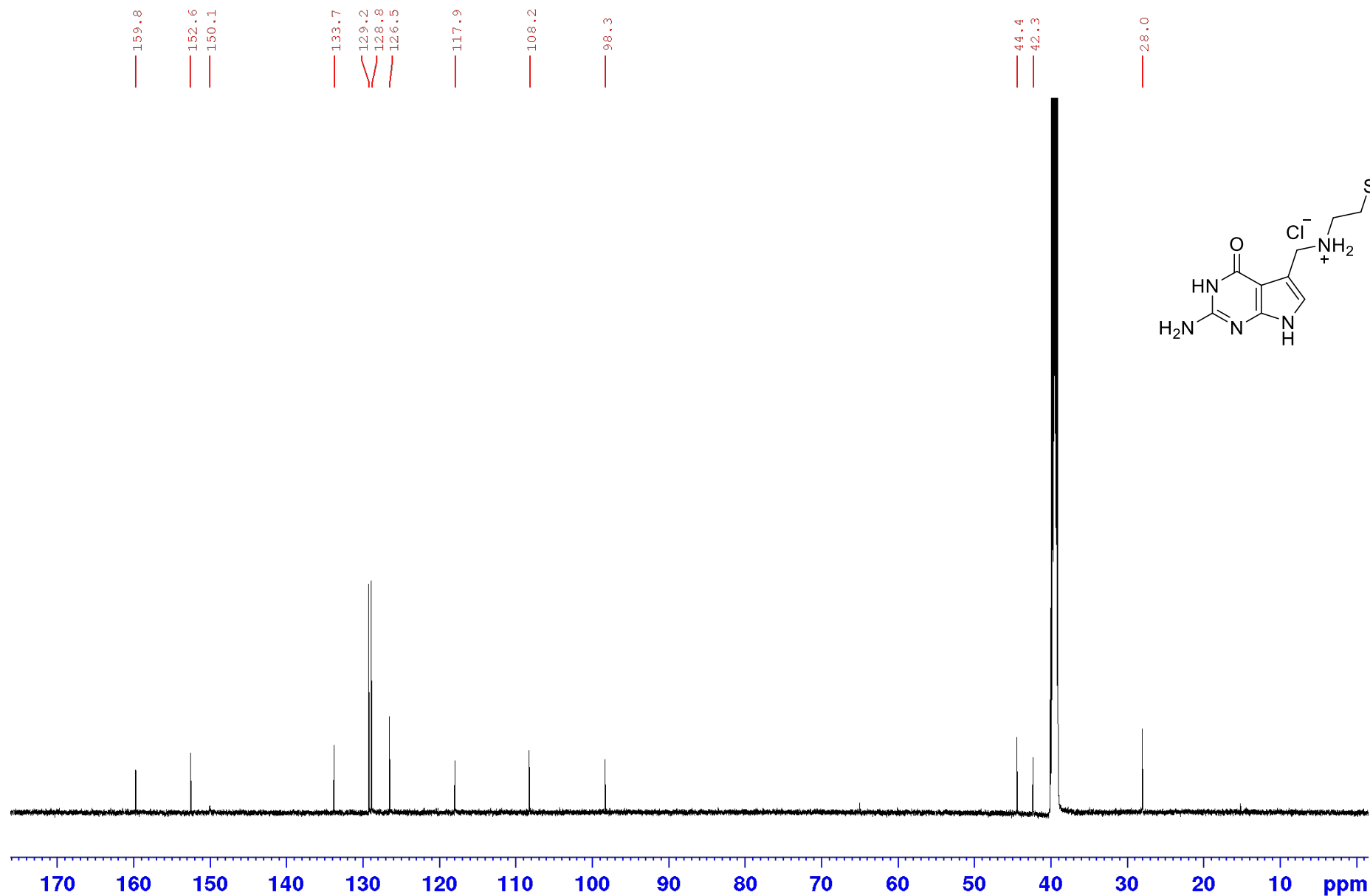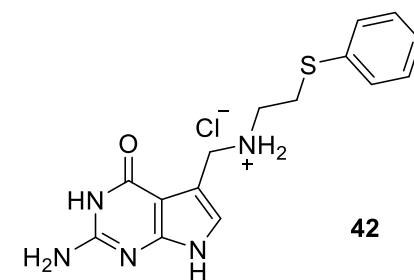

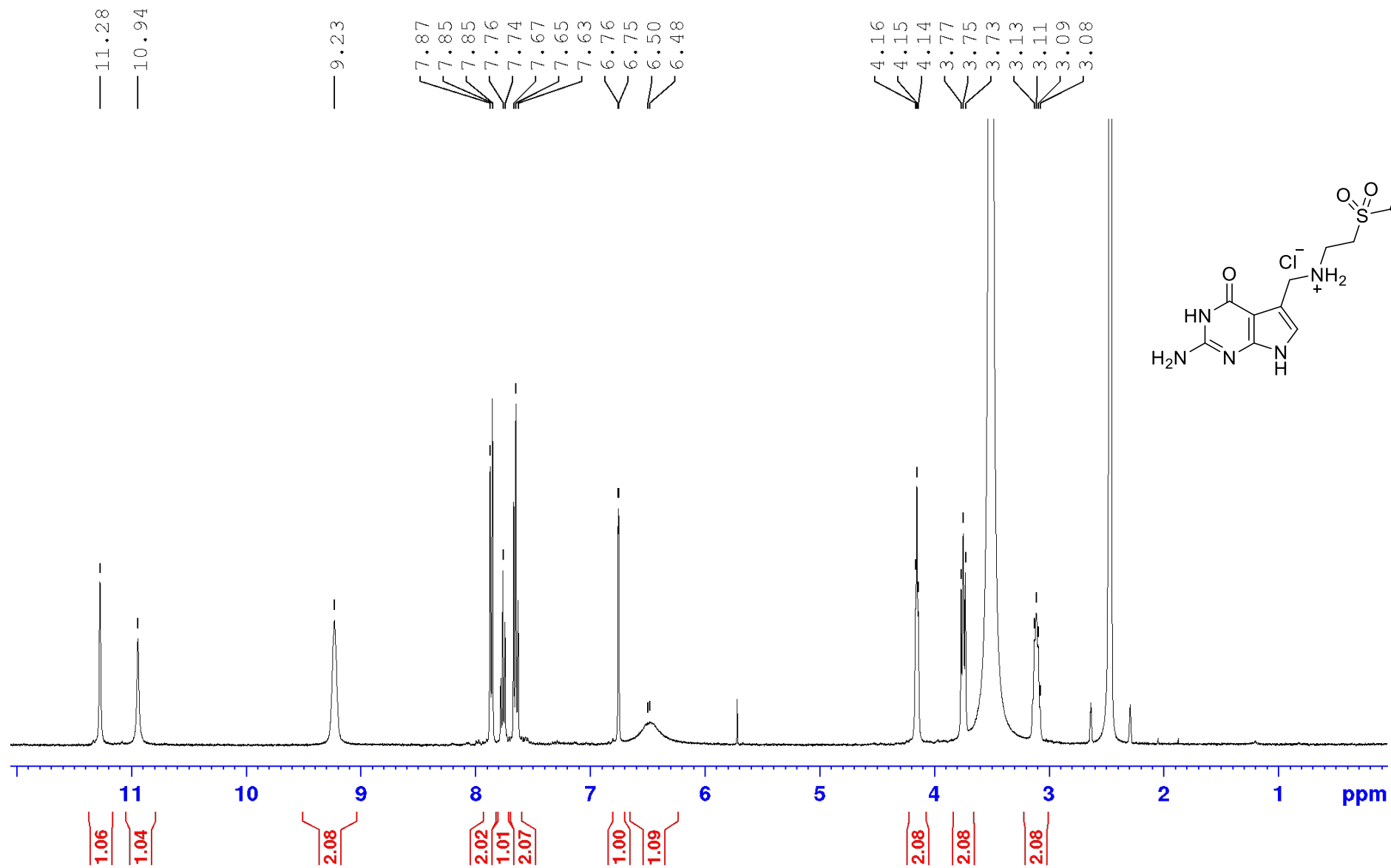

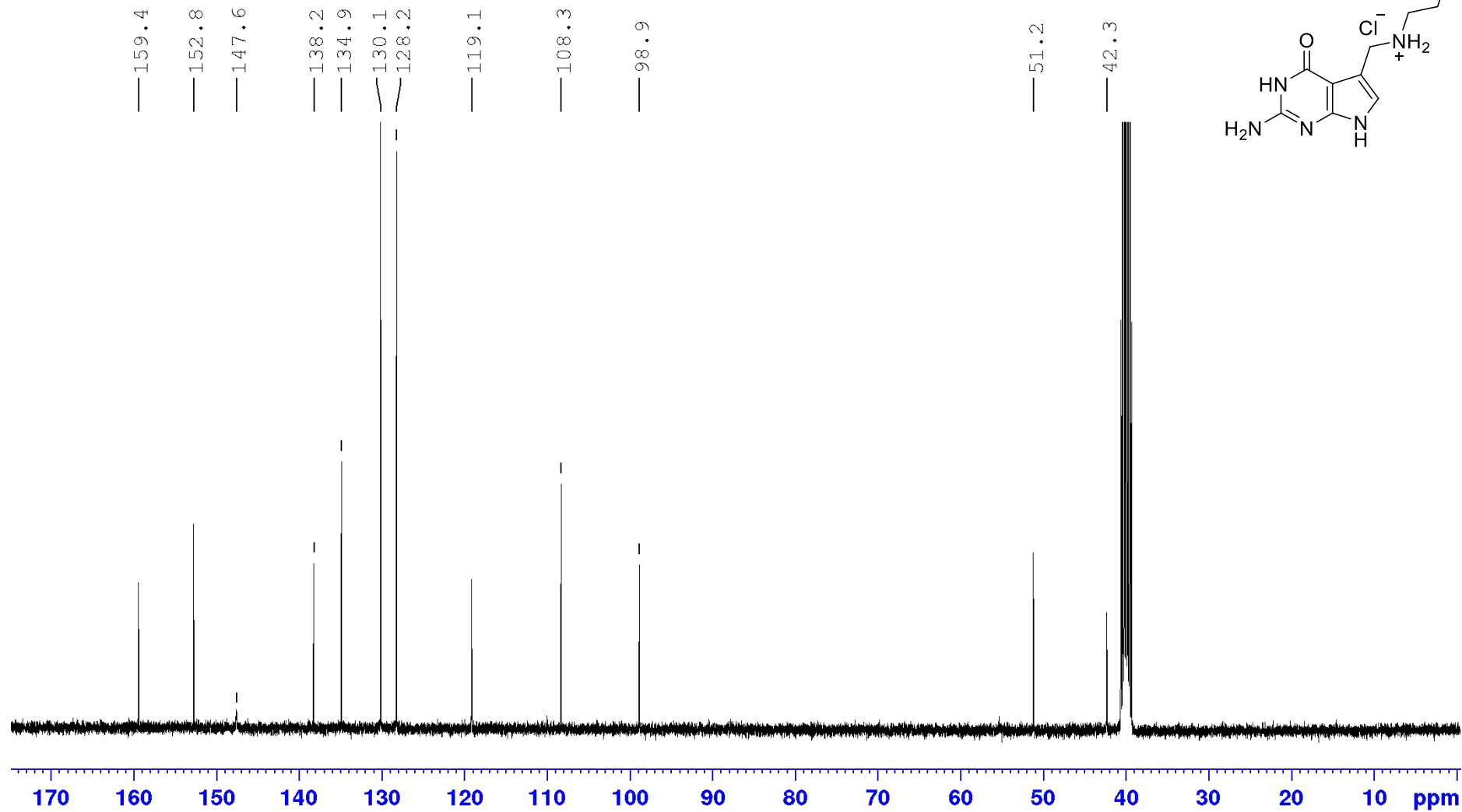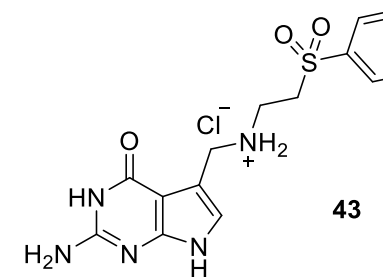

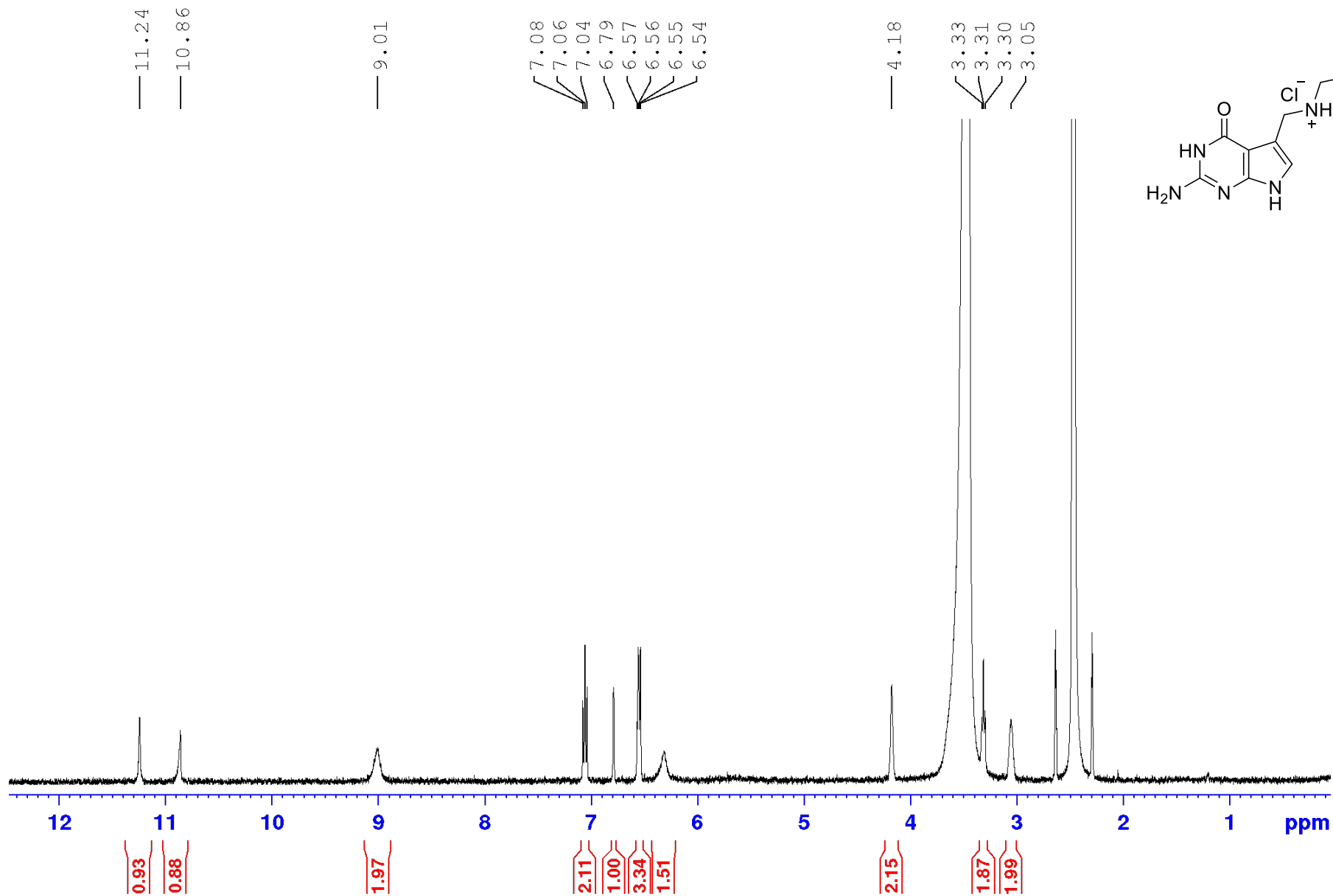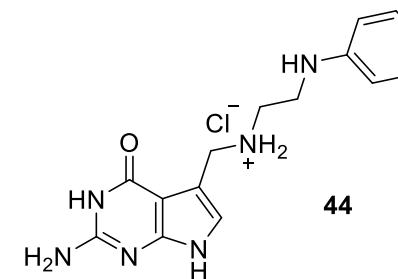

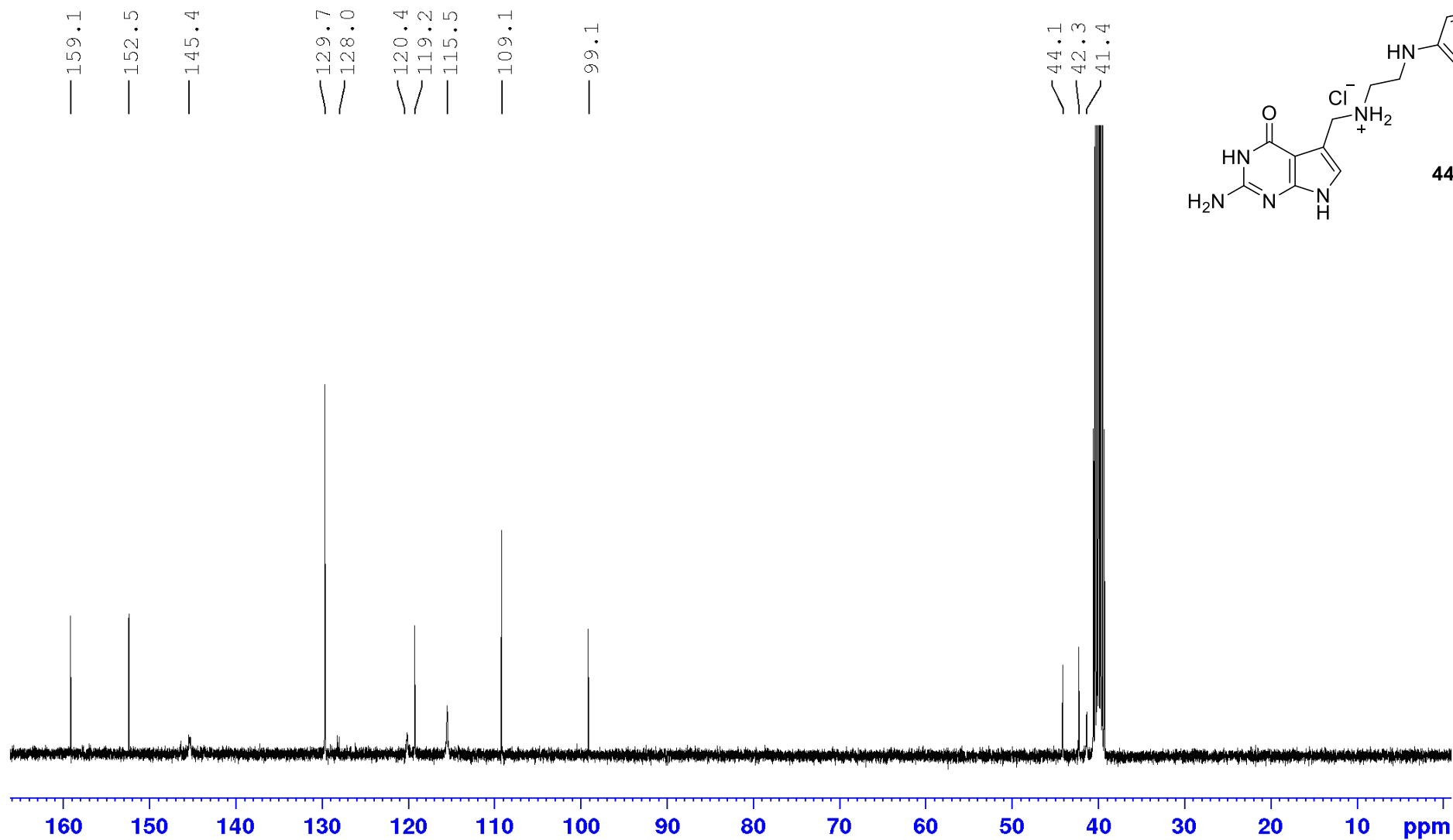

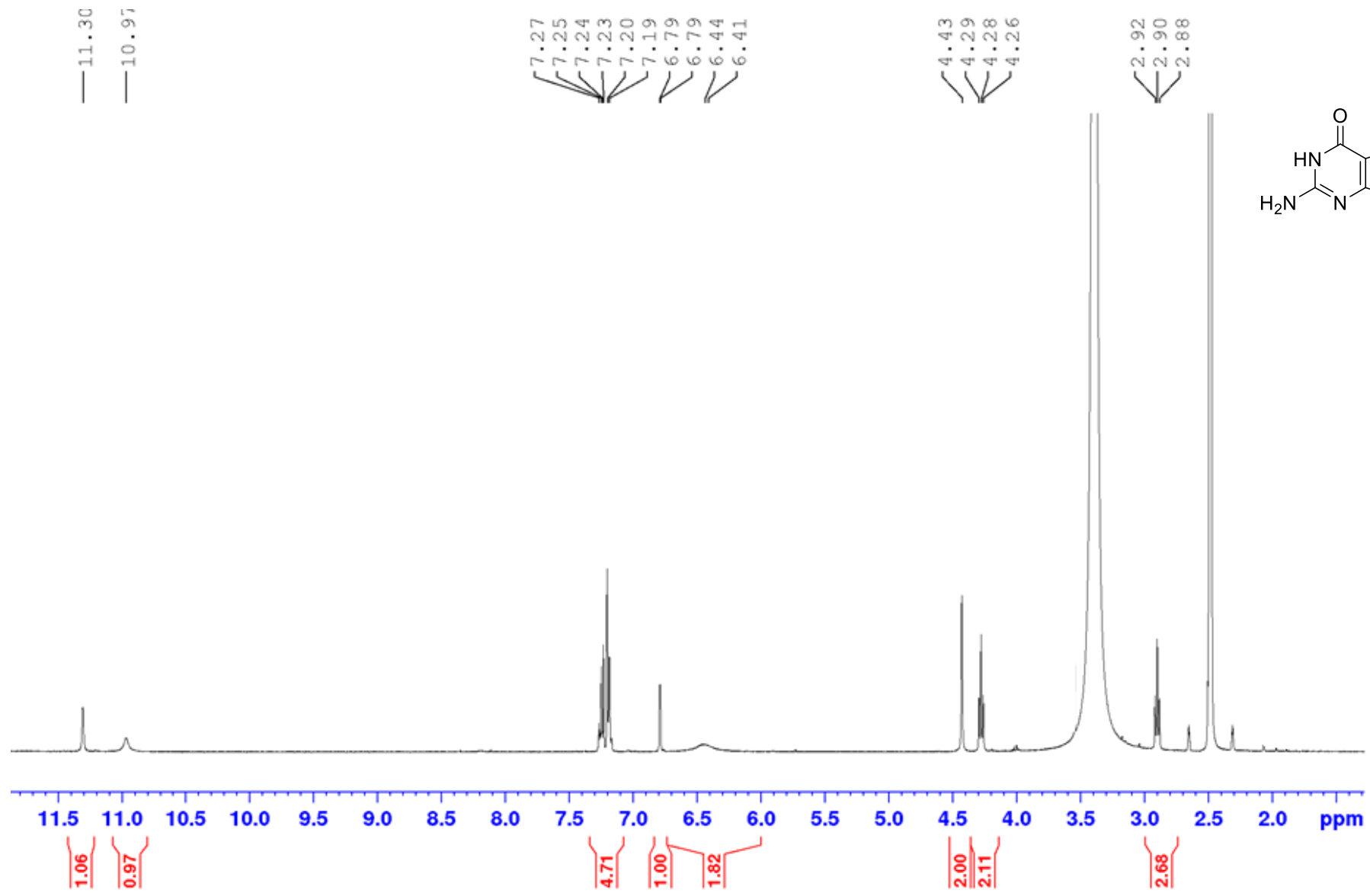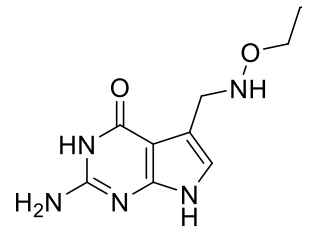

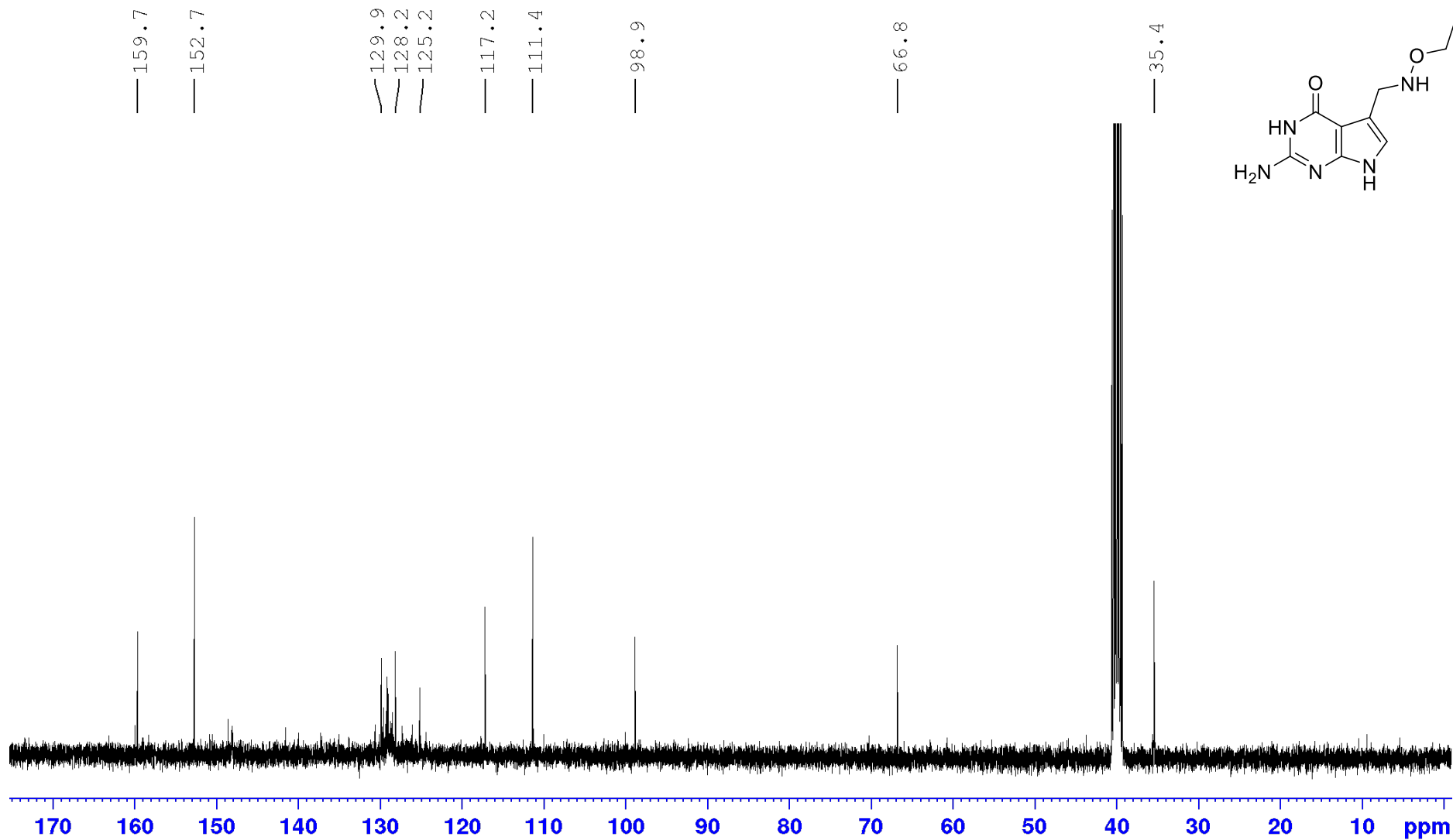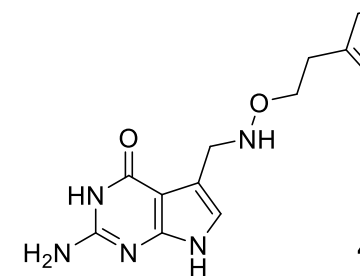

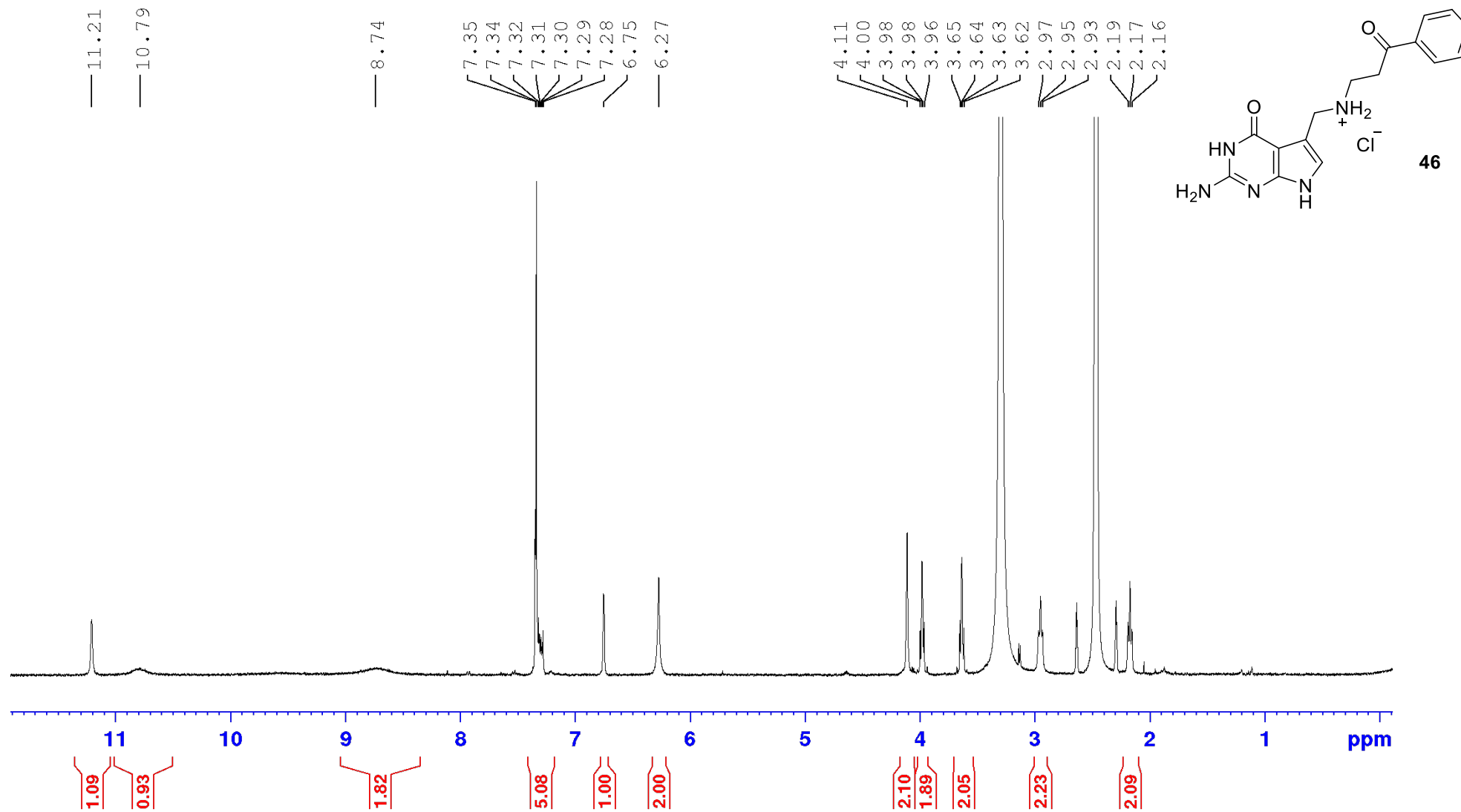

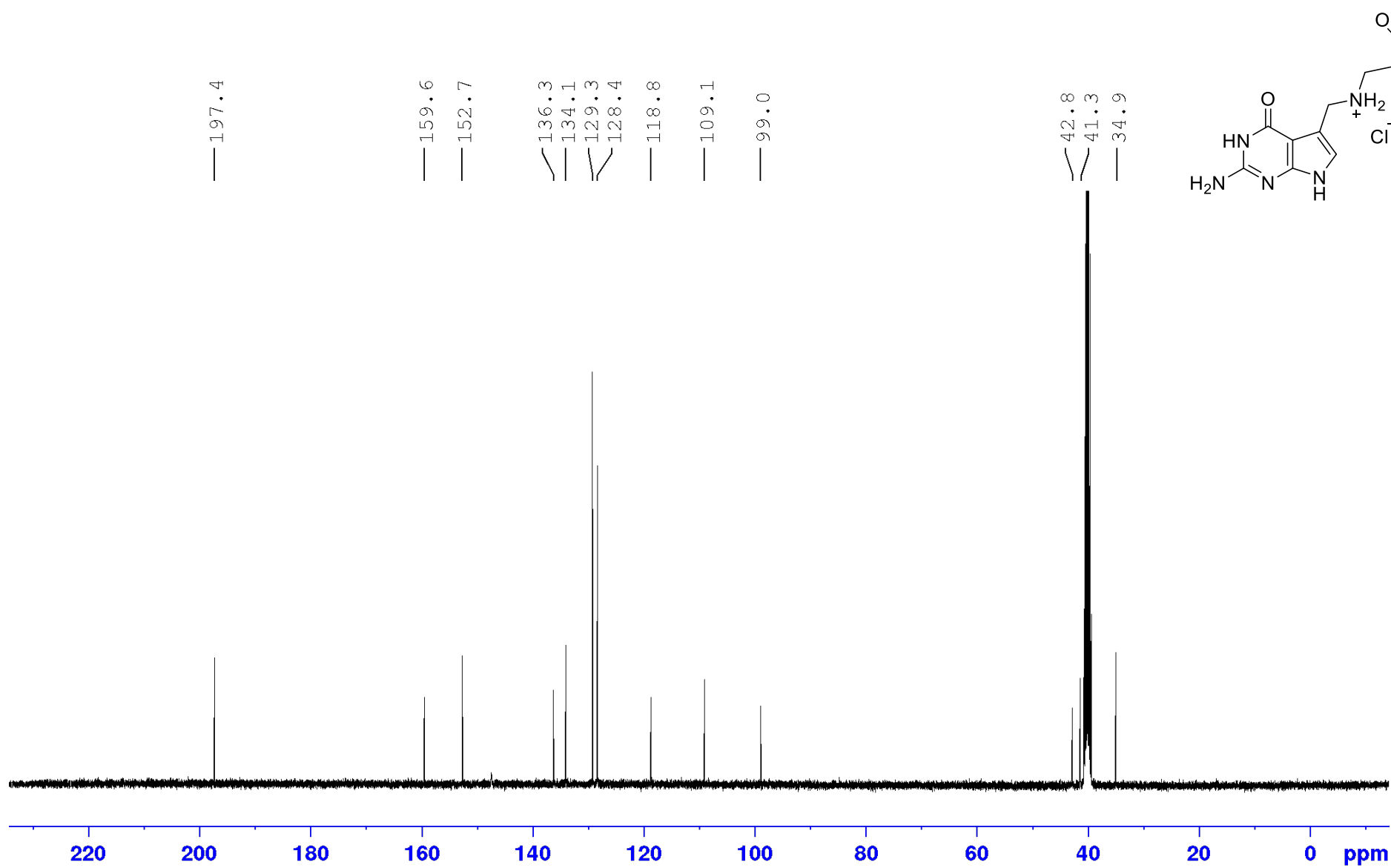

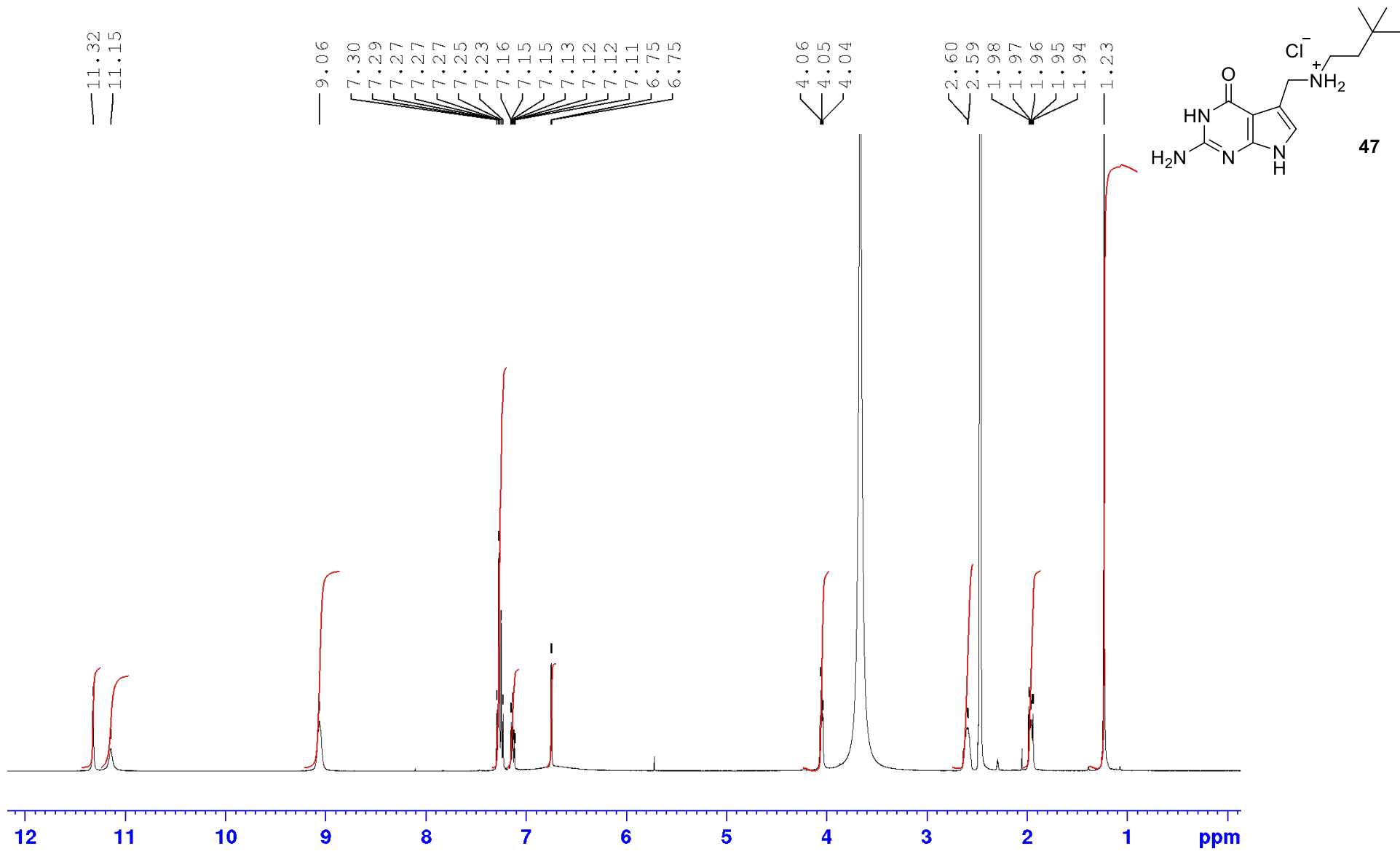

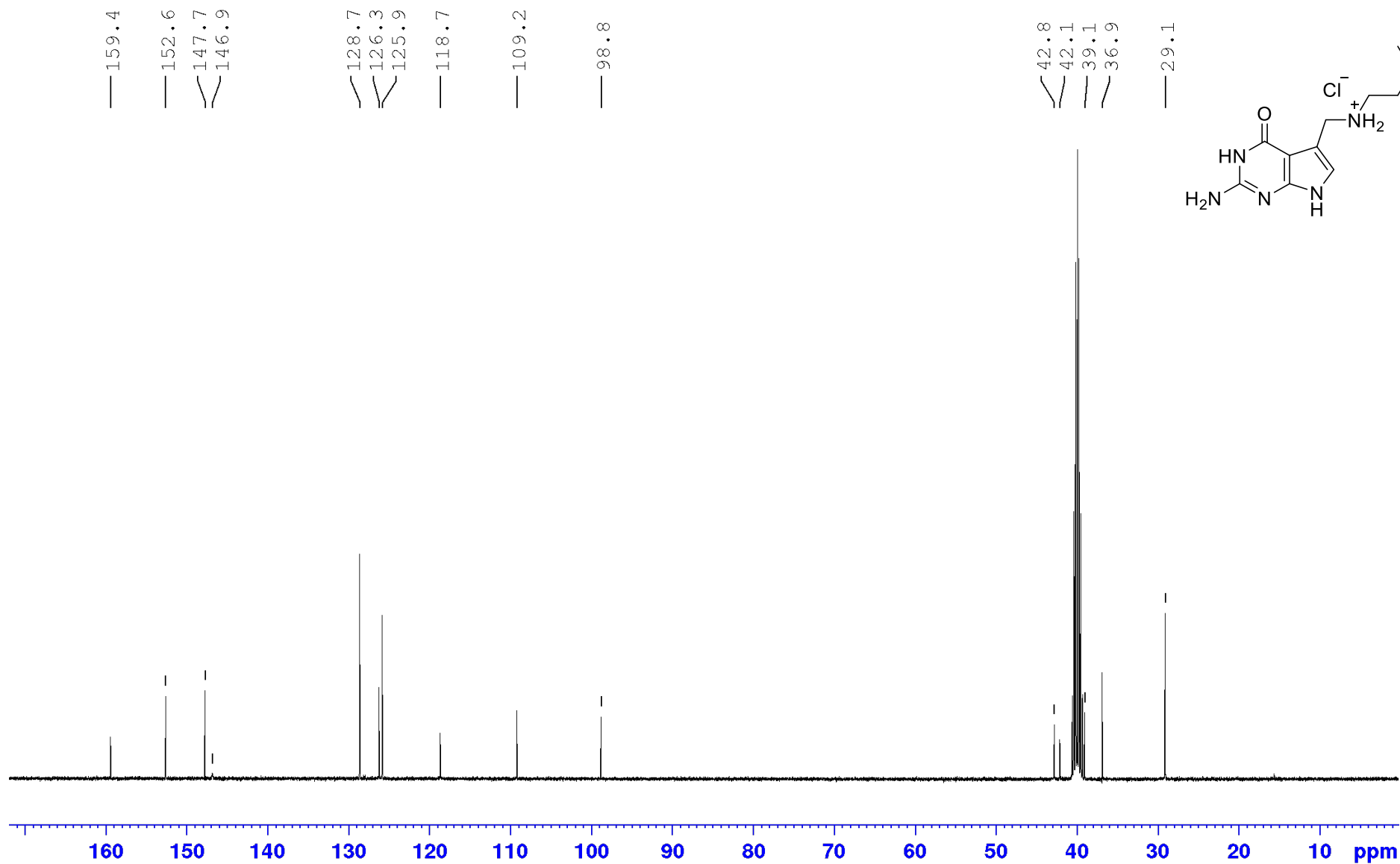

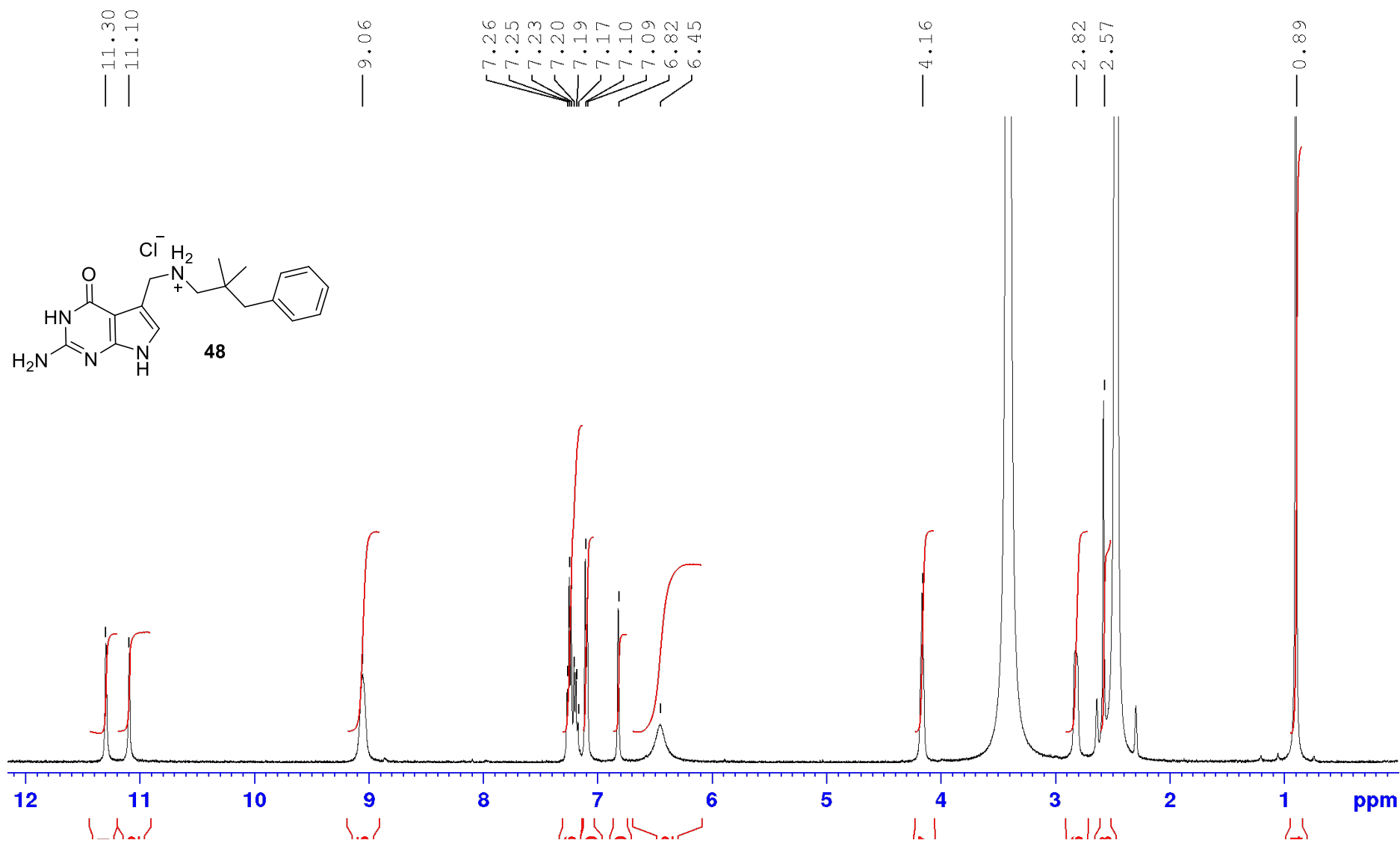

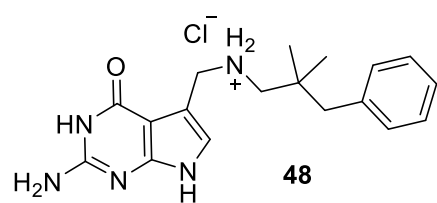

— 160.2

— 152.9

— 149.1

— 137.6

— 130.9

— 128.3

— 126.7

— 118.5

— 109.1

— 99.1

— 56.3

— 45.5

— 44.3

— 34.2

— 24.8

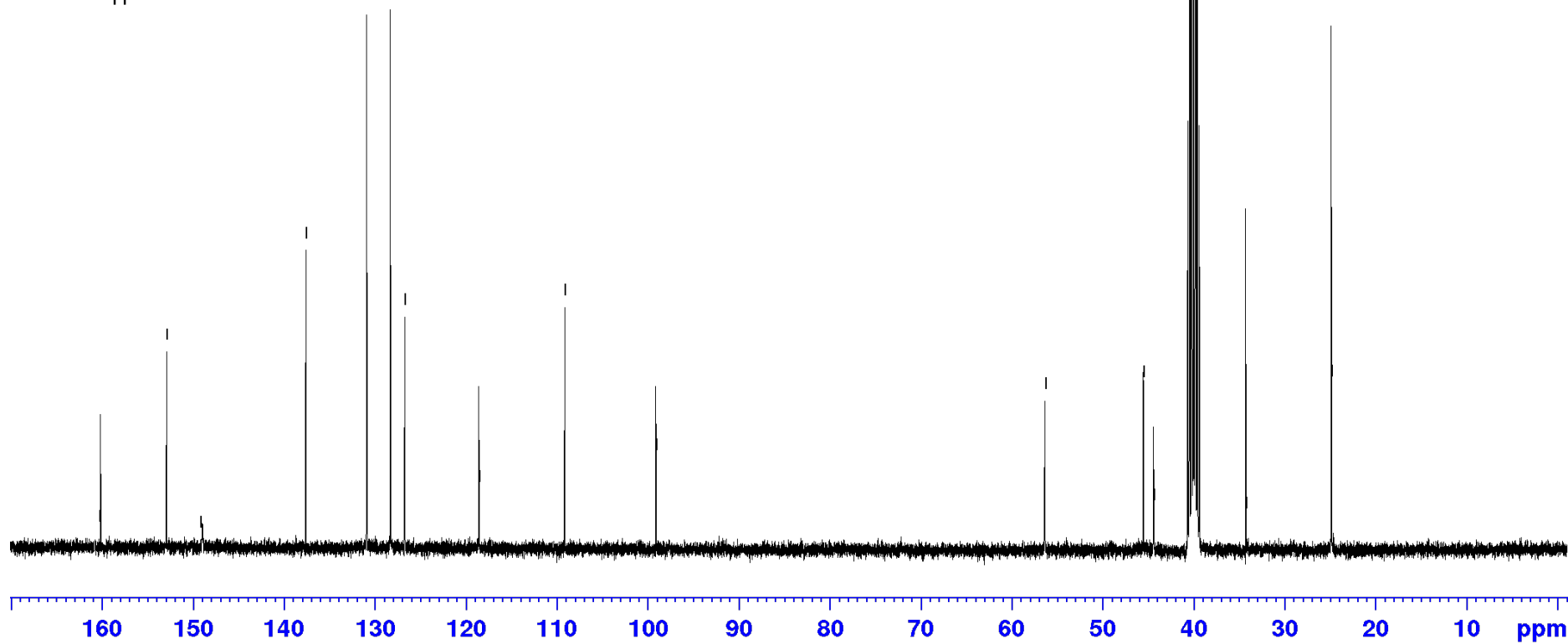

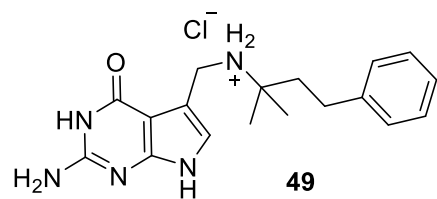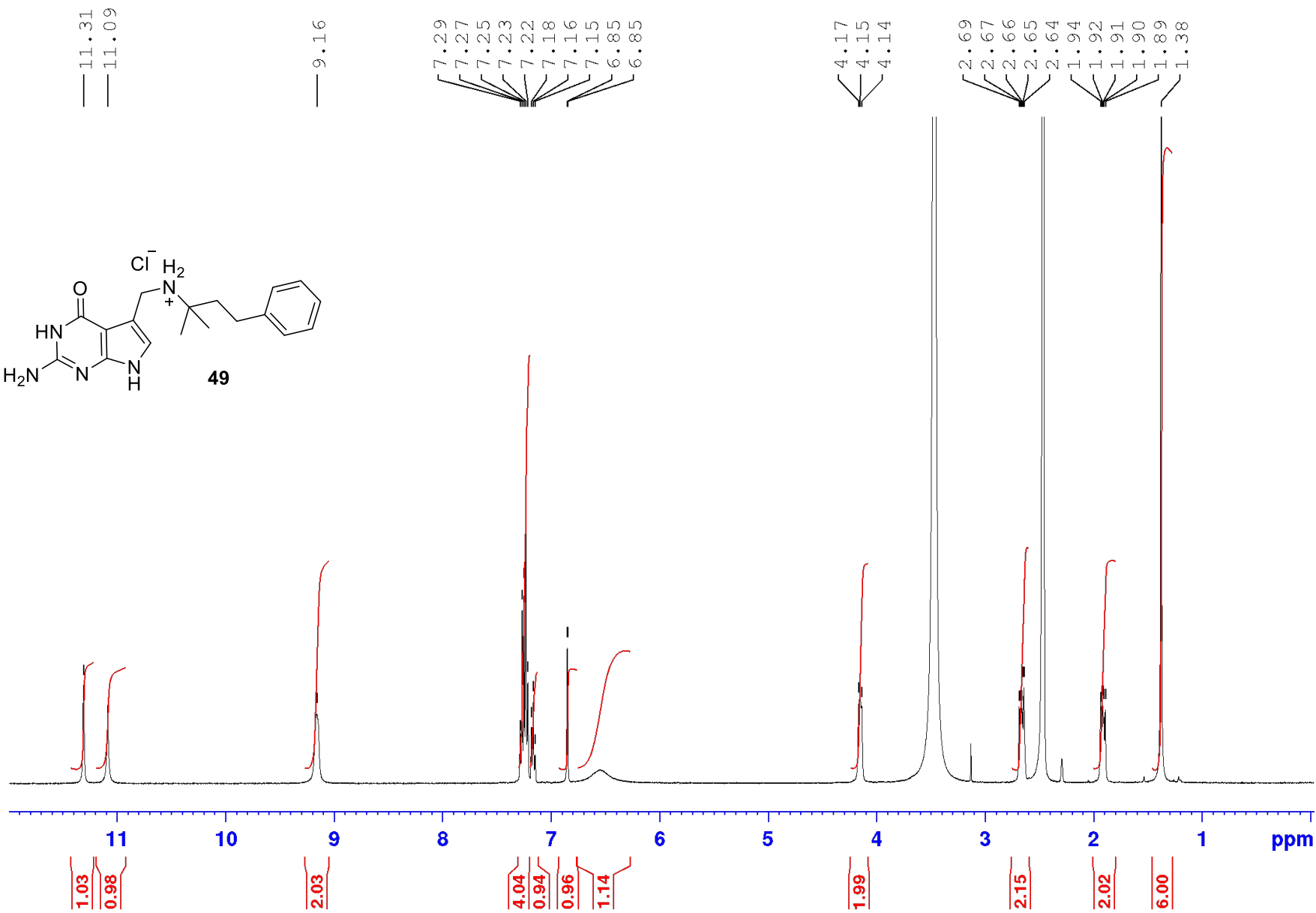

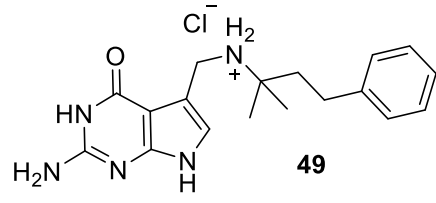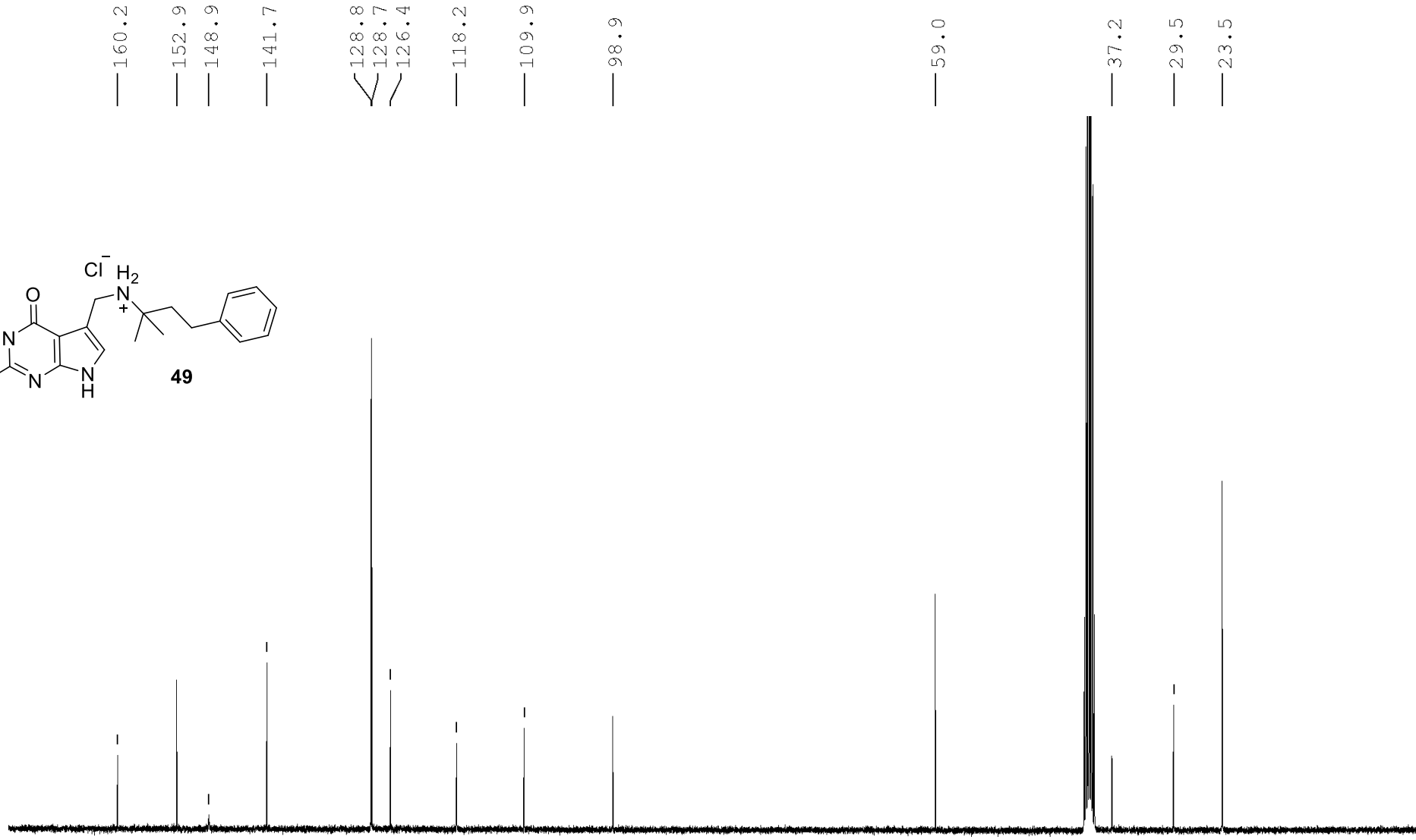

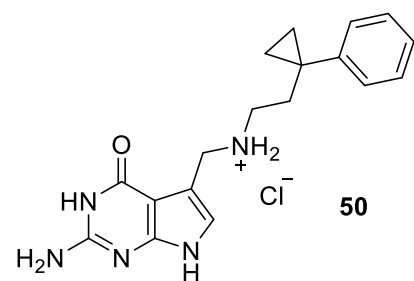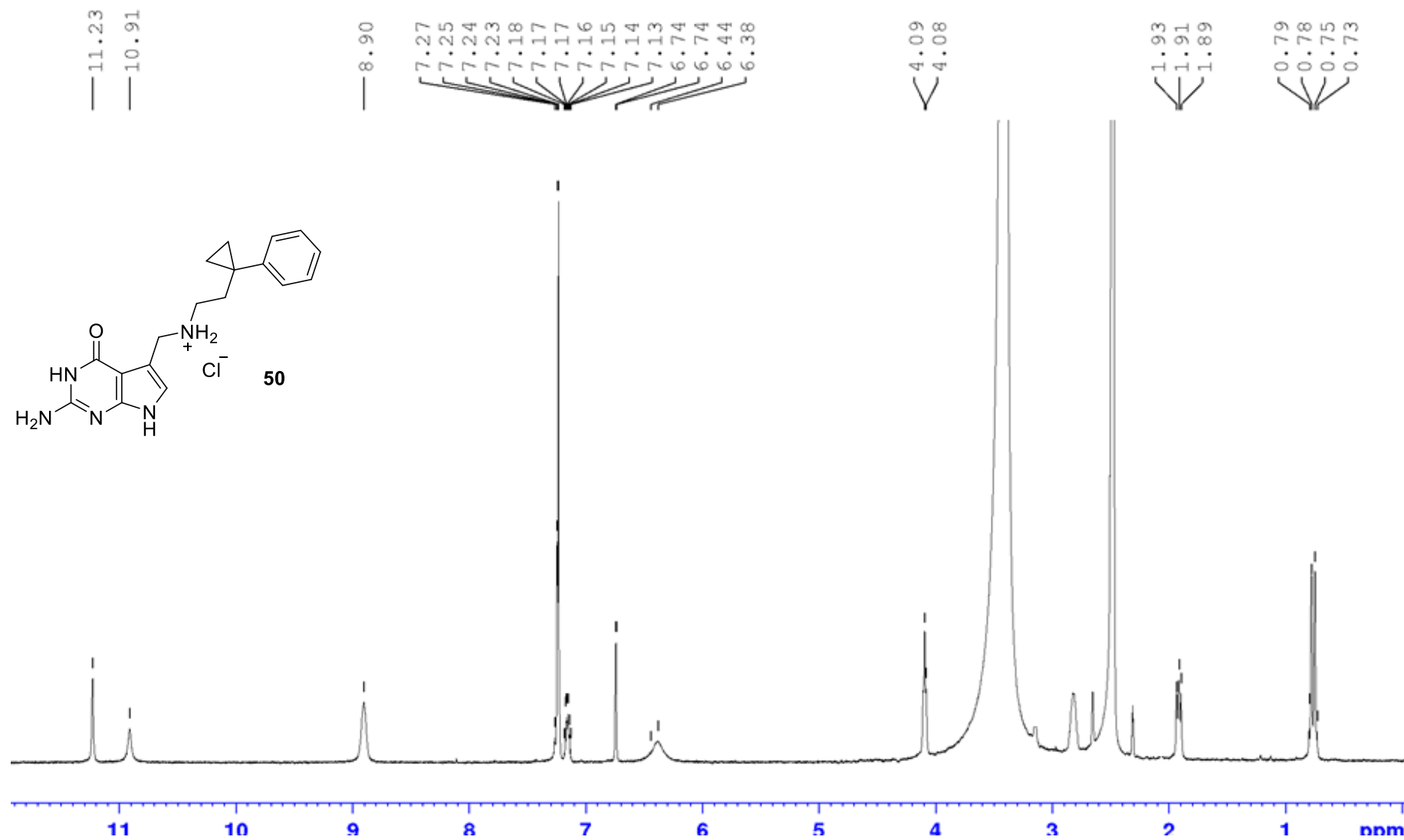

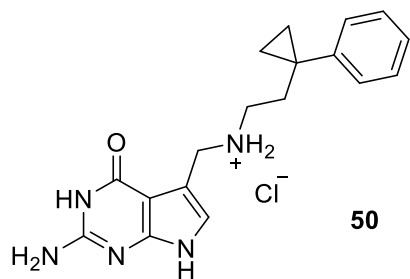

50

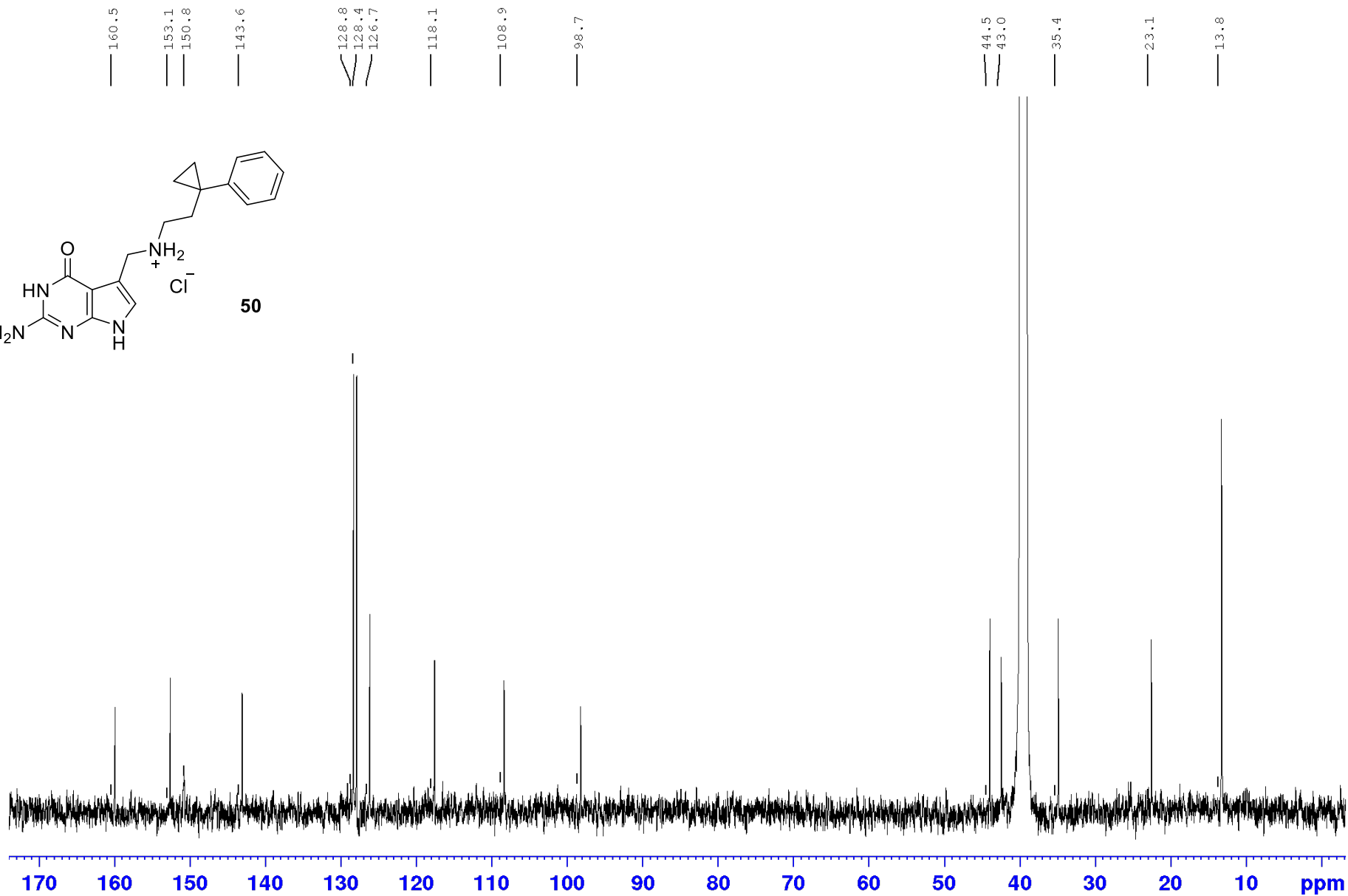

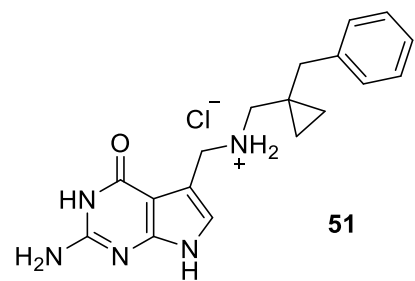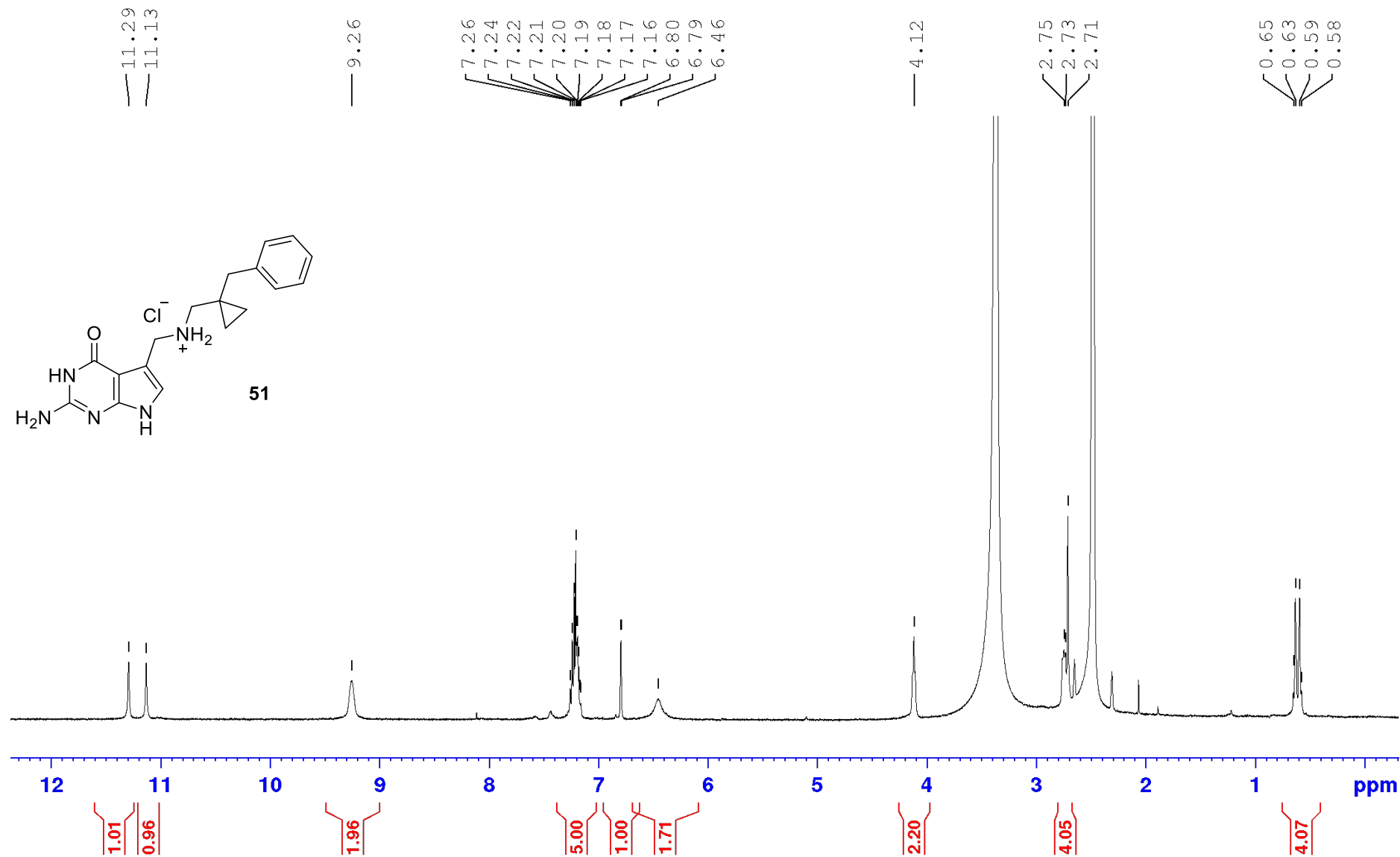

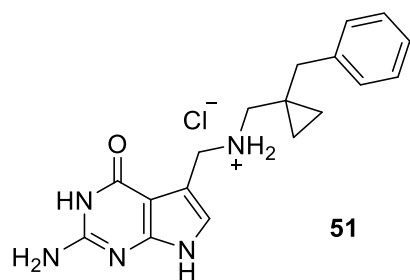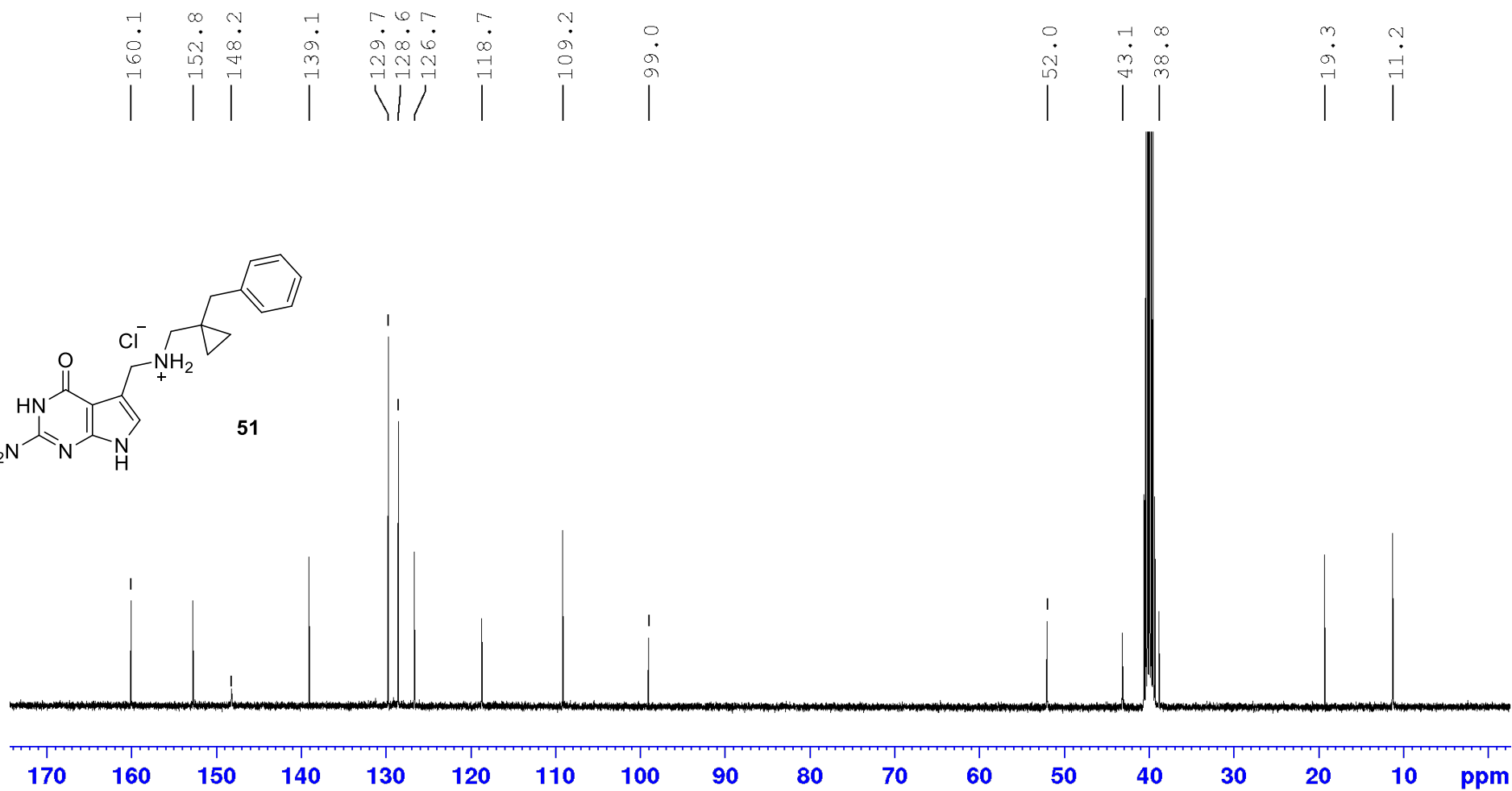

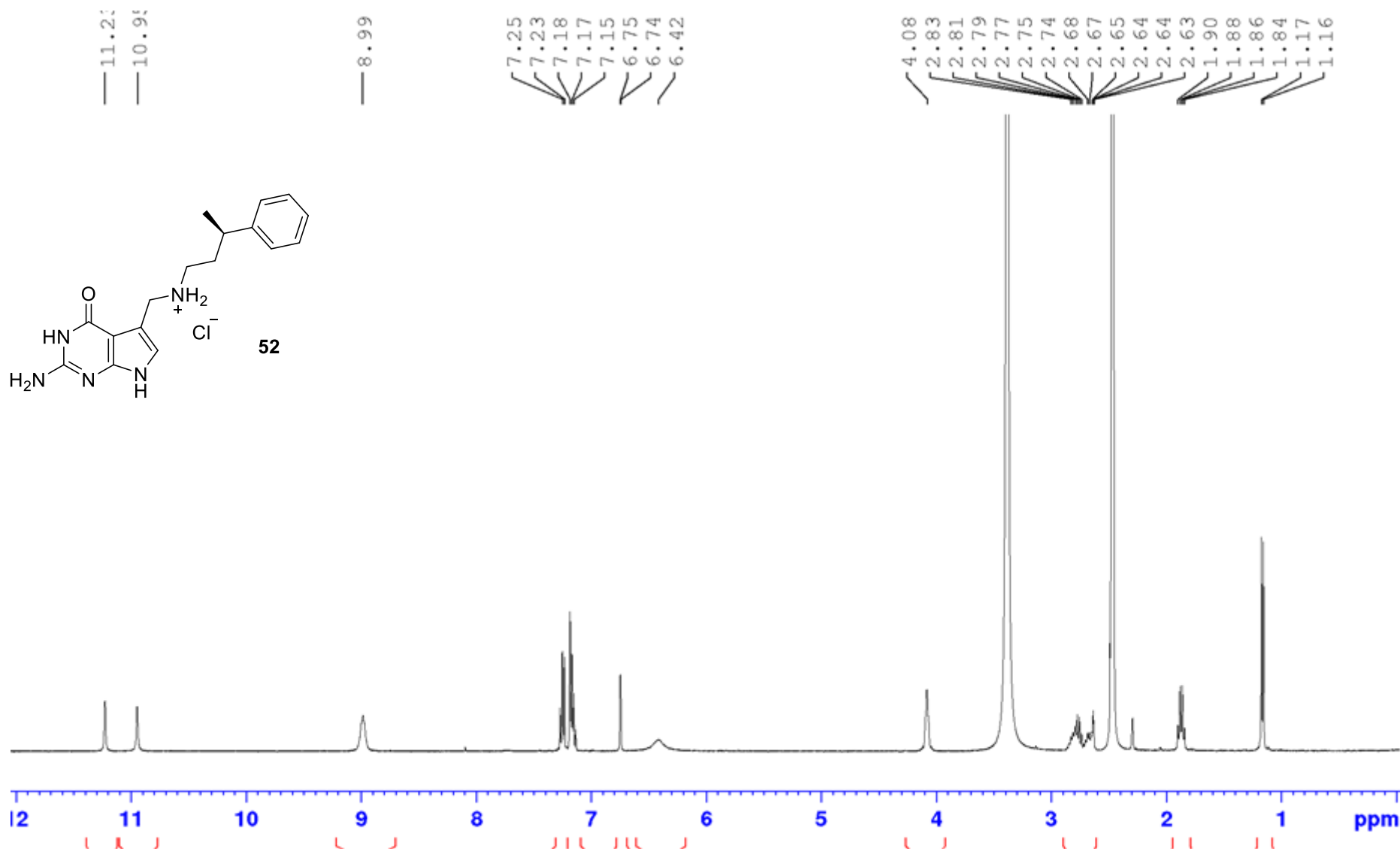

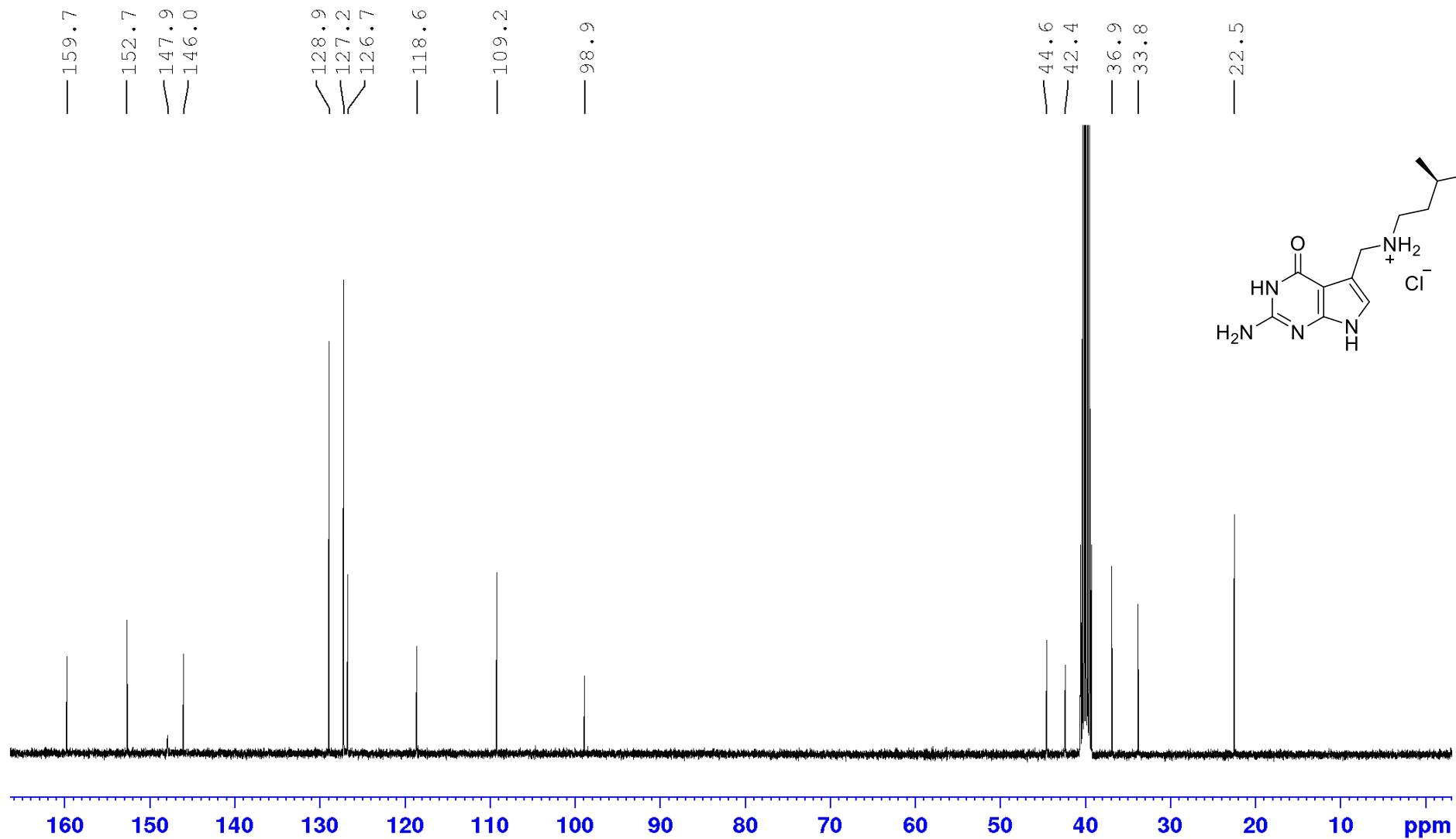

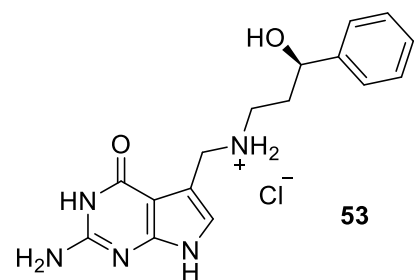

**53**

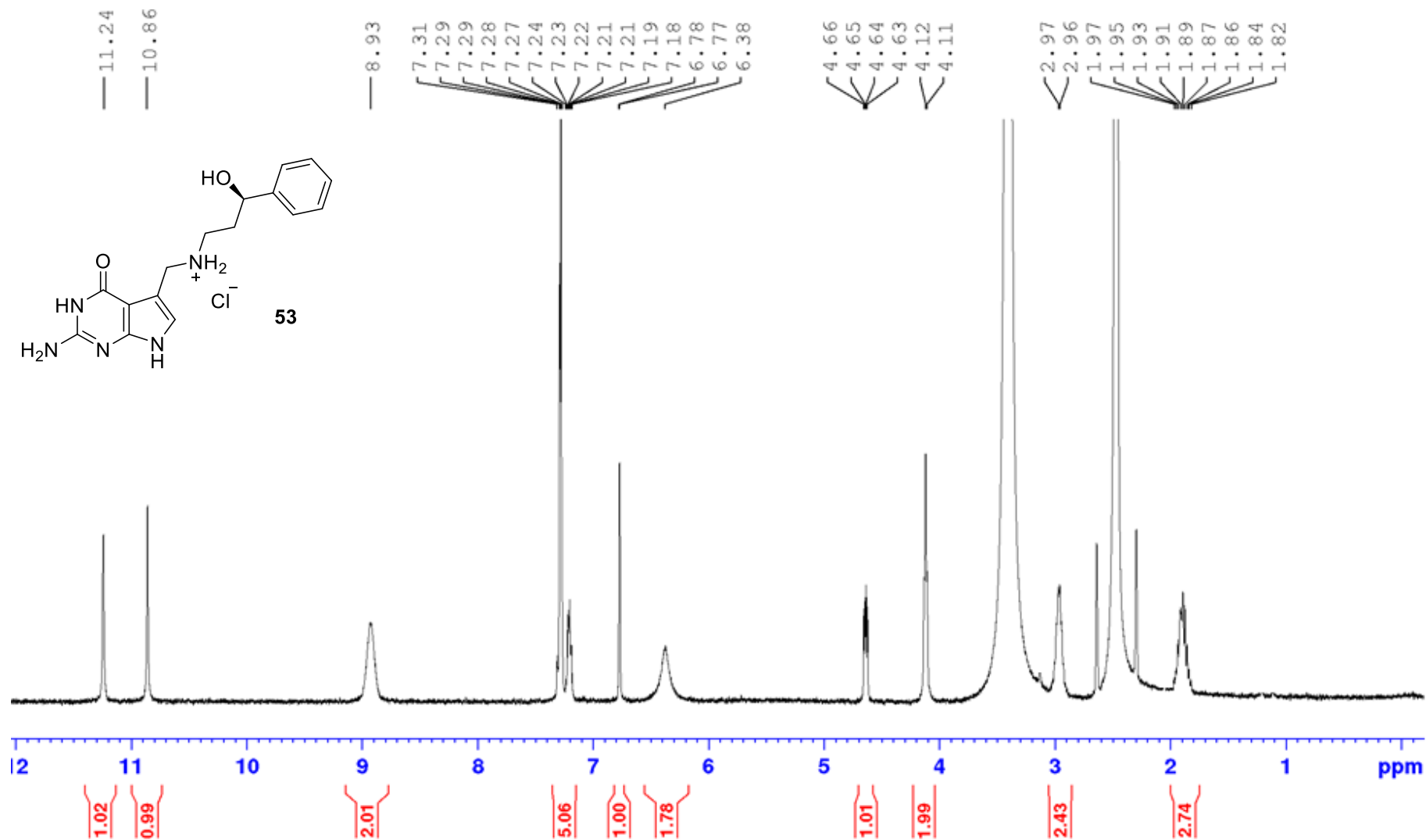

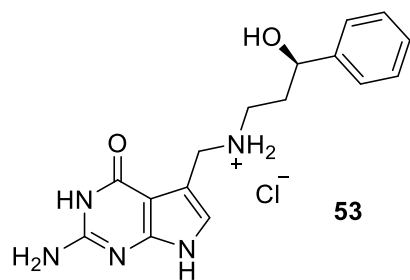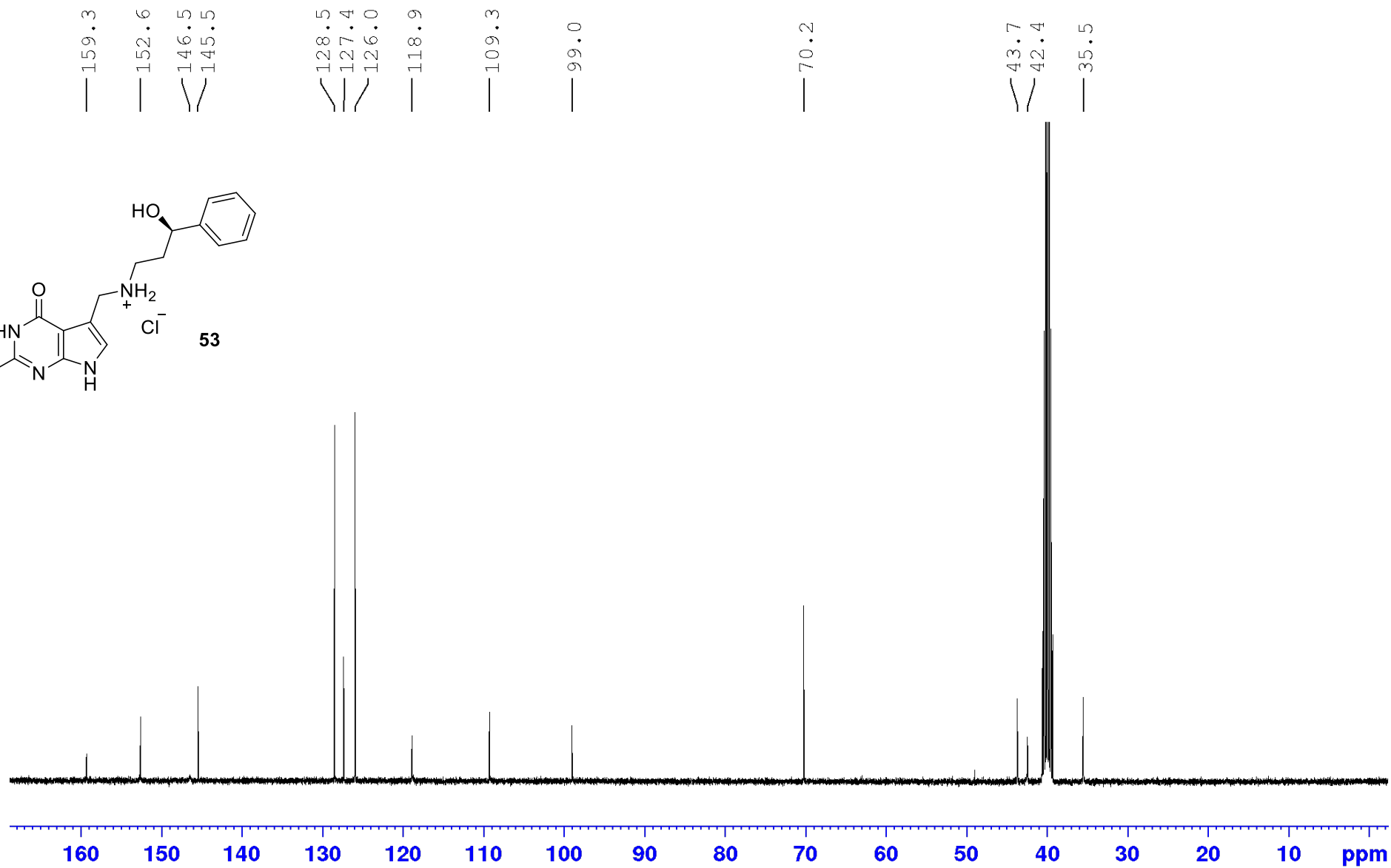

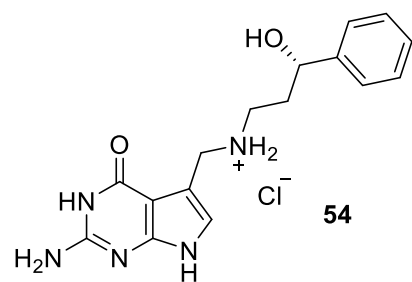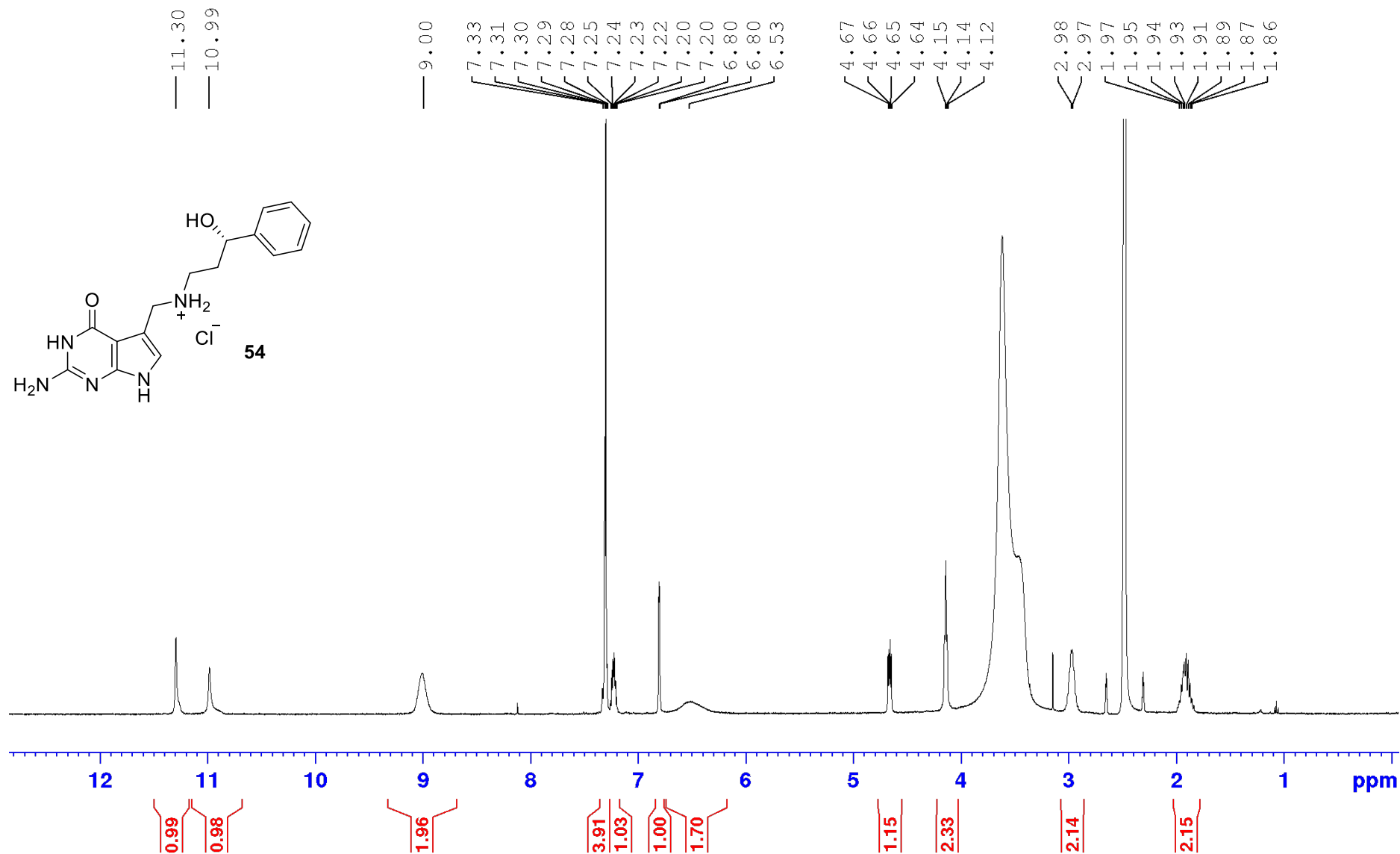

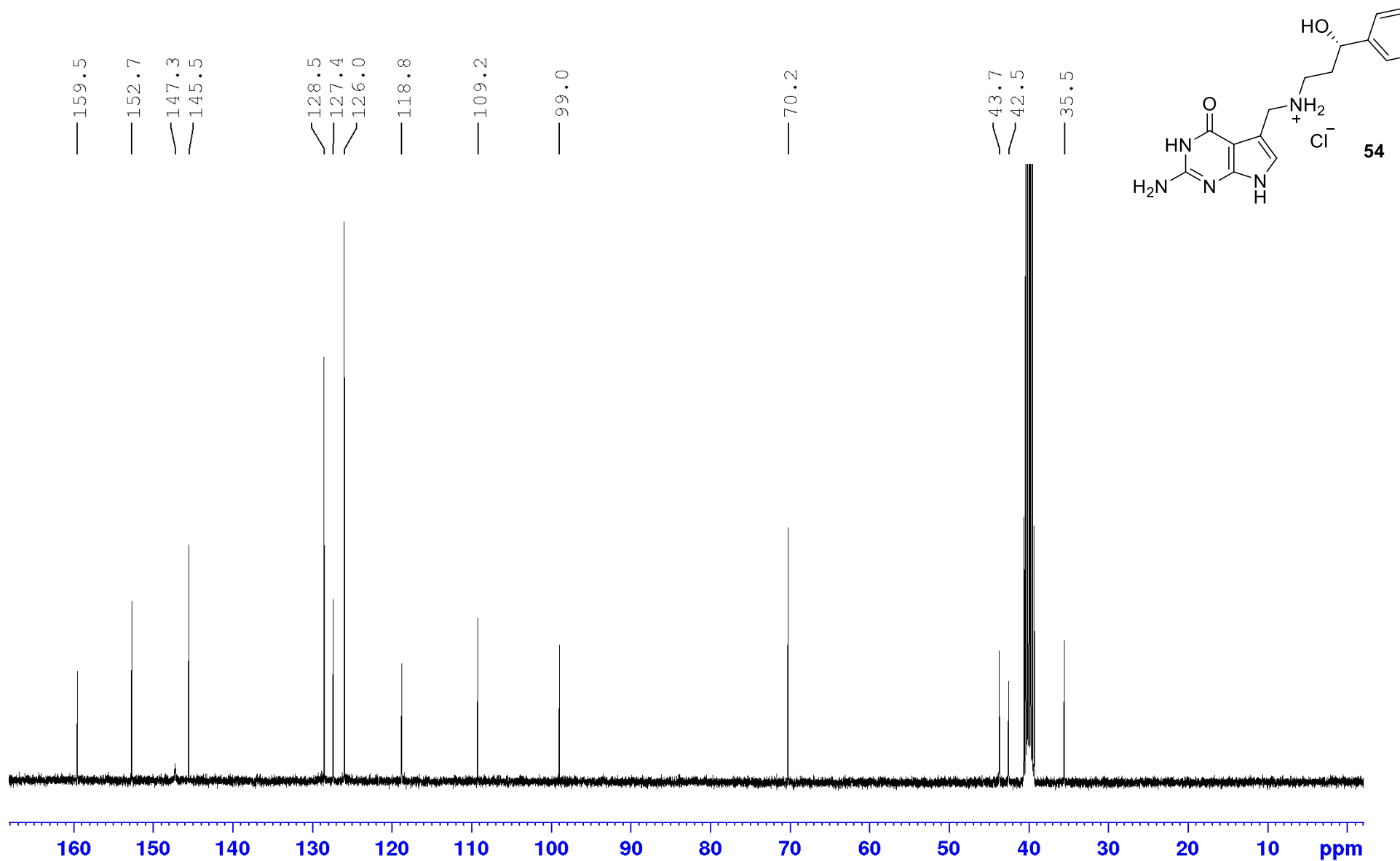

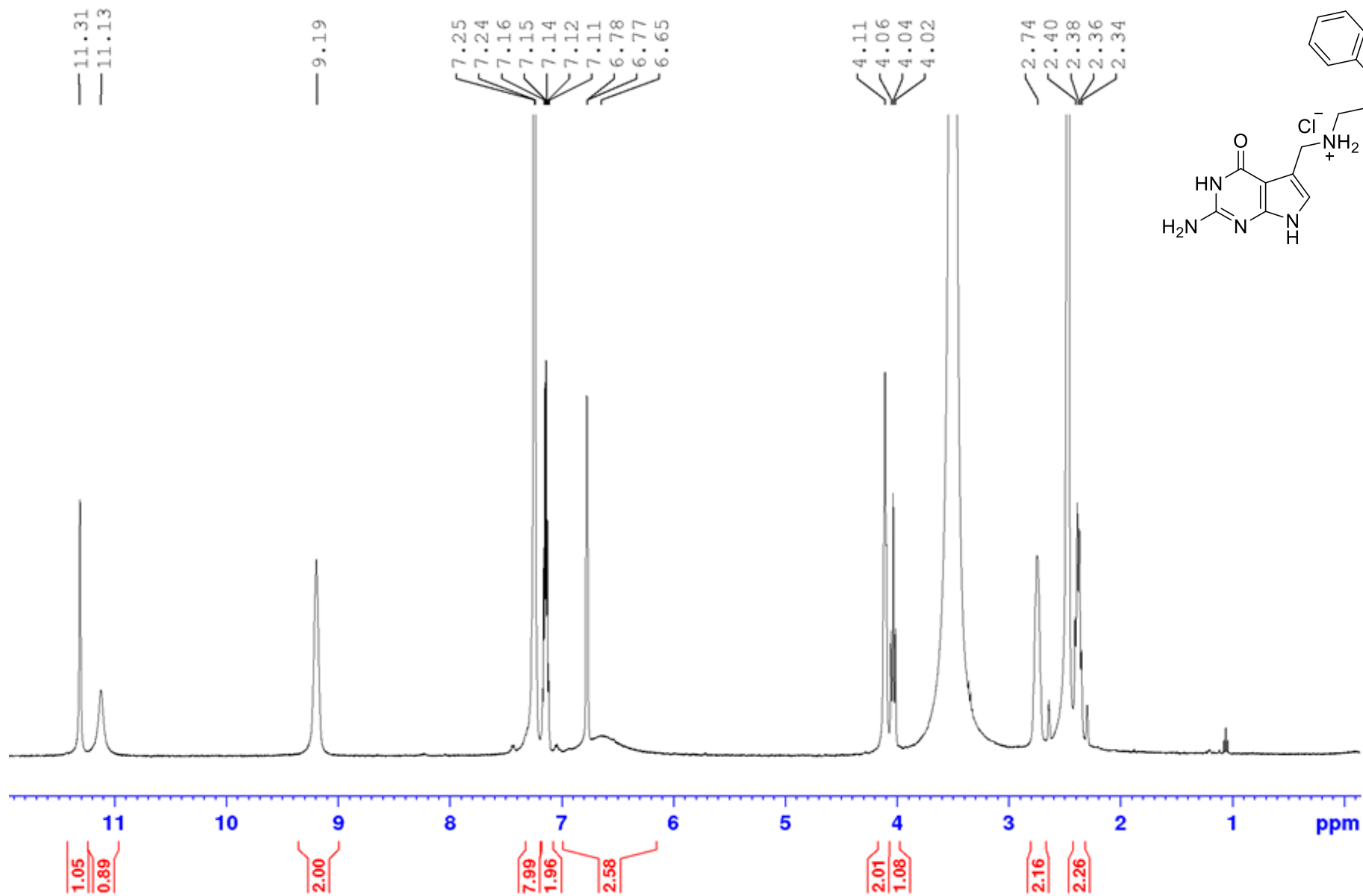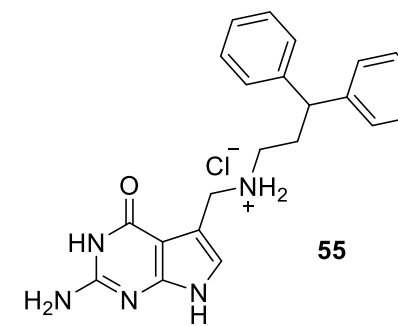

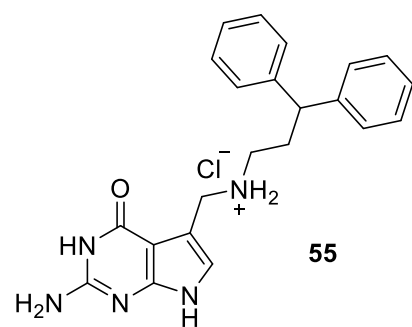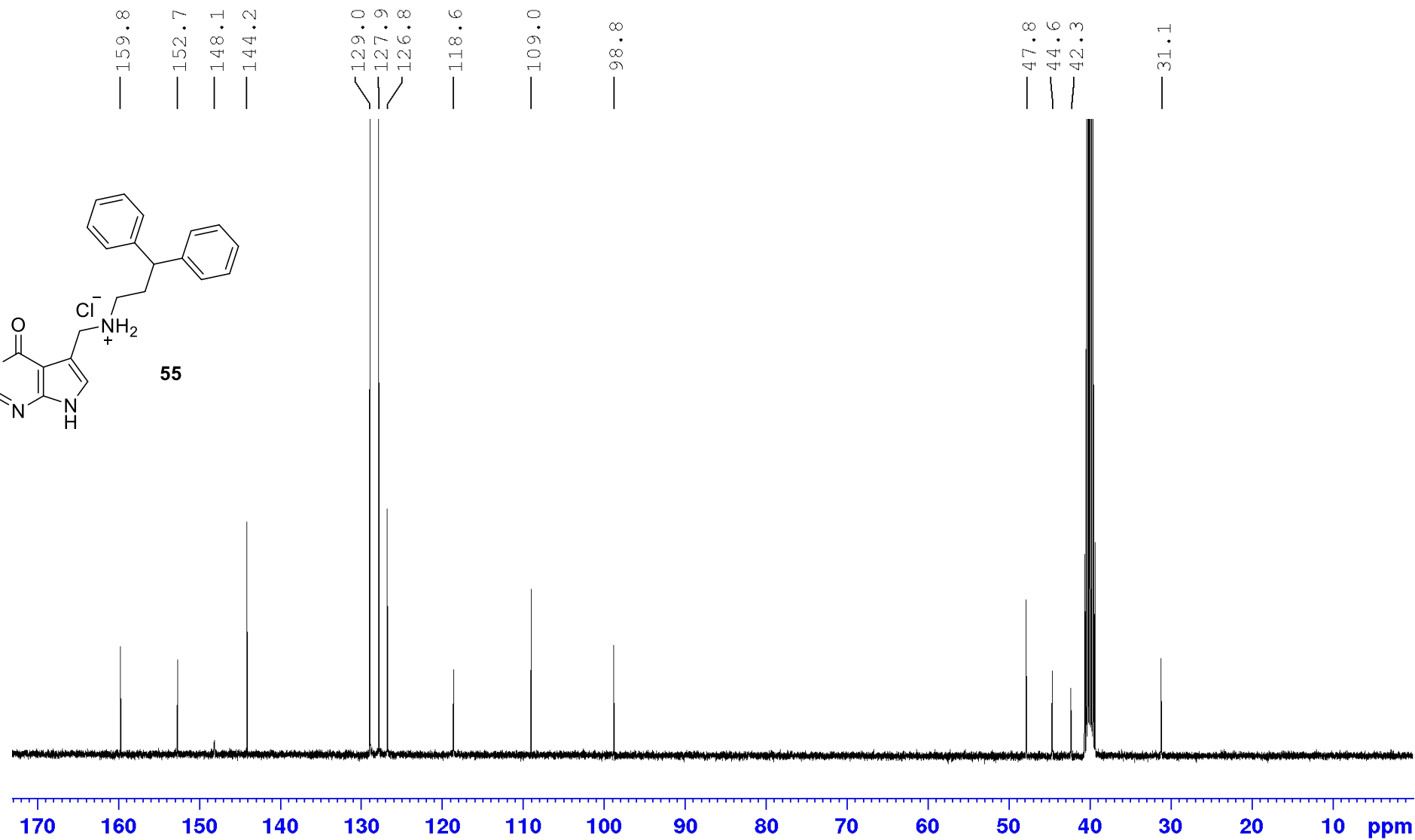



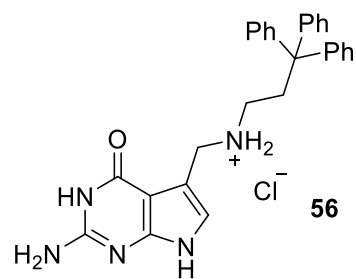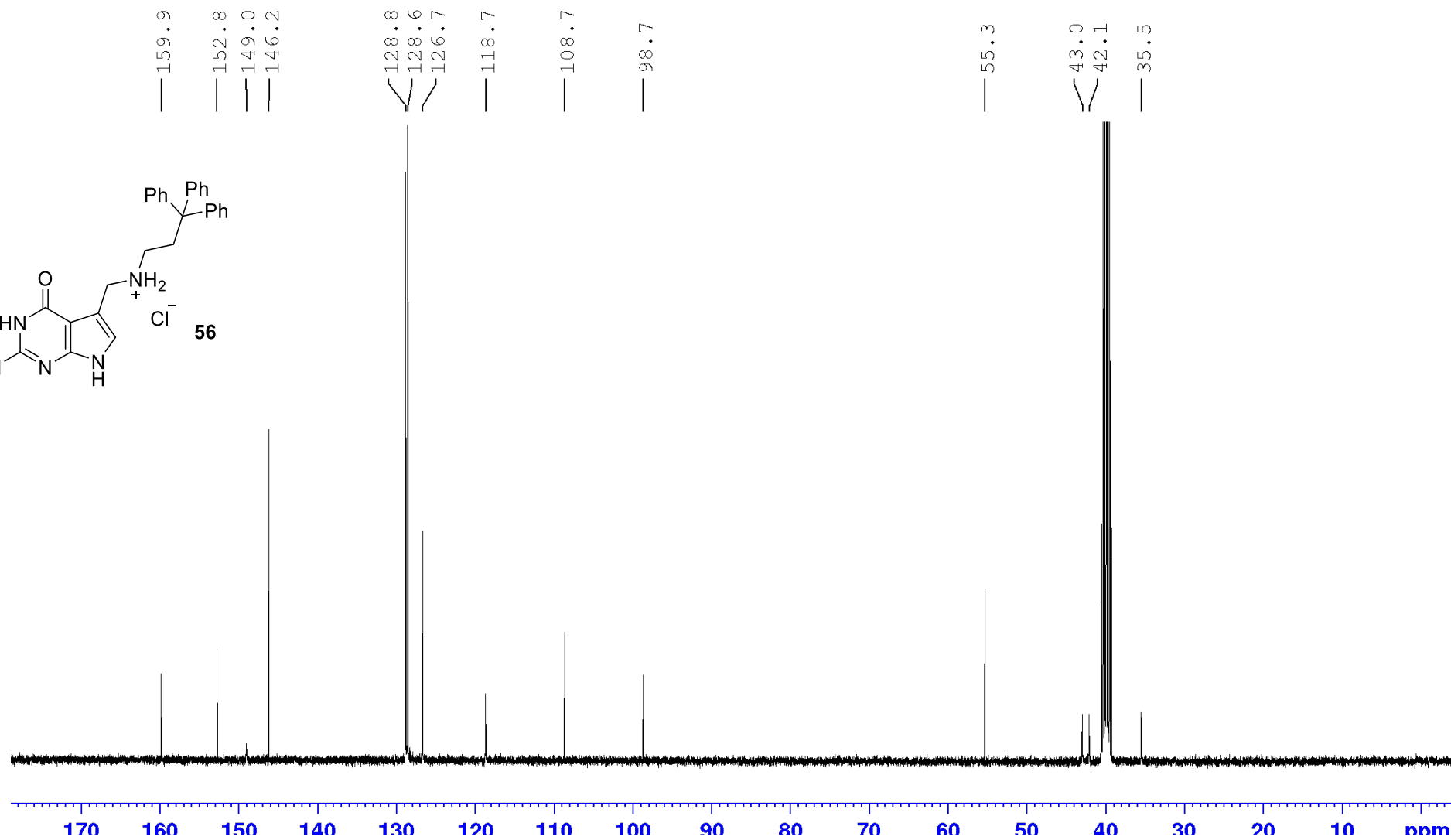

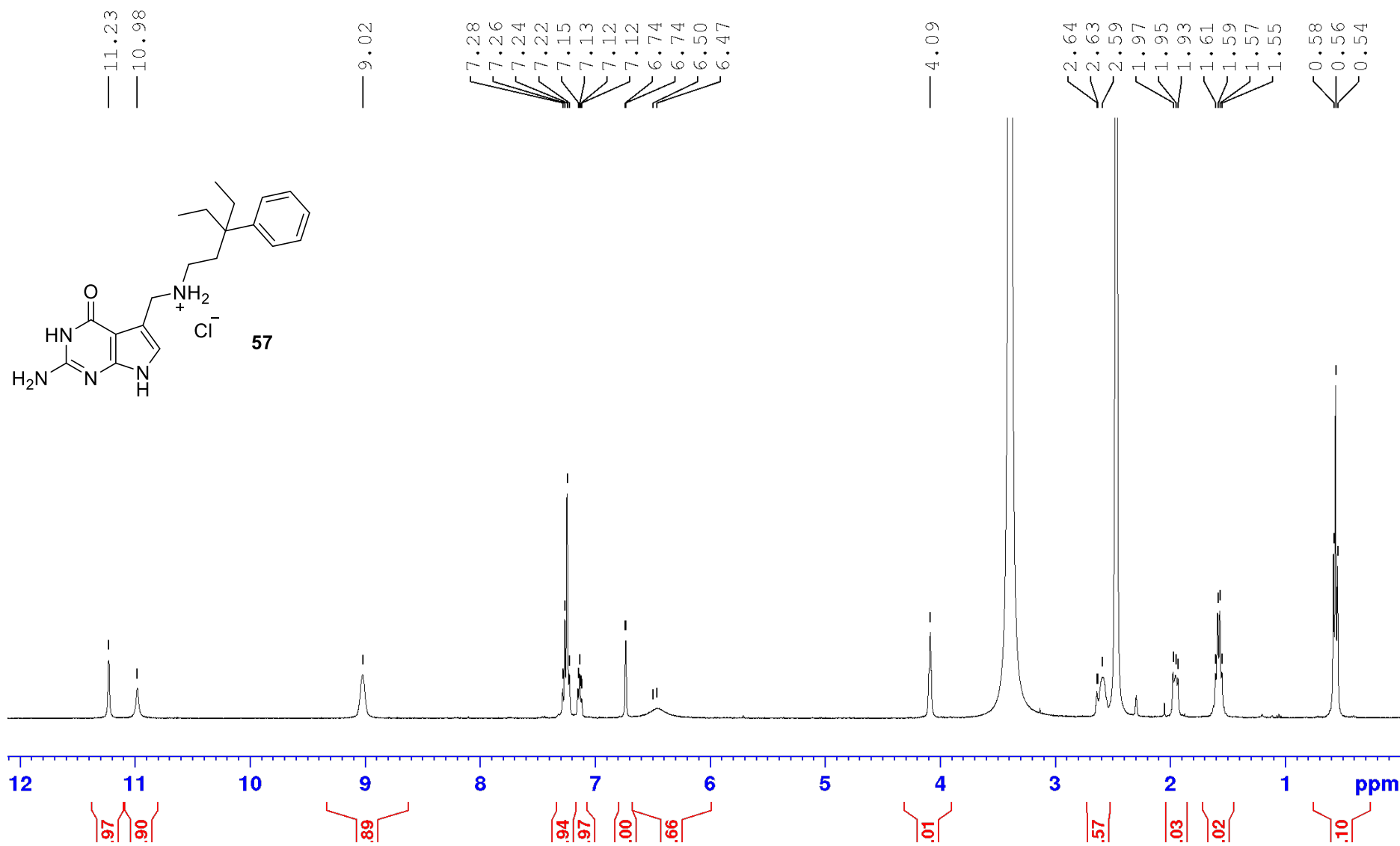

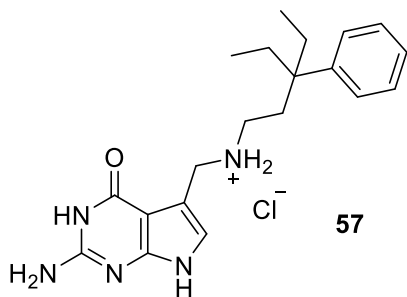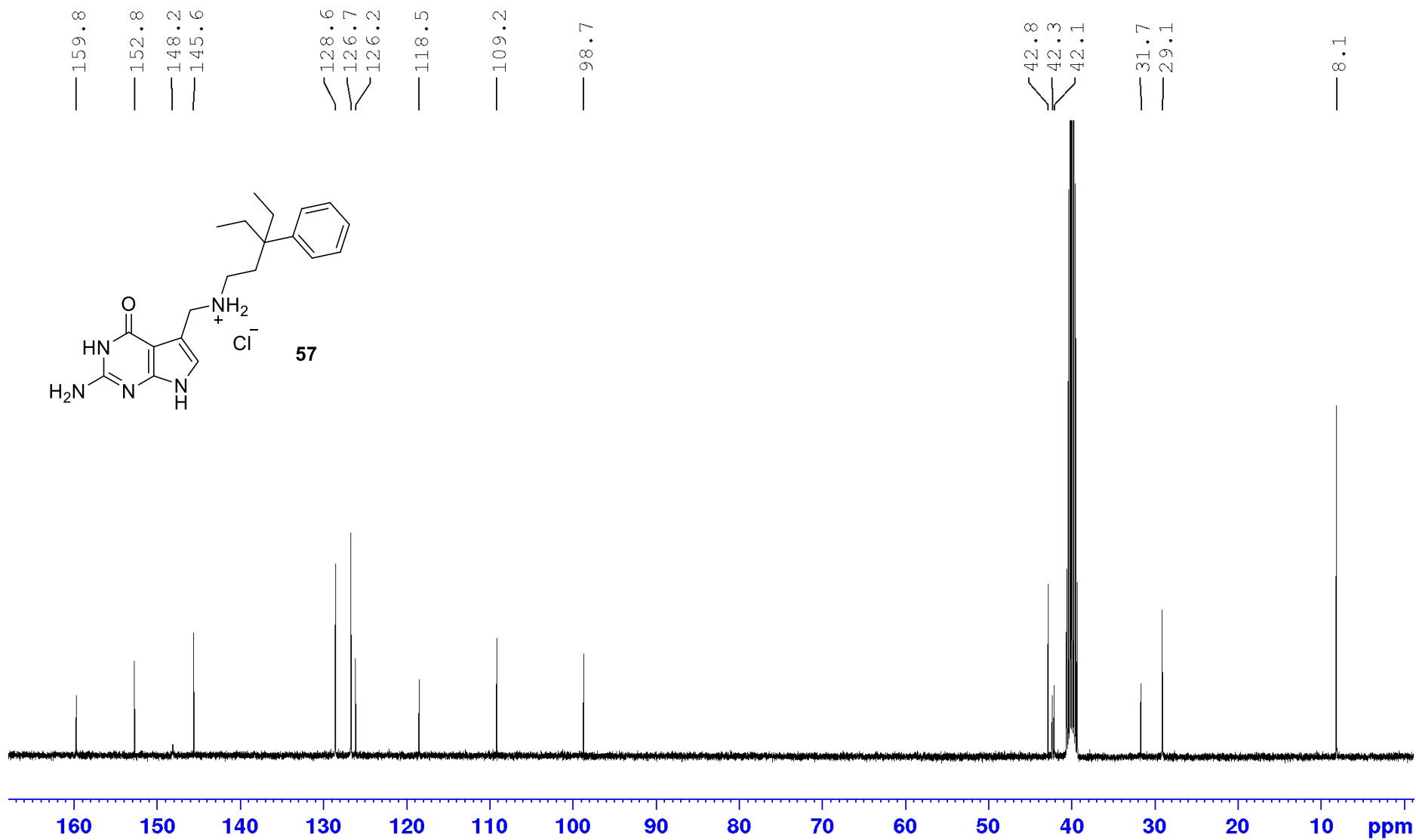

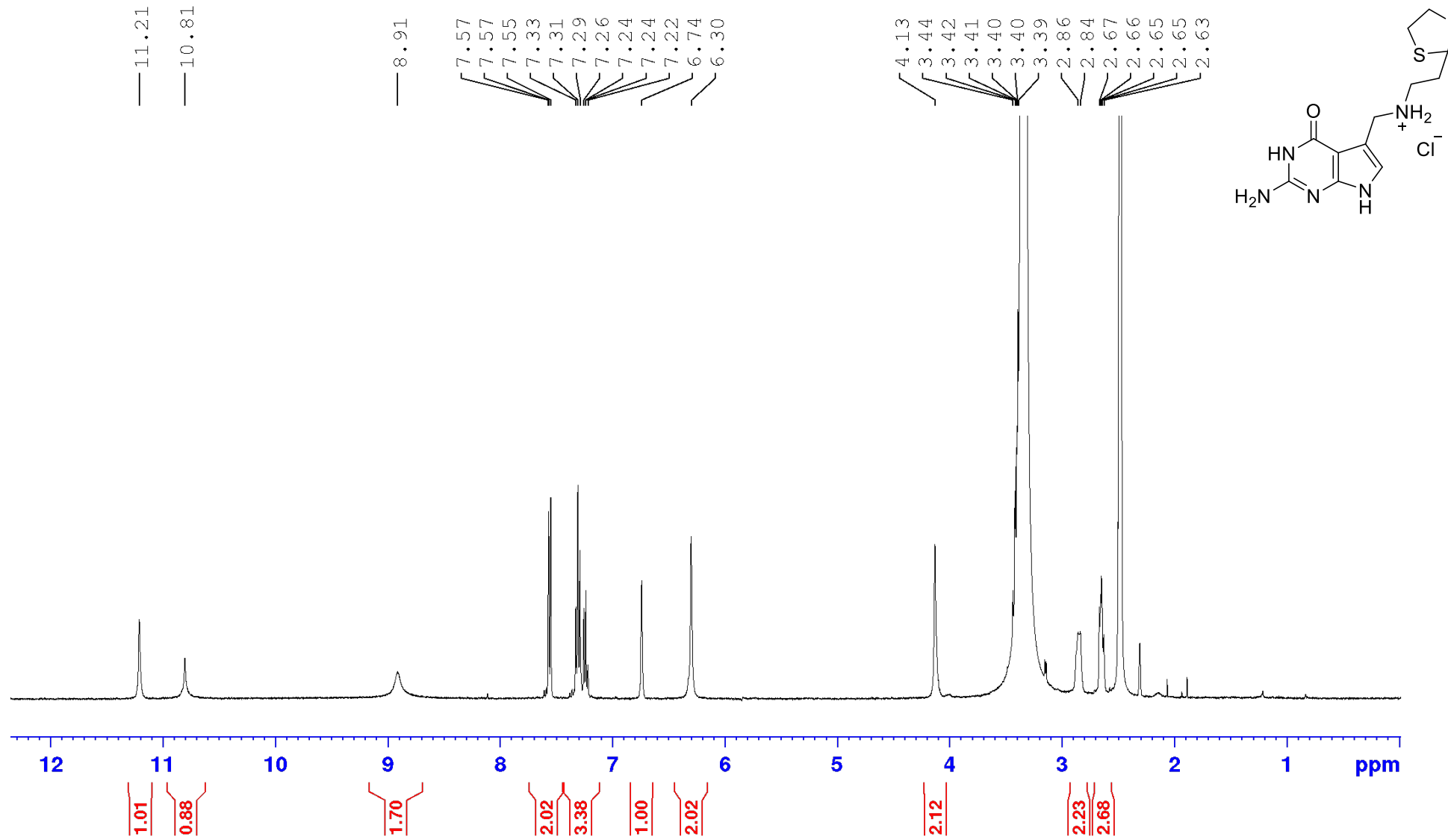

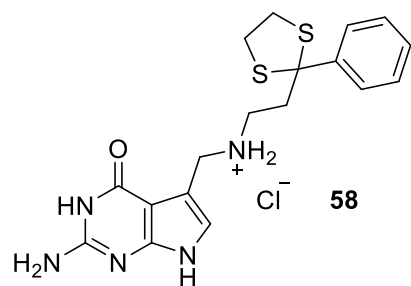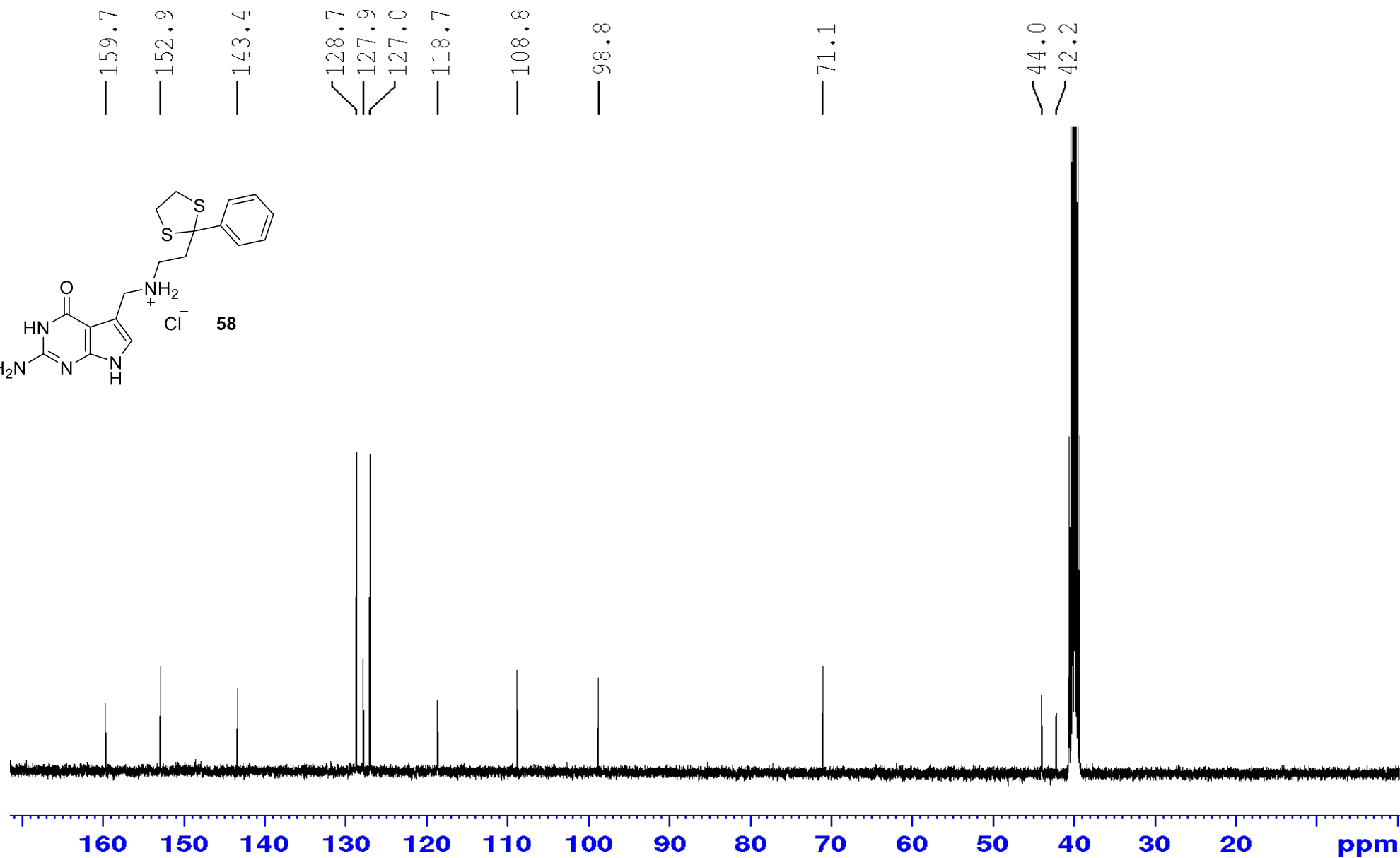

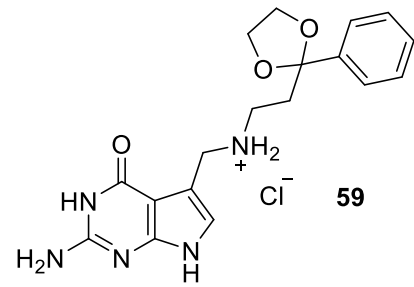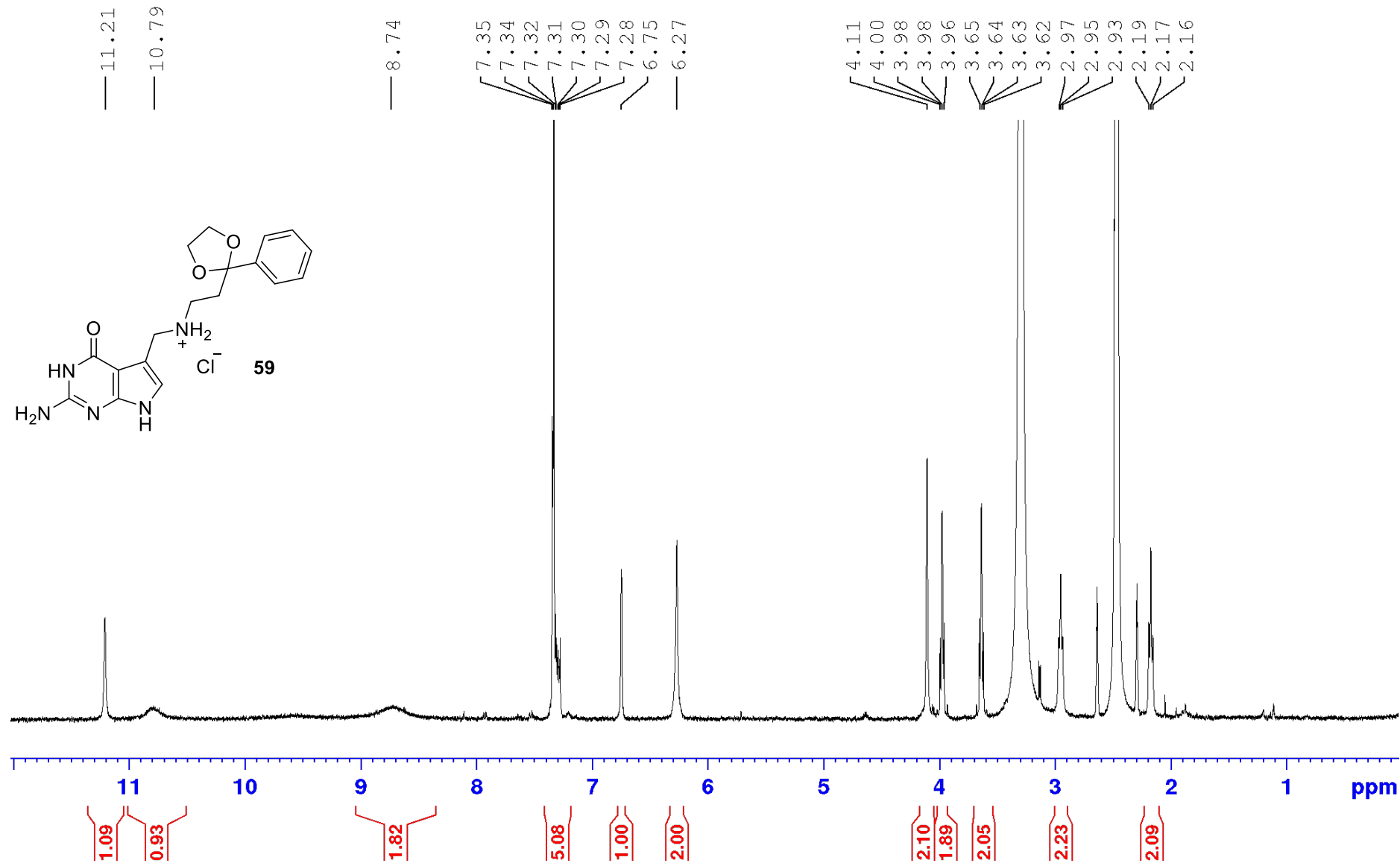

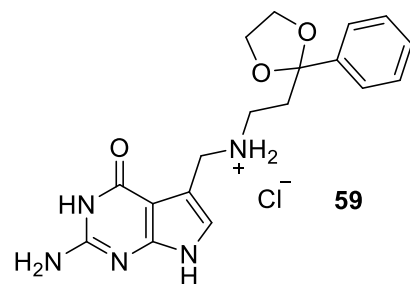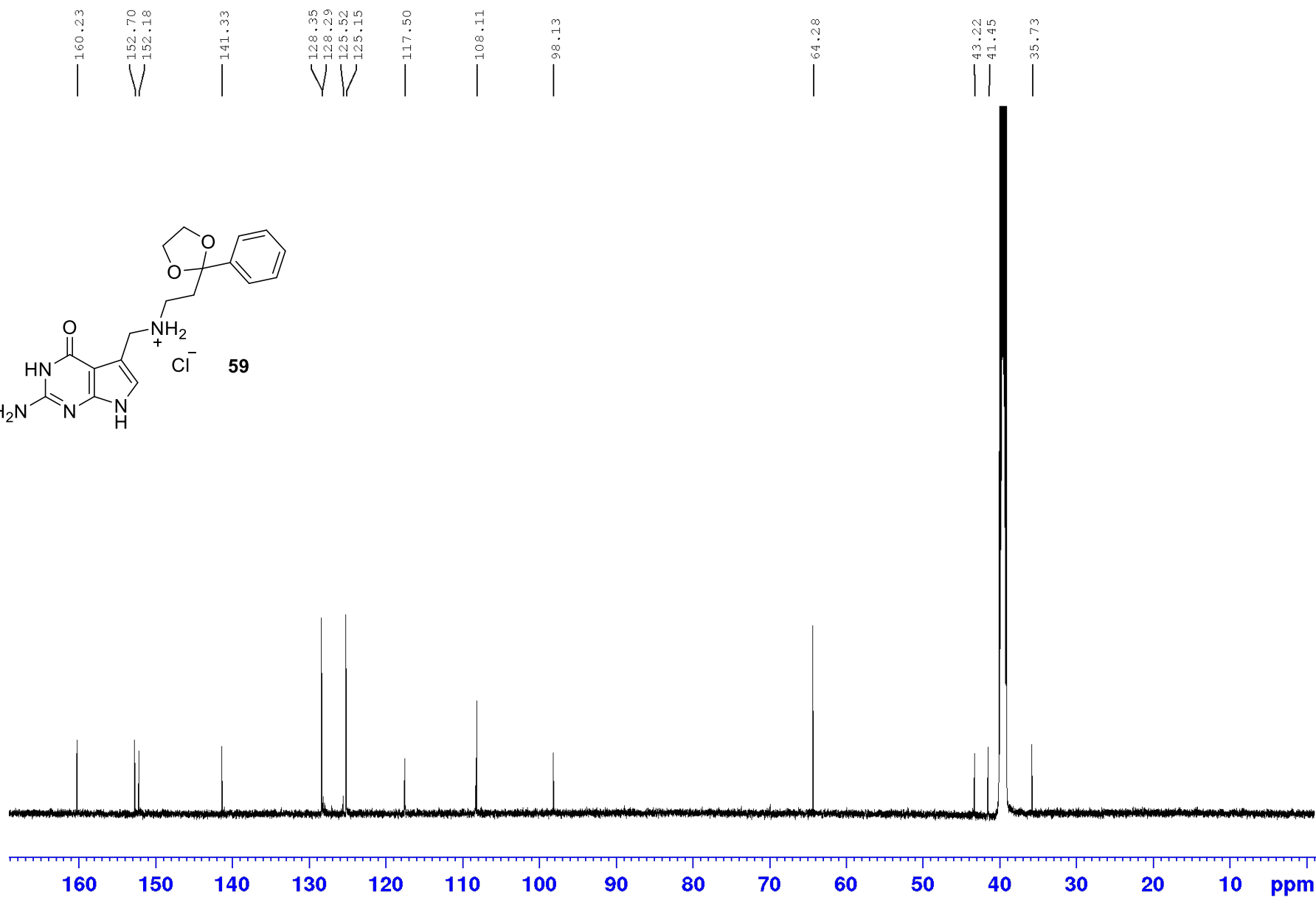

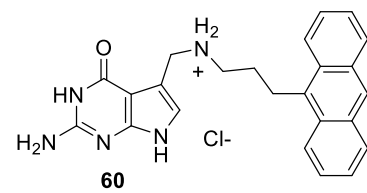

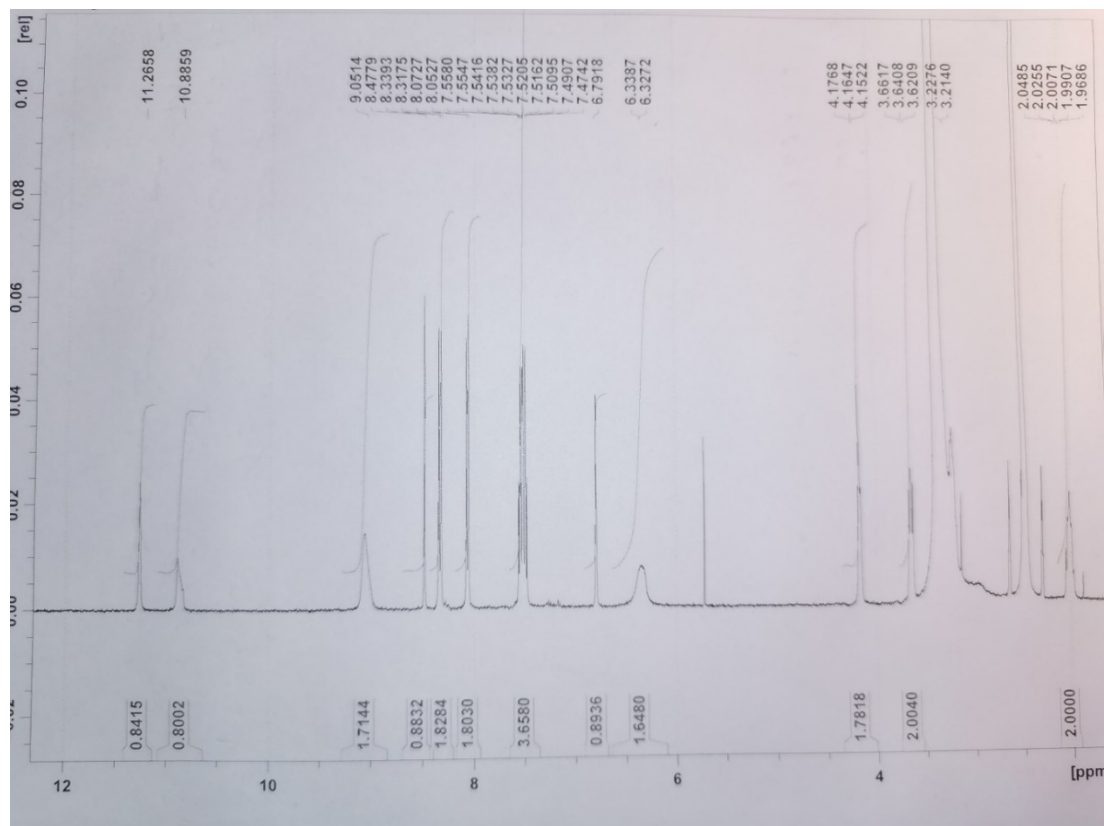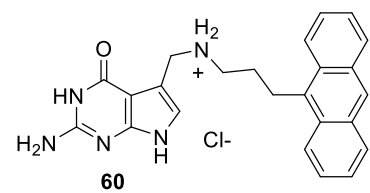

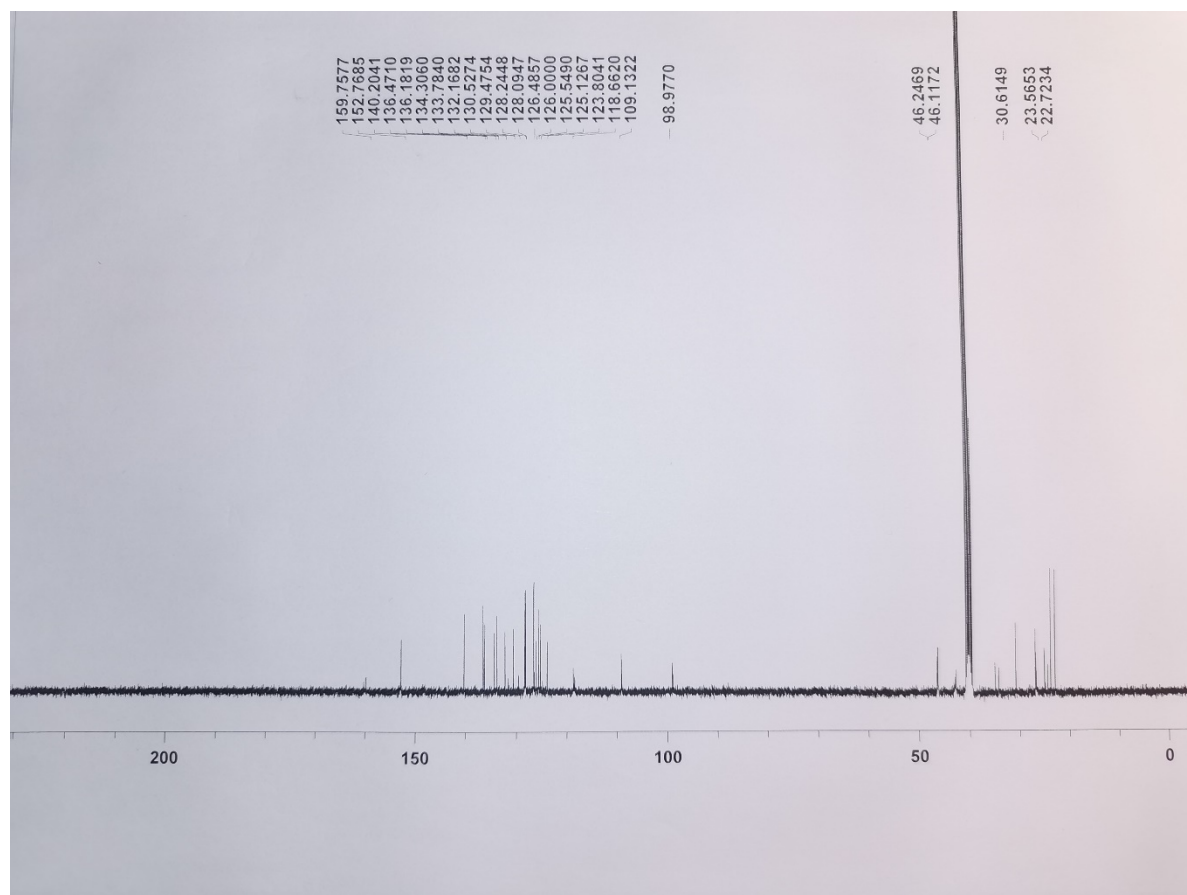

Supplement: gkab289_Supplemental_Files [file gkab289_supplemental_files.zip › 2c. Supplementary Data S3.pdf]
